# Supplementary material for: Steric control of signaling bias in the immunometabolic receptor GPR84
Source: bioRxiv. 2025 Aug 2:2025.07.30.667614. Preprint. [Version 1] doi: 10.1101/2025.07.30.667614 (PMC12324463; doi:10.1101/2025.07.30.667614)
Supplement: Supplement 1 [file media-1.pdf]

Supporting information for

Steric control of signaling bias in the immunometabolic receptor GPR84

Authors and affiliations:

Pinqi Wang[1,2,8], Xuan Zhang[3,8], Abdul-Akim Guseinov[4,5,8], Laura Jenkins[5,8], Carl von Hallerstein[6,8], Jonathan D. Colburn[6], Rowan Ives[1,2,7], Vincent B. Luscombe[7], Sara Marsango[5], Listiana Oktavia[1,2], Arun Raja[1,2], David R. Greaves[7], Philip C. Biggin[6], Graeme Milligan[5*], Cheng Zhang[3*], Irina G. Tikhonova[4*] & Angela J. Russell[1,2*]

[1] Department of Chemistry, Chemistry Research Laboratory, University of Oxford, Mansfield Road, Oxford OX1 3TA, U.K.

[2] Department of Pharmacology, University of Oxford, Mansfield Road, Oxford OX1 3QT, U.K.

[3] Department of Pharmacology and Chemical Biology, School of Medicine, University of Pittsburgh, Pittsburgh, PA 15213, U.S.A.

[4] School of Pharmacy, Queen's University Belfast, Belfast BT9 7BL, Northern Ireland, U.K.

[5] School of Molecular Biosciences, College of Medical, Veterinary and Life Sciences, University of Glasgow, Glasgow, G12 8QQ, U.K.

[6] Department of Biochemistry, University of Oxford, South Parks Road, Oxford, OX1 3QU, U.K.

[7] Sir William Dunn School of Pathology, University of Oxford, South Parks Road, Oxford OX1 3RE, U.K.

[8] These authors contributed equally: Pinqi Wang, Xuan Zhang, Abdul-Akim Guseinov, Laura Jenkins, Carl von Hallerstein

*email: Graeme.Milligan@glasgow.ac.uk; chengzh@pitt.edu; i.tikhonova@qub.ac.uk; angela.russell@chem.ox.ac.uk

General information

All reactions involving moisture sensitive reagents were carried out under a nitrogen atmosphere. Anhydrous solvents were dried by passing over an activated alumina column, under an inert atmosphere, using a solvent purification system. All other solvents and reagents were used as supplied (analytical or HPLC grade) without prior purification. Flash column chromatography was performed on Kieselgel 60 silica gel (230-400 mesh particle size) on a glass column or on a Biotage SP4 automated flash column chromatography platform. NMR spectra were recorded on Bruker Advance spectrometers at 400, 500 or 600 MHz in the deuterated solvent stated at room temperature. The field was locked by external referencing to the relevant deuteron resonance. Chemical shifts (δ) are reported in parts per million (ppm) and coupling constants (*J*) are quoted in Hz. Data are reported as follows: chemical shift, multiplicity (s = singlet, d = doublet, t = triplet, q = quartet, sext = sextuplet, hept = heptet, and m = multiplet), coupling constant and integration. Low-resolution mass spectra (*m/z*) were recorded on an Agilent 1260 Infinity II with Diode Array and Single Quadrupole Detectors in solutions of MeOH. A selected peak is reported in Daltons and its intensity given as percentage of the base peak. High resolution mass spectra (HRMS) were run on a Bruker microTOF (ESI and APCI) or on a Waters GCT (EI). Experiments conducted in CROs used their standard equipment. The purity for test compounds was determined by HPLC on a Shimadzu SIL-20AC HT instrument. HPLC conditions were as follows: Atlantis dC18, 100 Å, 5 μm, Column 4.6 × 150 mm, 35−100% MECN in water, 15 min run, flow rate 1.5 mL/min, UV detection (λ = 220, 254, 280 nm). The mass spectra were obtained using Agilent 6120 bench-top single quadrupole instrument using electrospray ionization (ESI).

Reaction Schemes

Scheme 1. Synthesis of **OX04539**[a]

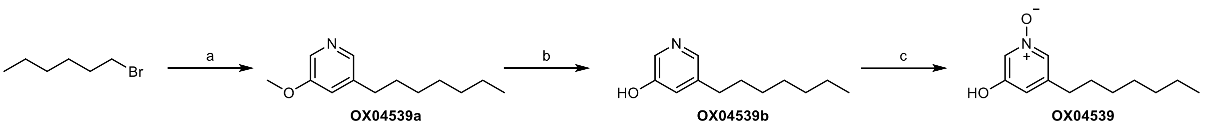

[a]Reagents and conditions: (a) (1) PPh_3, Toluene, reflux, 16h, 63%; (2) 5-methoxynicotinaldehyde, NaOtBu, dichloromethane, 0ºC–rt, 91%; (3) Pd/C, H_2, MeOH, rt, 1 h, 99%; (b) L-selectride, THF, 70ºC, 72 h, 39%; (c) *m*-CPBA, CH_2Cl_2, rt, 2 h, 55%.

Scheme 2. Synthesis of **OX04540**[a]

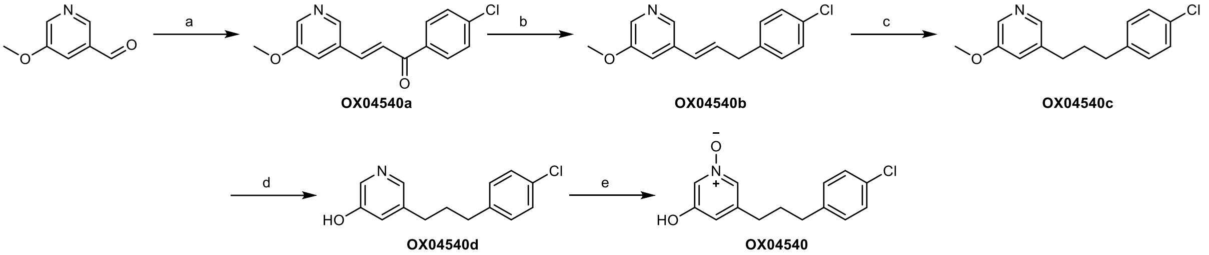

[a]Reagents and conditions: (a) 1-(4-chlorophenyl)ethan-1-one, $BF_3\cdot Et_2O$, 1,4-dioxane, 105ºC, 16 h, 70%; (b) Et_3SiH, TFA, rt, 48 h, 59%; (c) Pd/C, H_2, MeOH, rt, 1 h, 79%; (d) 48% HBr, 120ºC, 48 h, 23%; (e) *m*-CPBA, CH_2Cl_2, rt, 2 h, 28%.

Scheme 3. Synthesis of **OX04954**[a]

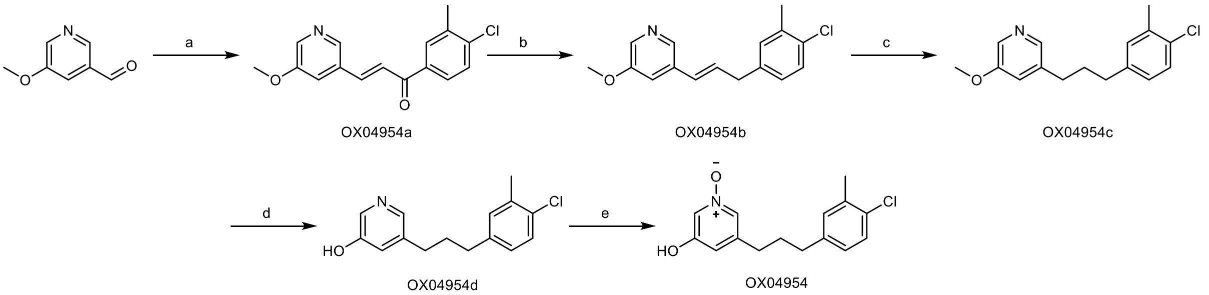

[a]Reagents and conditions: (a) 1-(4-chloro-3-methylphenyl)ethan-1-one, $BF_3\cdot Et_2O$, 1,4-dioxane, 105ºC, 16 h, 85%; (b) Et_3SiH, TFA, rt, 48 h, 82%; (c) Pd/C, H_2, MeOH, rt, 30 min, 85%; (d) 48% HBr, 120ºC, 48 h, 79%; (e) *m*-CPBA, CH_2Cl_2, rt, 2 h, 87%.

Scheme 4. Synthesis of **OX04956**[a]

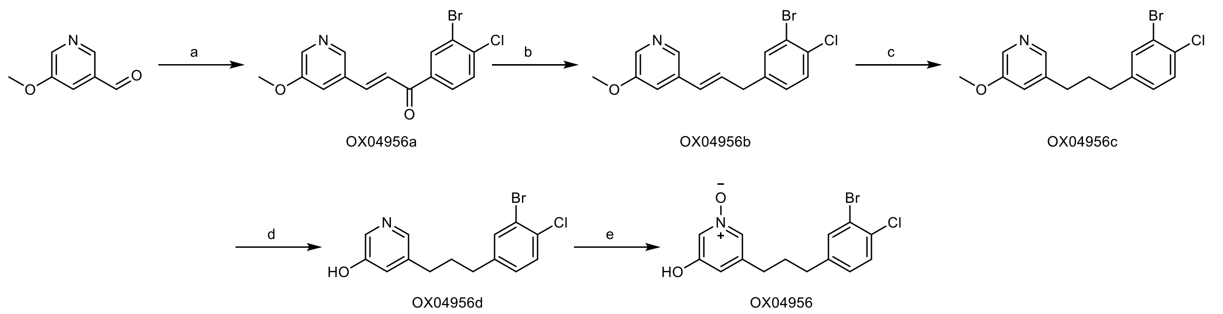

[a]Reagents and conditions: (a) 1-(3-bromo-4-chlorophenyl)ethan-1-one, $BF_3 \cdot Et_2O$, 1,4-dioxane, 105ºC, 16 h, 29%; (b) Et_3SiH, TFA, rt, 48 h, 59%; (c) PtO_2, H_2, MeOH, rt, 1 h, 85%; (d) 48% HBr, 120ºC, 48 h, 28%; (e) *m*-CPBA, CH_2Cl_2, rt, 2 h, 52%.

Scheme 5. Synthesis of **OX04955**[a]

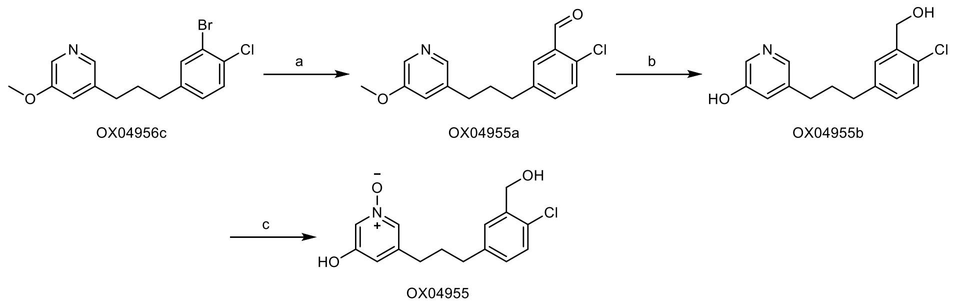

[a]Reagents and conditions: (a) (1) n-BuLi, THF, -78ºC, 5 min; (2) DMF, THF, -78ºC, 15 min, 45%; (b) L-Selectride, THF, 70ºC, 48 h, 39%; (c) *m*-CPBA, CH_2Cl_2, rt, 2 h, 64%.

Scheme 6. Synthesis of **OX04957**[a]

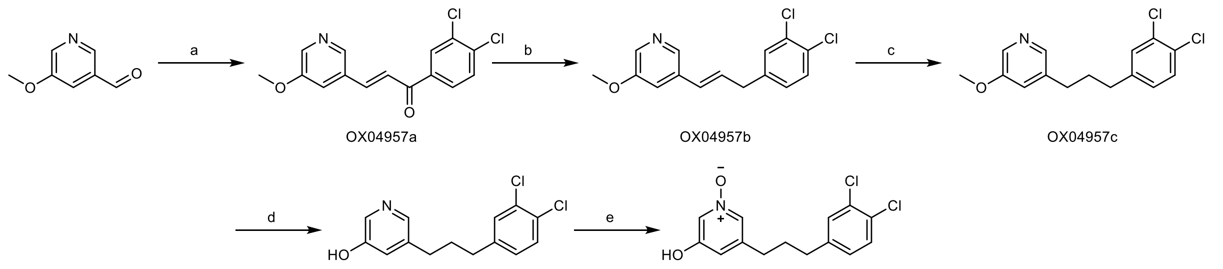

[a]Reagents and conditions: (a) 1-(3,4-dichlorophenyl)ethan-1-one, $BF_3 \cdot Et_2O$, 1,4-dioxane, 105ºC, 16 h, 67%; (b) Et_3SiH, TFA, rt, 48 h, 38%; (c) PtO2, H_2, MeOH, rt, 1 h, 76%; (d) 48% HBr, 120ºC, 48 h, 46%; (e) *m*-CPBA, CH_2Cl_2, rt, 2.5 h, 23%.

Scheme 7. Synthesis of **OX04958**[a]

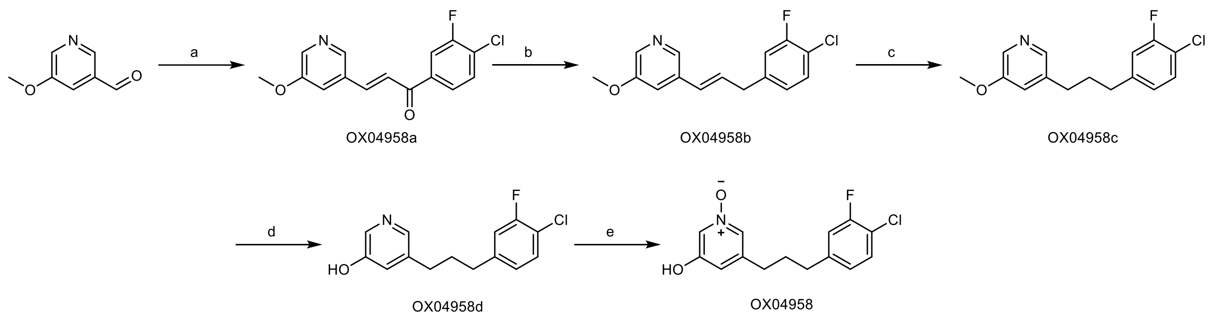

[a]Reagents and conditions: (a) 1-(4-chloro-3-fluorophenyl)ethan-1-one, $BF_3 \cdot Et_2O$, 1,4-dioxane, 105ºC, 16 h, 64%; (b) Et_3SiH, TFA, rt, 48 h, 38%; (c) PtO2, H_2, MeOH, rt, 1 h, 93%; (d) 48% HBr, 120ºC, 48 h, 18%; (e) *m*-CPBA, CH_2Cl_2, rt, 2.5 h, 65%.

Scheme 8. Synthesis of **OX04959**[a]

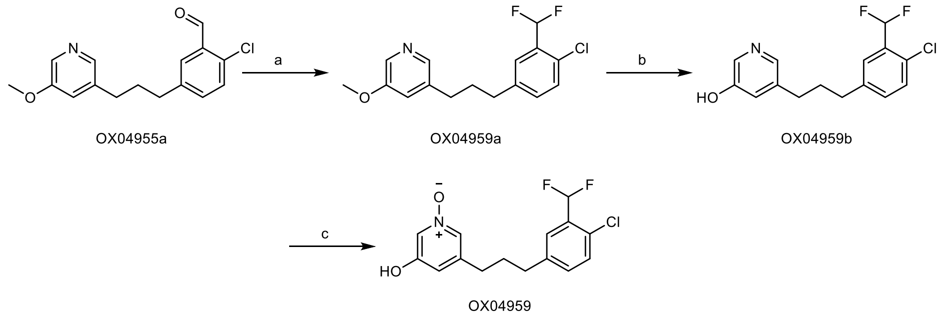

[a]Reagents and conditions: (a) DAST, CH_2Cl_2, rt, 16 h, 37%; (b) L-selectride, THF, 70ºC, 48 h, 92%; (c) *m*-CPBA, CH_2Cl_2, rt, 2 h, 43%.

Scheme 9. Synthesis of **OX04960**[a]

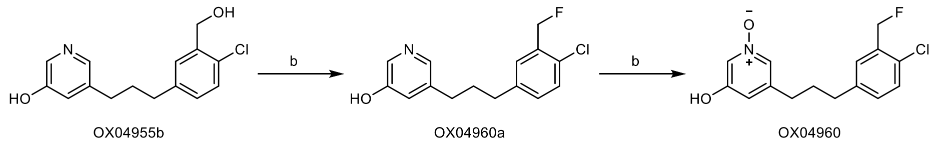

[a]Reagents and conditions: (a) DAST, CH_2Cl_2, rt, 24 h, 11%; (b) *m*-CPBA, CH_2Cl_2, rt, 2 h, 44%.

Scheme 10. Synthesis of **OX04961**[a]

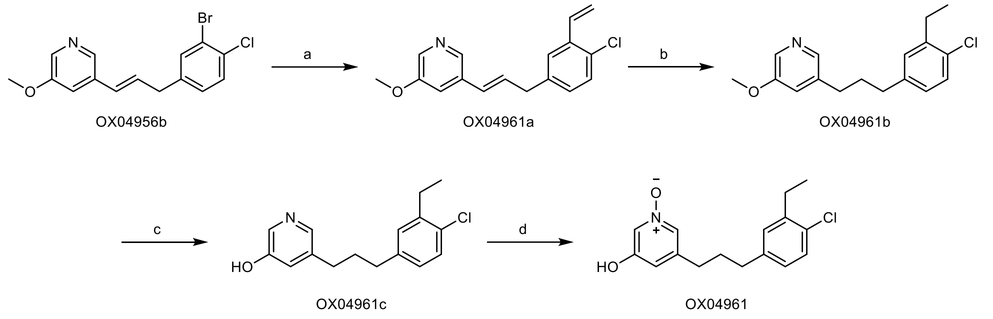

[a]Reagents and conditions: (a) vinyl boronic ester, Cs_2CO_3, PPh_3, $Pd(OAc)_2$, THF:H_2O 9:1, 70ºC, 25h, 56%; (b) Pd/C, H_2, MeOH, rt, 1 h, 39%; (c) 48% HBr, 120ºC, 48 h, 55%; (d) *m*-CPBA, CH_2Cl_2, rt, 2 h, 41%.

Scheme 11. Synthesis of **OX04962**[a]

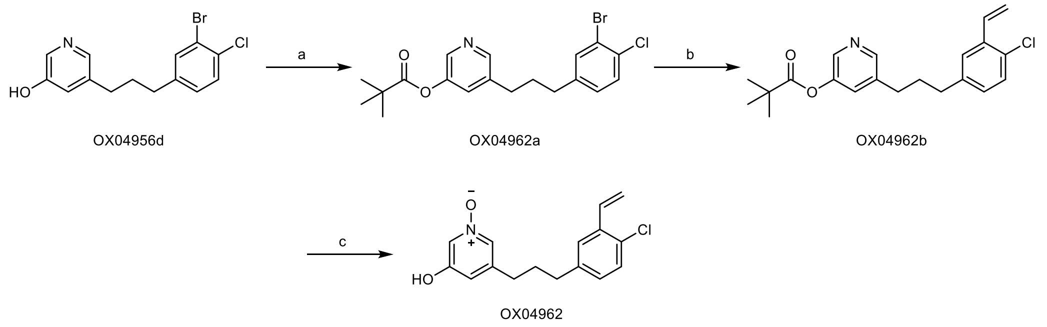

[a]Reagents and conditions: (a) trimethylacetyl chloride, NEt_3, CH_2Cl_2, rt, 1 h, 98%; (b) tributyl(vinyl)stannane, $Pd(PPh_3)_4$, DMF, 90ºC, 24 h, 34%; (c) (1) *m*-CPBA, CH_2Cl_2, rt, 2 h; (2) NaOH, MeOH, rt, 16h, 34%.

Scheme 12. Synthesis of **OX04963**[a]

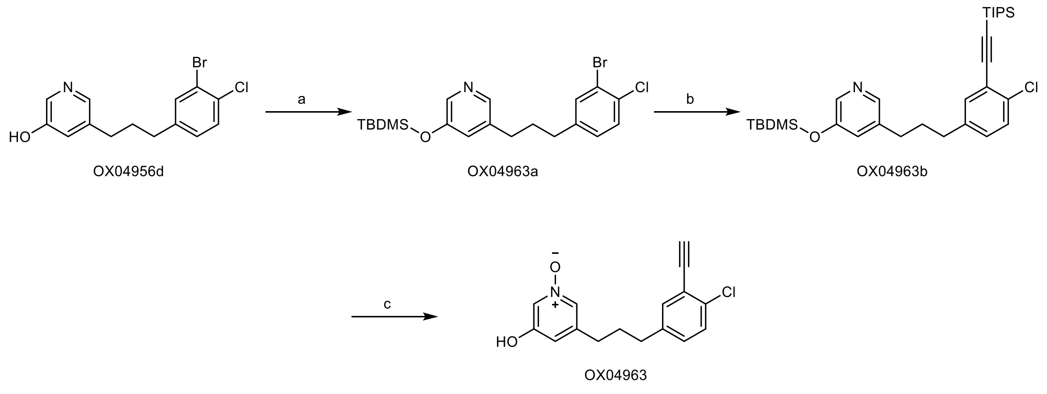

[a]Reagents and conditions: (a) TBDMSCl, imidazole, $CHCl_3$, 60ºC, 16 h, 26%; (b) ethynyltriisopropylsilane, PPh_3, $Pd_2(PPh_3)_2Cl_2$, CuI, THF:NEt_3 3:1, 60ºC, 16 h, 69%; (c) (1) *m*-CPBA, CH_2Cl_2, rt, 2 h; (2) TBAF, THF, rt, 16h, 22%.

Scheme 13. Synthesis of **OX04964**[a]

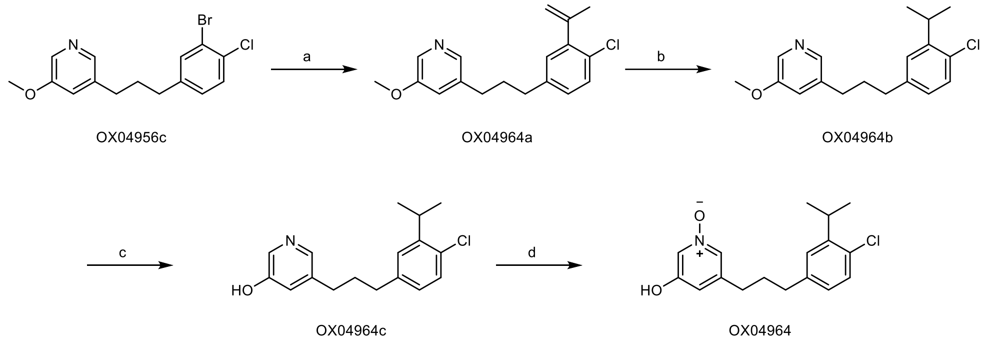

[a]Reagents and conditions: (a) tributyl(prop-1-en-2-yl)stannane, $Pd(PPh_3)_4$, DMF, 90ºC, 24 h, 39%; (b) PtO_2, H_2, MeOH, rt, 1 h, 64%; (c) 48% HBr, 120ºC, 48 h, 60%; (d) *m*-CPBA, CH_2Cl_2, rt, 2 h, 60%.

Scheme 14. Synthesis of **OX04965**[a]

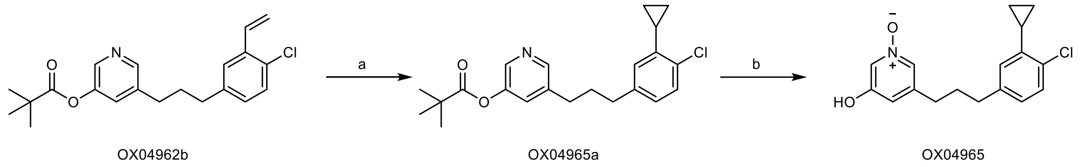

[a]Reagents and conditions: (a) (1) Et_2Zn, TFA, CH2Cl2, 0ºC, 30 min; (2) diiodomethane, CH_2Cl_2, 0ºC–rt, 16 h, 92%; (b) (1) *m*-CPBA, CH_2Cl_2, rt, 2 h; (2) NaOH, MeOH, rt, 16h, 86%.

Scheme 15. Synthesis of **OX04966**[a]

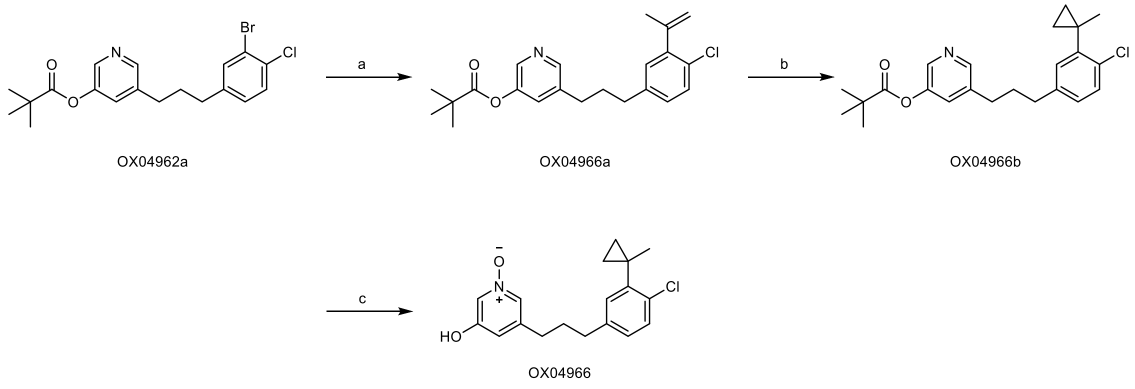

[a]Reagents and conditions: (a) tributyl(vinyl)stannane, $Pd(PPh_3)_4$, DMF, 90ºC, 24 h, 89%; (b) (1) Et_2Zn, TFA, CH_2Cl_2, 0ºC, 30 min; (2) diiodomethane, CH_2Cl_2, 0ºC–rt, 16 h, 31%; (c) (1) *m*-CPBA, CH_2Cl_2, rt, 2 h; (2) NaOH, MeOH, rt, 16h, 99%.

OX04539a

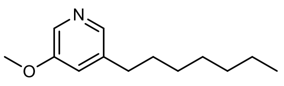

OX04539a

(1) To a solution of 1-bromohexane (825 mg, 5.0 mmol) in 20 mL toluene was added PPh_3 (1443 mg, 5.5 mmol), and the mixture was refluxed for 16h. After completion, the mixture was concentrated under vacuum, redissolved in CH_2Cl_2 and purified by flash column chromatography (0–10% MeOH in CH_2Cl_2) to afford hexyltriphenylphosphonium bromide as a white solid (1351 mg, 63%).

(2) The solid (1042mg, 3.0 mmol) was then dissolved in anhydrous CH_2Cl_2 (30 mL), NaOtBu (577 mg, 6.0 mmol) was added at 0ºC in one portion and the mixture was stirred for 15 minutes before adding 5-methoxynicotinaldehyde (274 mg, 2.0 mmol). The mixture was allowed to ward to rt and stirred for 16 h. After completion, the reaction mixture was diluted with CH_2Cl_2, washed with aq. sat. NH_4Cl solution, brine, dried (Na_2SO_4) and concentrated *in vacuo*. Purification by flash column chromatography (0–30% EtOAc in Pentane) afforded 3-heptyl-5-methoxypyridine as a transparent oil (400 mg, 91%).

(3) The oil (400 mg) was then dissolved in MeOH and the solution was degassed with N_2. 40 mg Pd/C (10 wt.%) was added and the atmosphere was replaced with H_2. The reaction was stirred for 1h, filtered through Celite®, and dried under vacuum to afford **OX04539a** as a transparent oil (400 mg, 99%). ^{1}H NMR (400 MHz, $CDCl_3$) δ 8.12 (d, J = 2.8 Hz, 1H), 8.05 (d, J = 1.8 Hz, 1H), 6.99 (dd, J = 2.8, 1.8 Hz, 1H), 3.83 (s, 3H), 2.57 (dd, J = 8.7, 6.8 Hz, 2H), 1.66–1.54 (m, 2H), 1.37–1.19 (m, 8H), 0.90–0.83 (m, 3H). ^{13}C NMR (101 MHz, $CDCl_3$) δ 155.7, 142.6, 138.9, 134.7, 120.8, 55.6, 32.0, 31.9, 31.2, 29.3, 29.2, 22.7, 14.2. HRMS (ESI +ve) $C_{13}H_{21}NO$ $[M+H]^+$ calc. 208.1696, found 208.1693.

OX04539b

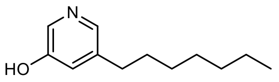

OX04539b

The demethylation using L-Selectride was adapted from a reported route.[1] L-Selectride (6 mL, 1 M in THF) was added to **OX04539a** (400 mg, 1.90 mmol) in THF (10 mL) and then the mixture was stirred at 70ºC for 48 h. After completion, the mixture was cooled to rt, quenched with MeOH, stirred for 30 min and concentrated *in vacuo*. The residue was then purified by flash column chromatography (0–8% MeOH in CH_2Cl_2) to afford **OX04539b** as a yellow oil (146 mg, 39%). ^{1}H NMR (400 MHz, $CDCl_3$) δ 8.73 (d, J = 2.5 Hz, 1H), 8.04 (d, J = 1.6 Hz, 1H), 7.89 (t, J = 2.0 Hz, 1H), 2.72 (t, J = 7.8 Hz, 2H), 1.71–1.58 (m, 2H), 1.50–1.02 (m, 8H), 0.90–0.79 (m, 3H). ^{13}C NMR (101 MHz, $CDCl_3$) δ 157.0, 144.1, 133.4, 130.9, 126.9, 32.9, 31.7, 30.4, 29.0, 29.0, 22.6, 14.1. HRMS (ESI +ve) $C_{12}H_{19}NO$ $[M+H]^+$ calc. 194.1539, found 194.1534.

OX04539

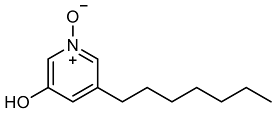

OX04539

To a solution of **OX04539b** (146 mg, 0.76 mmol) in CH_2Cl_2 (20 mL) at rt was added *m*-CPBA (1.5 eq.) and the resulting mixture stirred for 2 h. After completion, the reaction was quenched with $NaHCO_3$ (sat, aq.). The organic phase was washed with brine, dried (Na_2SO_4), filtered and concentrated *in vacuo*. Purification by flash column chromatography (0–6% MeOH in CH_2Cl_2) afforded **OX04539** as a yellow oil (87 mg, 55%). ^{1}H NMR (400 MHz, MeOD) δ 7.67 (q, *J* = 1.7 Hz, 2H), 6.87 (t, *J* = 1.6 Hz, 1H), 2.53–2.45 (m, 2H), 1.58–1.46 (m, 2H), 1.25–1.20 (m, 8H), 0.84–0.76 (m, 3H). ^{13}C NMR (101 MHz, MeOD) δ 157.8, 144.1, 131.8, 126.9, 119.5, 33.5, 32.9, 31.6, 30.1, 30.0, 23.7, 14.4. HRMS (ESI +ve) $C_{12}H_{19}NO_2$ [M+H$^+$] calc. 210.1489, found 210.1487. HPLC 98% (AUC), t_R = 5.1 min.

OX04540a

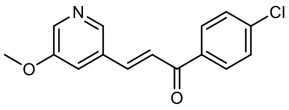

OX04540a

To a solution of 5-methoxynicotinaldehyde (500 mg, 3.65 mmol) and 1-(4-chlorophenyl)ethan-1-one (562 mg, 3.65 mmol) in 1,4-dioxane (10 mL) was added $BF_3·Et_2O$ (1.9 mL) under N_2 and then the mixture was stirred at 105°C for 16 h. After completion of the reaction, cooled to rt, the mixture was diluted with sat. aq. $NaHCO_3$ (50 mL) and extracted with EtOAc (3 × 30 mL). The combined organic extracts were washed with brine (100 mL), dried over Na_2SO_4, filtered, and concentrated under reduced pressure. The resulting residue was chromatographed over silica gel (0 − 60% EtOAc in pentane) to give **OX04540a** as a white solid (700 mg, 70%). ^{1}H NMR (400 MHz, $CDCl_3$) δ 8.46 (d, *J* = 1.7 Hz, 1H), 8.35 (d, *J* = 2.8 Hz, 1H), 8.00–7.93 (m, 2H), 7.77 (d, *J* = 15.8 Hz, 1H), 7.56–7.46 (m, 3H), 7.39 (dd, *J* = 2.8, 1.8 Hz, 1H), 3.92 (s, 3H). ^{13}C NMR (101 MHz, $CDCl_3$) δ 188.7, 155.9, 142.6, 141.6, 139.8, 139.7, 136.2, 131.2, 130.1, 129.2, 123.6, 118.4, 77.5, 77.2, 76.8, 55.9. **HRMS (ESI+ve)** $C_{15}H_{12}ClNO_2$ [M+H$^+$] calc. 274.0629, found 274.0623.

OX04540b

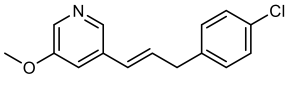

OX04540b

OX04540a (273 mg, 1.0 mmol) was dissolved in TFA (6 mL) using sonication. Triethylsilane (12 mL) was added and the reaction was vigorously stirred for 48h. Upon completion, the reaction mixture was poured into sat. aq. $NaHCO_3$ and stirred until bubbling ceased. This solution was taken up into a separatory funnel and extracted with CH_2Cl_2 (3 × 40 mL), washed with brine, and dried under Na_2SO_4. Organic extracts were dried under vacuum. Purification by flash column chromatography (30% EtOAc in Pentane) afforded

OX04540b as a transparent oil (627 mg, 59%). ^{1}H NMR (400 MHz, $CDCl_3$) δ 8.10 (dd, *J* = 8.6, 2.3 Hz, 2H), 7.25–7.17 (m, 2H), 7.11–7.06 (m, 3H), 6.34–6.29 (m, 2H), 3.77 (s, 3H), 3.48–3.43 (m, 2H). ^{13}C NMR (101 MHz, $CDCl_3$) δ 155.8, 140.8, 138.0, 136.5, 133.5, 132.3, 131.4, 130.1, 129.8, 128.8, 128.6, 127.9, 116.8, 55.6, 38.7.

OX04540c

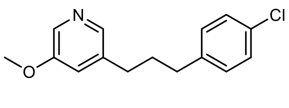

OX04540c

40 mg Pd/C (10% M/w) was added in one portion to a N_2 degassed solution of the **OX04540b** (400 mg, 1.5 mmol) in MeOH (50 mL) at rt, the atmosphere was replaced with H_2, and the reaction stirred for 1 h. After completion, the reaction was filtered through Celite® using MeOH as an eluent, concentrated *in vacuo* and purified by flash column chromatography (30% EtOAc in Pentane) to afford the **OX04540c** as a yellow oil (320 mg, 79%). ^{1}H NMR (400 MHz, $CDCl_3$) δ 8.15 (d, *J* = 2.8 Hz, 1H), 8.06 (d, *J* = 1.8 Hz, 1H), 7.28–7.21 (m, 2H), 7.13–7.05 (m, 2H), 6.98 (dd, *J* = 2.7, 1.8 Hz, 1H), 3.84 (s, 3H), 2.61 (td, *J* = 7.7, 3.0 Hz, 4H), 1.99–1.87 (m, 2H). ^{13}C NMR (101 MHz, $CDCl_3$) δ 155.7, 142.5, 140.2, 138.0, 135.0, 131.8, 129.9, 128.6, 120.8, 55.6, 34.7, 32.5, 32.3. **HRMS (ESI+ve)** $C_{15}H_{16}ClNO$ [M+H$^+$] calc. **262.0993**, found **262.0990**.

OX04540d

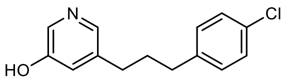

OX04540d

3 mL 48% HBr in H_2O was added to **OX04540c** (100 mg, 0.4 mmol) and then the mixture was stirred at 120ºC for 48 h. After completion of the reaction, cooled to rt, the mixture was neutralized with sat. aq. $NaHCO_3$ and extracted with EtOAc (3 × 30 mL). The combined organic extracts were washed with brine (100 mL), dried over Na_2SO_4, filtered, and concentrated under reduced pressure. The resulting residue was purified by flash column chromatography (5% MeOH in CH_2Cl_2) afforded the **OX04540d** as a transparent oil (22 mg, 23%). ^{1}H NMR (400 MHz, $CDCl_3$) δ 8.27–8.21 (m, 1H), 8.02 (s, 1H), 7.88 (s, 1H), 7.39 (t, *J* = 2.0 Hz, 1H), 7.26–7.22 (m, 2H), 7.11–7.06 (m, 2H), 2.63 (q, *J* = 7.1 Hz, 4H), 1.92 (dq, *J* = 9.3, 7.7 Hz, 2H). ^{13}C NMR (101 MHz, $CDCl_3$) δ 155.2, 140.1, 139.7, 139.6, 134.1, 131.8, 129.9, 128.7, 125.1, 34.7, 32.3, 32.2. HRMS (ESI+ve) $C_{14}H_{14}ClNO$ [M+H$^+$] calc. 248.0837, found 248.0835.

OX04540

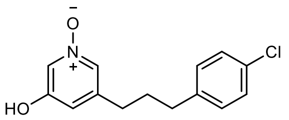

OX04540

To a solution of **OX04540d** (20 mg, 0.08 mmol) in CH_2Cl_2 (10 mL) at rt was added *m*-CPBA (1.5 eq.) and the resulting mixture stirred for 2 h. After completion, the reaction was quenched with $NaHCO_3$ (sat, aq.). The organic phase was washed with brine, dried (Na_2SO_4), filtered and concentrated *in vacuo*. Purification by flash column chromatography (0–100% EtOAc in Pentane then 0–6% MeOH in CH_2Cl_2) afforded **OX04540** as a yellow oil (6 mg, 28%). ^{1}H NMR (600 MHz, $CDCl_3$) δ 8.06 (t, *J* = 1.9 Hz, 1H), 7.63 (t, *J* = 1.5 Hz, 1H), 7.26–7.23 (m, 2H), 7.10–7.05 (m, 2H), 6.98 (t, *J* = 1.7 Hz, 1H), 2.60 (t, *J* = 7.6 Hz, 2H), 2.56–2.50 (m, 2H), 1.89 (tt, *J* = 9.2, 6.8 Hz, 2H). ^{13}C NMR (151 MHz, $CDCl_3$) δ 157.6, 141.7, 139.6, 132.1, 129.8, 129.7, 128.8, 126.6, 120.7, 34.5, 32.2, 31.7. **HRMS (ESI +ve)** $C_{14}H_{14}ClNO_2$ [M+H$^+$] calc. **264.0786**, found 2**64.0784**. HPLC 98% (AUC), t_R = 6.3 min.

OX04954a

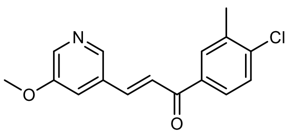

OX04954a

To a solution of 5-methoxynicotinaldehyde (609 mg, 4.45 mmol) and 1-(4-chloro-3-methylphenyl)ethan-1-one (500 mg, 2.96 mmol) in 1,4-dioxane (8 mL) was added $BF_3{\cdot}Et_2O$ (1.5 mL) under N_2 and then the mixture was stirred at 105°C for 16 h. After completion of the reaction, cooled to rt, the mixture was diluted with sat. aq. $NaHCO_3$ (50 mL) and extracted with EtOAc (3 × 30 mL). The combined organic extracts were washed with brine (100 mL), dried over Na_2SO_4, filtered, and concentrated under reduced pressure. The resulting residue was chromatographed over silica gel (0 − 60% EtOAc in pentane) to give **OX04954a** as a white solid (720 mg, 85%). ^{1}H NMR (400 MHz, $CDCl_3$) δ 8.47 (d, J = 1.7 Hz, 1H), 8.35 (d, J = 2.8 Hz, 1H), 7.89 (dd, J = 2.2, 0.9 Hz, 1H), 7.81–7.73 (m, 2H), 7.52 (d, J = 15.7 Hz, 1H), 7.48 (d, J = 8.3 Hz, 1H), 7.43–7.37 (m, 1H), 3.93 (s, 3H), 2.47 (s, 3H). ^{13}C NMR (101 MHz, $CDCl_3$) δ 189.0, 155.9, 142.5, 141.4, 139.9, 139.5, 137.0, 136.3, 131.3, 131.1, 129.6, 127.4, 123.8, 118.6, 55.9, 20.3. **LRMS** (ESI +ve) 288 [M+H]+. **HRMS** (ESI +ve) $C_{16}H_{14}ClNO_2$ [M+H$^+$] calc. 288.0822, found 288.0786.

OX04954b

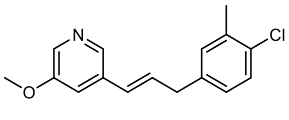

OX04954b

To a solution of **OX04954a** (700 mg, 2.43 mmol) in TFA (3.0 mL) was added Triethylsilane (3.1 mL) under N_2 and then the mixture was stirred at room temperature for 48 h. After completion of the reaction, the mixture was diluted with CH_2Cl_2 (50 mL), quenched with sat. aq. 1N NaOH (30 mL) and extracted with CH_2Cl_2 (3 × 30 mL). The combined organic extracts were washed with brine (100 mL), dried over Na_2SO_4, filtered, and concentrated under reduced pressure. The resulting residue was chromatographed over silica gel (0 − 60% EtOAc in pentane) to give **OX04954b** as a colourless oil (540 mg, 82%). ^{1}H NMR (400 MHz,

$CDCl_3$) δ 8.17 (dd, J = 10.9, 2.3 Hz, 2H), 7.27 (d, J = 8.1 Hz, 1H), 7.15 (dd, J = 2.8, 1.8 Hz, 1H), 7.08 (d, J = 2.2 Hz, 1H), 6.99 (dd, J = 8.1, 2.3 Hz, 1H), 6.41–6.36 (m, 2H), 3.84 (s, 3H), 3.55–3.44 (m, 2H), 2.36 (s, 3H). ^{13}C NMR (101 MHz, $CDCl_3$) δ 155.8, 140.8, 138.1, 136.5, 136.2, 133.6, 132.4, 131.6, 131.4, 129.2, 127.7, 127.5, 116.8, 55.6, 38.8, 20.1. LRMS (ESI +ve) 274 [M+H]+. HRMS (ESI +ve) $C_{16}H_{16}ClNO$ [M+H$^+$] calc. 274.0993, found 274.1040.

OX04954c

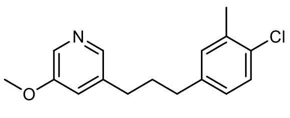

OX04954c

A mixture of **OX04954b** (500 mg, 1.82 mmol) and 50 mg PtO_2 (10% w/w) in MeOH (10 mL) was stirred at rt under an atmosphere of H_2 gas via balloon (1 atm of pressure) for 30 min. The mixture was filtered through a pad of Celite® with repeat rinsing using anhydrous MeOH. The filtrate was concentrated under reduced pressure, and the resulting crude residue was chromatographed over silica gel (0 − 40% EtOAc in pentane) to give **OX04954c** as a white solid (430 mg, 85%). ^1H NMR (400 MHz, $CDCl_3$) δ 8.14 (d, J = 2.8 Hz, 1H), 8.05 (d, J = 1.8 Hz, 1H), 7.22 (d, J = 8.1 Hz, 1H), 7.03–6.96 (m, 2H), 6.92 (dd, J = 8.2, 2.2 Hz, 1H), 3.83 (s, 3H), 2.59 (dt, J = 10.4, 7.6 Hz, 4H), 2.33 (s, 3H), 2.01–1.85 (m, 2H). ^{13}C NMR (101 MHz, $CDCl_3$) δ 155.7, 142.4, 140.3, 138.0, 135.9, 134.9, 131.8, 131.1, 129.0, 127.2, 120.7, 55.5, 34.6, 32.5, 32.3, 20.1. **LRMS** (ESI +ve) 276 [M+H]+. **HRMS** (ESI +ve) $C_{16}H_{18}ClNO$ [M+H$^+$] calc. 276.1150, found 276.1181.

OX04954d

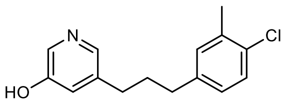

OX04954d

To 3-(3-(4-chloro-3-methylphenyl)propyl)-5-methoxypyridine **OX04954c (**250 mg, 0.906 mmol) in a vial was added 48% HBr in water (5 mL) and then the vial was sealed and the mixture was stirred at 120ºC for 48 h. After completion of the reaction, cooled to rt, the mixture was neutralized with sat. aq. $NaHCO_3$ and extracted with EtOAc (3 × 30 mL). The combined organic extracts were washed with brine (100 mL), dried over Na_2SO_4, filtered, and concentrated under reduced pressure. The resulting residue was chromatographed over silica gel (0 − 10% MeOH in CH_2Cl_2) to give **OX04954d** as a white solid (187 mg, 79%). ^1H NMR (400 MHz, Acetone) δ 8.84 (s, 1H), 8.00 (dd, J = 34.7, 2.3 Hz, 2H), 7.31–7.13 (m, 2H), 7.11–7.00 (m, 2H), 2.61 (td, J = 7.8, 3.8 Hz, 4H), 2.32 (s, 3H), 1.97–1.88 (m, 2H). ^{13}C NMR (101 MHz, Acetone) δ 154.4, 142.0, 142.0, 139.1, 136.6, 136.3, 132.1, 132.0, 129.6, 128.3, 122.7, 35.2, 33.5, 32.7, 20.0.. **LRMS** (ESI +ve) 262 [M+H]+. **HRMS** (ESI +ve) $C_{15}H_{16}ClNO$ [M+H$^+$] calc. 262.0993, found 262.1028

OX04954

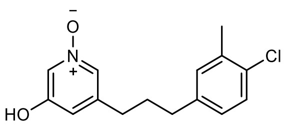

OX04954

To a solution of **OX04954a** (150 mg, 0.573 mmol) in CH_2Cl_2 (10 mL) at rt was added *m*-CPBA (1.5 eq.) and the resulting mixture stirred for 2 h. After completion, the reaction was quenched with $NaHCO_3$ (sat, aq.). The organic phase was washed with brine, dried (Na_2SO_4), filtered and concentrated *in vacuo*. Purification by flash column chromatography (0–6% MeOH in CH_2Cl_2) afforded **OX04954** as a white solid (138 mg, 87%). ^{1}H NMR (400 MHz, $CDCl_3$) δ 7.99 (t, J = 1.9 Hz, 1H), 7.62 (t, J = 1.5 Hz, 1H), 7.22 (d, J = 8.1 Hz, 1H), 7.00 (d, J = 2.2 Hz, 1H), 6.94–6.85 (m, 2H), 2.53 (dt, J = 16.2, 7.7 Hz, 4H), 2.37 (s, 3H), 1.95–1.81 (m, 2H). ^{13}C NMR (101 MHz, $CDCl_3$) δ 157.5, 141.6, 139.7, 136.1, 132.1, 131.1, 129.8, 129.1, 127.2, 126.6, 119.8, 34.4, 32.2, 31.8, 20.1. **LRMS** (ESI +ve) 278 [M+H]+. **HRMS** (ESI +ve) $C_{15}H_{16}ClNO_2$ [M+H$^+$] calc. 278.0942, found 278.0992. HPLC 97% (AUC), t_R = 7.4 min.

OX04956a

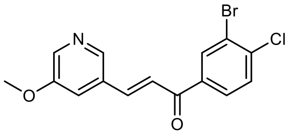

OX04956a

To a solution of 5-methoxynicotinaldehyde (1181 mg, 8.62 mmol) and 1-(3-bromo-4-chlorophenyl)ethan-1-one (1000 mg, 4.31 mmol) in 1,4-dioxane (10 mL) was added $BF_3{\cdot}Et_2O$ (2.2 mL) under N_2 and then the mixture was stirred at 105°C for 16 h. After completion of the reaction, cooled to rt, the mixture was diluted with sat. aq. $NaHCO_3$ (50 mL) and extracted with EtOAc (3 × 30 mL). The combined organic extracts were washed with brine (100 mL), dried over Na_2SO_4, filtered, and concentrated under reduced pressure. The resulting residue was chromatographed over silica gel (0 − 60% EtOAc in pentane) to give **OX04956a** as a white solid (1168 mg, 29%). ^{1}H NMR (400 MHz, DMSO) δ 8.79 (d, *J* = 1.7 Hz, 1H), 8.52 (dd, *J* = 5.8, 2.4 Hz, 2H), 8.27 (dd, *J* = 2.7, 1.7 Hz, 1H), 8.22–8.14 (m, 2H), 7.90–7.80 (m, 2H), 3.97 (s, 3H). ^{13}C NMR (101 MHz, DMSO) δ 186.7, 156.4, 140.2, 140.2, 138.5, 137.2, 136.4, 133.7, 132.4, 131.1, 129.3, 124.8, 122.4, 122.3, 56.5, 39.9, 39.7, 39.5, 39.3, 39.1.

OX04956b

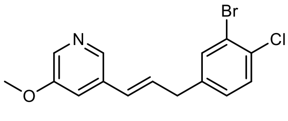

OX04956b

OX04956a (1100 mg, 3.1 mmol) was dissolved in TFA (10 mL) using sonication. Triethylsilane (20 mL) was added and the reaction was vigorously stirred for 48h. Upon completion, the reaction mixture was poured into sat. aq. $NaHCO_3$ and stirred until bubbling ceased. This solution was taken up into a separatory

funnel and extracted with CH_2Cl_2 (3 × 70 mL), washed with brine, and dried under Na_2SO_4. Organic extracts were dried under vacuum. Purification by flash column chromatography (30% EtOAc in Pentane) afforded **OX04956b** as a transparent oil (627 mg, 59%). ^{1}H NMR (400 MHz, $CDCl_3$) δ 8.17 (dd, *J* = 6.7, 2.3 Hz, 2H), 7.48 (d, *J* = 2.0 Hz, 1H), 7.37 (d, *J* = 8.2 Hz, 1H), 7.15 (t, *J* = 2.3 Hz, 1H), 7.10 (dd, *J* = 8.2, 2.1 Hz, 1H), 6.43–6.29 (m, 2H), 3.84 (s, 3H), 3.50 (d, *J* = 6.1 Hz, 2H). ^{13}C NMR (101 MHz, $CDCl_3$) δ 155.8, 140.8, 139.9, 136.6, 133.9, 133.3, 132.5, 130.5, 130.4, 128.9, 128.4, 122.6, 116.9, 55.6, 38.4. **HRMS (ESI+ve)** $C_{15}H_{13}BrClNO$ [M+H$^+$] calc. **337.9942**, found **337.9936**.

OX04956c

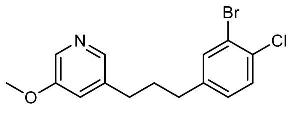

OX04956c

30 mg PtO2 was added in one portion to a N_2 degassed solution of the **OX04956b** (600 mg, 1.8 mmol) in MeOH (80 mL) at rt, the atmosphere was replaced with H_2, and the reaction stirred for 1 h. After completion, the reaction was filtered through Celite® using MeOH as an eluent, concentrated *in vacuo* and purified by flash column chromatography (30% EtOAc in Pentane) to afford **OX04956c** as a transparent oil (514 mg, 85%). ^{1}H NMR (400 MHz, $CDCl_3$) δ 8.12 (d, *J* = 2.8 Hz, 1H), 8.02 (d, *J* = 1.8 Hz, 1H), 7.40 (d, *J* = 2.1 Hz, 1H), 7.31 (d, *J* = 8.2 Hz, 1H), 7.02 (dd, *J* = 8.2, 2.1 Hz, 1H), 6.96 (dd, *J* = 2.7, 1.8 Hz, 1H), 3.82 (s, 3H), 2.58 (q, *J* = 7.3 Hz, 4H), 1.95–1.85 (m, 2H). ^{13}C NMR (101 MHz, $CDCl_3$) δ 155.7, 142.2, 142.1, 137.7, 134.0, 133.6, 131.8, 130.2, 128.6, 122.3, 120.7, 55.5, 34.3, 32.2. **HRMS (ESI+ve)** $C_{15}H_{15}BrClNO$ [M+H$^+$] calc. **340.0098**, found **340.0094**.

OX04956d

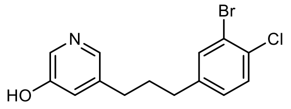

OX04956d

6 mL 48% HBr in H_2O was added to **OX04956c** (440 mg, 1.3 mmol) and then the mixture was stirred at 120ºC for 48 h. After completion of the reaction, cooled to rt, the mixture was neutralised with sat. aq. $NaHCO_3$ and extracted with EtOAc (3 × 40 mL). The combined organic extracts were washed with brine (100 mL), dried over Na_2SO_4, filtered, and concentrated under reduced pressure. The resulting residue was purified by flash column chromatography (5% MeOH in CH_2Cl_2) afforded **OX04956d** as a transparent oil (120 mg, 28%). ^{1}H NMR (400 MHz, MeOD) δ 7.92 (d, *J* = 2.7 Hz, 1H), 7.84 (d, *J* = 1.8 Hz, 1H), 7.48 (d, *J* = 2.0 Hz, 1H), 7.37 (d, *J* = 8.2 Hz, 1H), 7.13 (dd, *J* = 8.2, 2.1 Hz, 1H), 7.07 (dd, *J* = 2.6, 1.8 Hz, 1H), 2.59 (td, *J* = 7.7, 5.1 Hz, 4H), 1.96–1.82 (m, 2H). ^{13}C NMR (101 MHz, MeOD) δ 155.7, 144.1, 140.9, 140.3, 135.9, 134.7, 132.6, 131.3, 130.1, 124.4, 122.9, 35.2, 33.4, 32.9. **HRMS (ESI+ve)** $C_{14}H_{13}BrClNO$ [M+H$^+$] calc. **325.9942**, found **325.9936**.

OX04956

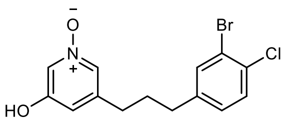

OX04956

To a solution of **OX04956d** (20 mg, 0.035 mmol) in CH_2Cl_2 (10 mL) at rt was added *m*-CPBA (1.5 eq.) and the resulting mixture stirred for 2 h. After completion, the reaction was quenched with $NaHCO_3$ (sat., aq.). The organic phase was washed with brine, dried (Na_2SO_4), filtered and concentrated *in vacuo*. Purification by flash column chromatography (0–100% EtOAc in Pentane then 0–6% MeOH in CH_2Cl_2) afforded **OX04956** as a yellow oil (11 mg, 52%). ^{1}H NMR (400 MHz, MeOD) δ 7.77 (d, *J* = 1.8 Hz, 2H), 7.53 (d, *J* = 2.1 Hz, 1H), 7.40 (d, *J* = 8.2 Hz, 1H), 7.17 (dd, *J* = 8.2, 2.1 Hz, 1H), 6.96 (t, *J* = 1.8 Hz, 1H), 2.62 (dt, *J* = 13.4, 7.7 Hz, 4H), 1.97–1.86 (m, 2H). ^{13}C NMR (101 MHz, MeOD) δ 157.8, 143.9, 143.4, 134.8, 132.8, 131.8, 131.3, 130.1, 127.1, 123.0, 119.4, 49.6, 49.4, 49.2, 49.0, 48.8, 48.6, 48.4, 35.1, 33.0, 32.8. **HRMS (ESI +ve)** $C_{14}H_{13}BrClNO_2$ [M+H$^+$] calc. **339.9745**, found **339.9745**. HPLC 98% (AUC), t_R = 7.5 min.

OX04955a

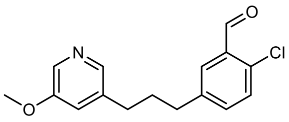

OX04955a

To a solution of **OX4956c** (555 mg, 1.6 mmol) in THF (4 mL) was added *n*-BuLi (1.4 mL, 2.5 M in hexane) dropwise over 10 min at -78ºC. The reaction was stirred for 5 min before adding DMF:THF mixture (1:9, 4 mL) in one portion at -78ºC. The reaction was stirred for 15 min then quenched with brine (5 mL) at -78ºC, and the mixture was allowed to warm to room temperature. The organic layer was washed with brine (8 mL × 2), dried (Na_2SO_4), concentrated *in vacuo* and purified with flash column chromatography (10–30% EtOAc in petroleum ether) to afford the **OX04955a** as a transparent oil (212 mg, 45%). ^{1}H NMR (400 MHz, $CDCl_3$) δ 10.46 (s, 1H), 8.15 (d, *J* = 2.8 Hz, 1H), 8.05 (s, 1H), 7.73 (dd, *J* = 2.1, 0.7 Hz, 1H), 7.38–7.31 (m, 2H), 6.98 (dd, *J* = 2.7, 1.8 Hz, 1H), 3.84 (s, 3H), 2.65 (dt, *J* = 20.7, 7.7 Hz, 4H), 2.01–1.90 (m, 2H). ^{13}C NMR (101 MHz, $CDCl_3$) δ 190.1, 155.8, 142.4, 141.4, 137.7, 135.7, 135.5, 135.2, 132.4, 130.7, 129.0, 120.7, 55.6, 34.6, 32.3, 32.3. LRMS (ESI +ve) 290 [M+H]$^+$. HRMS (ESI +ve) $C_{16}H_{16}ClNO_2$ [M+H]$^+$ calc. 290.0942, found 290.0941.

OX04955b

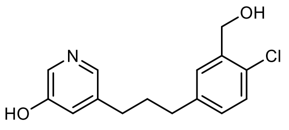

OX04955b

The demethylation using L-Selectride was adapted from a reported route.[1] L-Selectride (1.2 mL, 1 M in THF) was added to **OX04955a** (120 mg, 0.4 mmol) in THF (4 mL) and then the mixture was stirred at 70ºC for 48 h. After completion, the mixture was cooled to rt, quenched with MeOH, stirred for 30 min and concentrated *in vacuo*. The residue was then purified by flash column chromatography (0–8% MeOH in CH_2Cl_2) to afford **OX04955b** as a transparent oil (45 mg, 39%). ^{1}H NMR (400 MHz, MeOD) δ 7.91 (d, *J* = 2.6 Hz, 1H), 7.84 (d, *J* = 1.8 Hz, 1H), 7.37 (d, *J* = 2.2 Hz, 1H), 7.25 (d, *J* = 8.1 Hz, 1H), 7.11–7.03 (m, 2H), 4.67 (s, 2H), 2.62 (dt, *J* = 17.9, 7.8 Hz, 4H), 1.99–1.85 (m, 2H). ^{13}C NMR (101 MHz, MeOD) δ 155.7, 142.2, 140.9, 140.6, 139.8, 135.8, 130.8, 130.0, 129.6, 129.5, 124.3, 62.3, 35.7, 33.7, 33.0. LRMS (ESI +ve) 278 $[M+H]^+$. HRMS (ESI +ve) $C_{15}H_{16}ClNO_2$ $[M+H]^+$ calc. 278.0942, found 278.0932.

OX04955

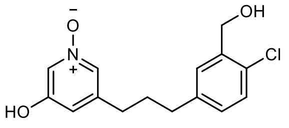

OX04955

To a solution of **OX04955b** (25 mg, 0.1 mmol) in CH_2Cl_2 (15 mL) at rt was added *m*-CPBA (1.5 eq.) and the resulting mixture stirred for 2 h. After completion, the reaction was quenched with $NaHCO_3$ (sat., aq.). The organic phase was washed with brine, dried (Na_2SO_4), filtered and concentrated *in vacuo*. Purification by flash column chromatography (0–10% MeOH in CH_2Cl_2) afforded **OX04955** as a yellow solid (17 mg, 64%). ^{1}H NMR (400 MHz, MeOD) δ 7.76 (p, *J* = 1.7 Hz, 2H), 7.38 (d, *J* = 2.2 Hz, 1H), 7.26 (d, *J* = 8.1 Hz, 1H), 7.10 (dd, *J* = 8.1, 2.3 Hz, 1H), 6.95 (dd, *J* = 2.1, 1.4 Hz, 1H), 4.67 (s, 2H), 2.68 (t, *J* = 7.6 Hz, 2H), 2.65–2.59 (m, 2H), 2.01–1.91 (m, 2H). ^{13}C NMR (151 MHz, MeOD) δ 157.8, 143.6, 141.9, 139.9, 131.9, 130.9, 130.1, 129.6, 129.5, 127.0, 119.4, 62.3, 35.5, 33.0, 33.0. LRMS (ESI +ve) 294 $[M+H]^+$. HRMS (ESI +ve) $C_{15}H_{16}ClNO_3$ $[M+H]^+$ calc. 294.0892, found 294.0890. HPLC 96% (AUC), t_R = 3.2 min.

OX04957a

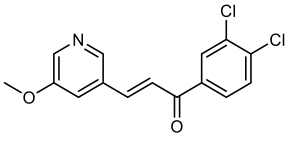

OX04957a

To a solution of 5-methoxynicotinaldehyde (1.00 g, 7.3 mmol) in 10 mL 1,4-dioxane was added 1-(3,4-dichlorophenyl)ethan-1-one (1.15 g, 6.1 mmol). $BF_3·OEt_2$ (6.0 mL) was added and the mixture was heated at 105ºC for 16h. After completion, the reaction was cooled to room temperature and slowly poured into aq. sat. $NaHCO_3$ and stirred until bubbling ceased. This solution was taken up into a separatory funnel and

extracted with CH_2Cl_2. The organic layer was dried over Na_2SO_4 then under vacuum. To the crude residue, 5 mL MeOH was added. This mixture was diluted with pentane and allowed to stir for 20 minutes. The resulting suspension was filtered and washed with pentane to afford **OX04957a** as a yellow-brown solid (1.52 g, 67%). ^1H NMR (400 MHz, $CDCl_3$) δ 8.47 (d, *J* = 1.8 Hz, 1H), 8.36 (d, *J* = 2.8 Hz, 1H), 8.10 (d, *J* = 2.0 Hz, 1H), 7.85 (dd, *J* = 8.3, 2.1 Hz, 1H), 7.80 (d, *J* = 15.7 Hz, 1H), 7.60 (d, *J* = 8.4 Hz, 1H), 7.48 (d, *J* = 15.7 Hz, 1H), 7.41 (dd, *J* = 2.7, 2.0 Hz, 1H), 3.93 (s, 3H). ^{13}C NMR (101 MHz, $CDCl_3$) δ 187.6, 156.0, 142.7, 142.3, 139.9, 137.9, 137.4, 133.6, 131.0, 131.0, 130.7, 127.7, 123.1, 118.5, 55.9. HRMS (ESI +ve) $C_{15}H_{11}Cl_2NO_2$ $[M+H]^+$ calc. 308.0240, found 308.0255.

OX04957b

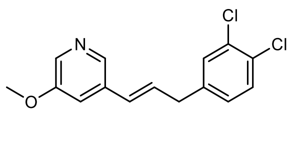

OX04957b

OX04957a (1.52 g, 4.9 mmol) was dissolved in TFA (10 mL) using sonication. Triethylsilane (20 mL) was added and the reaction was vigorously stirred for 48h. Upon completion, the reaction mixture was poured into sat. aq. $NaHCO_3$ and stirred until bubbling ceased. This solution was taken up into a separatory funnel and extracted with CH_2Cl_2, washed with brine, and dried under Na_2SO_4. Organic extracts were dried under vacuum. Purification by flash column chromatography (0–50% EtOAc in pentane) afforded **OX04957b** as a pale yellow solid (547 mg, 38%). ^1H NMR (400 MHz, $CDCl_3$) δ 8.19–8.16 (m, 2H), 7.37 (d, *J* = 8.2 Hz, 1H), 7.31 (d, *J* = 2.1 Hz, 1H), 7.15 (dd, *J* = 2.8, 1.8 Hz, 1H), 7.08–7.03 (m, 1H), 6.41 (d, *J* = 15.9 Hz, 1H), 6.34 (dt, *J* = 15.8, 6.1 Hz, 1H), 3.85 (s, 3H), 3.51 (d, *J* = 6.0 Hz, 2H). ^{13}C NMR (101 MHz, $CDCl_3$) δ 155.5, 140.4, 139.5, 136.3, 133.0, 132.3, 130.4, 130.2, 130.2, 130.2, 128.1, 127.9, 116.6, 55.2, 38.1. HRMS (ESI +ve) $C_{15}H_{13}Cl_2NO$ $[M+H]^+$ calc. 294.0447, found 294.0449.

OX04957c

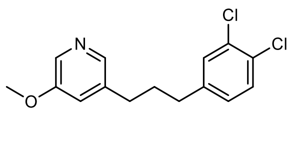

OX04957c

OX04957b (547 mg, 1.83 mmol) was dissolved in MeOH and the solution was degassed with N_2. PtO_2 (27 mg, 5 mol%) was added and the atmosphere was replaced with H_2. The reaction was stirred for 1h, filtered through Celite®, and dried under vacuum. Purification by flash column chromatography (0–40% EtOAc in pentane) afforded **OX04957c** as a pale yellow oil (411 mg, 76%). ^1H NMR (400 MHz, $CDCl_3$) δ 8.16 (d, *J* = 2.8 Hz, 1H), 8.06 (d, *J* = 1.8 Hz, 1H), 7.34 (d, *J* = 8.2 Hz, 1H), 7.26 (s, 1H), 7.00 (dd, *J* = 8.1, 2.1 Hz, 2H), 3.85 (s, 3H), 2.62 (d, *J* = 4.9 Hz, 4H), 1.99–1.89 (m, 2H). ^{13}C NMR (101 MHz, $CDCl_3$) δ 155.8, 142.3, 142.1, 137.8, 135.0, 132.4, 130.5, 130.5, 130.1, 128.0, 120.9, 55.7, 34.5, 32.3, 32.3. HRMS (ESI +ve) $C_{15}H_{15}Cl_2NO$ $[M+H^+]$ calc. 296.0604, found 296.0609.

OX04957d

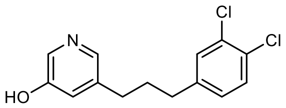

OX04957d

6 mL 48% HBr in H_2O was added to **OX04957c** (411 mg, 1.39 mmol), and the reaction was heated to 120℃ and stirred for 48 h. Upon completion, the reaction mixture was poured into aq. sat. $NaHCO_3$ and stirred until bubbling ceased. This solution was taken up into a separatory funnel and extracted with EtOAc, washed with brine, and dried under Na_2SO_4. Organic extracts were dried under vacuum. Purification by flash column chromatography (0–100% EtOAc in pentane) afforded **OX04957d** as a pale orange solid (180 mg, 46%). ^1H NMR (400 MHz, $CDCl_3$) δ 8.12 (d, *J* = 2.6 Hz, 1H), 7.91 (d, *J* = 1.8 Hz, 1H), 7.33 (d, *J* = 8.2 Hz, 1H), 7.25 (d, *J* = 2.1 Hz, 1H), 7.14 (t, *J* = 2.1 Hz, 1H), 6.99 (dd, *J* = 8.2, 2.1 Hz, 1H), 2.61 (td, *J* = 8.3, 3.4 Hz, 4H), 1.98–1.86 (m, 2H). ^{13}C NMR (101 MHz, $CDCl_3$) δ 155.4, 141.9, 139.6, 139.2, 133.9, 132.4, 130.5, 130.4, 130.1, 128.0, 125.3, 34.5, 32.2, 32.1. HRMS (ESI +ve) $C_{14}H_{13}Cl_2NO$ [M+H$^+$] calc. 282.0447, found 282.0459.

OX04957

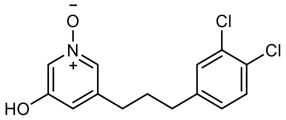

OX04957

To a solution of **OX04957d** (50 mg, 0.18 mmol) in CH_2Cl_2 (10 mL) was added 77% *meta*-chloroperoxybenzoic acid (60 mg, 0.27 mmol) and the reaction was stirred at room temperature for 2 h. Another portion of 77% *meta*-chloroperoxybenzoic acid (40 mg, 0.18 mmol) was added and the reaction was further stirred for 30 mins. Upon completion, aq. 2M NaOH (5 mL) was added to the reaction mixture. The pH was adjusted to 3 – 4 using aq. 1M HCl, and a precipitate formed in the organic phase. The precipitate was filtered and washed with CH_2Cl_2 to afford **OX04957** as a white solid (12 mg, 23%). ^1H NMR (400 MHz, DMSO) δ 10.38 (s, 1H), 7.68 (t, *J* = 1.5 Hz, 1H), 7.61 (t, *J* = 1.9 Hz, 1H), 7.53 (d, *J* = 8.3 Hz, 1H), 7.51 (d, *J* = 2.1 Hz, 1H), 7.22 (dd, *J* = 8.2, 2.1 Hz, 1H), 6.67 (t, *J* = 1.7 Hz, 1H), 2.63–2.56 (m, 2H), 2.47 (t, *J* = 6.9 Hz, 2H), 1.84 (tt, *J* = 9.5, 6.6 Hz, 2H). ^{13}C NMR (101 MHz, $CDCl_3$) δ 157.6, 141.4, 141.3, 132.5, 130.6, 130.4, 130.3, 129.8, 128.0, 126.7, 119.8, 34.3, 32.2, 31.5. HRMS (ESI +ve) $C_{14}H_{13}Cl_2NO_2$ [M+H$^+$] calc. 298.0396, found 298.0408. HPLC 98% (AUC), t_R = 7.3 min.

OX04958a

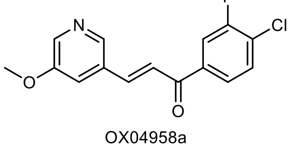

OX04958a

To a solution of 5-methoxynicotinaldehyde (1.00 g, 7.3 mmol) in 10 mL 1,4-dioxane was added 1-(4-chloro-3-fluorophenyl)ethan-1-one (1.05 g, 6.1 mmol). $BF_3{\cdot}OEt_2$ (6.0 mL) was added and the mixture was heated to 105°C for 16h. After completion, the reaction was cooled to room temperature and slowly poured into aq. sat. $NaHCO_3$ and stirred until bubbling ceased. This solution was taken up into a separatory funnel and extracted with CH_2Cl_2. Organic extracts were dried under vacuum. To the crude residue, a small amount of MeOH was added. This mixture was diluted with pentane, and allowed to stir for 20 minutes. The resulting suspension was filtered and washed with pentane to afford **OX04958a** as a yellow-brown solid (1.13 g, 64%). ^{1}H NMR (400 MHz, $CDCl_3$) δ 8.47 (d, *J* = 1.8 Hz, 1H), 8.36 (d, *J* = 2.8 Hz, 1H), 7.82–7.73 (m, 3H), 7.55 (dd, *J* = 8.2, 7.1 Hz, 1H), 7.48 (d, *J* = 15.7 Hz, 1H), 7.42–7.38 (m, 1H), 3.93 (s, 3H). ^{13}C NMR (101 MHz, $CDCl_3$) δ 187.5, 158.4 (d, *J* = 251.3 Hz), 156.0, 142.6, 142.3, 139.9, 138.1 (d, *J* = 5.4 Hz), 131.2, 131.0, 126.7 (d, *J* = 17.9 Hz), 125.0 (d, *J* = 3.7 Hz), 118.5, 116.7 (d, *J* = 22.1 Hz), 116.6, 55.9. ^{19}F NMR (376 MHz, $CDCl_3$) δ -113.42 (dd, *J* = 9.4, 7.1 Hz). HRMS (ESI +ve) $C_{15}H_{11}ClFNO_2$ $[M+H]^+$ calc. 292.0535, found 292.0532.

OX04958b

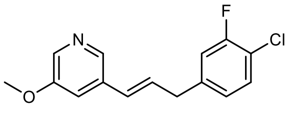

OX04958b

OX04958a (1.13 g, 3.9 mmol) was dissolved in TFA (8 mL) using sonication. Triethylsilane (16 mL) was added and the reaction was vigorously stirred for 48h. Upon completion, the reaction mixture was poured into aq. sat. $NaHCO_3$ and stirred until bubbling ceased. This solution was taken up into a separatory funnel and extracted with CH_2Cl_2, washed with brine, and dried under Na_2SO_4. Organic extracts were dried under vacuum. Purification by flash column chromatography (0–40% EtOAc in pentane) afforded **OX04958b** as a pale yellow solid (406 mg, 38%). ^{1}H NMR (400 MHz, $CDCl_3$) δ 8.19 (d, *J* = 1.8 Hz, 1H), 8.17 (d, *J* = 2.8 Hz, 1H), 7.33 (t, *J* = 7.9 Hz, 1H), 7.16 (dd, *J* = 2.8, 1.8 Hz, 1H), 7.02 (dd, *J* = 9.9, 2.0 Hz, 1H), 6.96 (ddd, *J* = 8.2, 2.0, 0.8 Hz, 1H), 6.42 (d, *J* = 16.0 Hz, 1H), 6.35 (dt, *J* = 15.8, 6.0 Hz, 1H), 3.86 (s, 3H), 3.54 (d, *J* = 5.9 Hz, 2H). ^{13}C NMR (101 MHz, $CDCl_3$) δ 158.1 (d, *J* = 248.8 Hz), 155.8, 140.6, 140.4 (d, *J* = 6.3 Hz), 136.5, 133.3, 130.6, 130.5, 128.3, 125.1 (d, *J* = 3.5 Hz), 118.9 (d, *J* = 8.1 Hz), 116.9, 116.9 (d, *J* = 21.2 Hz), 55.6, 38.6 (d, *J* = 1.6 Hz). ^{19}F NMR (376 MHz, $CDCl_3$) δ -115.44 (dd, *J* = 10.0, 7.6 Hz). HRMS (ESI +ve) $C_{15}H_{13}ClFNO$ $[M+H]^+$ calc. 278.0743, found 278.0736

OX04958c

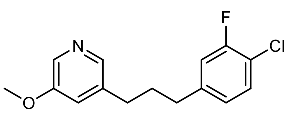

OX04958c

OX04958b (406 mg, 1.46 mmol) was dissolved in MeOH and the solution was degassed with N_2. PtO_2 (20 mg, 5 mol%) was added and the atmosphere was replaced with H_2. The reaction was stirred for 1h, filtered through Celite®, and dried under vacuum to afford **OX04958c** as a pale yellow oil (380 mg, 93%). ^{1}H NMR (400 MHz, $CDCl_3$) δ 8.16 (d, *J* = 2.8 Hz, 1H), 8.06 (d, *J* = 1.7 Hz, 1H), 7.29 (t, *J* = 7.9 Hz, 1H), 7.00–6.98 (m, 1H), 6.96 (dd, *J* = 10.1, 2.0 Hz, 1H), 6.89 (ddd, *J* = 8.2, 2.1, 0.8 Hz, 1H), 3.85 (s, 3H), 2.63 (dd, *J* = 7.4, 1.7 Hz, 4H), 1.99–1.88 (m, 2H). ^{13}C NMR (151 MHz, $CDCl_3$) δ 158.2 (d, *J* = 248.5 Hz), 155.8, 142.8 (d, *J* = 6.5 Hz), 142.4, 137.8, 135.1, 130.5, 125.0 (d, *J* = 3.4 Hz), 120.9, 118.4 (d, *J* = 17.7 Hz), 116.6 (d, *J* = 20.4 Hz), 55.7, 34.7, 32.3, 32.3. ^{19}F NMR (376 MHz, $CDCl_3$) δ -115.94 (dd, *J* = 10.1, 7.6 Hz). HRMS (ESI +ve) $C_{15}H_{15}ClFNO$ [M+H$^+$] calc. 280.0899, found 280.0913

OX04958d

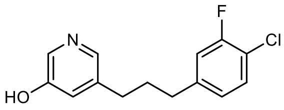

OX04958d

5 mL 48% HBr in H_2O was added to **OX04958c** (380 mg, 1.36 mmol), and the reaction was heated to 120℃ and stirred for 48h. Upon completion, the reaction mixture was poured into aq. sat. $NaHCO_3$ and stirred until bubbling ceased. This solution was taken up into a separatory funnel and extracted with EtOAc, washed with brine, and dried under Na_2SO_4. Organic extracts were dried under vacuum. Purification by flash column chromatography (0–100% EtOAc in pentane) afforded **OX04958d** as a pale orange oil (63.8 mg, 18%). ^{1}H NMR (400 MHz, $CDCl_3$) δ 8.15–8.11 (m, 1H), 7.92 (s, 1H), 7.28 (t, *J* = 8.0 Hz, 1H), 7.15 (t, *J* = 2.1 Hz, 1H), 6.95 (dd, *J* = 10.1, 2.0 Hz, 1H), 6.88 (ddd, *J* = 8.1, 2.0, 0.8 Hz, 1H), 2.64 (d, *J* = 2.8 Hz, 2H), 2.60 (d, *J* = 2.9 Hz, 2H), 1.99–1.87 (m, 2H). ^{13}C NMR (101 MHz, $CDCl_3$) δ 158.1 (d, *J* = 248.6 Hz), 155.4, 142.6 (d, *J* = 6.5 Hz), 139.6, 139.1, 133.9, 130.6, 125.4, 125.0 (d, *J* = 3.4 Hz), 118.5 (d, *J* = 17.5 Hz), 116.6 (d, *J* = 20.5 Hz), 34.6, 32.2, 32.1. ^{19}F NMR (376 MHz, $CDCl_3$) δ -115.85 (dd, *J* = 10.1, 7.6 Hz). HRMS (ESI +ve) $C_{14}H_{13}ClFNO$ [M+H$^+$] calc. 266.0743, found 266.0751

OX04958

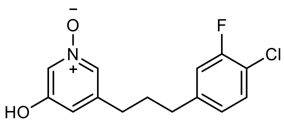

OX04958

To a solution of **OX04958c** (60 mg, 0.23 mmol) in CH_2Cl_2 (10 mL). 77% *meta*-chloroperoxybenzoic acid (83 mg, 0.36 mmol) was added and the reaction was stirred at room temperature for 2 h. Upon completion, aq. 2M NaOH (5 mL) was added to the reaction mixture. The pH was adjusted to 3-4 using aq. 1M HCl, and the aqueous phase was extracted with 2:1 $CHCl_3$:EtOH. The organic extract was dried and purified by flash column chromatography (0–5% MeOH in CH_2Cl_2) to afford **OX04958** as a pale yellow solid (40.3 mg, 65%). ^{1}H NMR (400 MHz, $CDCl_3$) δ 8.00 (d, *J* = 2.1 Hz, 1H), 7.64 (s, 1H), 7.28 (t, *J* = 7.2 Hz, 1H), 6.94–

6.90 (m, 2H), 6.86 (dd, *J* = 8.2, 2.0 Hz, 1H), 2.60 (t, *J* = 7.6 Hz, 2H), 2.53 (t, *J* = 7.7 Hz, 2H), 1.93–1.84 (m, 2H). ^{13}C NMR (101 MHz, $CDCl_3$) δ 158.1 (d, *J* = 249.5 Hz), 157.5, 142.1 (d, *J* = 6.4 Hz), 141.3, 130.6, 129.8, 126.7, 124.9 (d, *J* = 3.5 Hz), 119.8, 118.6 (d, *J* = 17.7 Hz), 116.6 (d, *J* = 20.6 Hz), 34.4, 32.1, 31.4. ^{19}F NMR (377 MHz, $CDCl_3$) δ -115.60 (dd, *J* = 10.1, 7.8 Hz). HRMS (ESI +ve) $C_{14}H_{13}ClFNO_2$ [M+H$^+$] calc. 282.0692, found 282.0701. HPLC 96% (AUC), t_R = 6.5 min.

OX04959a

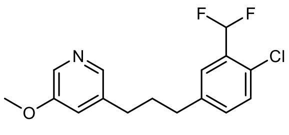

OX04959a

To a solution of **OX04955a** (100 mg, 0.35 mmol) in CH_2Cl_2 (8 mL) was added DAST (0.11 mL, 0.84 mmol) dropwise. The reaction was stirred for 16 h at rt before quenched with $NaHCO_3$ (10 mL, sat., aq.) and the two phases were separated. The organic layer was washed with $NaHCO_3$ (10 mL, sat., aq.), brine (15 mL), dried (Na_2SO_4), filtered and concentrated *in vacuo*. The residue was purified by flash column chromatography (10–30% EtOAc in petroleum ether) to afford **OX04959a** as a yellow oil (40 mg, 37%). ^{1}H NMR (400 MHz, $CDCl_3$) δ 8.16 (d, *J* = 2.8 Hz, 1H), 8.06 (d, *J* = 1.8 Hz, 1H), 7.47 (d, *J* = 2.2 Hz, 1H), 7.33 (dt, *J* = 8.3, 1.2 Hz, 1H), 7.21 (ddt, *J* = 8.2, 1.9, 0.9 Hz, 1H), 7.09–6.77 (m, 2H), 3.85 (s, 3H), 2.65 (dt, *J* = 17.4, 7.7 Hz, 4H), 2.03–1.91 (m, 2H). ^{13}C NMR (101 MHz, $CDCl_3$) δ 155.8, 142.4, 141.3, 137.7, 135.2, 132.1 (t, *J* = 2.0 Hz), 131.7, 130.3, 130.0, 126.8 (t, *J* = 5.8 Hz), 120.8, 112.2 (t, *J* = 238.0 Hz), 55.6, 34.7, 32.4, 32.3. ^{19}F NMR (376 MHz, $CDCl_3$) δ -115.0. LRMS (ESI +ve) 312 [M+H]$^+$. HRMS (ESI +ve) $C_{16}H_{16}ClF_2NO$ [M+H]$^+$ calc. 312.0961, found 312.0967.

OX04959b

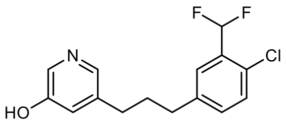

OX04959b

The demethylation using L-Selectride was adapted from a reported route.[1] L-Selectride (0.4 mL, 1 M in THF) was added to **OX04959a** (40 mg, 0.13 mmol) in THF (3 mL) and then the mixture was stirred at 70ºC for 48 h. After completion, the mixture was cooled to rt, quenched with MeOH, stirred for 30 min and concentrated *in vacuo*. The residue was then purified by flash column chromatography (0–8% MeOH in CH_2Cl_2) to afford **OX04959b** as a white solid (40 mg, 92%). ^{1}H NMR (400 MHz, $CDCl_3$) δ 8.12 (d, *J* = 2.7 Hz, 1H), 7.91 (d, *J* = 1.8 Hz, 1H), 7.45 (d, *J* = 2.1 Hz, 1H), 7.32 (dt, *J* = 8.2, 1.2 Hz, 1H), 7.22–7.18 (m, 1H), 7.14 (t, *J* = 2.2 Hz, 1H), 6.92 (t, *J* = 55.0 Hz, 1H), 2.64 (dt, *J* = 19.9, 7.7 Hz, 4H), 2.00–1.90 (m, 2H). ^{13}C NMR (101 MHz, $CDCl_3$) δ 155.4, 141.2, 139.5, 139.3, 134.1, 132.1 (t, *J* = 1.8 Hz), 130.0, 129.1, 127.2, 126.8 (t, *J* = 5.9 Hz), 125.2, 112.2 (t, *J* = 237.9 Hz), 34.8, 32.3, 32.2. ^{19}F NMR (376 MHz, $CDCl_3$) δ -115.0. HRMS (ESI +ve) $C_{15}H_{14}ClF_2NO$ [M+H]$^+$ calc. 298.0805, found 298.0814.

OX04959

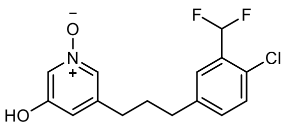

OX04959

To a solution of **OX04959b** (20 mg, 0.07 mmol) in CH_2Cl_2 (10 mL) at rt was added *m*-CPBA (1.5 eq.) and the resulting mixture stirred for 2 h. After completion, the reaction was quenched with $NaHCO_3$ (sat, aq.). The organic phase was washed with brine, dried (Na_2SO_4), filtered and concentrated *in vacuo*. Purification by flash column chromatography (0–6% MeOH in CH_2Cl_2) afforded **OX04959** as a white solid (9 mg, 43%). ^{1}H NMR (400 MHz, $CDCl_3$) δ 8.01 (s, 1H), 7.63 (s, 1H), 7.44 (d, J = 2.2 Hz, 1H), 7.33 (d, J = 8.2 Hz, 1H), 7.19 (dd, J = 8.2, 2.1 Hz, 1H), 7.08–6.77 (m, 2H), 2.65 (t, J = 7.7 Hz, 2H), 2.55 (t, J = 7.7 Hz, 2H), 1.97–1.85 (m, 2H). ^{13}C NMR (151 MHz, CDCl3) δ 157.5, 141.3, 140.7, 132.0, 131.9, 130.5, 130.1, 130.1, 126.8 (t, J = 5.9 Hz), 126.7, 119.8, 112.2 (t, J = 237.1 Hz), 34.6, 32.3, 31.6. ^{19}F NMR (376 MHz, $CDCl_3$) δ -115.0. LRMS (ESI +ve) 314 $[M+H]^+$. HRMS (ESI +ve) $C_{15}H_{14}ClF_2NO_2$ $[M+H]^+$ calc. 314.0754, found 314.0768. HPLC 98% (AUC), t_R = 6.8 min.

OX04960a

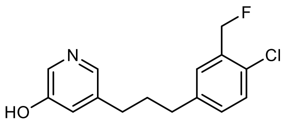

OX04960a

OX04955b (110 mg, 0.4 mmol) was dissolved in anhydrous CH_2Cl_2 (CH_2Cl_2) (4 mL) at room temperature under inert conditions. The solution was then cooled to 0°C and stirred for 5 minutes. Diethylaminosulfur trifluoride (DAST) (0.2 mL, 1.2 mmol) was added dropwise, and the mixture was allowed to warm gradually to room temperature. The reaction was stirred for 24 h. Upon completion, $NaHCO_3$ solution (4 mL) was added, followed by EtOAc. The organic layer was separated, dried over Na_2SO_4, and concentrated. Purification by flash chromatography (0–10% MeOH in CH_2Cl_2) afforded **OX04960a** as a brown solid (12 mg, 11% yield). ^{1}H NMR (400 MHz, $CDCl_3$) δ 8.14 (d, J = 2.7 Hz, 1H), 7.93 (d, J = 1.8 Hz, 1H), 7.28 (dd, J = 8.2, 1.3 Hz, 2H), 7.14–7.11 (m, 1H), 7.09 (dt, J = 7.9, 1.6 Hz, 1H), 5.54 (s, 1H), 5.42 (s, 1H), 2.63 (dt, J = 13.3, 7.7 Hz, 4H), 2.01–1.89 (m, 2H). ^{13}C NMR (101 MHz, $CDCl_3$) δ 154.8, 140.7, 139.7, 139.4, 134.1, 129.7, 129.7, 129.4, 128.6, 128.5, 124.8, 81.7 (d, J = 168.5 Hz), 34.6, 32.2, 32.1. ^{19}F NMR (377 MHz, $CDCl_3$) δ -216.85. HRMS (ESI +ve) $C_{15}H_{15}ClFNO$ $[M+H^+]$ calc. 280.0899, found 280.0964

OX04960

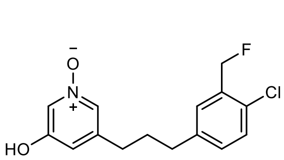

OX04960

To a solution of **OX04960a** (11.7 mg, 0.04 mmol, 1.0 eq.) in CH_2Cl_2 (7 mL), *meta*-chloroperoxybenzoic acid (1.5 eq.) was added at room temperature. The reaction progress was monitored by TLC. After 2 hours, $NaHCO_3$ solution (10 mL) was added. The mixture was diluted with CH_2Cl_2, and the organic layer was separated, washed with brine, and dried over Na_2SO_4. Purification by flash chromatography (0–10% MeOH in CH_2Cl_2) yielded **OX04960** (5 mg, 44% yield) as a light brown solid. ^{1}H NMR (600 MHz, $CDCl_3$) δ 7.99 (s, 1H), 7.63 (s, 1H), 7.29 (d, *J* = 8.1 Hz, 1H), 7.27 (s, 1H), 7.07 (d, *J* = 7.5 Hz, 1H), 6.92 (s, 1H), 5.48 (d, *J* = 47.2 Hz, 2H), 2.58 (dt, *J* = 54.0, 7.7 Hz, 4H), 1.91 (p, *J* = 7.7 Hz, 2H). ^{13}C NMR (151 MHz, $CDCl_3$) δ 157.3, 141.3, 140.1, 134.1 (d, *J* = 17.7 Hz), 129.9 (d, *J* = 5.3 Hz), 129.7, 129.6 (d, *J* = 3.0 Hz), 129.4, 128.3 (d, *J* = 8.7 Hz), 126.4, 119.8, 81.6 (d, *J* = 168.3 Hz), 34.3, 32.0, 31.4. ^{19}F NMR (377 MHz, CDCl3) δ -217.37. **HRMS (ESI +ve)** $C_{15}H_{16}ClFNO_2$ [$M+H^+$] calc. **296.0848**, found **296.0829**. HPLC 95% (AUC), t_R = 7.3 min.

OX04961a

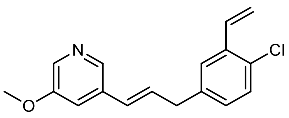

OX04961a

To a nitrogen-degassed microwave vial with **OX04956b** (1150 mg, 3.4 mmol), vinyl boronic ester (787 mg, 5.1 mmol) and Cs_2CO_3 (1111 mg, 10.2 mmol) in THF:H_2O mixture (9:1, 10 mL) was added PPh_3 (89 mg, 0.3 mmol) and $Pd(OAc)_2$ (38 mg, 0.2 mmol). After degassing 5 min with nitrogen, the vial was sealed and the mixture was placed on a pre-heated heating plate and stirred at 70°C for 25 h and then the mixture was diluted with EtOAc and filtered through Celite®. The organic layer was washed with brine (30 mL × 2), dried over Na_2SO_4 and concentrated *in vacuo*. **OX04961a** was purified via column chromatography (0–30% EtOAc in petroleum) as a yellow oil (543 mg, 56%). ^{1}H NMR (400 MHz, $CDCl_3$) δ 8.19 (d, *J* = 1.8 Hz, 1H), 8.16 (d, *J* = 2.8 Hz, 1H), 7.42 (d, *J* = 2.2 Hz, 1H), 7.30 (d, *J* = 8.2 Hz, 1H), 7.15 (dd, *J* = 2.8, 1.8 Hz, 1H), 7.13–7.05 (m, 2H), 6.42–6.38 (m, 2H), 5.74 (dd, *J* = 17.6, 1.1 Hz, 1H), 5.38 (dd, *J* = 11.0, 1.1 Hz, 1H), 3.85 (s, 3H), 3.56–3.52 (m, 2H). ^{13}C NMR (101 MHz, $CDCl_3$) δ 155.8, 140.8, 138.3, 136.5, 135.9, 133.6, 133.3, 131.4, 131.3, 129.9, 129.4, 127.9, 126.9, 116.9, 116.8, 55.7, 38.9. LRMS (ESI +ve) 286 $[M+H]^+$. HRMS (ESI +ve) $C_{17}H_{16}ClNO$ $[M+H]^+$ calc. 286.0993, found 286.0971.

OX04961b

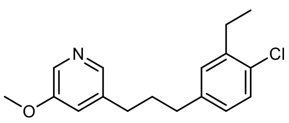

OX04961b

54 mg Pd/C (10% M/w) was added in one portion to a N_2 degassed solution of the **OX04961a** (540 mg, 1.9 mmol) in MeOH (60 mL) at rt, the atmosphere was replaced with H_2, and the reaction stirred for 1 h. After

completion, the reaction was filtered through Celite® using MeOH as an eluent, concentrated *in vacuo* and purified by flash column chromatography (30% EtOAc in Pentane) to afford the **OX04961b** as a yellow oil (215 mg, 39%). ^{1}H NMR (400 MHz, $CDCl_3$) δ 8.17 (d, J = 2.8 Hz, 1H), 8.09 (d, J = 1.8 Hz, 1H), 7.30–7.23 (m, 1H), 7.06–7.01 (m, 2H), 6.96 (dd, J = 8.1, 2.3 Hz, 1H), 3.87 (s, 3H), 2.74 (q, J = 7.5 Hz, 2H), 2.64 (q, J = 7.7 Hz, 4H), 2.01–1.90 (m, 2H), 1.24 (t, J = 7.5 Hz, 3H). ^{13}C NMR (101 MHz, $CDCl_3$) δ 155.8, 142.2, 141.6, 140.5, 138.3, 134.6, 131.4, 129.8, 129.4, 127.2, 121.2, 55.7, 34.8, 32.6, 32.4, 26.9, 14.3. LRMS (ESI +ve) 290 $[M+H]^+$. HRMS (ESI +ve) $C_{17}H_{20}ClNO$ $[M+H]^+$ calc. 290.1306, found 290.1323.

OX04961c

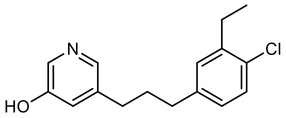

OX04961c

5 mL 48% HBr in H_2O was added to **OX04961b** (215 mg, 0.7 mmol) and then the mixture was stirred at 120ºC for 48 h. After completion of the reaction, cooled to rt, the mixture was neutralized with sat. aq. $NaHCO_3$ and extracted with EtOAc (3 × 40 mL). The combined organic extracts were washed with brine (100 mL), dried over Na_2SO_4, filtered, and concentrated under reduced pressure. The resulting residue was purified by flash column chromatography (0 – 6% MeOH in CH_2Cl_2) afforded **OX04961c** as a yellow oil (113 mg, 55%). ^{1}H NMR (400 MHz, $CDCl_3$) δ 11.27 (s, 1H), 8.10 (d, J = 2.6 Hz, 1H), 7.90 (d, J = 1.8 Hz, 1H), 7.22 (d, J = 8.1 Hz, 1H), 7.14 (t, J = 2.2 Hz, 1H), 7.01 (d, J = 2.2 Hz, 1H), 6.91 (dd, J = 8.1, 2.2 Hz, 1H), 2.71 (q, J = 7.5 Hz, 2H), 2.59 (td, J = 7.8, 2.4 Hz, 4H), 1.97–1.86 (m, 2H), 1.21 (t, J = 7.5 Hz, 3H). ^{13}C NMR (101 MHz, $CDCl_3$) δ 155.5, 141.6, 140.4, 139.9, 139.1, 133.8, 131.4, 129.7, 129.4, 127.2, 125.3, 34.8, 32.4, 32.3, 26.9, 14.3. **LRMS (ESI +ve)** 276 $[M+H]^+$. **HRMS (ESI +ve)** $C_{16}H_{18}ClNO$ $[M+H^+]$ calc. 276.1150, found 276.1124.

OX04961

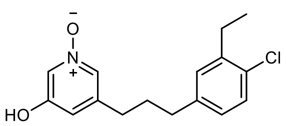

OX04961

3-(3-(4-chloro-3-ethylphenyl)propyl)-5-hydroxypyridine 1-oxide

To a solution of **OX04961c** (64 mg, 0.2 mmol) in CH_2Cl_2 (20 mL) at rt was added *m*-CPBA (1.5 eq.) and the resulting mixture stirred for 2 h. After completion, the reaction was quenched with $NaHCO_3$ (sat., aq.). The organic phase was washed with brine, dried (Na_2SO_4), filtered and concentrated *in vacuo*. Purification by flash column chromatography (0–6% MeOH in CH_2Cl_2) afforded **OX04961** as a yellow oil (28 mg, 41%). ^{1}H NMR (600 MHz, $CDCl_3$) δ 7.99 (s, 1H), 7.63 (s, 1H), 7.23 (d, J = 8.1 Hz, 1H), 7.00 (d, J = 2.2 Hz, 1H), 6.93–6.88 (m, 2H), 2.72 (q, J = 7.5 Hz, 2H), 2.58 (t, J = 7.6 Hz, 2H), 2.53 (t, J = 7.7 Hz, 2H), 1.89 (h, J

= 6.9 Hz, 2H), 1.22 (t, *J* = 7.5 Hz, 3H). ^{13}C NMR (151 MHz, $CDCl_3$) δ 157.5, 141.8, 141.6, 139.9, 131.6, 130.0, 129.7, 129.5, 127.2, 126.6, 119.9, 34.6, 32.3, 31.8, 26.9, 14.3. LRMS (ESI +ve) 292 $[M+H]^+$. HRMS (ESI +ve) $C_{16}H_{18}ClNO_2$ $[M+H]^+$ calc. 292.1099, found 292.1100. HPLC 96% (AUC), t_R = 3.0 min.

OX04962a

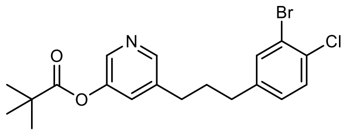

OX04962a

To a slurry of **OX04956d** (2.2 g, 6.8 mmol) and NEt_3 (1.43 mL, 10.2 mmol) in CH_2Cl_2 (80 mL) was added trimethylacetyl chloride (1.09 mL, 8.9 mmol) dropwise at 0ºC over 10 min. The mixture was then stirred at rt for 3 h before poured into H_2O (100 mL). The organic layer was separated, dried (Na_2SO_4) and concentrated *in vacuo* to afford **OX04962a** as an orange oil (2.7 g, 98%). ^{1}H NMR (400 MHz, $CDCl_3$) δ 8.32–8.28 (m, 1H), 8.23 (d, *J* = 2.4 Hz, 1H), 7.43 (d, *J* = 2.1 Hz, 1H), 7.35 (d, *J* = 8.2 Hz, 1H), 7.26–7.23 (m, 1H), 7.04 (dd, *J* = 8.2, 2.1 Hz, 1H), 2.63 (dt, *J* = 17.2, 7.7 Hz, 4H), 2.01–1.89 (m, 2H), 1.37 (s, 9H). ^{13}C NMR (101 MHz, $CDCl_3$) δ 176.8, 147.8, 147.0, 142.0, 141.1, 137.9, 133.7, 132.0, 130.3, 129.1, 128.7, 122.4, 39.3, 34.3, 32.2, 32.0, 27.2. LRMS (ESI +ve) 410 $[M+H]^+$. HRMS (ESI +ve) $C_{19}H_{21}BrClNO_2$ $[M+H]^+$ calc. 410.0517, found 410.0513.

OX04962b

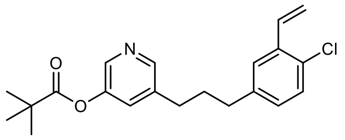

OX04962b

To a degassed microwave vial with **OX04962a** (701 mg, 1.7 mmol) and tributyl(vinyl)stannane (599 mg, 1.9 mmol) in DMF (4 mL) was added $Pd(PPh_3)4$ (109 mg, 0.1 mmol). The mixture was degassed for 5 min before the vial was sealed and placed on a pre-heated heating plate. The mixture was stirred for 24 h at 90ºC. After completion, the mixture was diluted with EtOAc (5 mL), poured into KF (5 mL, sat., aq.) then the mixture was stirred for 30 min before filtered through Celite®. The organic layer was washed with NH_4Cl (20 mL × 2, sat., aq.), dried (Na_2SO_4) and concentrated *in vacuo*. The crude product was purified with flash column chromatography (0–17% EtOAc in petroleum ether) to afford **OX04962b** as a colourless oil (207 mg, 34%). ^{1}H NMR (400 MHz, $CDCl_3$) δ 8.31 (d, *J* = 1.9 Hz, 1H), 8.24 (d, *J* = 2.5 Hz, 1H), 7.36 (d, *J* = 2.2 Hz, 1H), 7.29–7.24 (m, 2H), 7.09 (dd, *J* = 17.5, 11.0 Hz, 1H), 7.02 (dd, *J* = 8.2, 2.2 Hz, 1H), 5.74 (dd, *J* = 17.5, 1.1 Hz, 1H), 5.37 (dd, *J* = 11.0, 1.1 Hz, 1H), 2.66 (dt, *J* = 10.9, 7.6 Hz, 4H), 2.03–1.92 (m, 2H), 1.37 (s, 9H). ^{13}C NMR (101 MHz, $CDCl_3$) δ 176.8, 147.7, 147.0, 141.0, 140.4, 138.1, 135.6, 133.4, 130.9, 129.7,

129.1, 129.1, 126.6, 116.5, 39.3, 34.8, 32.4, 32.1, 27.2. LRMS (ESI +ve) 358 $[M+H]^+$. HRMS (ESI +ve) $C_{21}H_{24}ClNO_2$ $[M+H]^+$ calc. 358.1568, found 358.1576.

OX04962

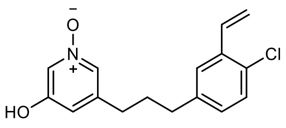

OX04962

To a mixture of **OX04962b** (30 mg, 0.1 mmol) in CH_2Cl_2 (10 mL) was added *m*-CPBA (1.5 eq.) in one portion at rt. The mixture was stirred at rt for 2 h then concentrated *in vacuo*, followed by purification with flash column chromatography (20–100% EtOAc in petroleum ether then 0–20% MeOH in EtOAc). The MeOH in EtOAc fractions were checked by thin layer chromatography, collected, concentrated *in vacuo* and dissolved in MeOH (10 mL). 4M aq. NaOH (2 mL) was added subsequently and the mixture was stirred for 16 h at rt. After completion, the mixture was concentrated *in vacuo*, taken up with sat. aq. NH_4Cl to adjust pH < 8 and extracted with CH_2Cl_2 (3 × 20 mL). The organic layer was washed with brine, dried with anhydrous Na_2SO_4, concentrated *in vacuo* and purified with flash column chromatography (0–10% MeOH in CH_2Cl_2) to afford **OX04962** as a brown solid (8.3 mg, 34%). ^{1}H NMR (400 MHz, $CDCl_3$) δ 8.00 (s, 1H), 7.63 (s, 1H), 7.33 (d, *J* = 2.1 Hz, 1H), 7.27–7.25 (m, 2H), 7.07 (dd, *J* = 17.5, 11.0 Hz, 1H), 6.97 (dd, *J* = 8.2, 2.2 Hz, 1H), 6.91 (s, 1H), 5.73 (dd, *J* = 17.6, 1.1 Hz, 1H), 5.37 (dd, *J* = 11.0, 1.1 Hz, 1H), 2.60 (t, *J* = 7.6 Hz, 2H), 2.54 (t, *J* = 7.8 Hz, 2H), 1.95–1.85 (m, 2H). ^{13}C NMR (151 MHz, $CDCl_3$) δ 157.6, 141.5, 139.9, 135.8, 133.3, 131.0, 129.9, 129.8, 129.0, 126.7, 126.6, 119.9, 116.7, 34.6, 32.3, 31.7. LRMS (ESI +ve) 290 $[M+H]^+$. HRMS (ESI +ve) $C_{16}H_{16}ClNO_2$ $[M+H]^+$ calc. 290.0942, found 290.0947. HPLC 95% (AUC), t_R = 8.0 min.

OX04963a

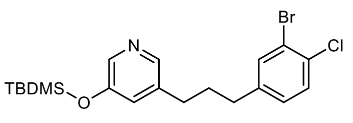

OX04963a

To a solution of **OX04956d** (4.8 g, 14.8 mmol) in chloroform (60 mL) was added TBDMSCl (3.3 g, 22.2 mmol) and imidazole (4.1 g, 59.1 mmol). The organic mixture was stirred at 60ºC for 16 h before washed with H_2O (50 mL × 2), brine (50 mL × 1), dried (Na_2SO_4), concentrated *in vacuo*. Purification with flash column chromatography (0–30% EtOAc in petroleum ether) afforded **OX04963a** as a yellow oil (1.7 g, 26%). ^{1}H NMR (400 MHz, $CDCl_3$) δ 8.07 (d, *J* = 2.7 Hz, 1H), 8.05 (d, *J* = 1.8 Hz, 1H), 7.43 (d, *J* = 2.0 Hz, 1H), 7.35 (d, *J* = 8.2 Hz, 1H), 7.04 (dd, *J* = 8.2, 2.1 Hz, 1H), 6.94 (dd, *J* = 2.6, 1.9 Hz, 1H), 2.59 (t, *J* = 7.7 Hz, 4H), 1.96–1.86 (m, 2H), 0.99 (s, 9H), 0.21 (s, 6H). ^{13}C NMR (101 MHz, $CDCl_3$) δ 157.6, 144.1, 141.6, 139.8, 133.5, 131.4, 129.9, 129.7, 127.5, 126.6, 119.9, 34.6, 32.4, 31.8, 25.2, 20.9, 14.3. LRMS (ESI +ve) 440 $[M+H]^+$. HRMS (ESI +ve) $C_{20}H_{27}BrClNOSi$ $[M+H]^+$ calc. 440.0807, found 440.0815.

OX04963b

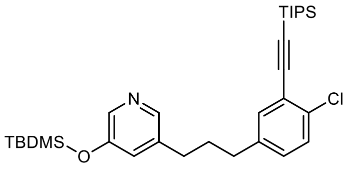

OX04963b

To a microwave vial was added **OX04963a** (773 mg, 1.8 mmol), PPh_3 (92 mg, 0.4 mmol) and ethynyltriisopropylsilane (352 mg, 1.9 mmol) sequentially before the addition of degassed THF/NEt_3- mixture (3:1, 8 mL). The mixture was degassed for 5 min before adding $Pd_2(PPh_3)_2Cl_2$ (123 mg, 0.2 mmol) and CuI (134 mg, 0.7 mmol). The mixture was degassed for another 5 min then the vial was sealed, placed on a 60°C pre-heated heating plate and stirred for 16 h. After completion, the mixture was diluted with EtOAc (40 mL), filtered through Celite® and concentrated *in vacuo*. The dried mixture was taken up with CH_2Cl_2 (50 mL), washed with brine (50 mL × 2), dried (Na_2SO_4) and concentrated *in vacuo*. The residue was then purified by flash column chromatography (0–100% CH_2Cl_2 in petroleum ether then 0–10% EtOAc in CH_2Cl_2) to afford **OX04963b** as a transparent oil (660 mg, 69%). ^{1}H NMR (400 MHz, $CDCl_3$) δ 8.07 (d, *J* = 2.7 Hz, 2H), 7.31 (d, *J* = 2.2 Hz, 1H), 7.29 (d, *J* = 8.3 Hz, 1H), 7.03 (dd, *J* = 8.2, 2.2 Hz, 1H), 6.95 (dd, *J* = 2.6, 1.9 Hz, 1H), 2.63–2.55 (m, 4H), 1.96–1.86 (m, 2H), 1.15 (s, 21H), 0.99 (s, 9H), 0.21 (s, 6H). ^{13}C NMR (101 MHz, $CDCl_3$) δ 152.2, 143.0, 140.3, 140.3, 138.0, 134.0, 133.6, 129.6, 129.3, 127.0, 123.4, 103.4, 96.6, 34.6, 32.5, 32.2, 25.8, 18.8, 18.4, 11.4, -4.3. LRMS (ESI +ve) 542 $[M+H]^+$. HRMS (ESI +ve) $C_{31}H_{48}ClNOSi_2$ $[M+H]^+$ calc. 542.3036, found 542.3040.

OX04963

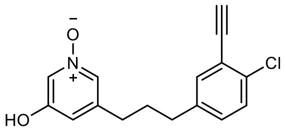

OX04963

To a solution of **OX04963b** (60 mg, 0.1 mmol) in CH_2Cl_2 (10 mL) was added *m*-CPBA (60 mg, 0.27 mmol) in one portion at rt. The mixture was stirred for 2 h before adding $NaHCO_3$ (20 mL, sat., aq.) to quench the reaction. The organic layer was separated and washed with $NaHCO_3$ (25 mL × 2), brine (25 mL), dried (Na_2SO_4) and concentrated *in vacuo*. The resulting while solid was dissolved in THF (10 mL) and TBAF (0.3 mL, 1M in THF) was added at 0ºC. The mixture was stirred for 1 h and MeOH (10 mL) was added, then the mixture was concentrated *in vacuo*. Flash column chromatography (20–100% EtOAc in petroleum ether, then 0–15% MeOH in EtOAc) afforded **OX04963** as a yellow oil (7.0 mg, 22%). ^{1}H NMR (400 MHz, $CDCl_3$) δ 7.93 (t, *J* = 1.9 Hz, 1H), 7.56 (d, *J* = 1.5 Hz, 1H), 7.26 (s, 1H), 7.22 (d, *J* = 14.1 Hz, 1H), 7.00 (dd, *J* = 8.3, 2.2 Hz, 1H), 6.85 (d, *J* = 1.6 Hz, 1H), 3.29 (s, 1H), 2.49 (dt, *J* = 20.6, 7.6 Hz, 4H), 1.82 (tt, *J* = 9.2, 6.7 Hz, 2H). ^{13}C NMR (101 MHz, $CDCl_3$) δ 157.6, 141.3, 139.8, 134.1, 133.9, 130.2, 129.8, 129.5, 126.7,

122.1, 119.8, 82.5, 80.4, 34.3, 32.2, 31.5. LRMS (ESI +ve) 288 $[M+H]^+$. HRMS (ESI +ve) $C_{16}H_{14}ClNO_2$ $[M+H]^+$ calc. 288.0786, found 288.0800. HPLC 99% (AUC), t_R = 6.6 min.

OX04964a

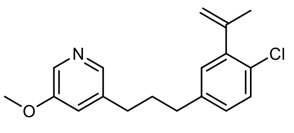

OX04964a

To a degassed microwave vial with **OX04956c** (339 mg, 1.45 mmol) and tributyl(prop-1-en-2-yl)stannane (660 mg, 2.0 mmol) in DMF (4 mL) was added $Pd(PPh_3)_4$ (173 mg, 0.15 mmol). The mixture was degassed for 5 min before the vial was sealed and placed on a pre-heated heating plate. The mixture was stirred for 24 h at 90°C. After completion, the mixture was diluted with EtOAc (5 mL), poured into KF (5 mL, sat., aq.) then the mixture was stirred for 30 min before filtered through Celite®. The organic layer was washed with NH_4Cl (20 mL × 2, sat., aq.), dried (Na_2SO_4) and concentrated *in vacuo*. The crude product was purified with flash column chromatography (0–30% EtOAc in petroleum ether) to afford **OX04964a** as a colourless oil (170 mg, 39%). ^{1}H NMR (400 MHz, $CDCl_3$) δ 8.15 (d, J = 2.8 Hz, 1H), 8.07 (d, J = 1.8 Hz, 1H), 7.30–7.22 (m, 1H), 7.00 (td, J = 3.0, 1.2 Hz, 3H), 5.22 (p, J = 1.6 Hz, 1H), 4.96 (dq, J = 1.9, 1.0 Hz, 1H), 3.85 (s, 3H), 2.62 (q, J = 7.2 Hz, 4H), 2.10 (dd, J = 1.5, 0.9 Hz, 3H), 1.98–1.91 (m, 2H). ^{13}C NMR (101 MHz, $CDCl_3$) δ 155.7, 144.6, 142.7, 142.5, 140.5, 138.0, 135.0, 129.9, 129.6, 129.4, 128.3, 120.8, 116.2, 55.6, 34.7, 32.5, 32.4, 23.5. LRMS (ESI +ve) 302 $[M+H]^+$. HRMS (ESI +ve) $C_{18}H_{20}ClNO$ $[M+H]^+$ calc. 302.1306, found 302.1311.

OX04964b

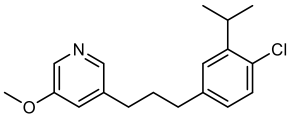

OX04964b

7 mg PtO_2 was added in one portion to a N_2 degassed solution of the **OX04964a** (130 mg, 0.43 mmol) in MeOH (180 mL) at rt, the atmosphere was replaced with H_2, and the reaction stirred for 1 h. After completion, the reaction was filtered through Celite® using MeOH as an eluent, concentrated *in vacuo* and purified by flash column chromatography (30% EtOAc in Pentane) to afford **OX04964b** as a colourless oil (84 mg, 64%). ^{1}H NMR (400 MHz, $CDCl_3$) δ 8.15 (d, J = 2.8 Hz, 1H), 8.07 (d, J = 1.8 Hz, 1H), 7.24 (d, J = 8.1 Hz, 1H), 7.07 (d, J = 2.2 Hz, 1H), 7.03–6.99 (m, 1H), 6.92 (dd, J = 8.1, 2.2 Hz, 1H), 3.85 (s, 3H), 3.38 (p, J = 6.9 Hz, 1H), 2.62 (td, J = 7.7, 3.8 Hz, 4H), 2.00–1.88 (m, 2H), 1.23 (d, J = 6.9 Hz, 6H). ^{13}C NMR (101 MHz, $CDCl_3$) δ 155.8, 145.6, 142.3, 140.6, 138.2, 134.7, 131.0, 129.4, 127.0, 126.7, 121.0, 55.6, 35.0,

32.6, 32.4, 30.2, 22.8. LRMS (ESI +ve) 304 $[M+H]^+$. HRMS (ESI +ve) $C_{18}H_{22}ClNO$ $[M+H]^+$ calc. 304.1463, found 304.1467.

OX04964c

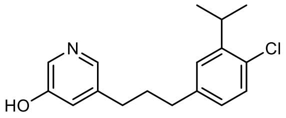

OX04964c

3 mL 48% HBr in H_2O was added to **OX04964b** (80 mg, 0.26 mmol) and then the mixture was stirred at 120ºC for 48 h. After completion of the reaction, cooled to rt, the mixture was neutralized with sat. aq. $NaHCO_3$ and extracted with EtOAc (3 × 30 mL). The combined organic extracts were washed with brine (100 mL), dried over Na_2SO_4, filtered, and concentrated under reduced pressure. The resulting residue was purified by flash column chromatography (5% MeOH in CH_2Cl_2) afforded **OX04964c** as a brown solid (46 mg, 60%). ^{1}H NMR (400 MHz, $CDCl_3$) δ 8.14 (d, *J* = 2.7 Hz, 1H), 7.92 (d, *J* = 1.8 Hz, 1H), 7.23 (d, *J* = 8.1 Hz, 1H), 7.16–7.13 (m, 1H), 7.06 (d, *J* = 2.2 Hz, 1H), 6.91 (dd, *J* = 8.1, 2.2 Hz, 1H), 3.37 (hept, *J* = 6.7 Hz, 1H), 2.66–2.57 (m, 4H), 1.99–1.87 (m, 2H), 1.23 (d, *J* = 6.9 Hz, 6H). ^{13}C NMR (101 MHz, $CDCl_3$) δ 155.2, 145.7, 140.5, 139.8, 139.6, 134.0, 131.0, 129.5, 127.0, 126.8, 125.2, 35.0, 32.4, 32.3, 30.2, 22.8. LRMS (ESI +ve) 290 $[M+H]^+$. HRMS (ESI +ve) $C_{17}H_{20}ClNO$ $[M+H]^+$ calc. 290.1306

, found 290.1313.

OX04964

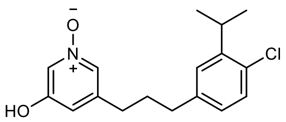

OX04964

To a solution of **OX04964c** (30 mg, 0.1 mmol) in CH_2Cl_2 (10 mL) at rt was added *m*-CPBA (1.5 eq.) and the resulting mixture stirred for 2 h. After completion, the reaction was quenched with $NaHCO_3$ (sat., aq.). The organic phase was washed with brine, dried (Na_2SO_4), filtered and concentrated *in vacuo*. Purification by flash column chromatography (0–8% MeOH in CH_2Cl_2) afforded **OX04964** as a yellow oil (19 mg, 60%). ^{1}H NMR (400 MHz, $CDCl_3$) δ 8.00 (s, 1H), 7.63 (s, 1H), 7.24 (d, *J* = 8.1 Hz, 1H), 7.05 (d, *J* = 2.2 Hz, 1H), 6.93–6.87 (m, 2H), 3.37 (p, *J* = 6.8 Hz, 1H), 2.56 (dt, *J* = 23.1, 7.7 Hz, 4H), 1.90 (h, *J* = 6.8 Hz, 2H), 1.23 (d, *J* = 6.9 Hz, 6H). ^{13}C NMR (151 MHz, $CDCl_3$) δ 157.5, 145.8, 141.6, 140.0, 131.3, 130.0, 129.6, 126.9, 126.7, 126.6, 119.9, 34.8, 32.3, 31.8, 30.2, 22.8. **LRMS (ESI +ve)** 306 $[M+H]^+$. **HRMS (ESI +ve)** $C_{17}H_{20}ClNO_2$ $[M+H^+]$ calc. 306.1255, found 306.1267. HPLC 99% (AUC), t_R = 6.5 min.

OX04965a

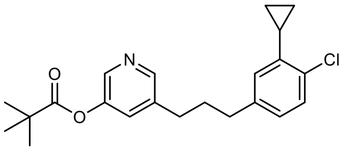

OX04965a

To CH_2Cl_2 (4 mL) was added Et_2Zn (1.3 mL, 1.0 M in hexane). The solution was cooled in an ice bath and TFA (140 mg, 1.2 mmol) was then added dropwise into the reaction mixture. Upon stirring for 30 min, a solution of CH_2I_2 (330 mg, 1.2 mmol) was added at 0ºC. After an additional 20 min of stirring, a solution of **OX04962b** (44 mg, 0.1 mmol) in CH_2Cl_2 (4 mL) was added, and the ice bath was removed. The reaction mixture was stirred for 16 h before it was quenched with $NaHCO_3$ (10 mL, sat., aq.) and the phases were separated. The organic layer was washed with NH_4Cl (20 mL × 2, sat., aq.), brine (15 mL) and then dried (Na_2SO_4), filtered and concentrated *in vacuo*. The residue was purified by flash column chromatography (10–18% EtOAc in petroleum ether) to afford **OX04965a** as a beige oil (32 mg, 92%). ^{1}H NMR (400 MHz, $CDCl_3$) δ 8.30 (d, *J* = 1.8 Hz, 1H), 8.23 (d, *J* = 2.5 Hz, 1H), 7.27–7.20 (m, 2H), 6.91 (dd, *J* = 8.1, 2.2 Hz, 1H), 6.72 (d, *J* = 2.2 Hz, 1H), 2.64 (t, *J* = 7.7 Hz, 2H), 2.58 (t, *J* = 7.7 Hz, 2H), 2.22–2.12 (m, 1H), 1.98–1.87 (m, 2H), 1.37 (s, 9H), 1.03–0.96 (m, 2H), 0.70–0.63 (m, 2H). ^{13}C NMR (101 MHz, $CDCl_3$) δ 176.8, 147.7, 147.1, 141.0, 140.8, 140.2, 138.2, 132.9, 129.1, 129.1, 126.8, 126.3, 39.3, 34.9, 32.4, 32.1, 27.2, 13.4, 8.1. LRMS (ESI +ve) 372 $[M+H]^+$. HRMS (ESI +ve) $C_{22}H_{26}ClNO_2$ $[M+H]^+$ calc. 372.1725, found 372.1713.

OX04965

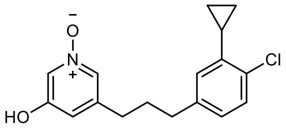

OX04965

To a mixture of **OX04965a** (20 mg, 0.05 mmol) in CH_2Cl_2 (10 mL) was added *m*-CPBA (1.5 eq.) in one portion at rt. The mixture was stirred at rt for 2 h then concentrated *in vacuo*, followed by purification with flash column chromatography (20–100% EtOAc in petroleum ether then 0–20% MeOH in EtOAc). The MeOH in EtOAc fractions were checked by thin layer chromatography, collected, concentrated *in vacuo* and dissolved in MeOH (10 mL). 4M aq. NaOH (2 mL) was added subsequently and the mixture was stirred for 16 h at rt. After completion, the mixture was concentrated *in vacuo*, taken up with sat. aq. NH_4Cl to adjust pH < 8 and extracted with CH_2Cl_2 (3 × 20 mL). The organic layer was washed with brine, dried with anhydrous Na_2SO_4, concentrated *in vacuo* and purified with flash column chromatography (0–10% MeOH in CH_2Cl_2) to afford **OX04965** as a white solid (14 mg, 86%). ^{1}H NMR (400 MHz, $CDCl_3$) δ 8.01 (t, *J* = 1.9 Hz, 1H), 7.63 (d, *J* = 1.5 Hz, 1H), 7.24 (d, *J* = 8.1 Hz, 1H), 6.93–6.83 (m, 2H), 6.69 (d, *J* = 2.1 Hz, 1H), 2.52 (dt, *J* = 14.7, 7.7 Hz, 4H), 2.16 (tt, *J* = 8.5, 5.3 Hz, 1H), 1.85 (tt, *J* = 9.3, 6.7 Hz, 2H), 1.05–0.93 (m, 2H), 0.72–0.62 (m, 2H). ^{13}C NMR (101 MHz, $CDCl_3$) δ 157.5, 141.6, 141.0, 139.8, 133.1, 129.9, 129.2, 126.7,

126.6, 126.3, 119.9, 34.7, 32.3, 31.8, 13.4, 8.1. LRMS (ESI +ve) 304 $[M+H]^+$. HRMS (ESI +ve) $C_{17}H_{18}ClNO_2$ $[M+H]^+$ calc. 304.1099, found 304.1097. HPLC 95% (AUC), t_R = 8.0 min.

OX04966a

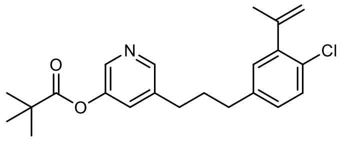

OX04966a

To an N2 degassed mixture of **OX04962a** (839 mg, 2.05 mmol) and tributyl(1-methylethenyl)Stannane (1362 mg, 4.10 mmol) in DMF (4.5 mL) was added $Pd(PPh_3)_4$ (118 mg, 0.10 mmol). The mixture was subsequently degassed for another 5 min before the mixture was heated to 90ºC and stirred for 72 h. After completion, the mixture was poured into sat. aq. KF (10 mL), stirred for 30 min and EtOAc (2 × 30 mL) was added to extract the organic layer. The organic layer was then washed with sat. aq. KF (1 × 10 mL), water (3 × 30 mL), brine (1 × 50 mL), dried over Na_2SO_4 and saturated *in vacuo*. The resulting brown oil was purified with flash column chromatography (0–30% EtOAc in petroleum ether) to afford **OX04966a** as a yellow oil (677 mg, 89%). ^{1}H NMR (400 MHz, $CDCl_3$) δ 8.30 (s, 1H), 8.22 (s, 1H), 7.26–7.23 (m, 2H), 7.02–6.98 (m, 2H), 5.21 (p, *J* = 1.6 Hz, 1H), 4.95 (dq, *J* = 1.9, 0.9 Hz, 1H), 2.64 (dt, *J* = 17.2, 7.7 Hz, 4H), 2.09 (dd, *J* = 1.5, 0.9 Hz, 3H), 2.01–1.90 (m, 2H), 1.36 (s, 9H). ^{13}C NMR (101 MHz, $CDCl_3$) δ 176.8, 147.1, 144.6, 142.8, 141.0, 140.3, 129.9, 129.6, 129.4, 129.1, 128.3, 116.2, 39.3, 34.7, 32.4, 32.2, 27.2, 23.5. **HRMS (ESI +ve)** $C_{22}H_{26}ClNO_2$ $[M+H^+]$ calc. 372.1725, found 372.1742.

OX04966b

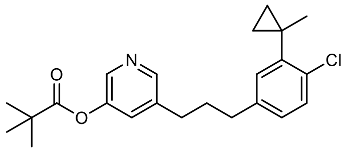

OX04966b

To an mixture of $ZnEt_2$ (8.1 mL, 1M in hexane) in CH_2Cl_2 (18 mL) was added TFA (0.62 mL) dropwise at 0ºC and the resulting white slurry was stirred vigorously for 30 min at 0ºC. Diiodomethane (0.65 mL) was added dropwise to the mixture at 0ºC to from a Clear solution, followed by stirring for 30 min. Then **OX04966a** (300 mg, 0.8 mmol) in CH_2Cl_2 (5 mL) was added to the mixture at 0ºC, the ice bath was removed and the reaction mixture was stirred for 16 h. After completion, the mixture was poured into sat. $NaHCO_3$ (50 mL) and separated. The organic layer was washed with sat. aq. NH_4Cl (2 × 50 mL), brine (1 × 30 mL) and concentrated *in vacuo*. Purification with flash column chromatography (0–20% EtOAc in petroleum ether) afforded **OX04966b** as a colorless oil (98 mg, 31%). ^{1}H NMR (400 MHz, $CDCl_3$) δ 8.23 (d, *J* = 1.9 Hz, 1H), 8.15 (d, *J* = 2.5 Hz, 1H), 7.18 (d, *J* = 2.4 Hz, 1H), 7.14 (d, *J* = 8.1 Hz, 1H), 7.07 (d, *J* = 2.2 Hz, 1H), 6.86 (dd, *J* = 8.1, 2.3 Hz, 1H), 2.55 (dt, *J* = 20.9, 7.7 Hz, 4H), 1.92–1.83 (m, 2H), 1.29 (s, 9H), 1.26 (s, 3H), 0.76–0.70 (m, 2H), 0.70–0.63 (m, 2H). ^{13}C NMR (101 MHz, $CDCl_3$) δ 176.8, 147.7, 147.0, 143.9,

140.9, 140.2, 138.2, 133.2, 131.5, 129.6, 129.0, 127.6, 39.3, 34.7, 32.4, 32.2, 27.2, 25.2, 20.9, 14.2. HRMS (ESI +ve) $C_{23}H_{28}ClNO_2$ $[M+H]^+$ calc. 386.1881, found 386.1906.

OX04966

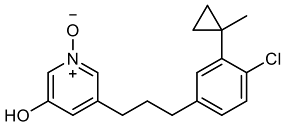

OX04966

To a mixture of **OX04966b** (98 mg, 0.3 mmol) in CH_2Cl_2 (30 mL) was added *m*-CPBA (1.5 eq.) in one portion at rt. The mixture was stirred at rt for 2 h then concentrated *in vacuo*, followed by purification with flash column chromatography (20–100% EtOAc in petroleum ether then 0–20% MeOH in EtOAc). The MeOH in EtOAc fractions were checked by thin layer chromatography, collected, concentrated *in vacuo* and dissolved in MeOH (10 mL). 4M aq. NaOH (5 mL) was added subsequently and the mixture was stirred for 16 h at rt. After completion, the mixture was concentrated *in vacuo*, taken up with sat. aq. NH_4Cl to adjust pH < 8 and extracted with CH_2Cl_2 (3 × 20 mL). The organic layer was washed with brine, dried with anhydrous Na_2SO_4, concentrated *in vacuo* and purified with flash column chromatography (0–10% MeOH in CH_2Cl_2) to afford **OX04966** as a white solid (80 mg, 99%). ^{1}H NMR (400 MHz, $CDCl_3$) δ 8.01 (s, 1H), 7.63 (s, 1H), 7.22 (d, J = 8.1 Hz, 1H), 7.13 (d, J = 2.3 Hz, 1H), 6.94–6.89 (m, 2H), 2.55 (dt, J = 15.1, 7.7 Hz, 4H), 1.95–1.84 (m, 2H), 1.33 (s, 3H), 0.83–0.77 (m, 2H), 0.77–0.71 (m, 2H). ^{13}C NMR (101 MHz, $CDCl_3$) δ 157.6, 144.1, 141.6, 139.8, 133.5, 131.4, 129.9, 129.7, 127.5, 126.6, 119.9, 34.6, 32.4, 31.8, 25.2, 20.9, 14.3. LRMS (ESI +ve) 318 $[M+H]^+$. HRMS (ESI +ve) $C_{18}H_{20}ClNO_2$ $[M+H]^+$ calc. 318.1255, found 318.1253. HPLC 99% (AUC), t_R = 8.6 min.

References

1. Makino, K. *et al.* Chemoselective Demethylation of Methoxypyridine. *Synlett* **30**, 951–954 (2019).

NMR

OX04539a

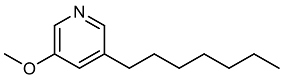

OX04539a

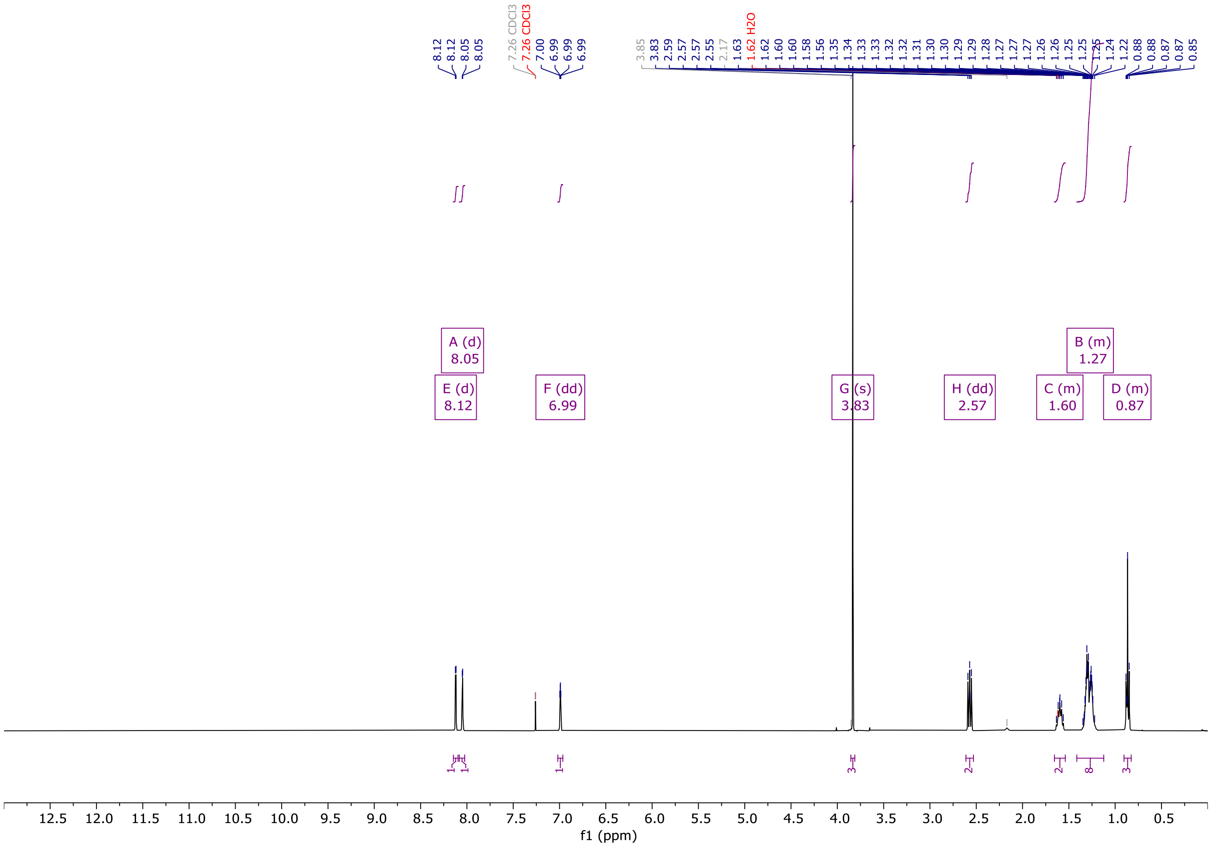

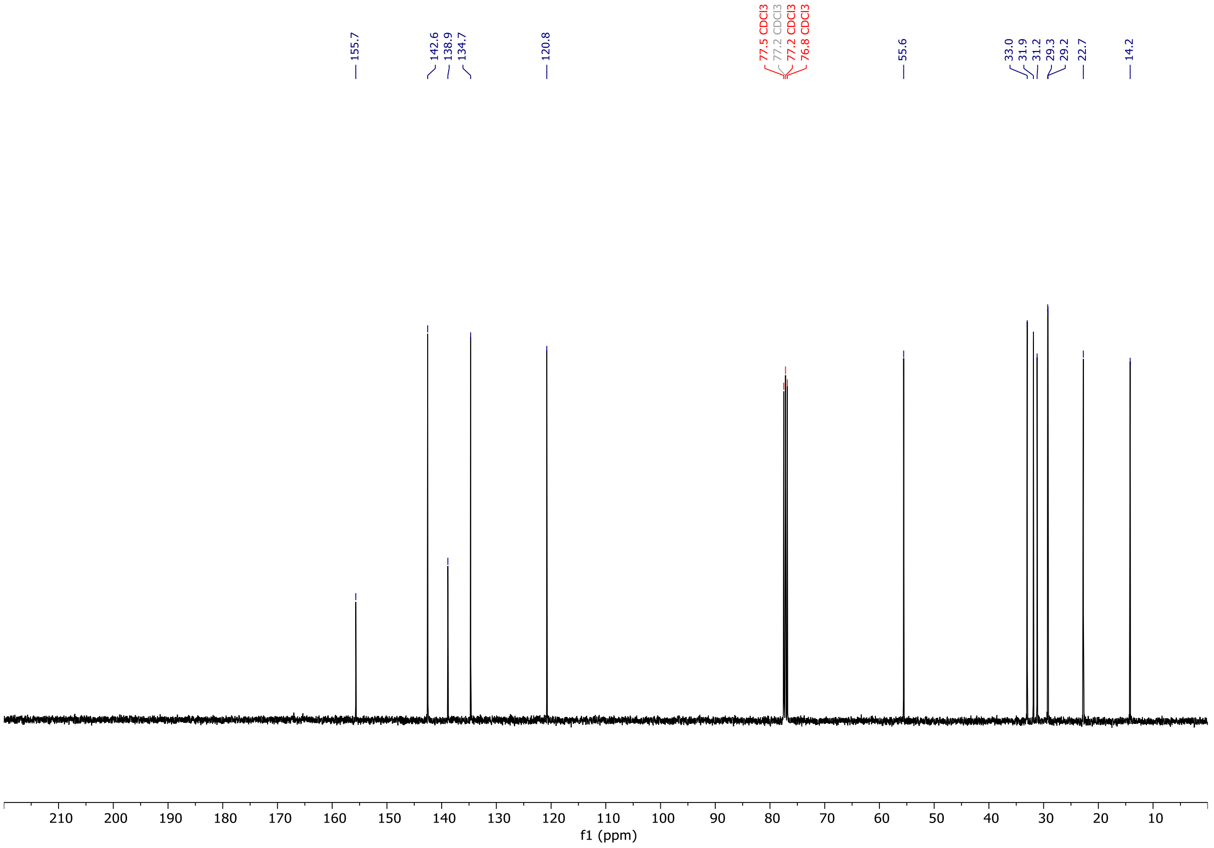

OX04539b

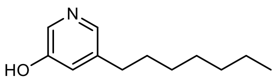

OX04539b

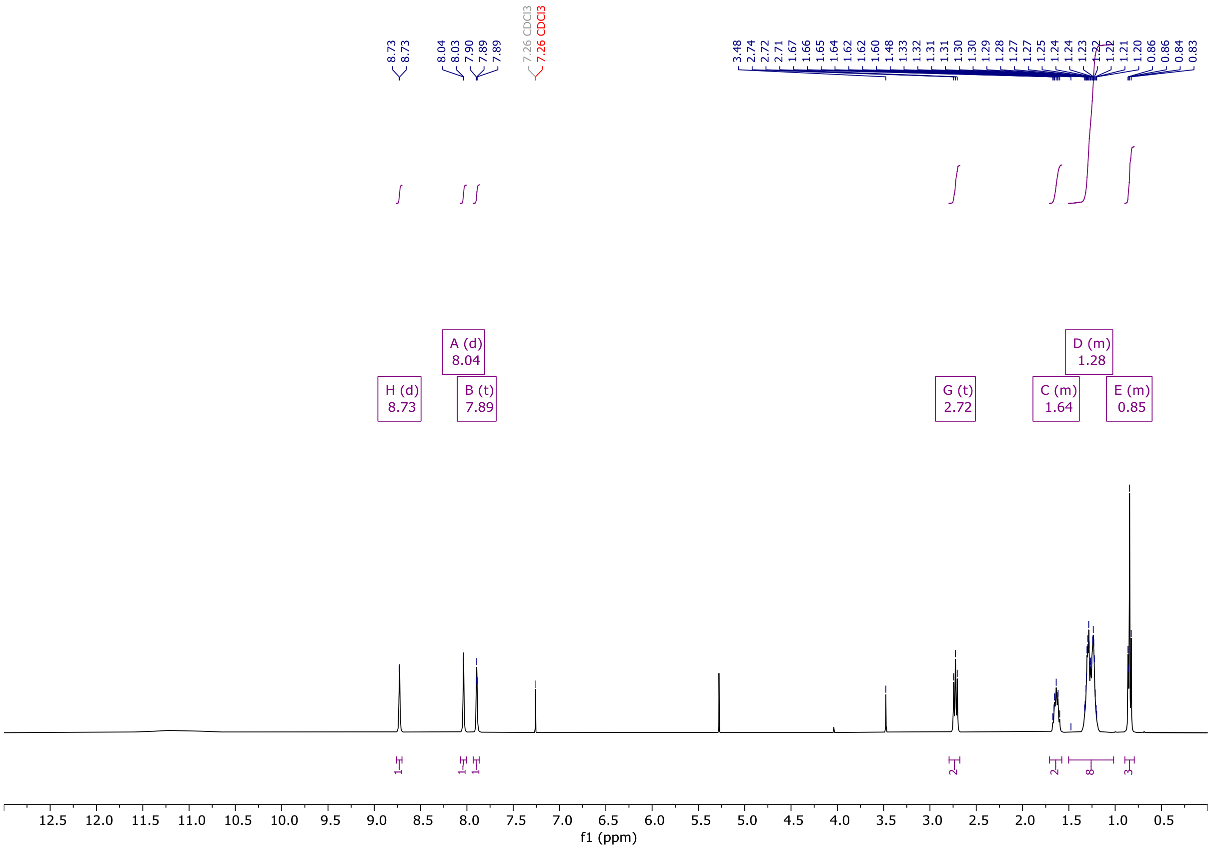

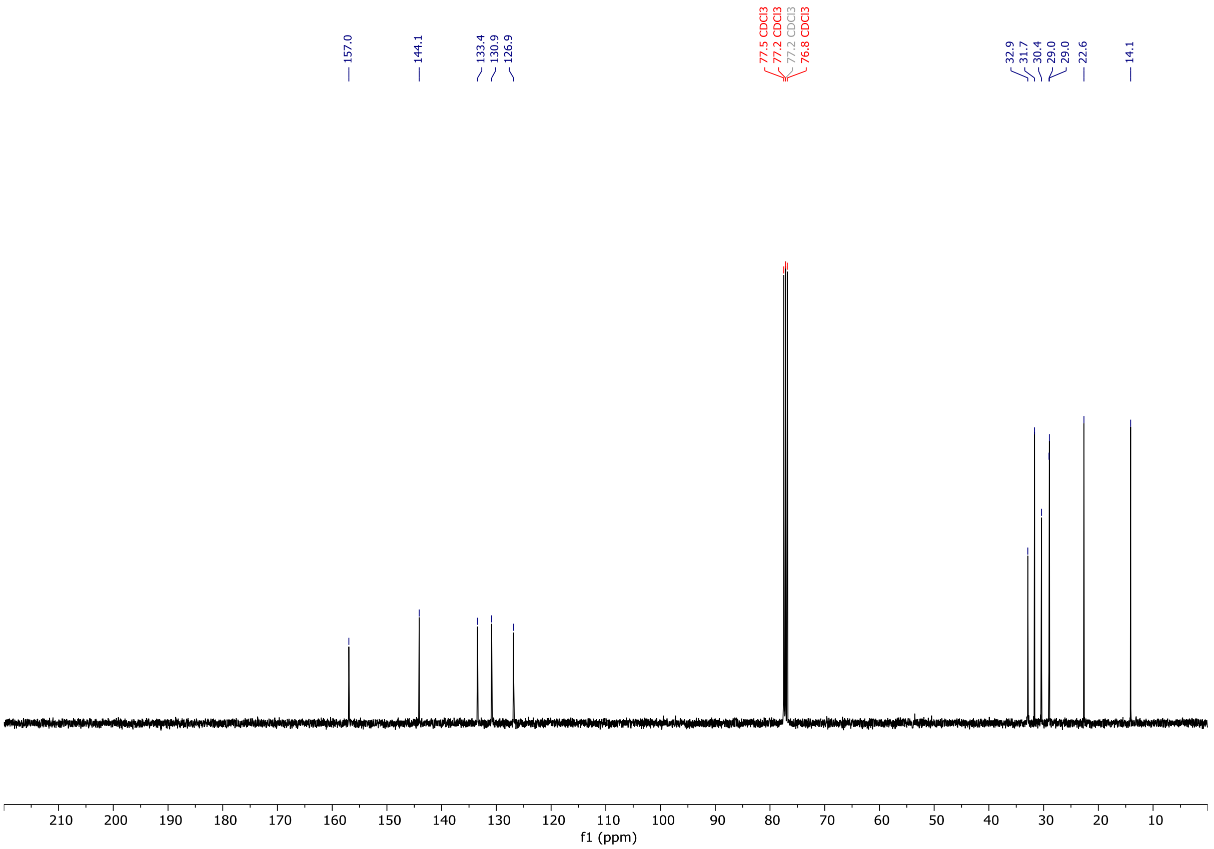

OX04539

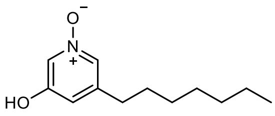

OX04539

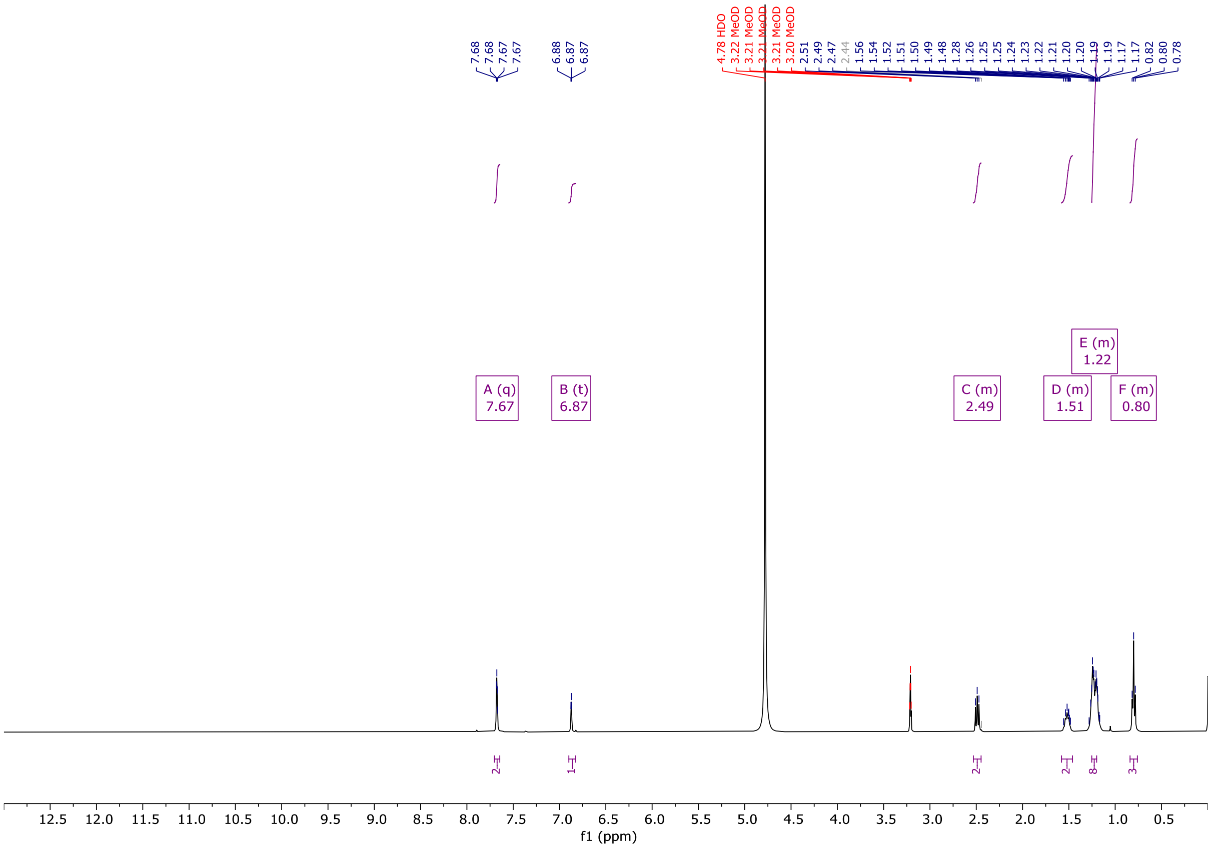

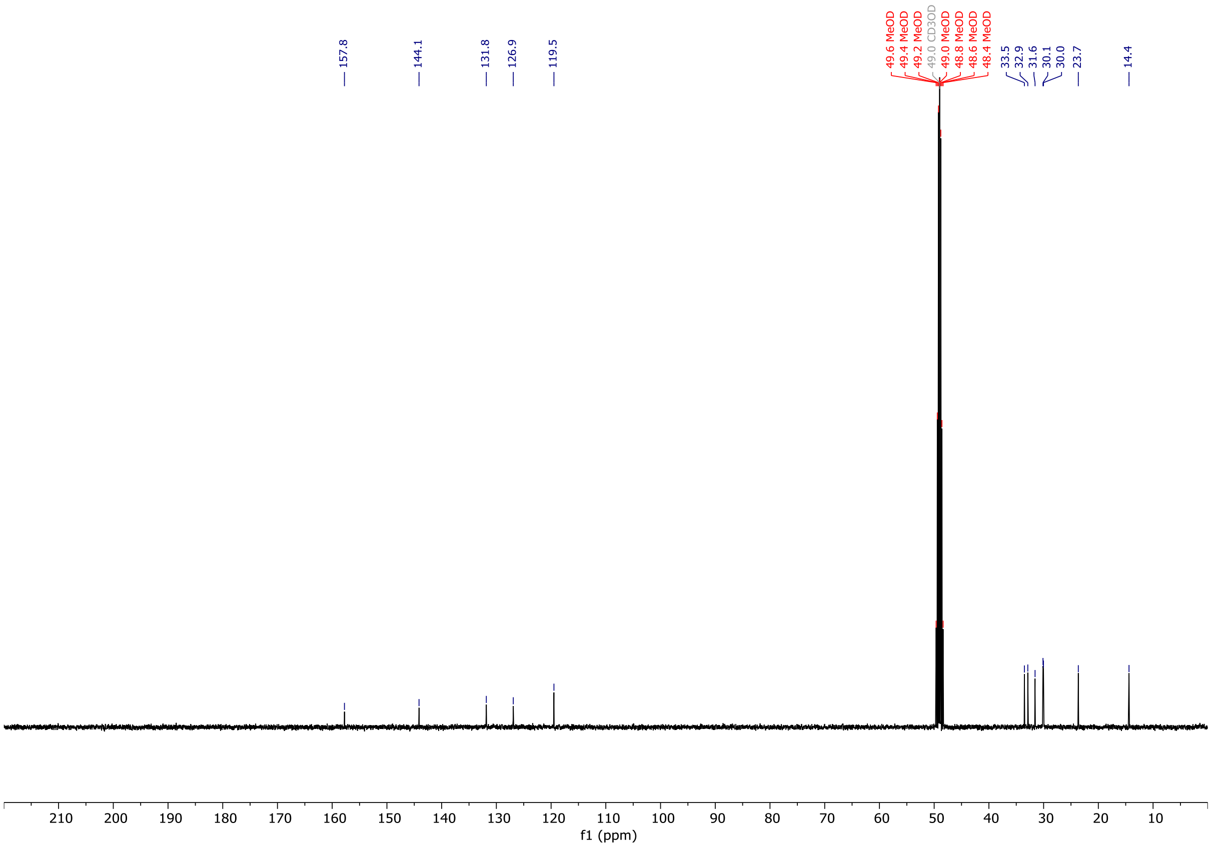

OX04540a

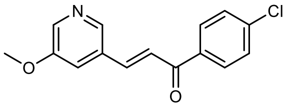

OX04540a

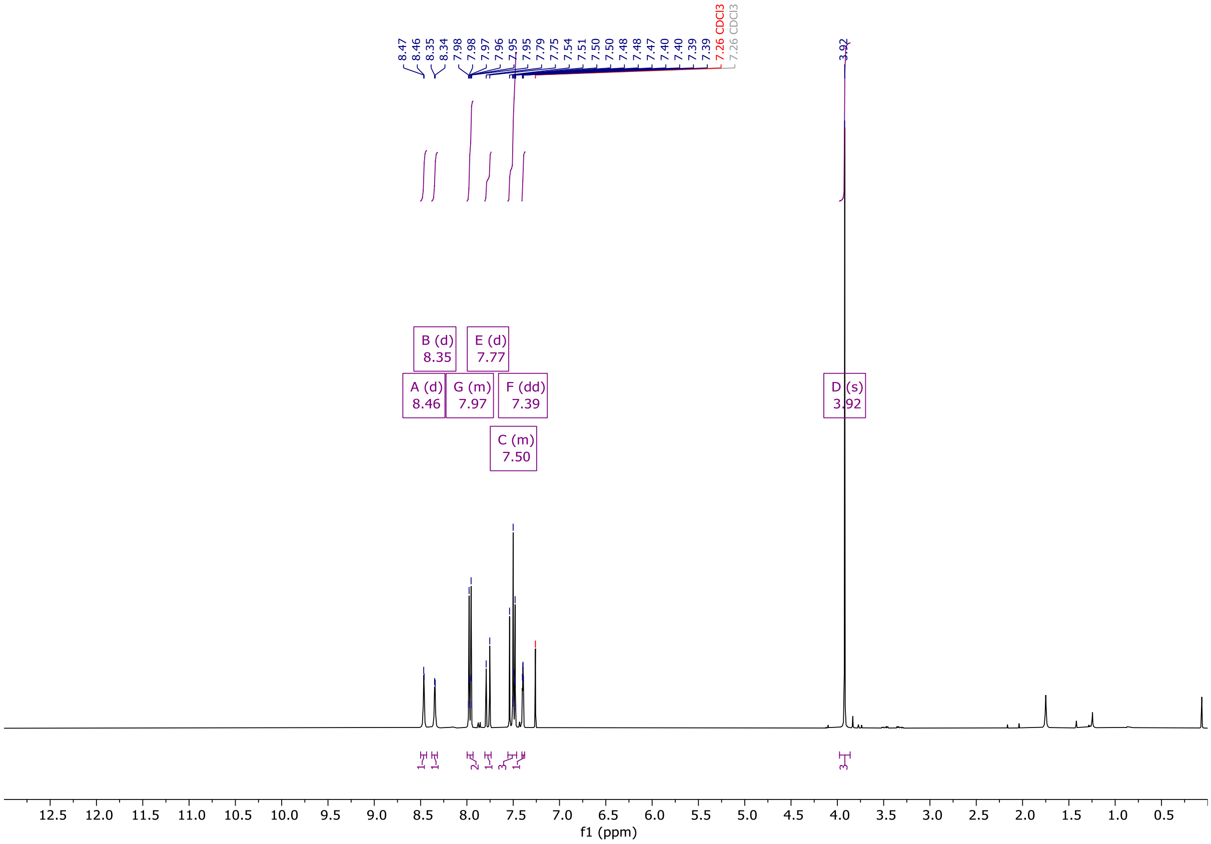

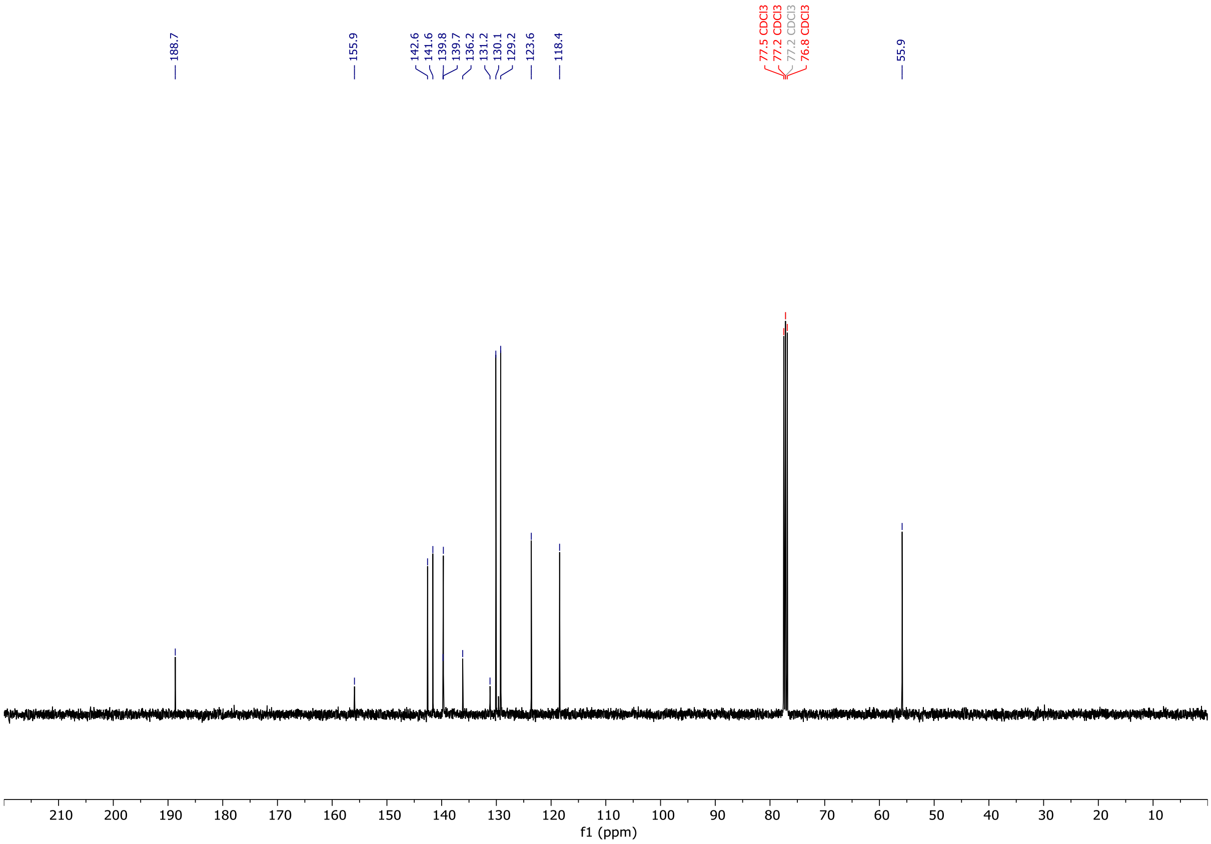

OX04540b

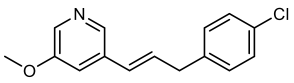

OX04540b

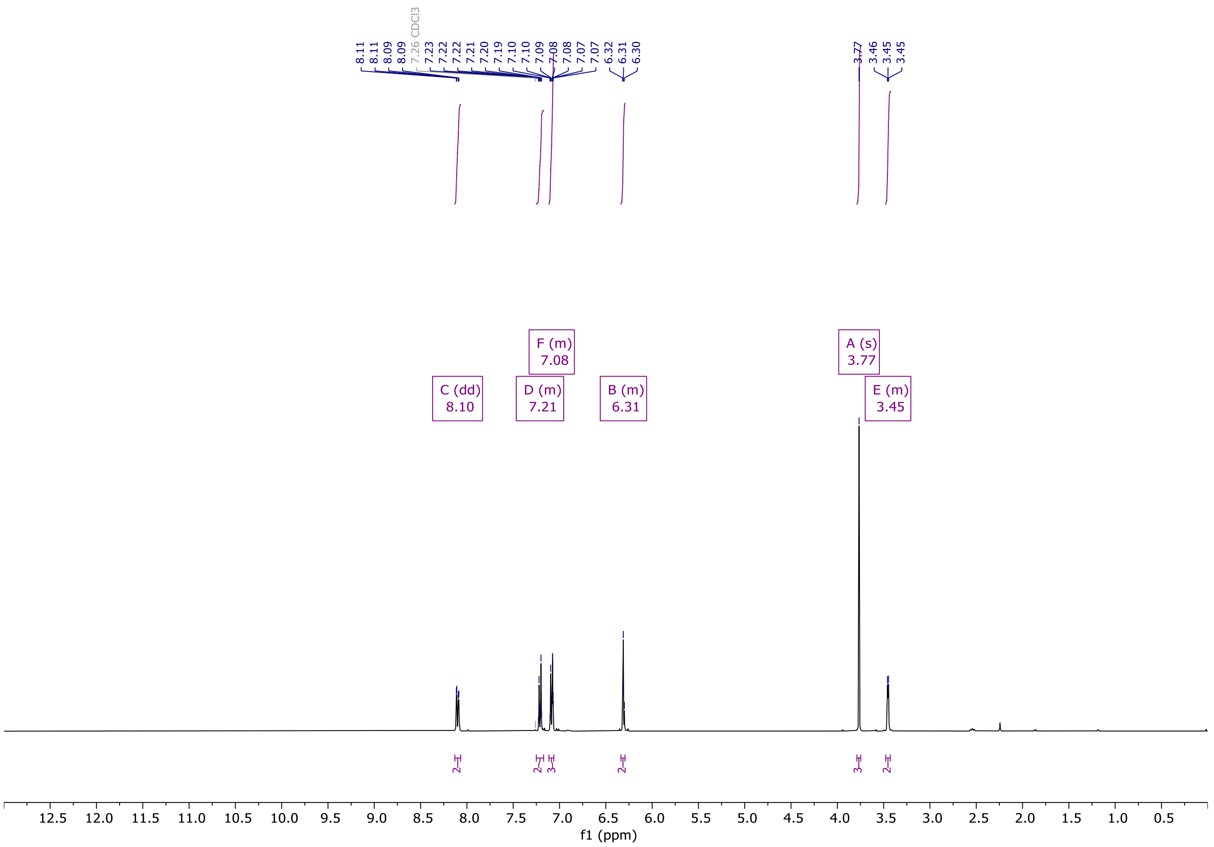

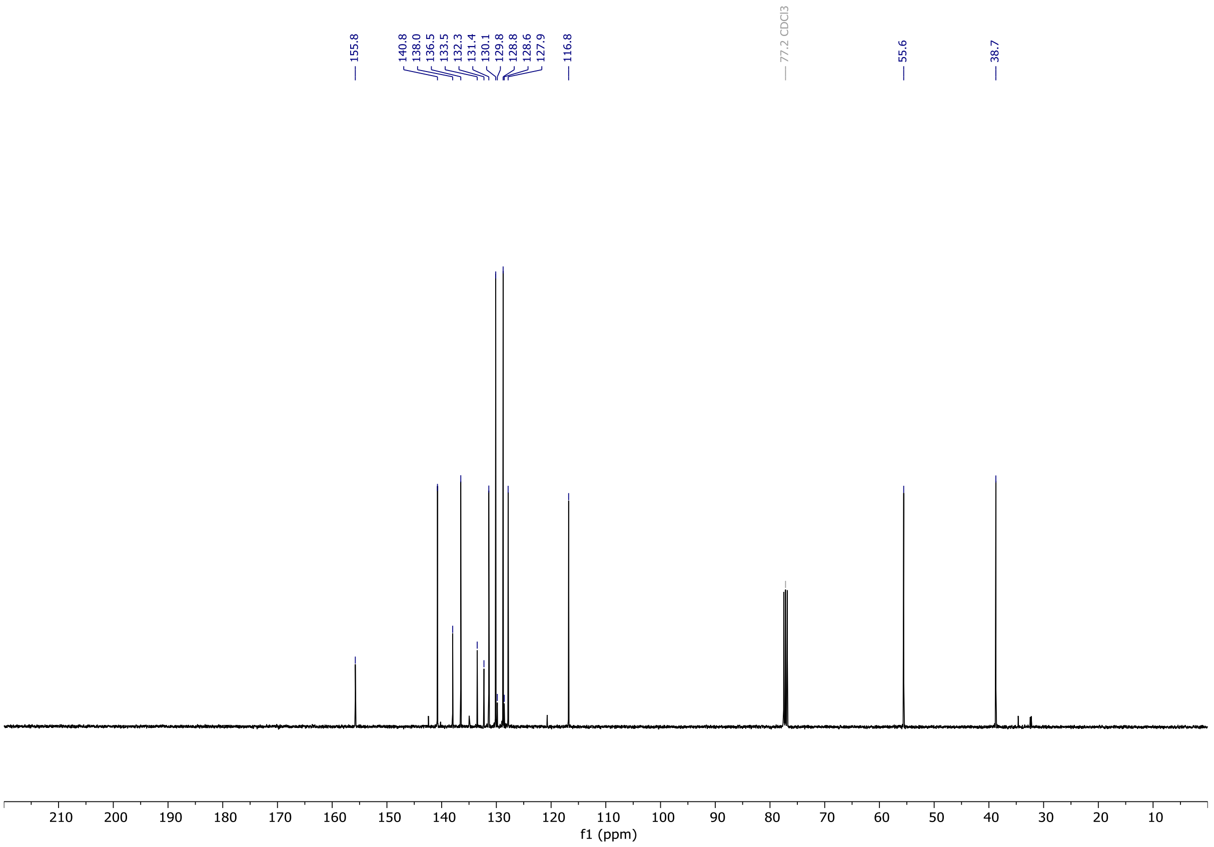

OX04540c

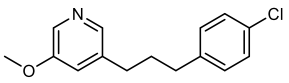

OX04540c

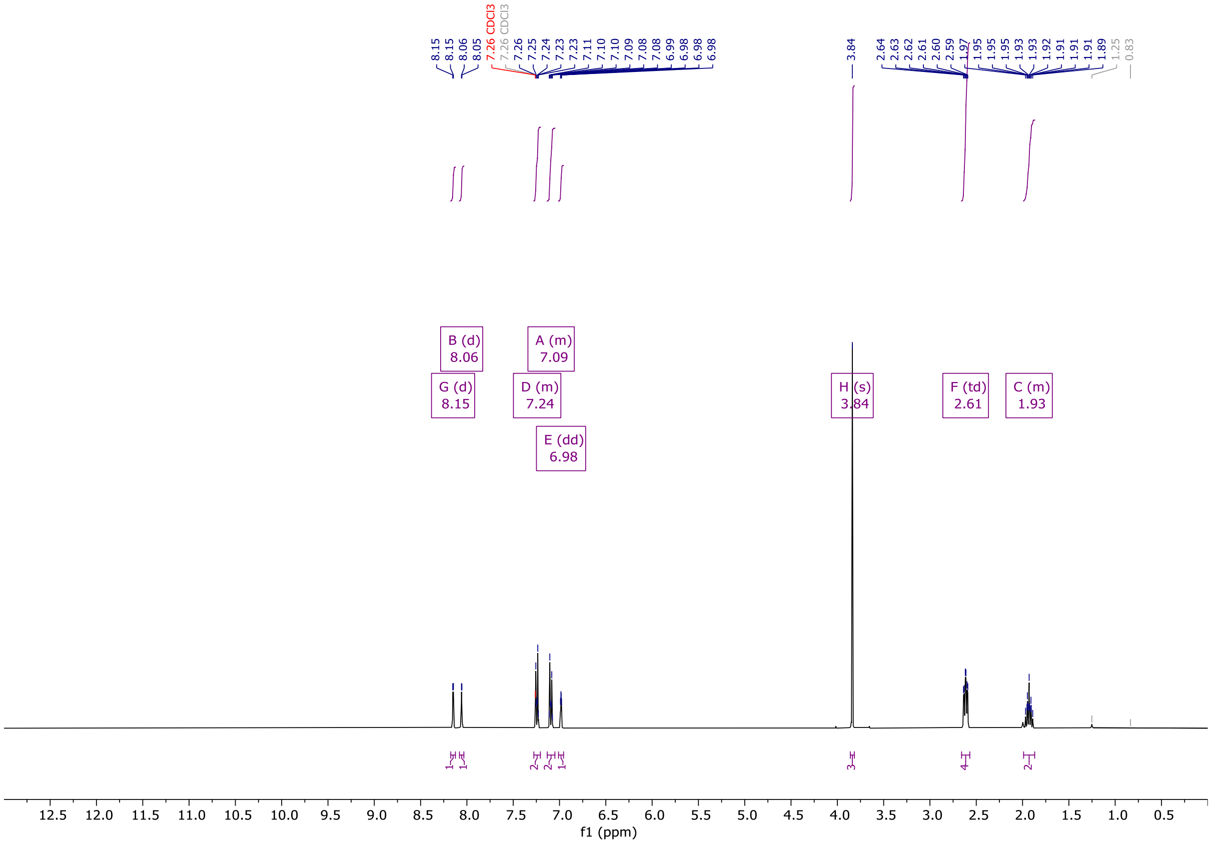

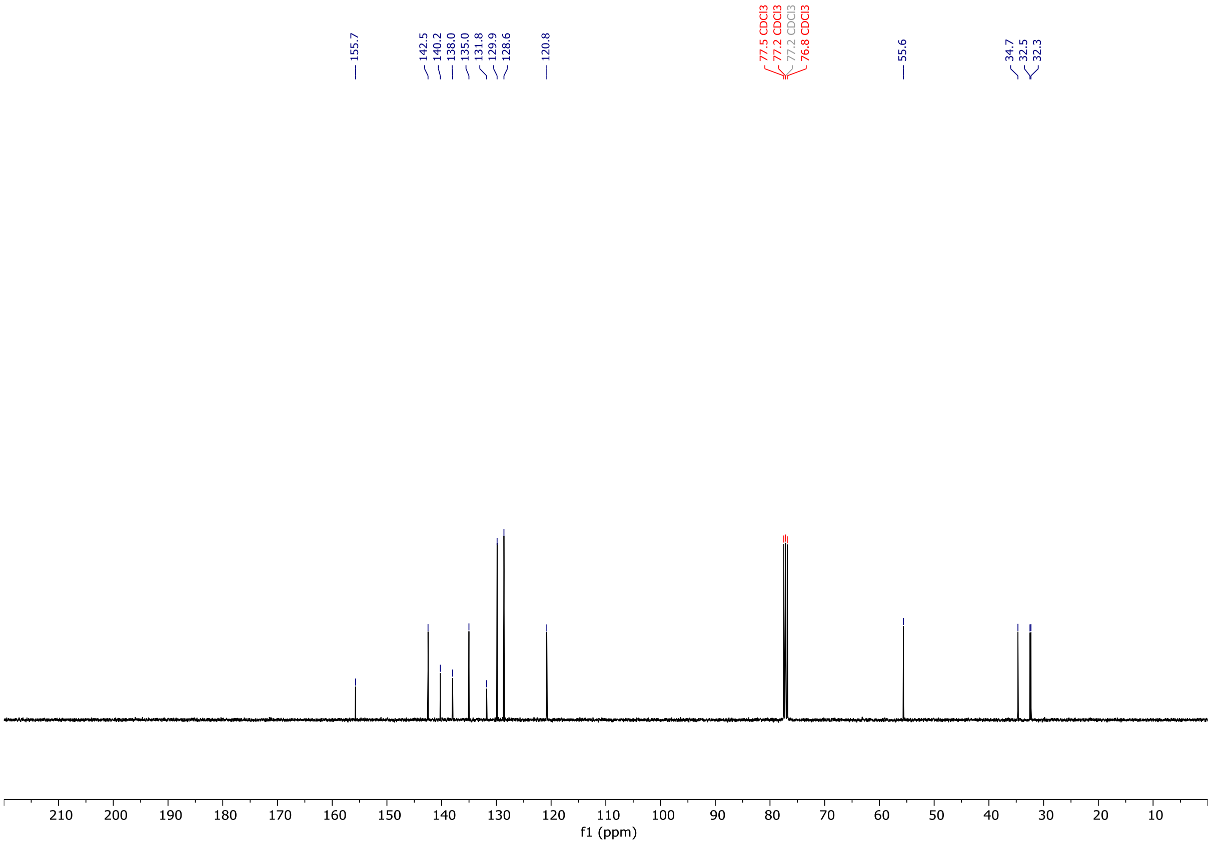

OX04540d

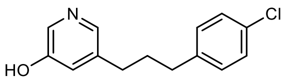

OX04540d

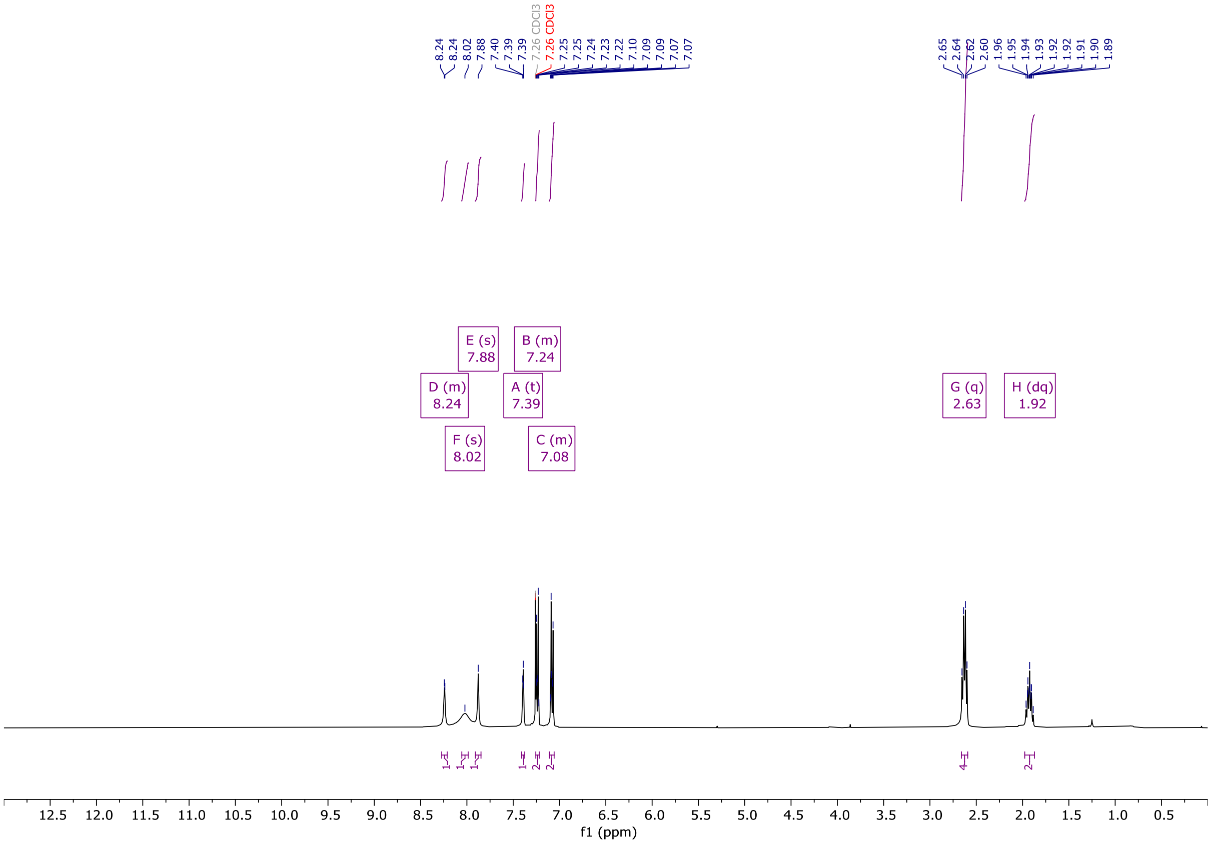

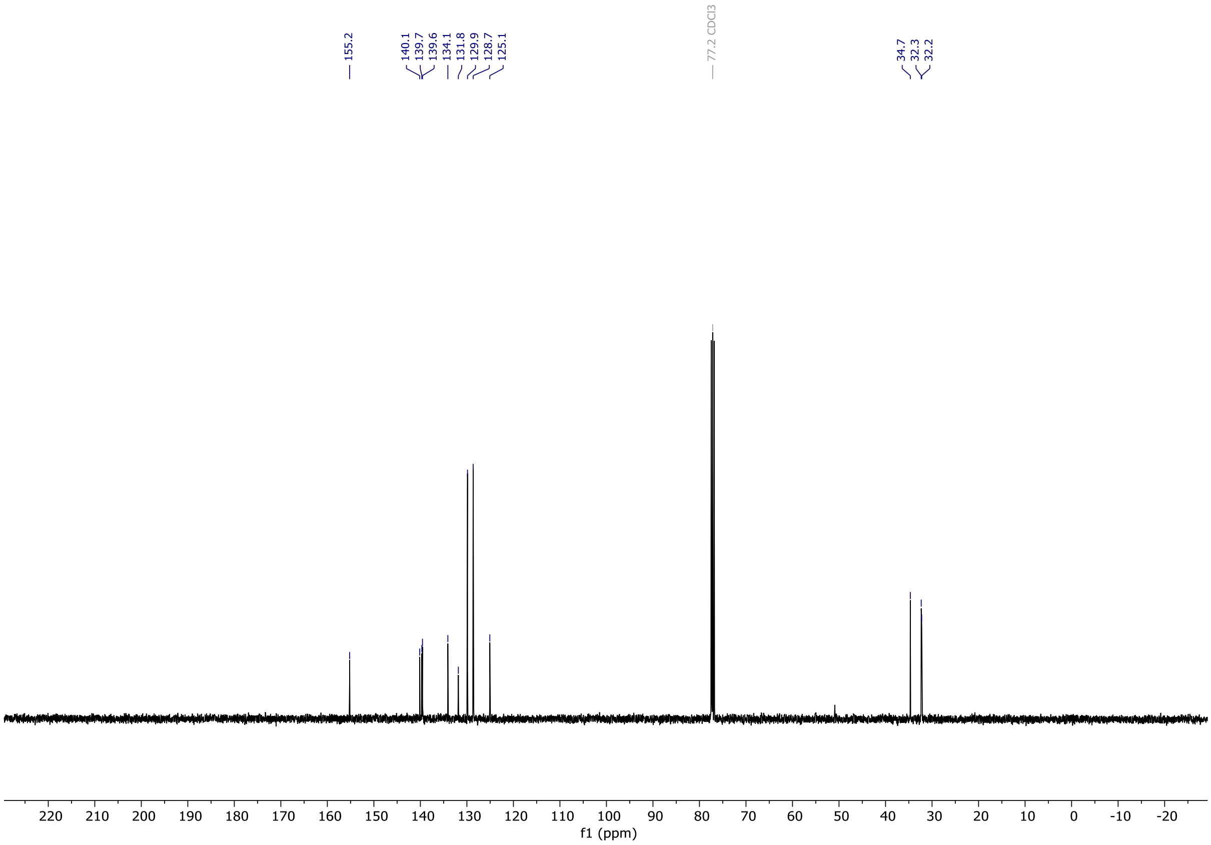

OX04540

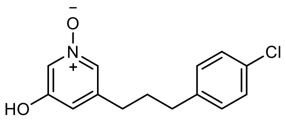

OX04540

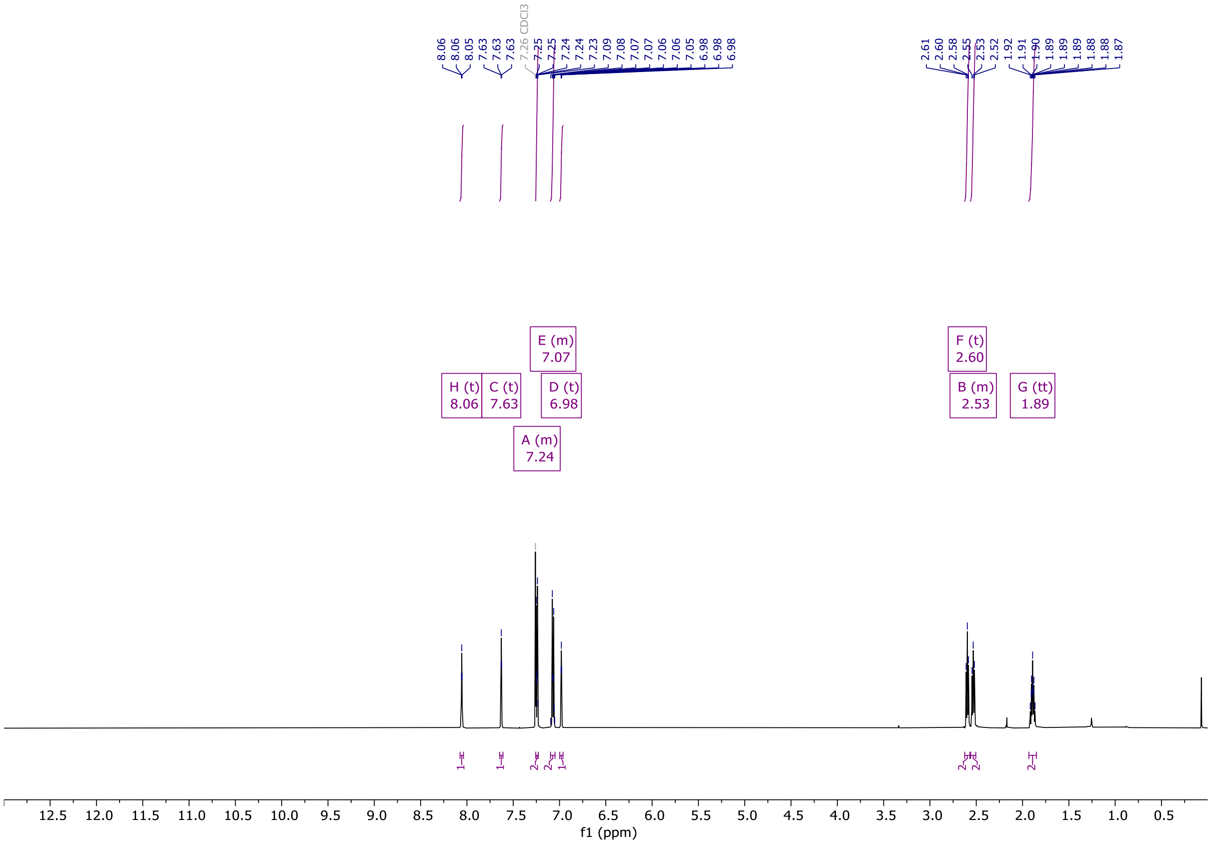

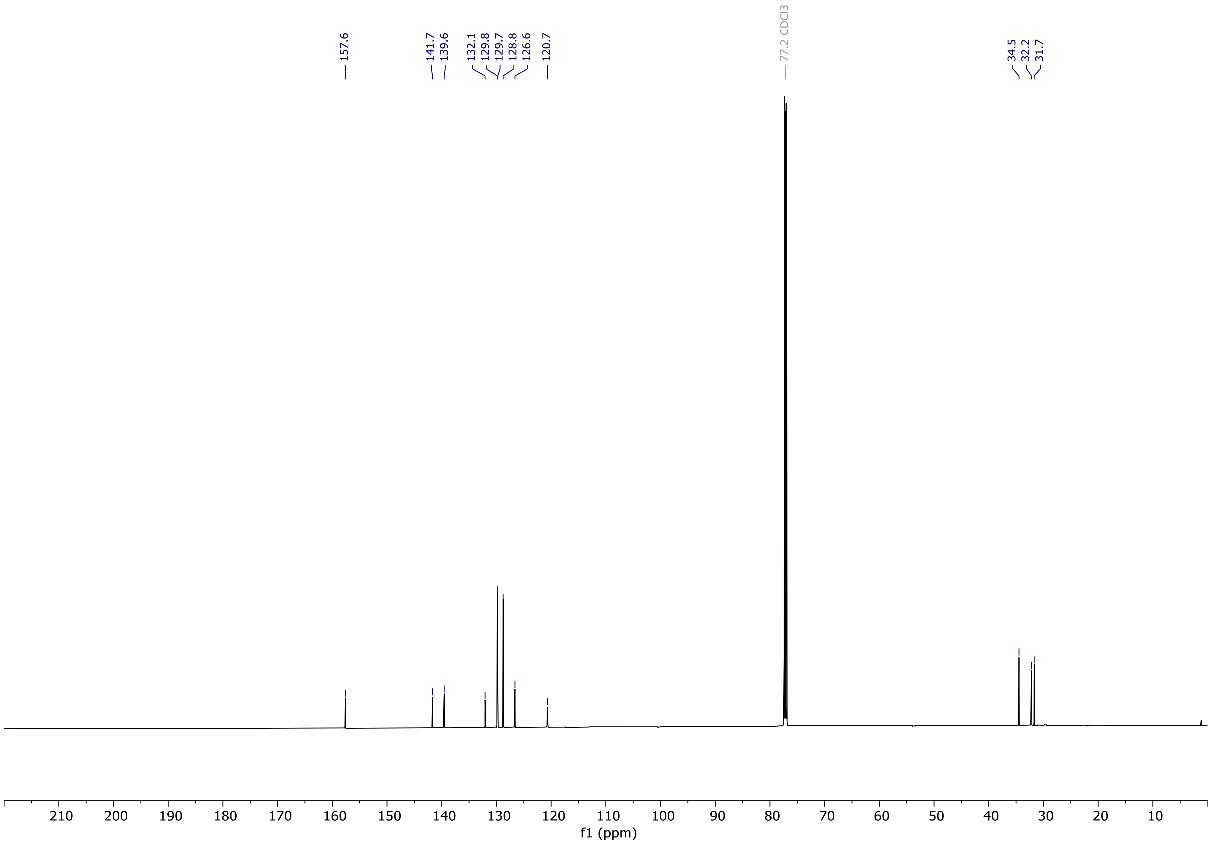

OX04954a

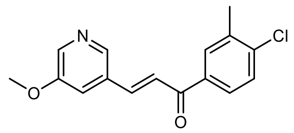

OX04954a

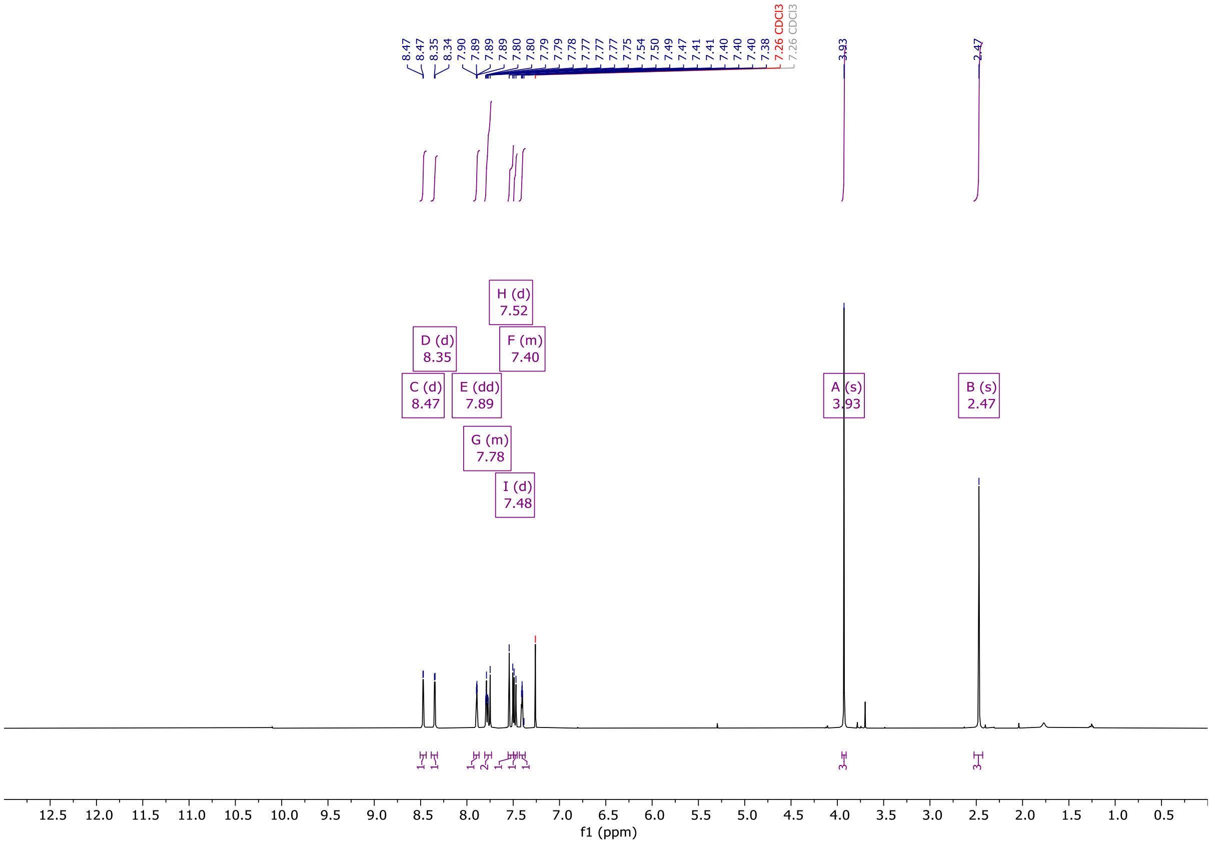

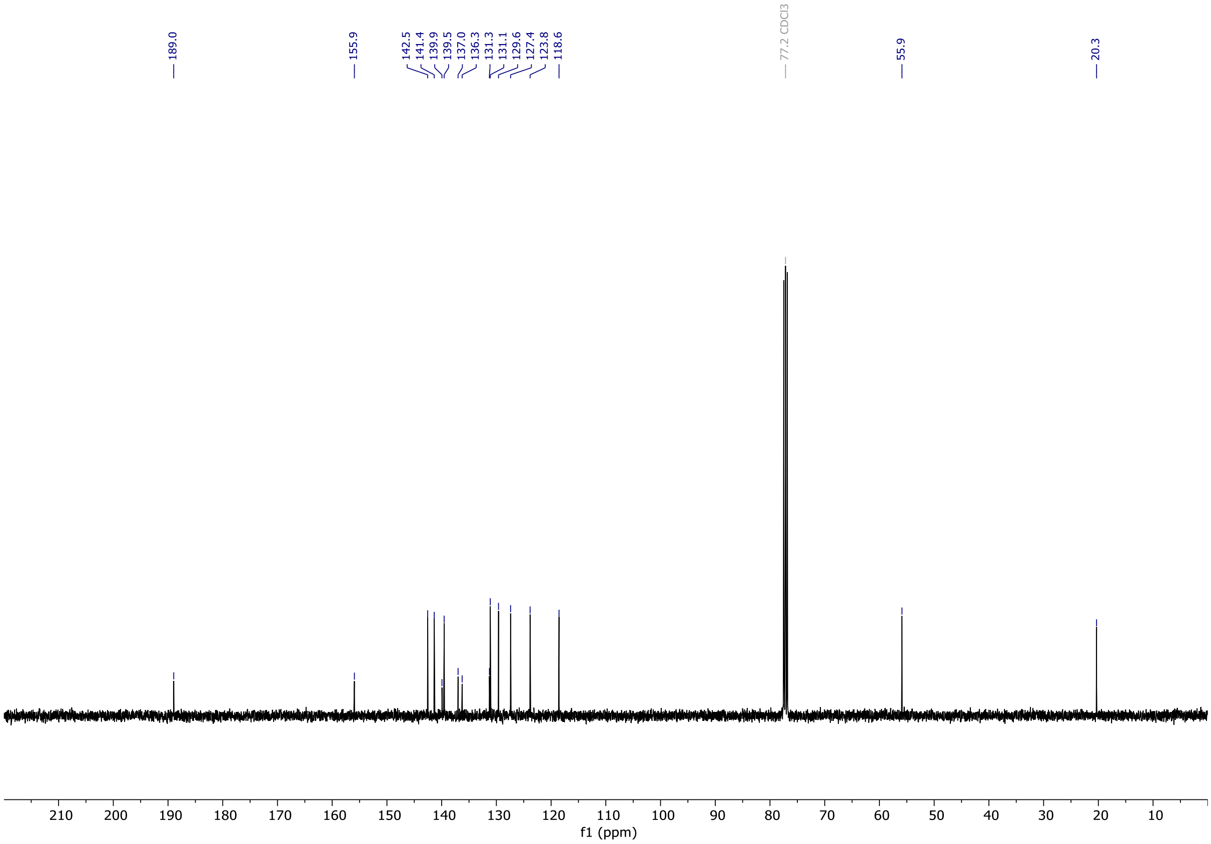

OX04954b

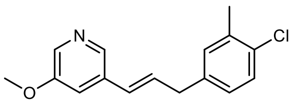

OX04954b

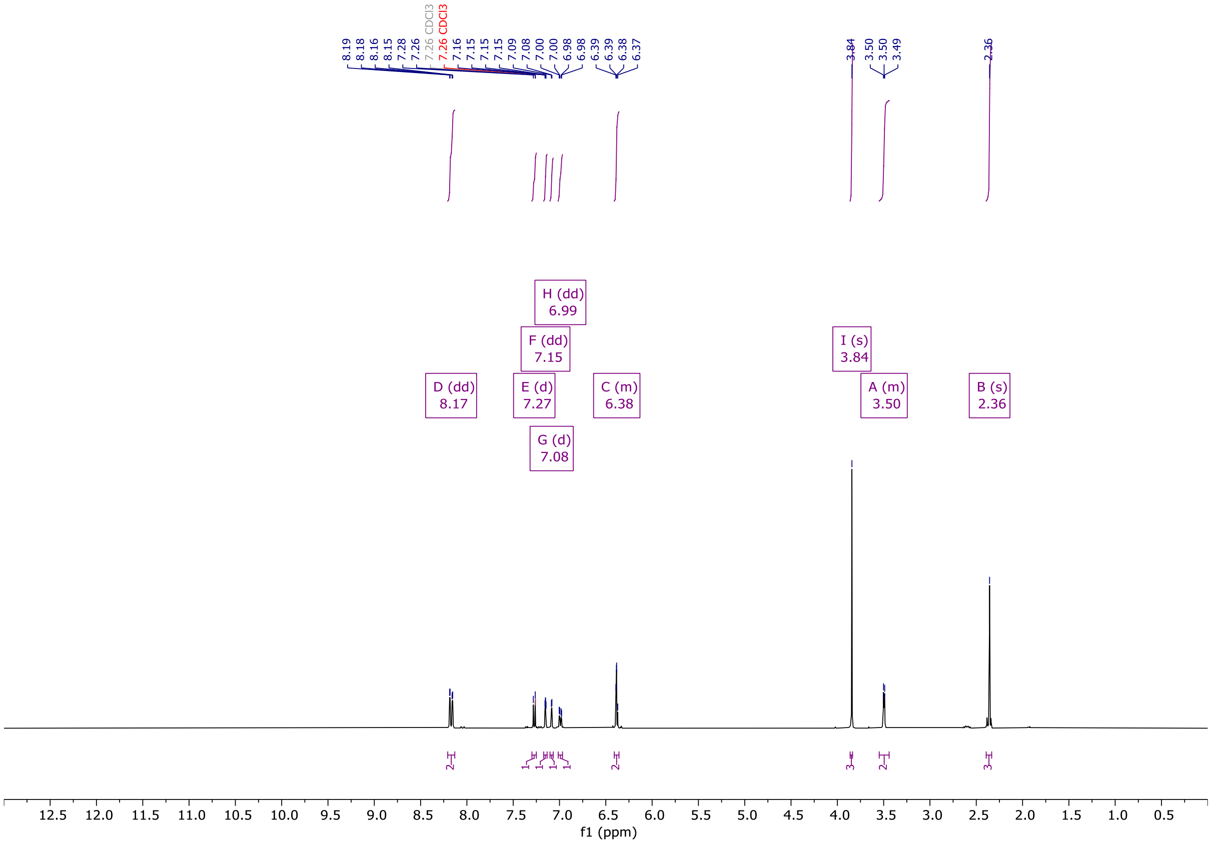

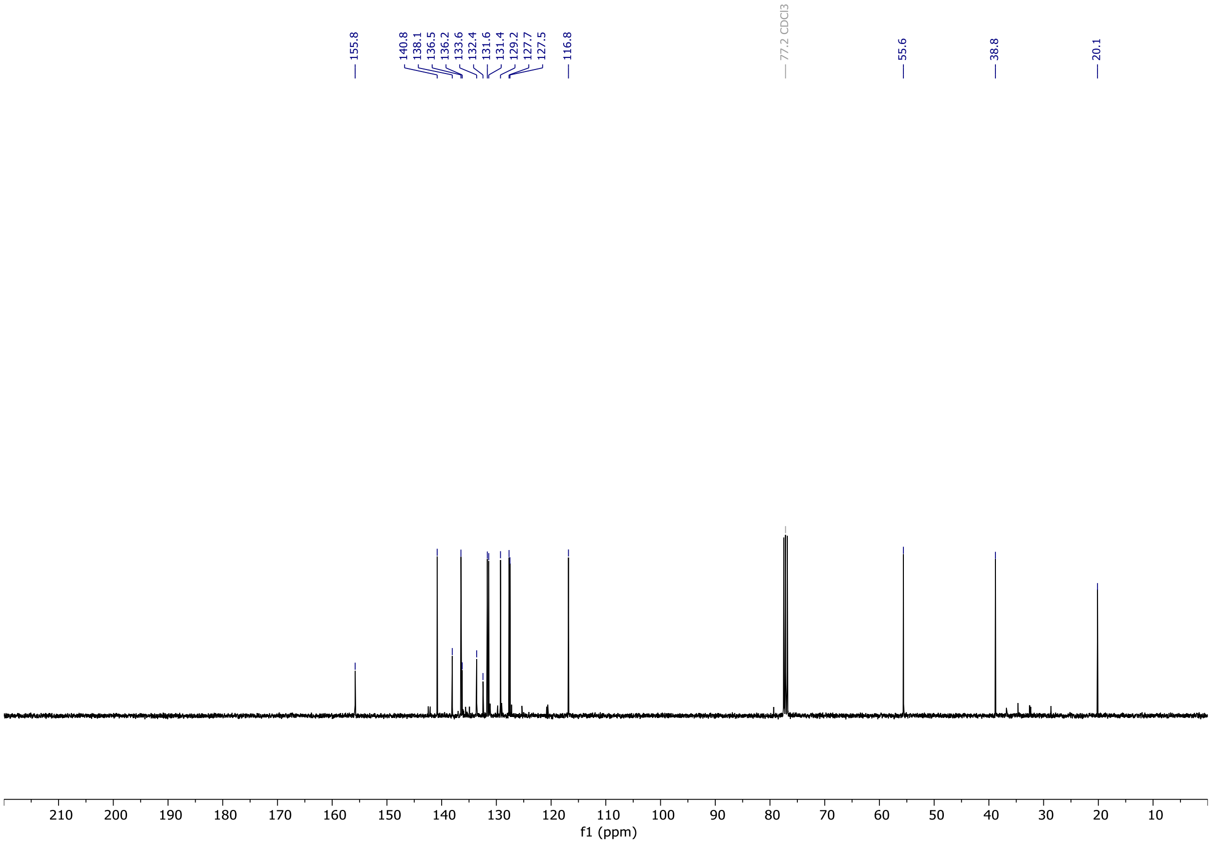

OX04954c

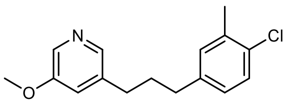

OX04954c

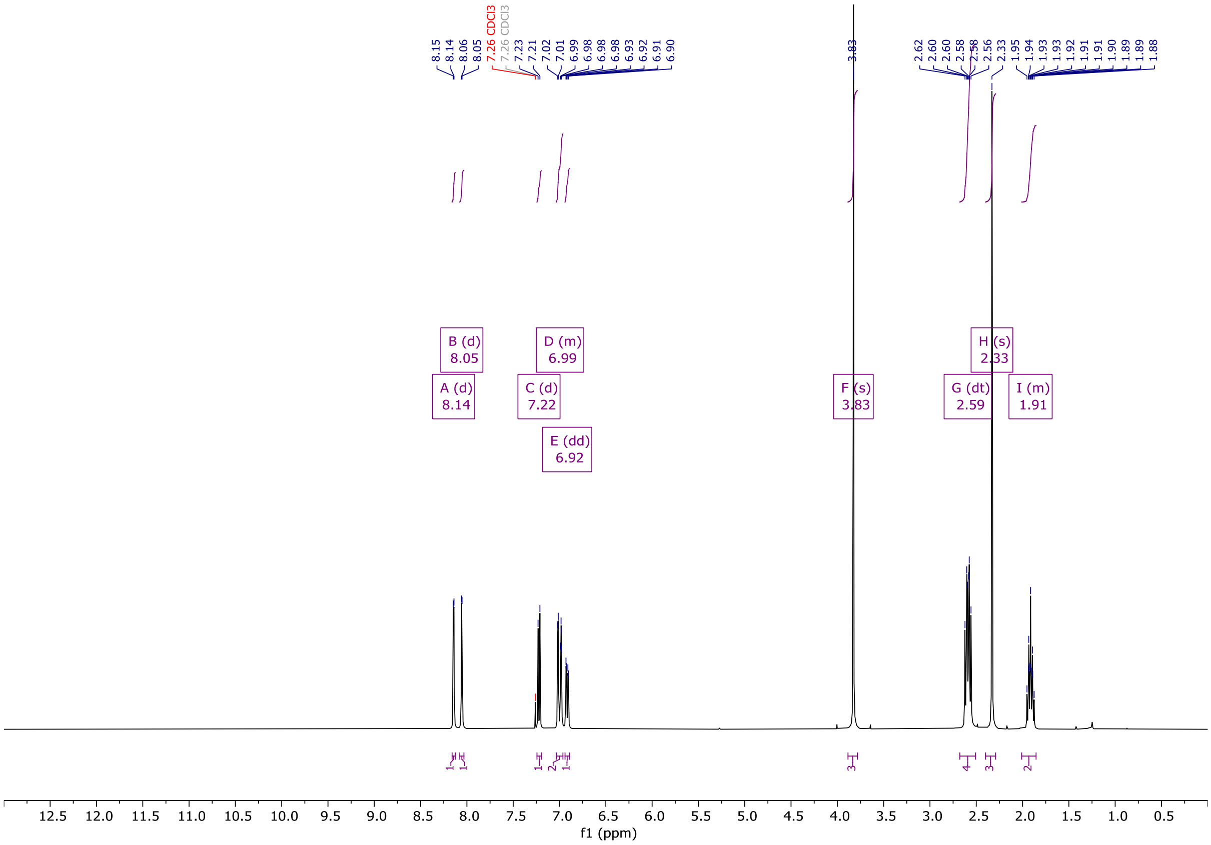

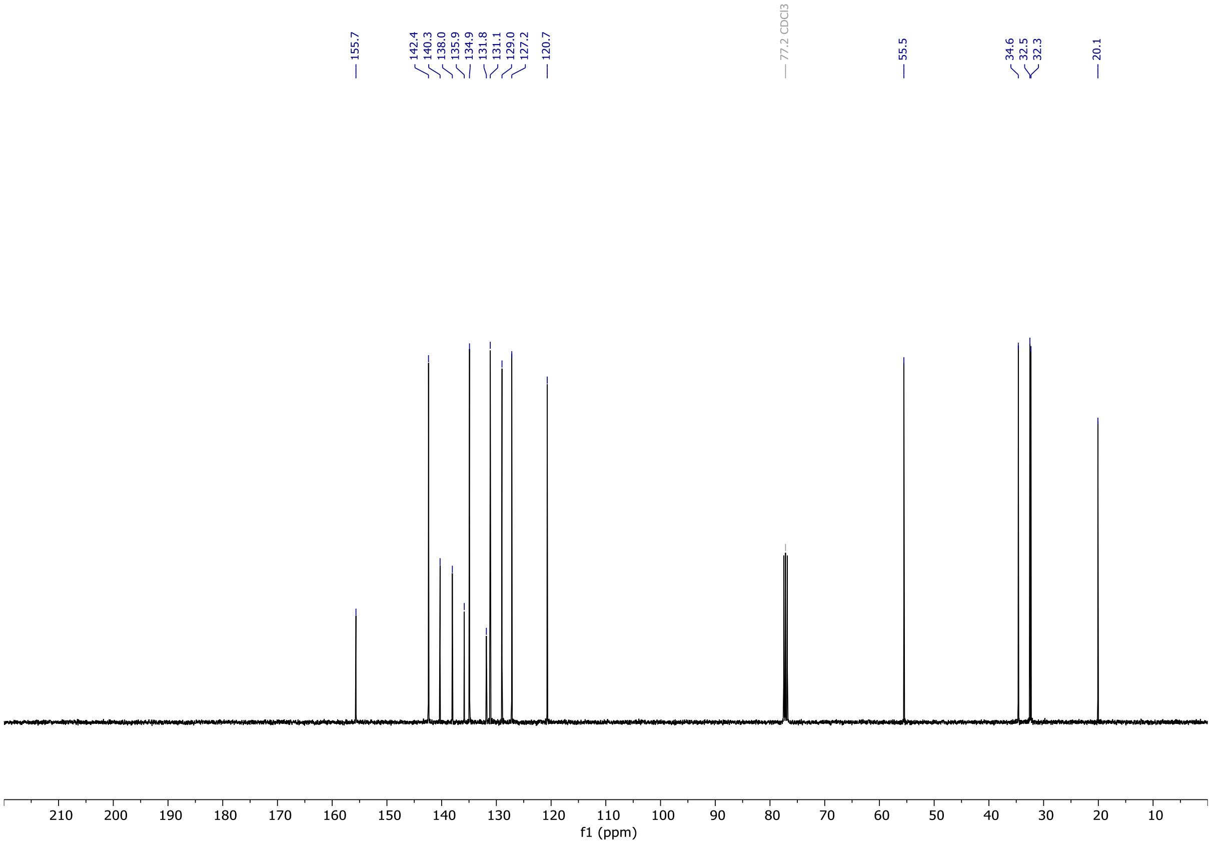

OX04954d

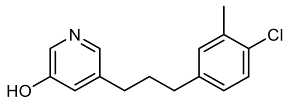

OX04954d

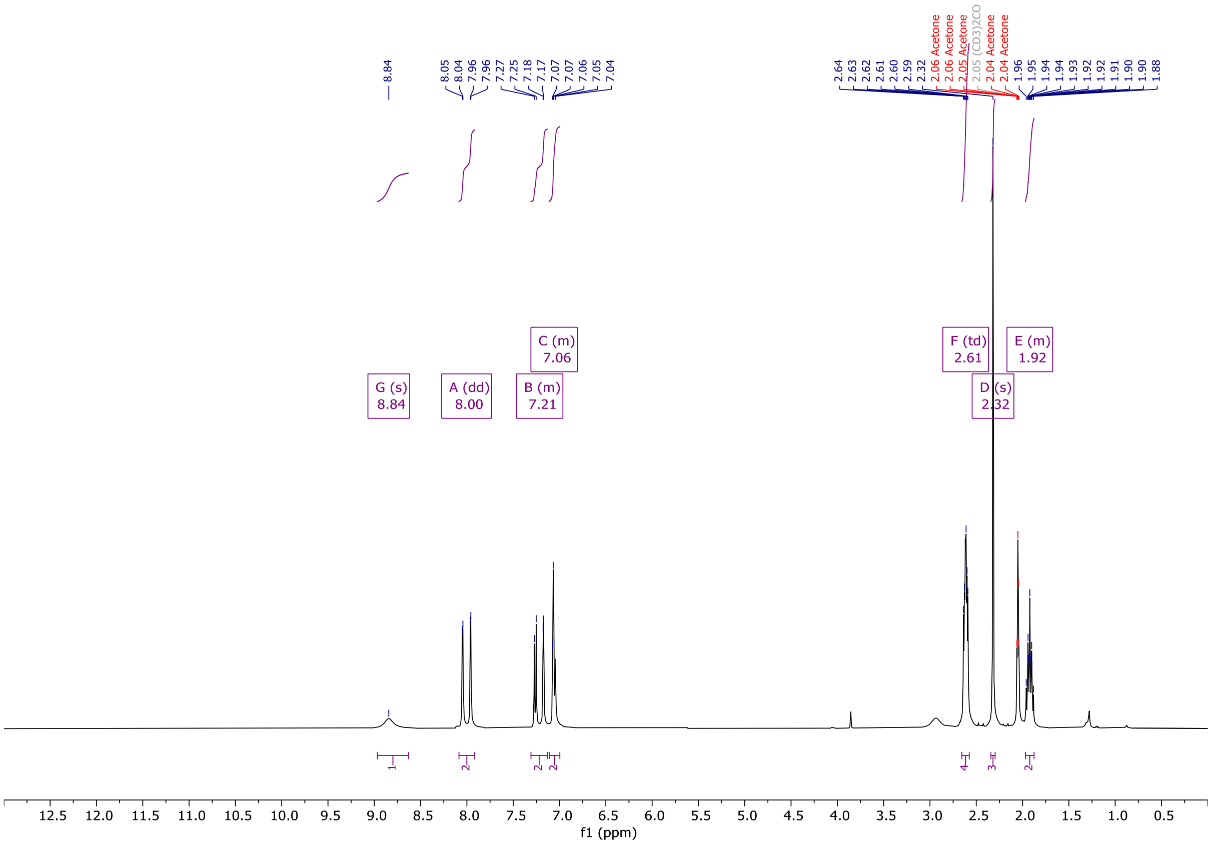

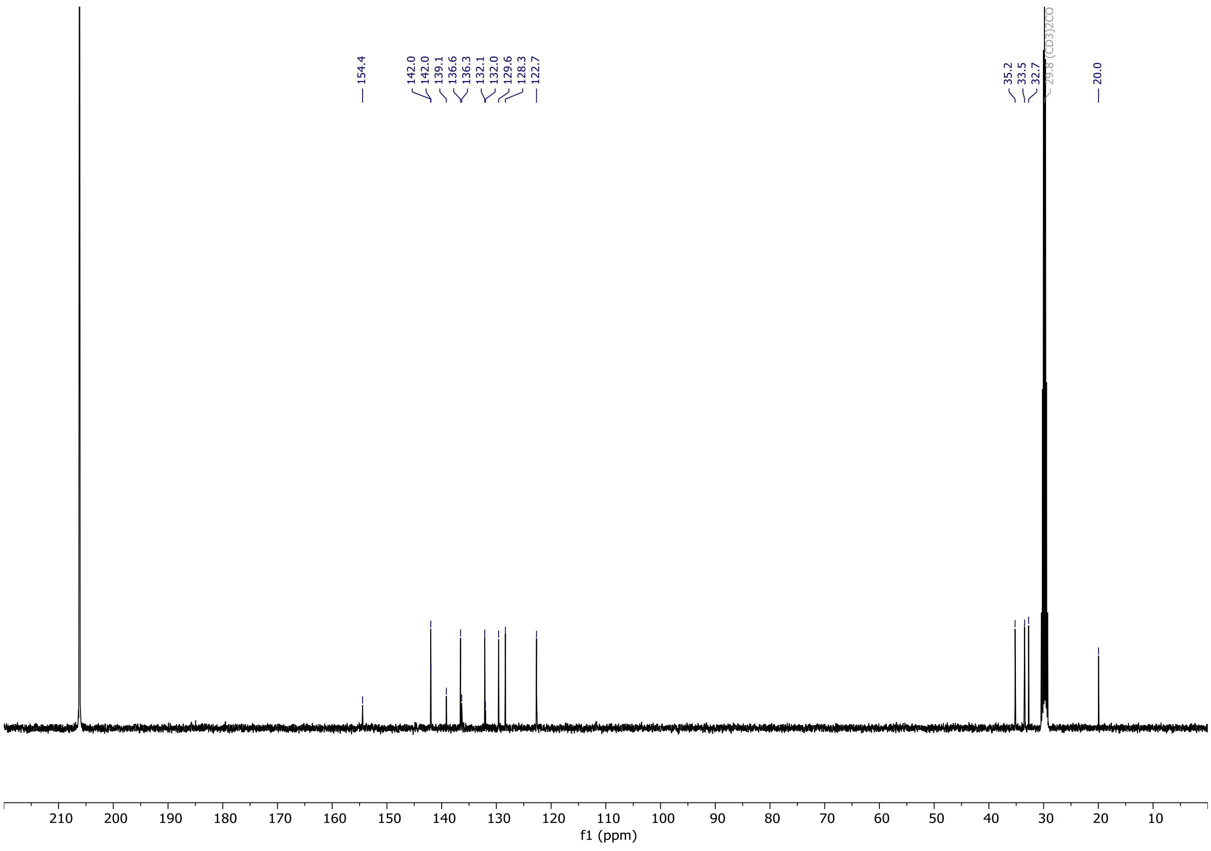

OX04954

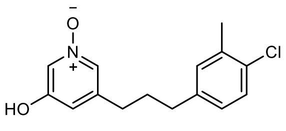

OX04954

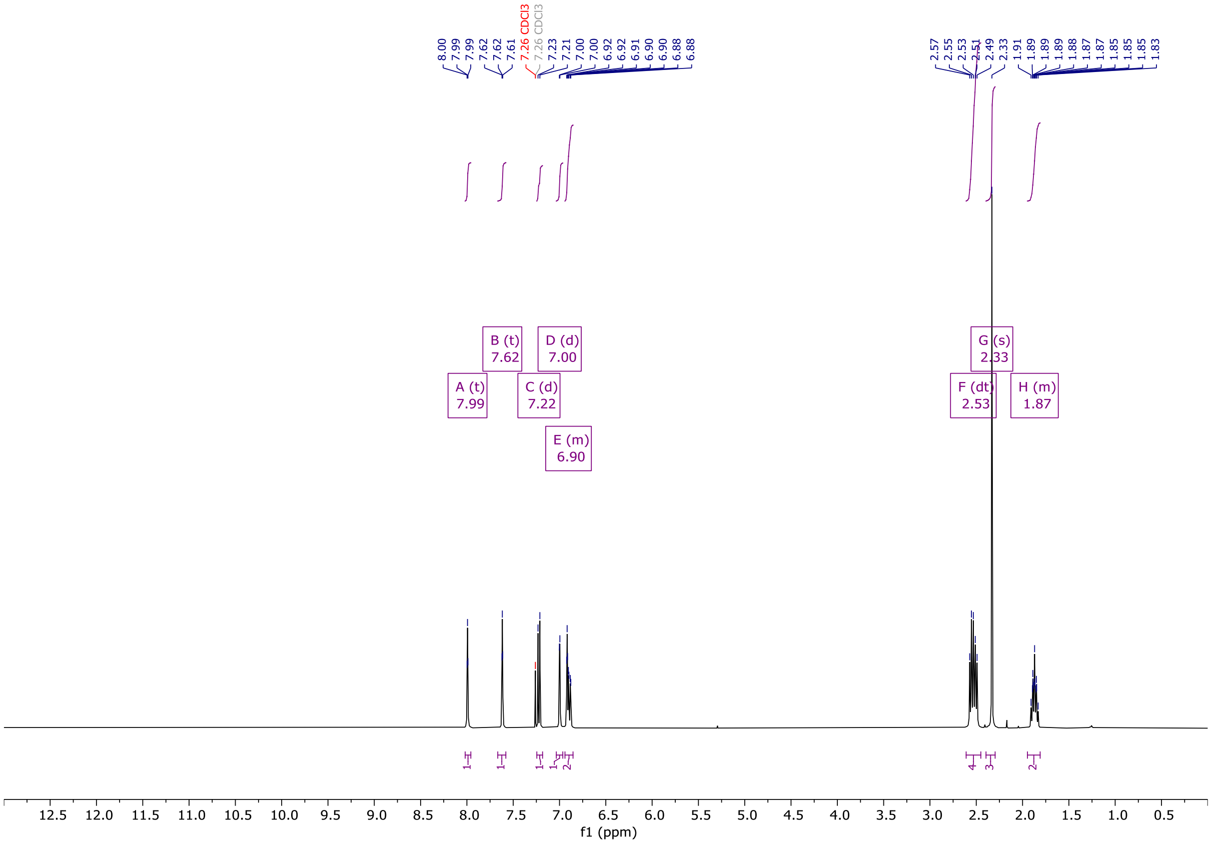

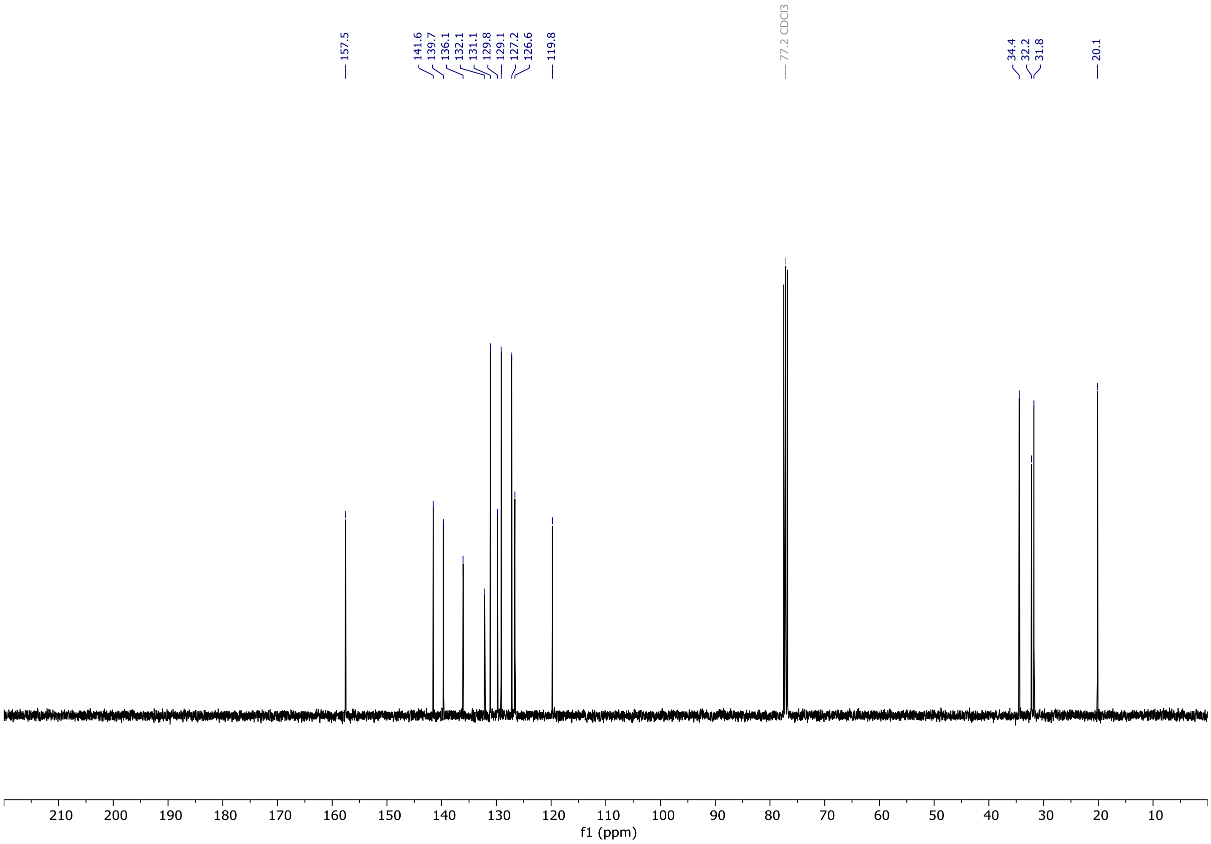

OX04956a

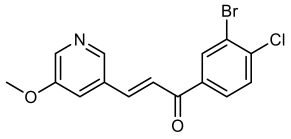

OX04956a

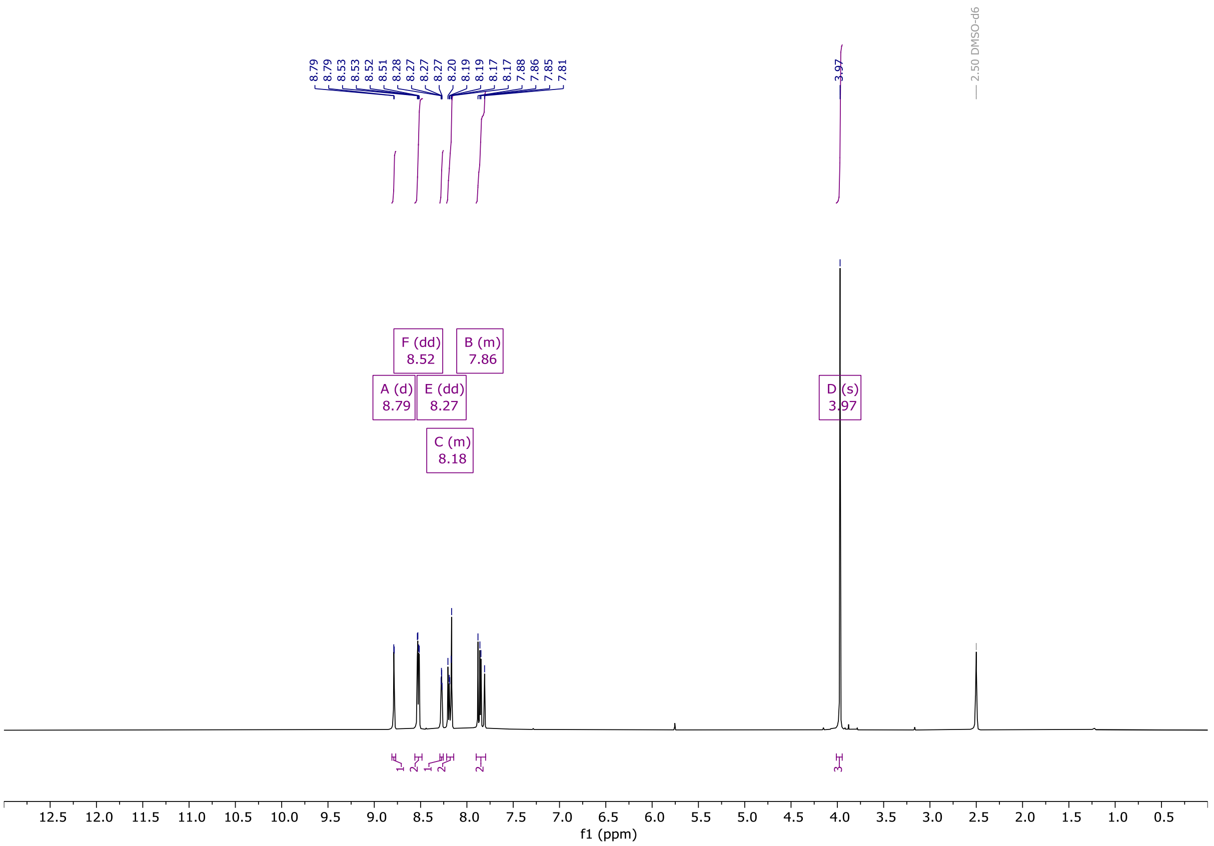

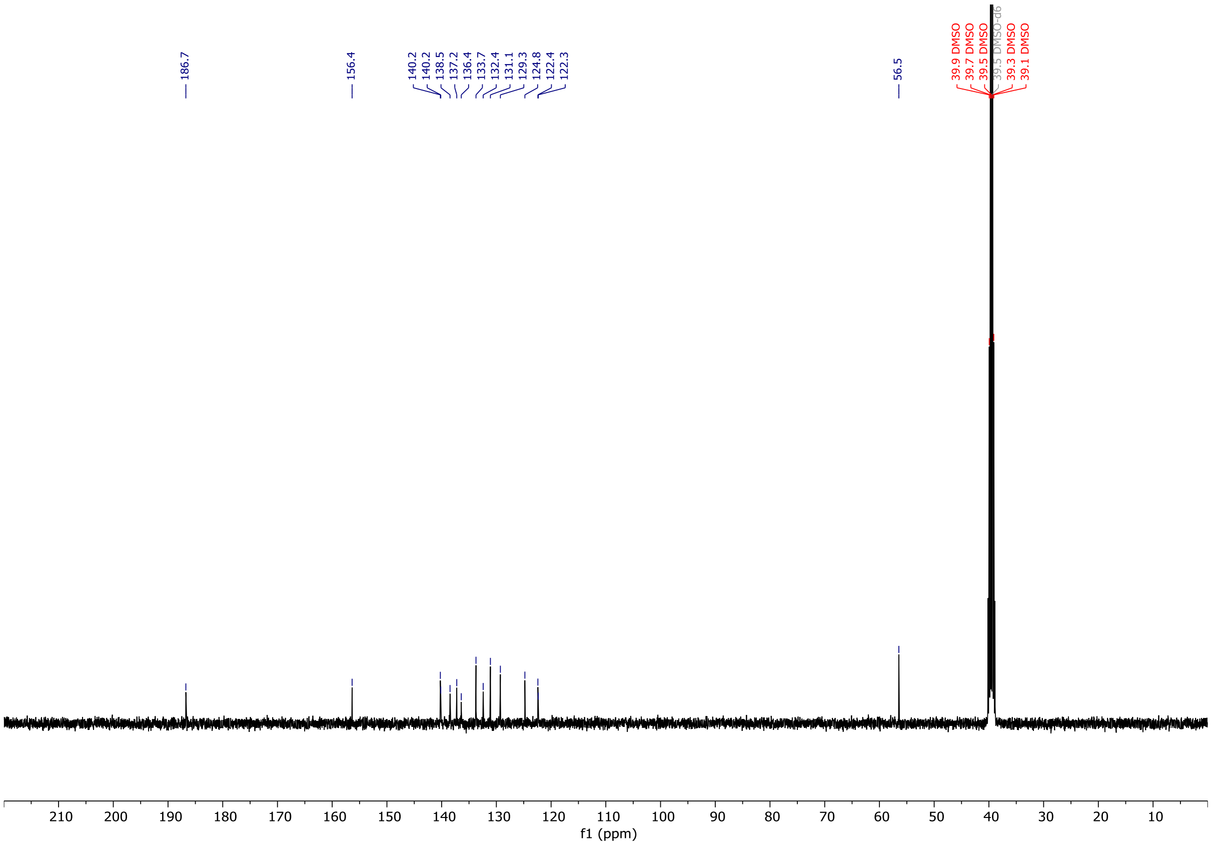

OX04956b

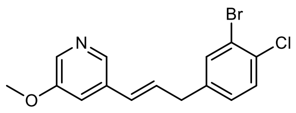

OX04956b

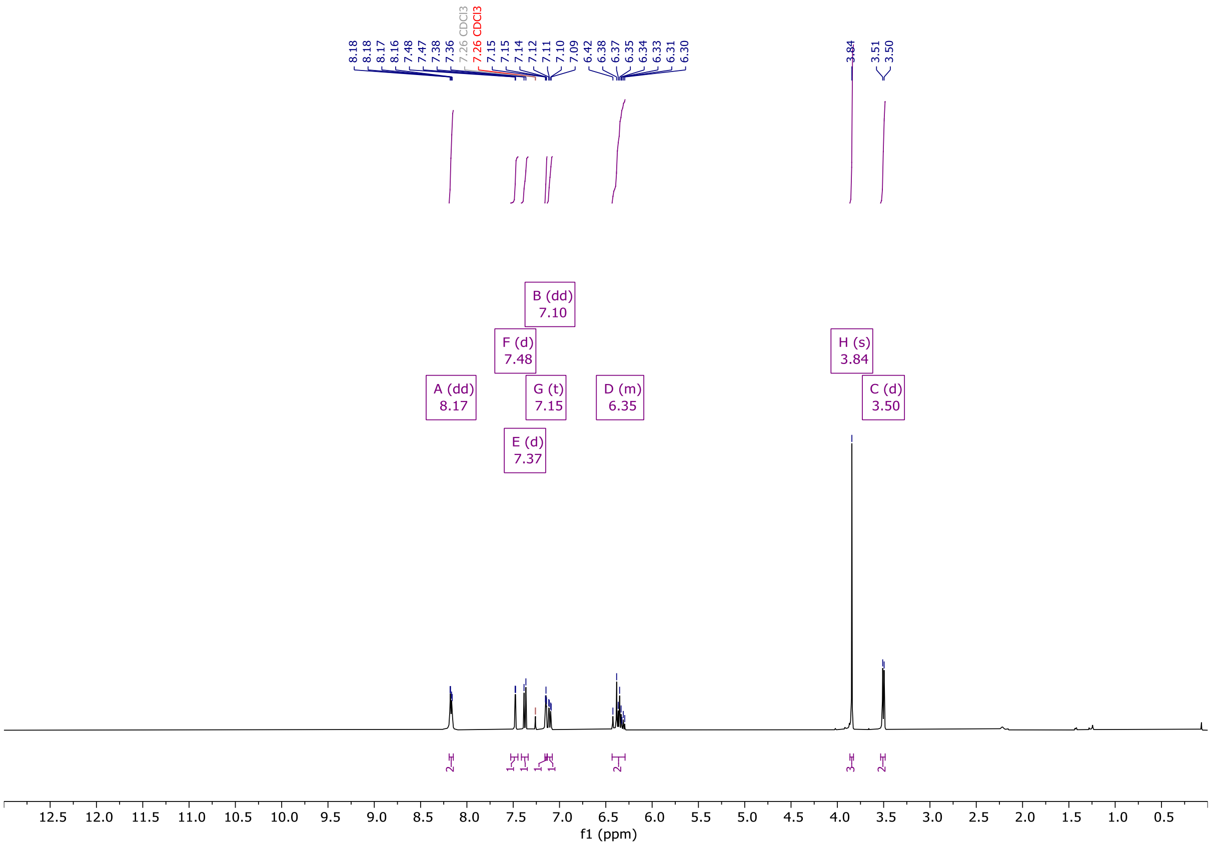

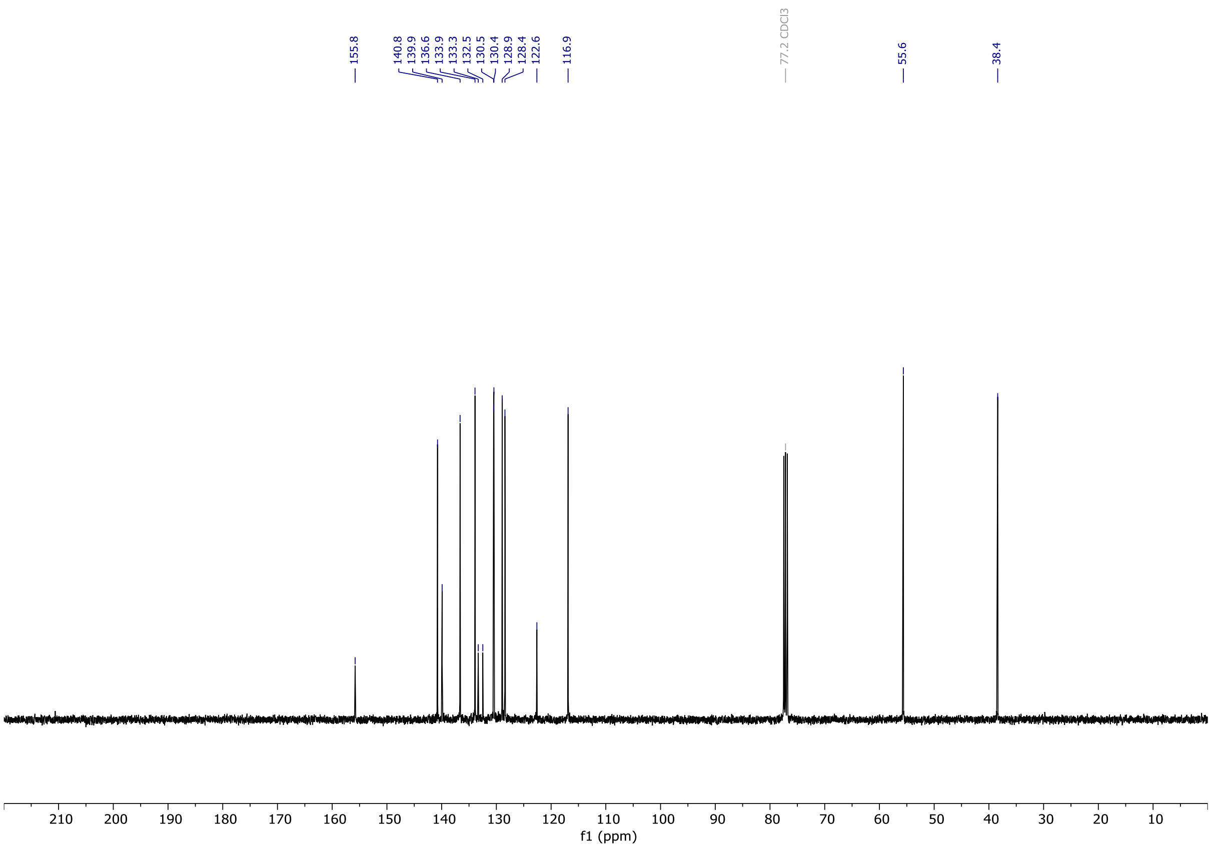

OX04956c

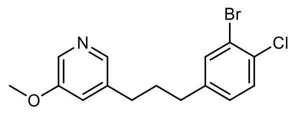

OX04956c

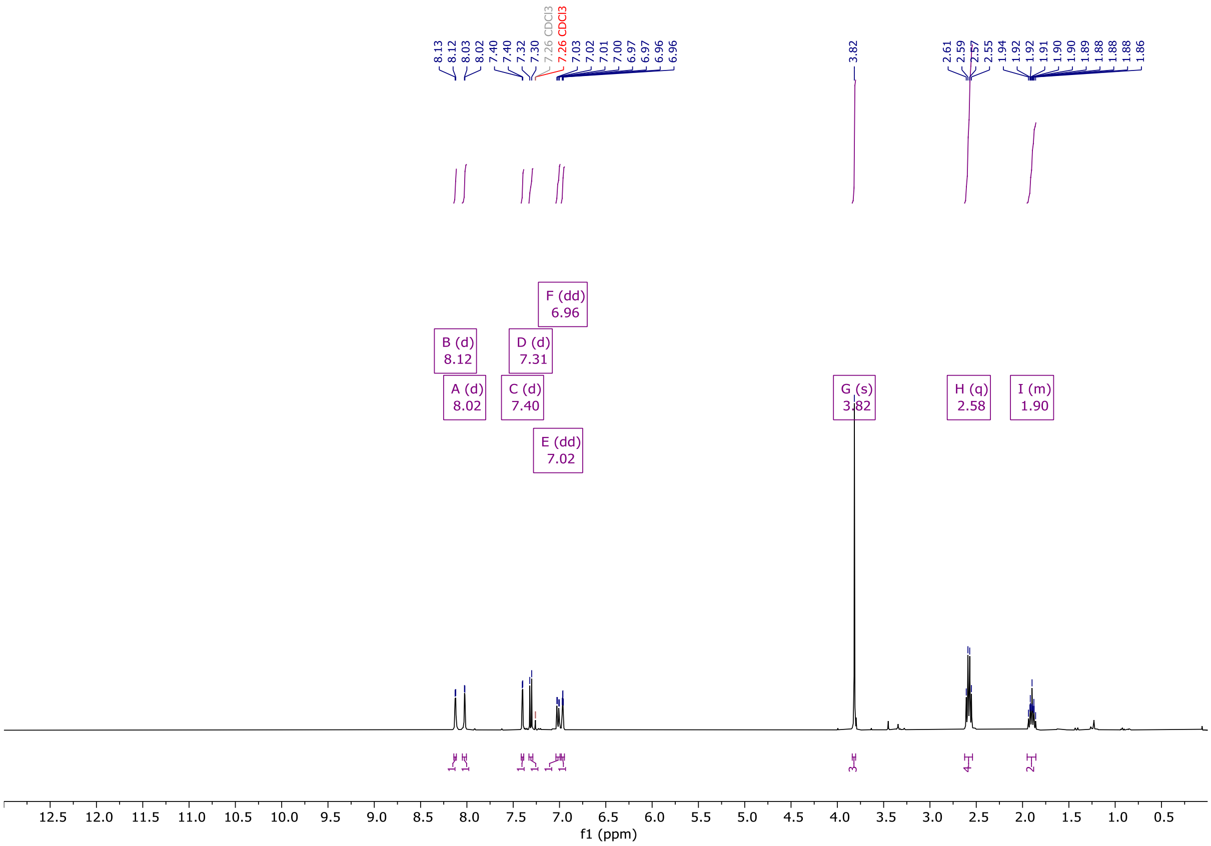

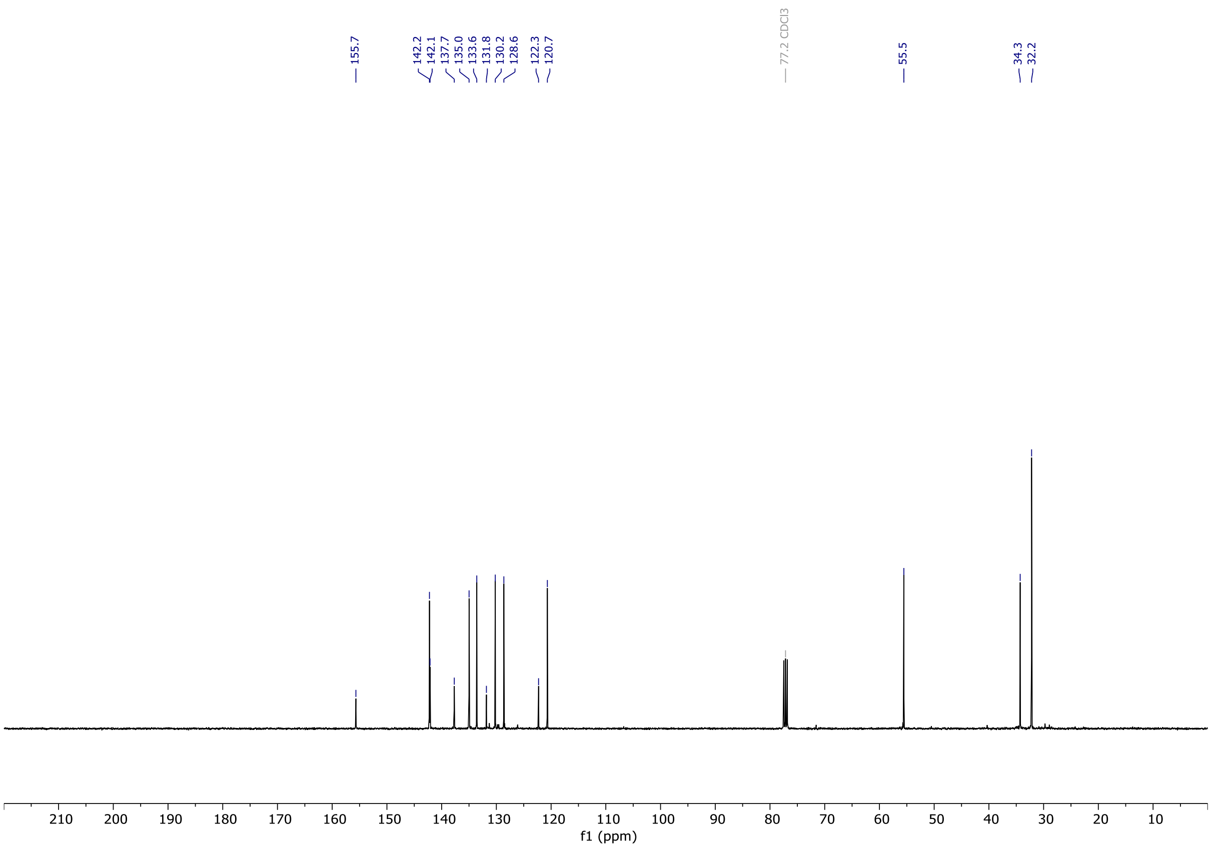

OX04956d

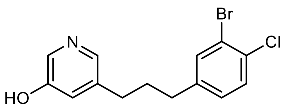

OX04956d

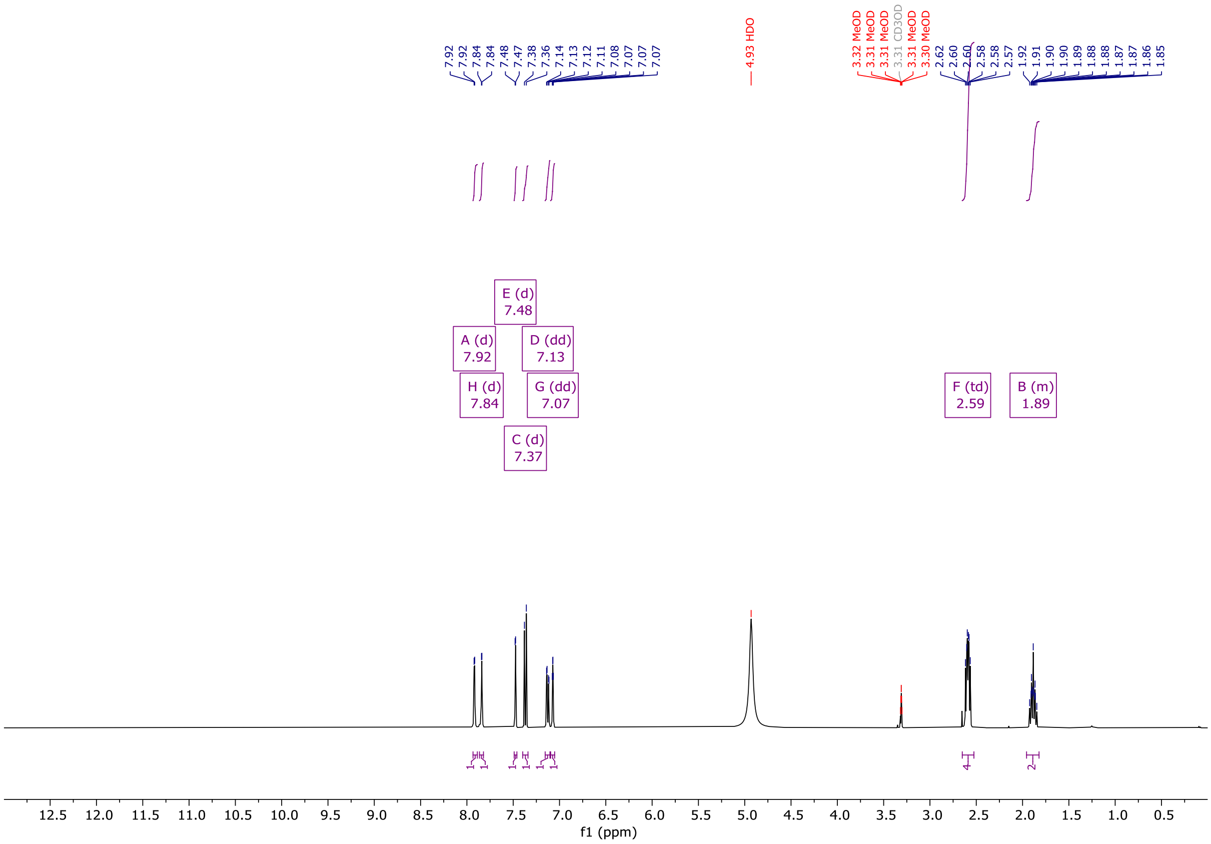

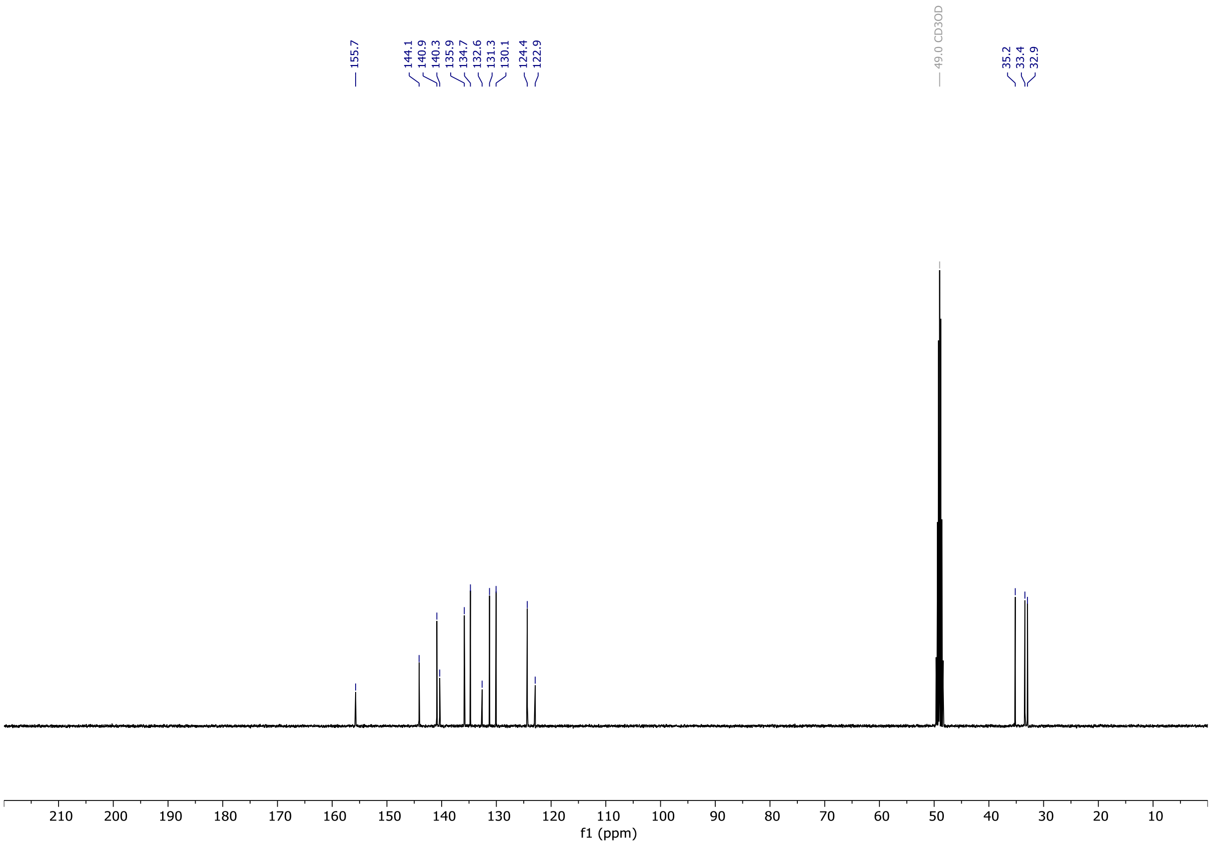

OX04956

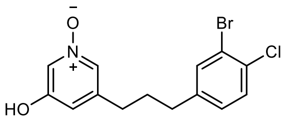

OX04956

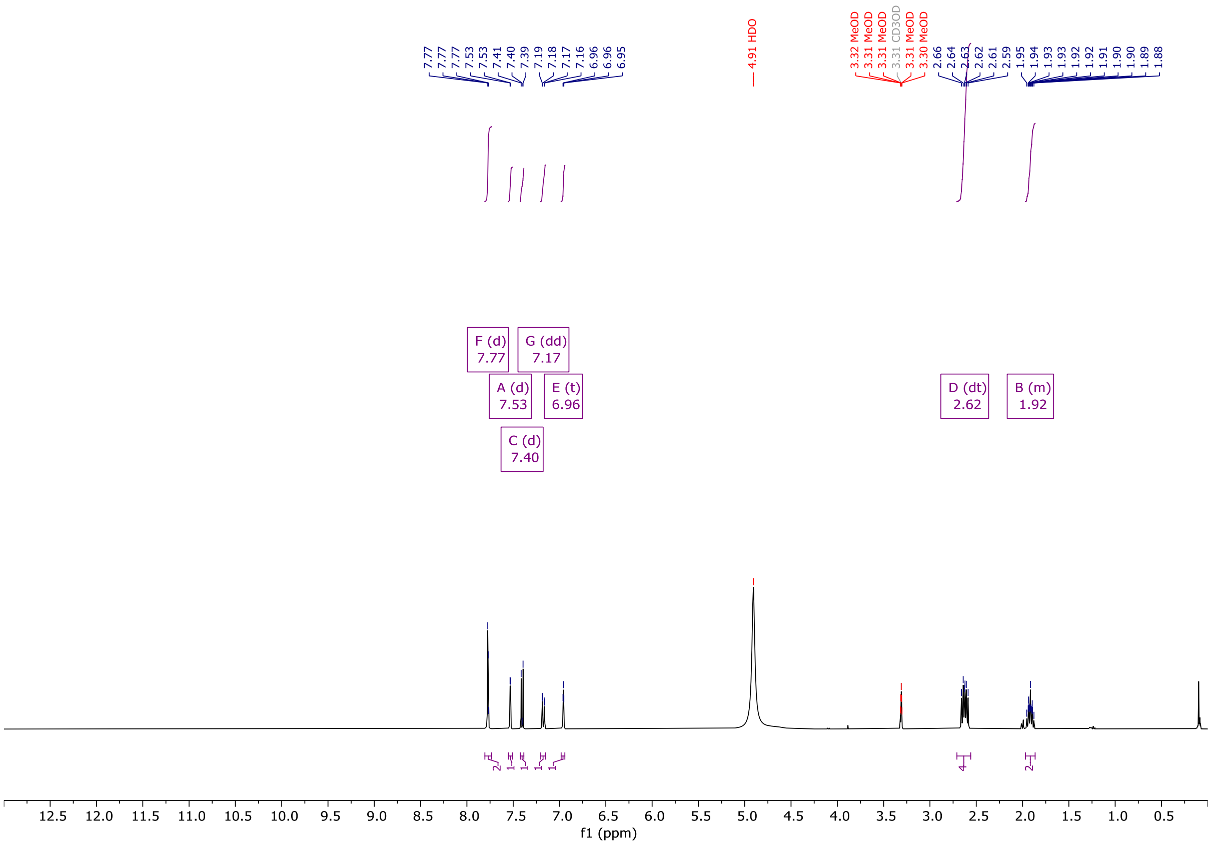

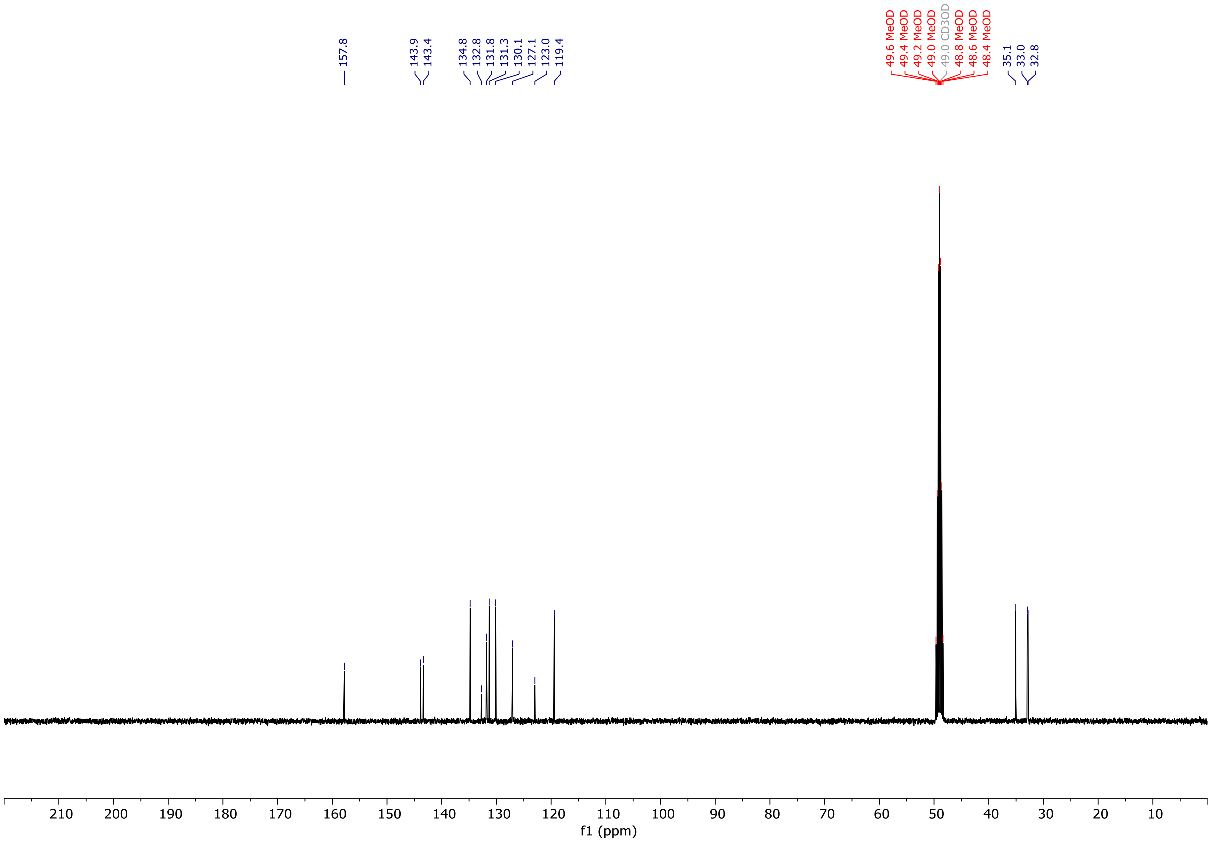

OX04955a

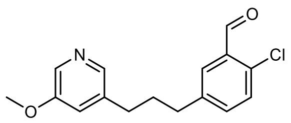

OX04955a

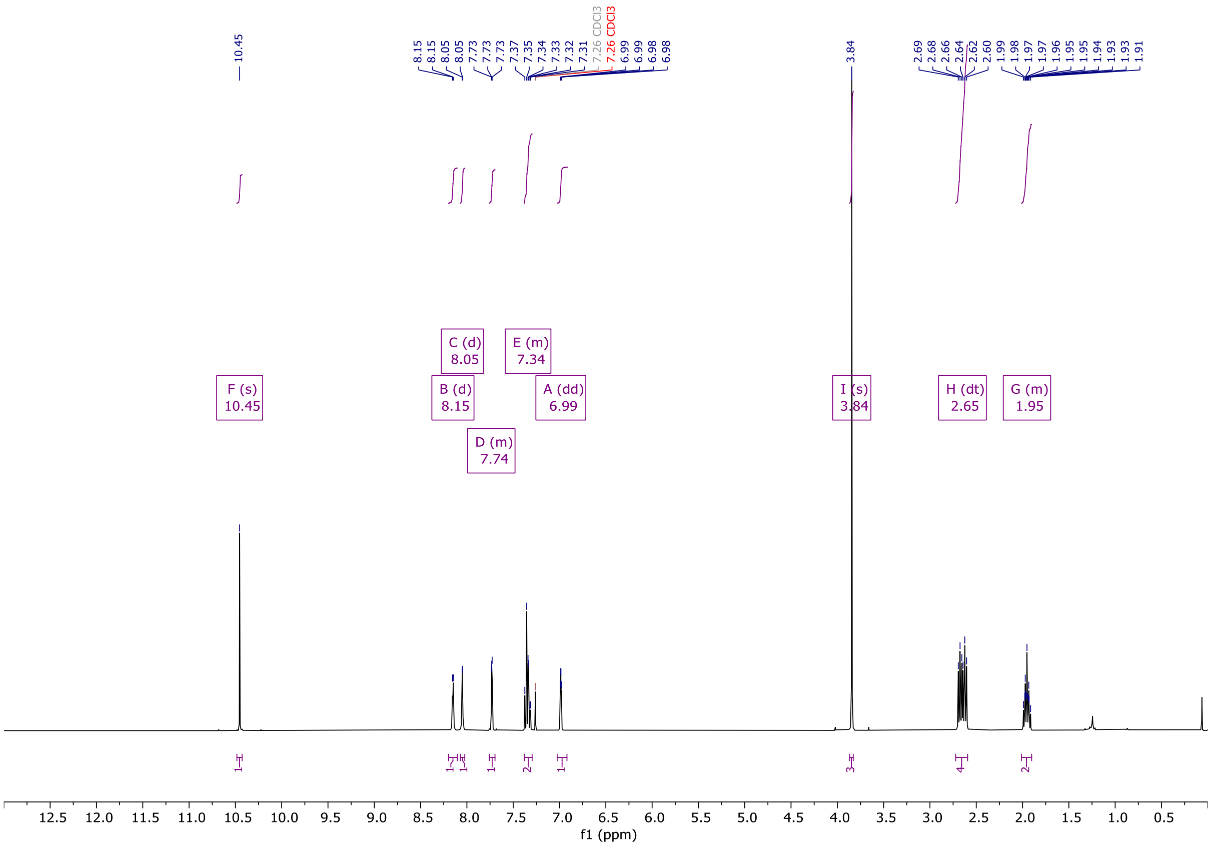

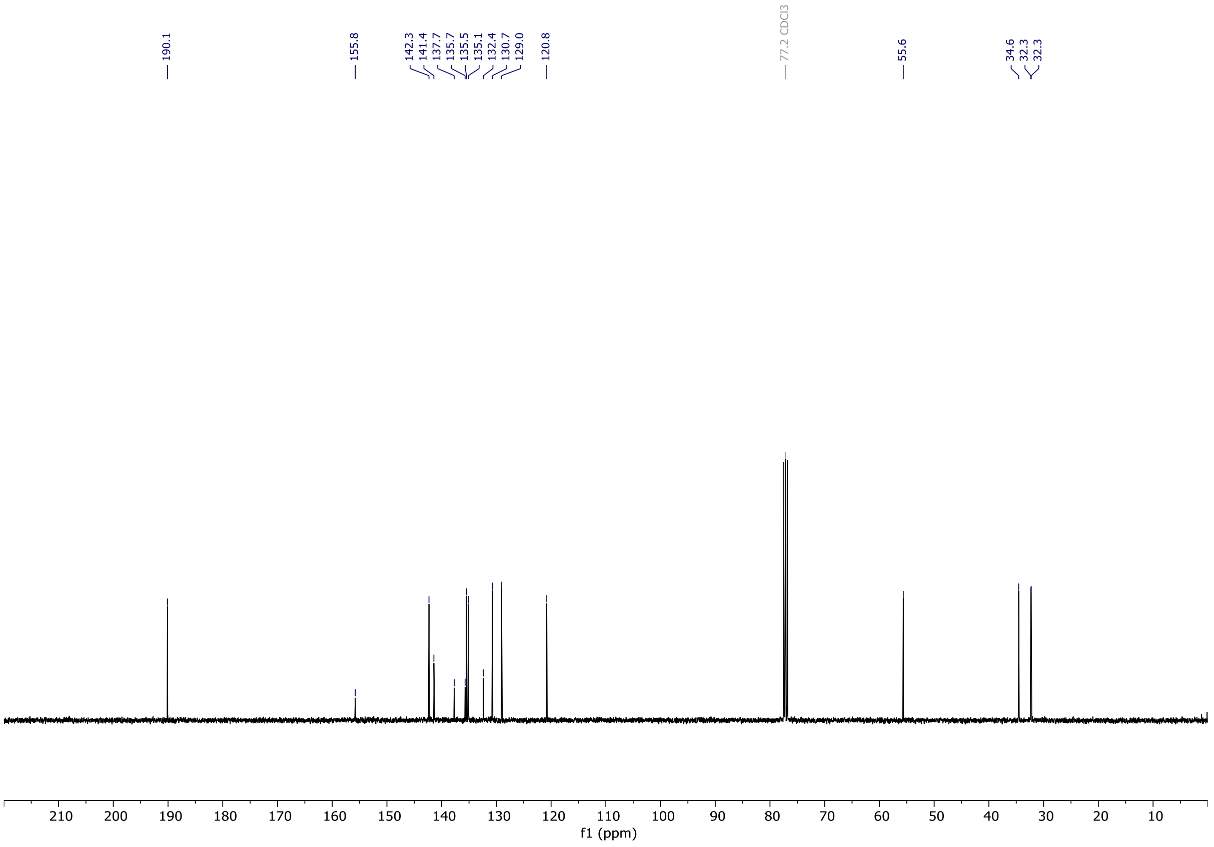

OX04955b

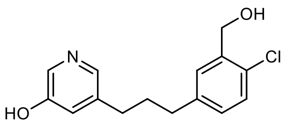

OX04955b

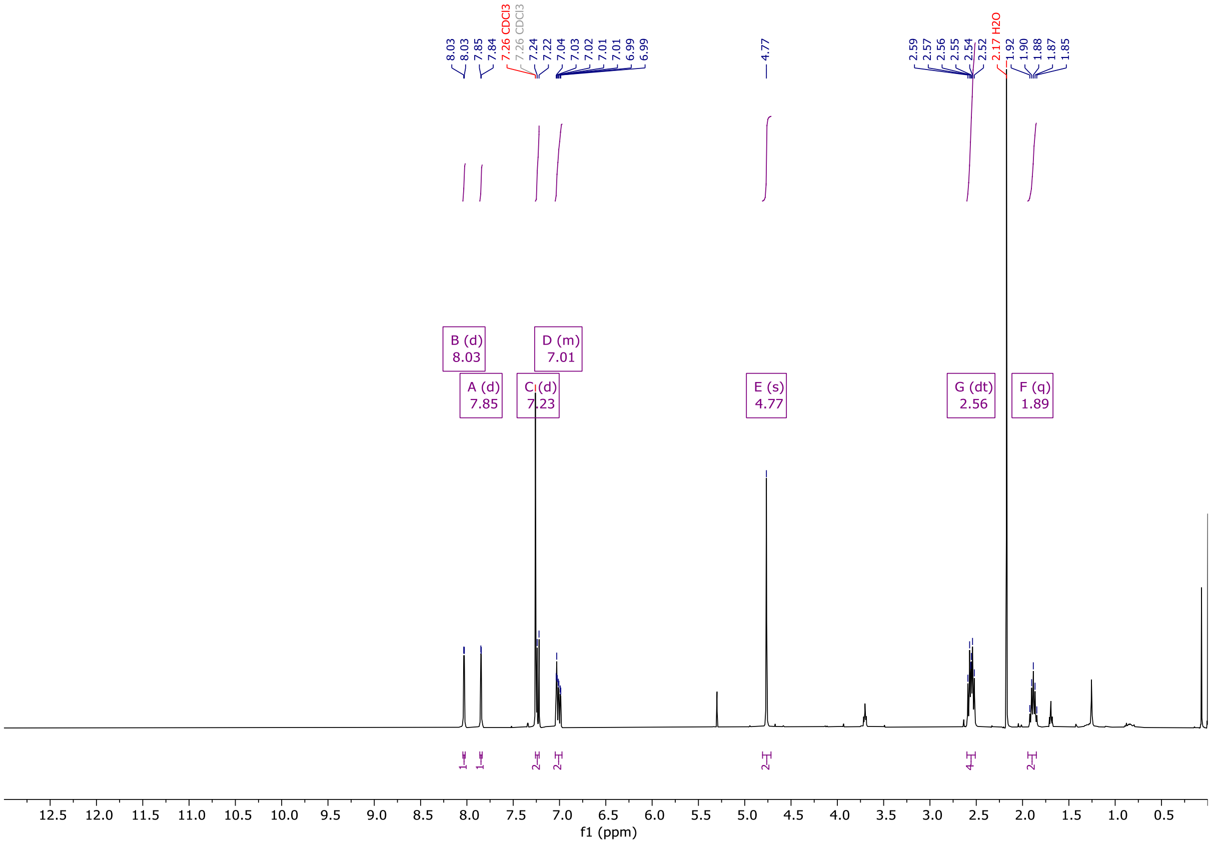

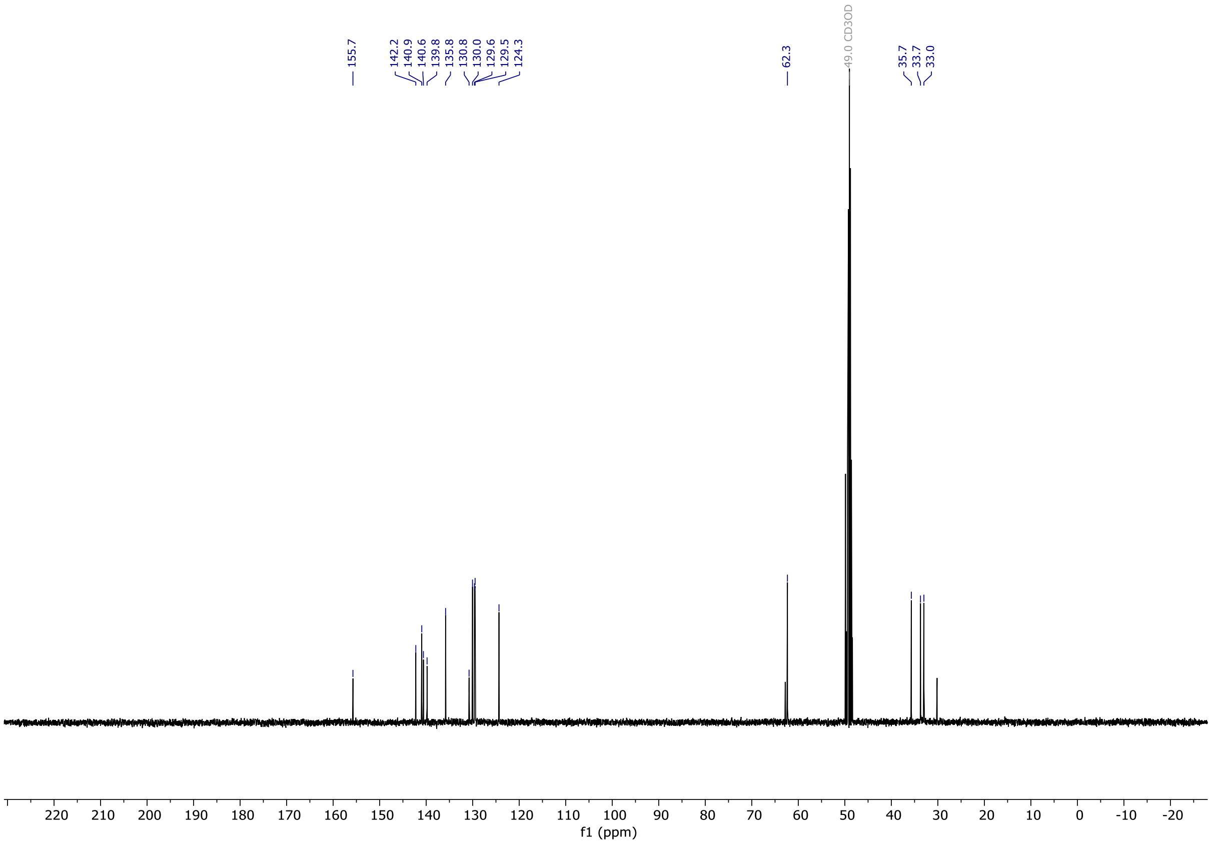

OX04955

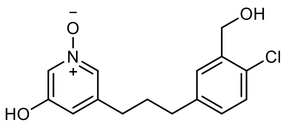

OX04955

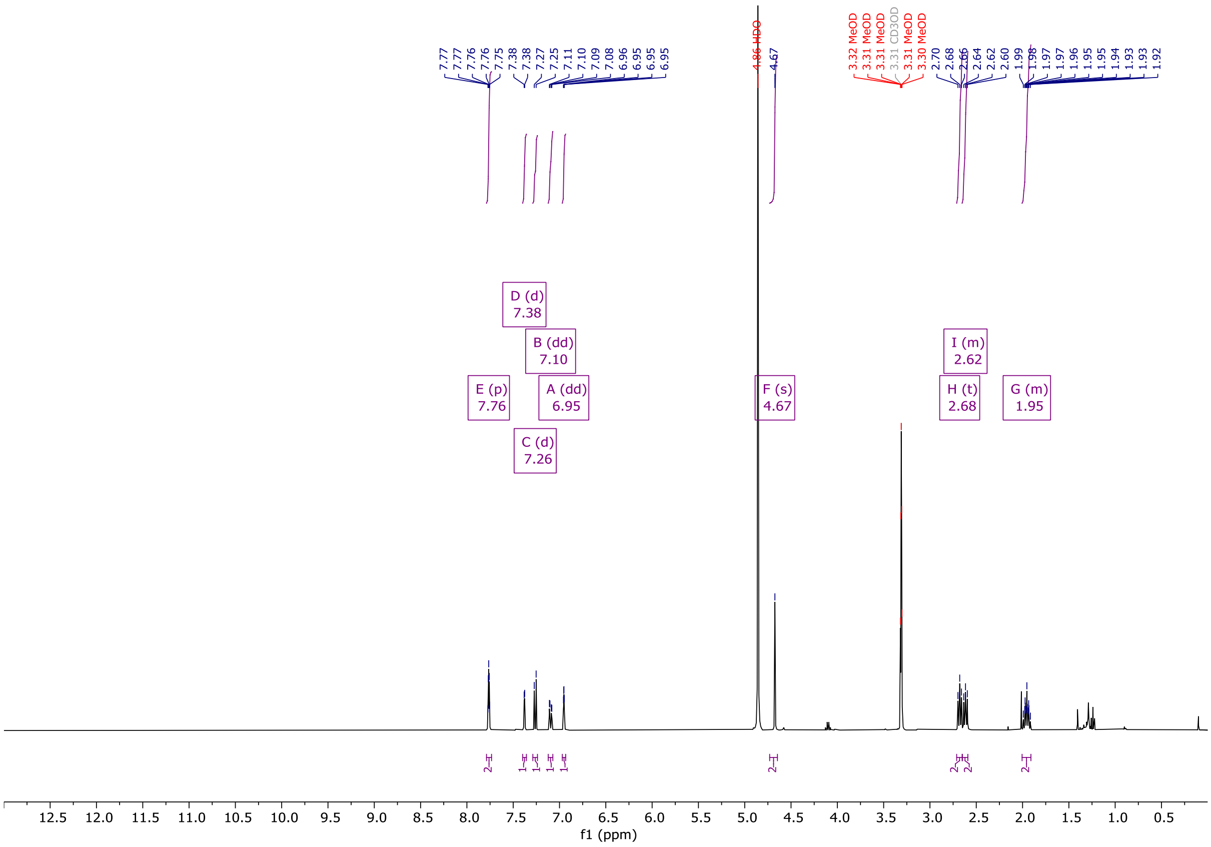

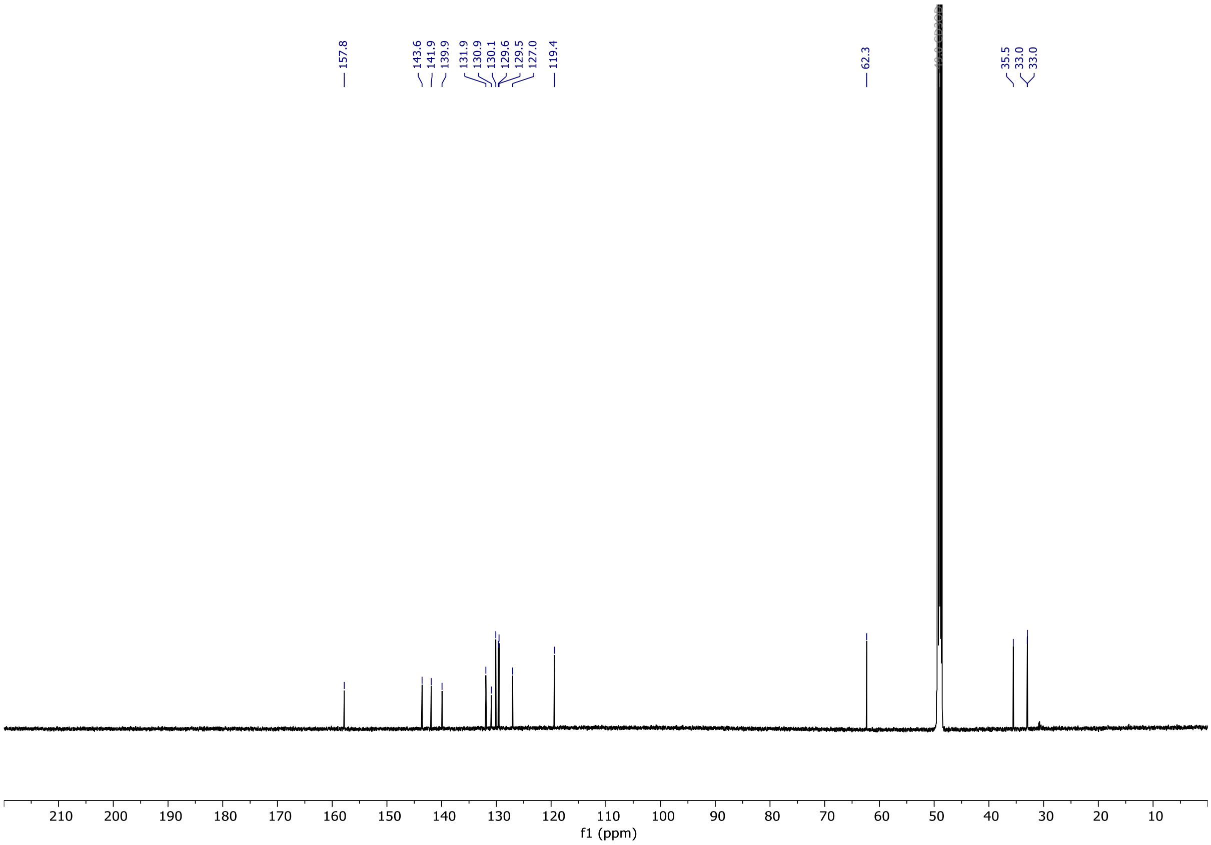

OX04957a

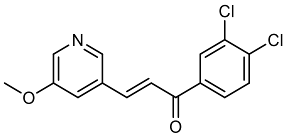

OX04957a

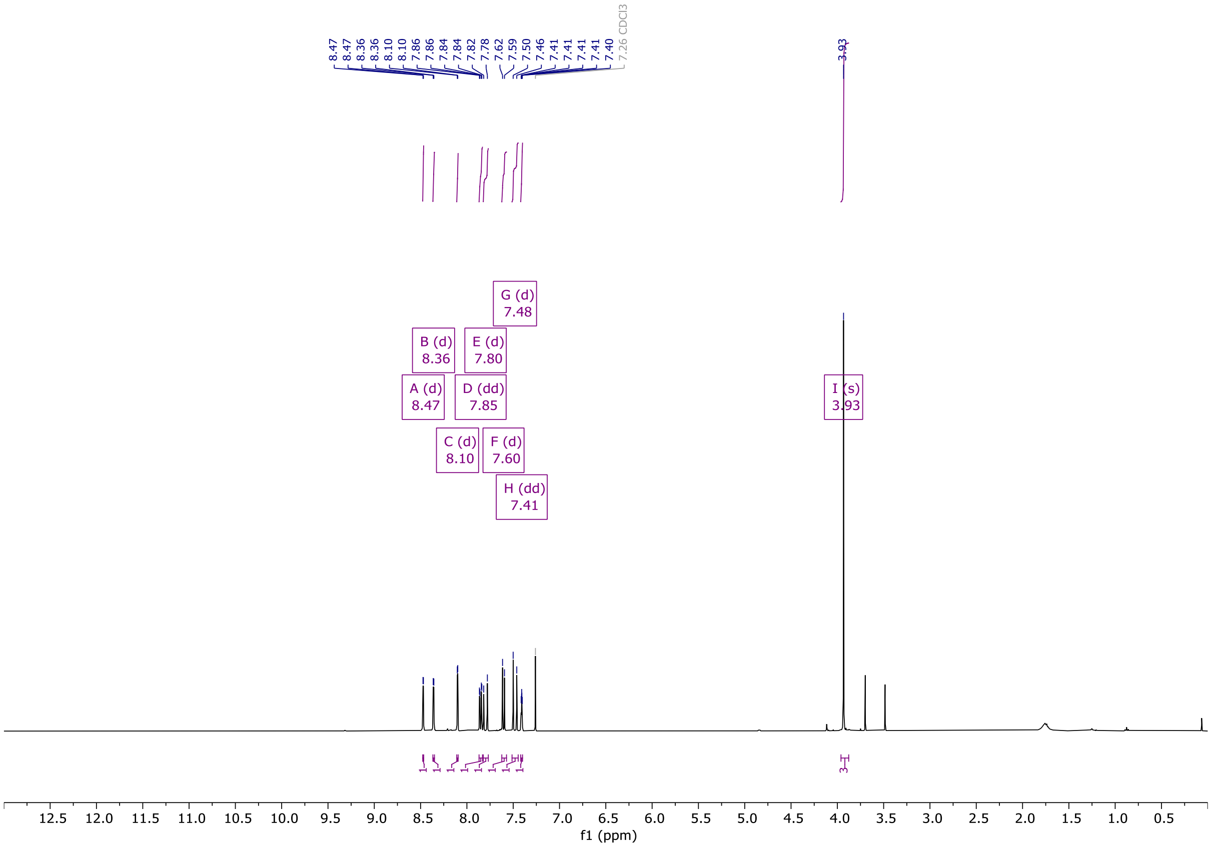

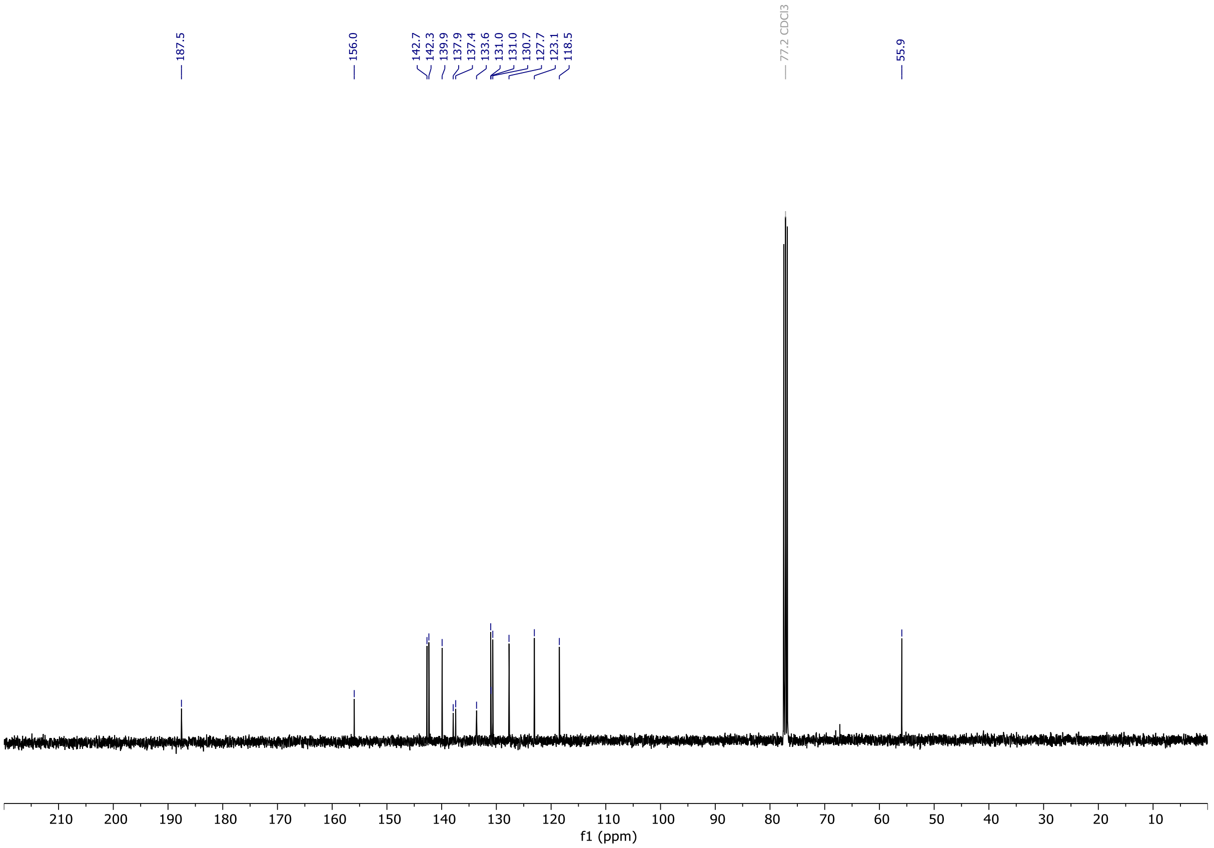

OX04957b

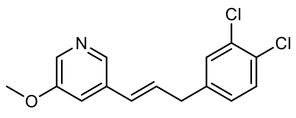

OX04957b

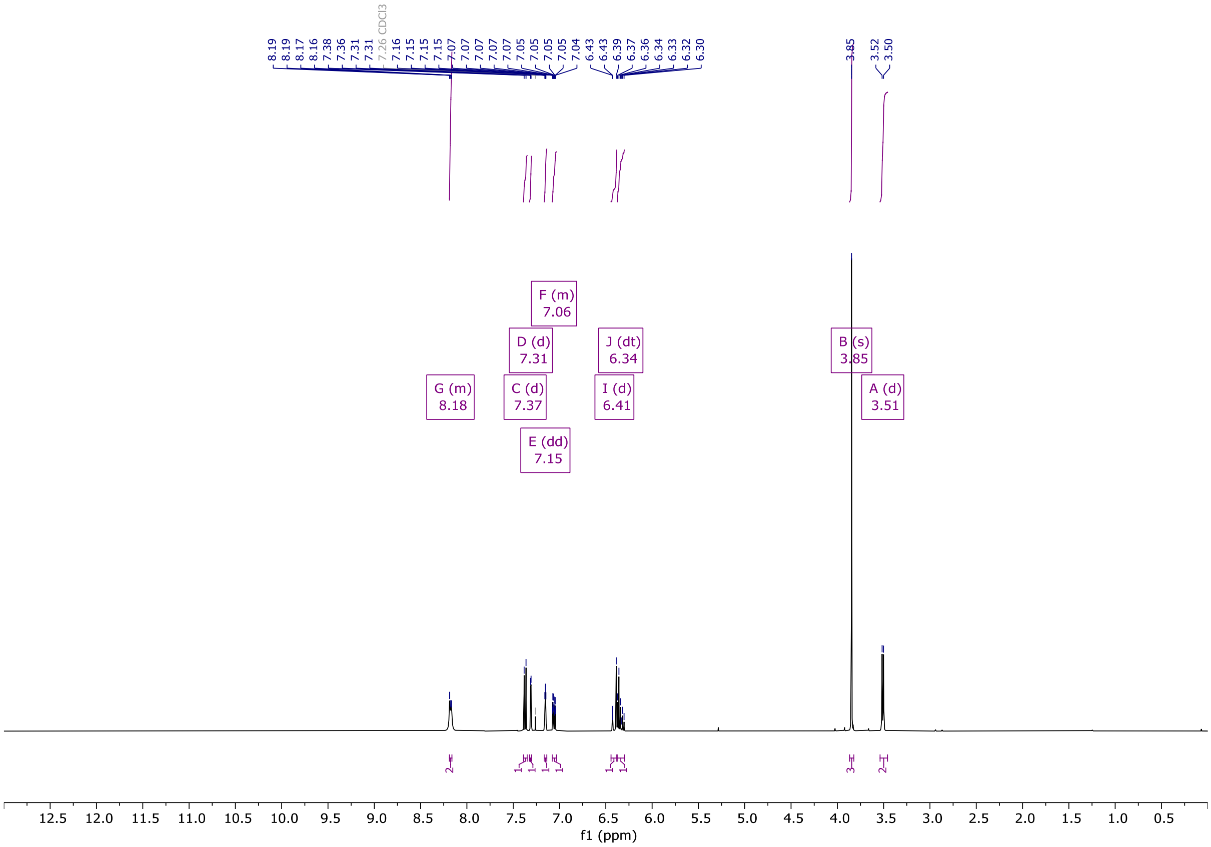

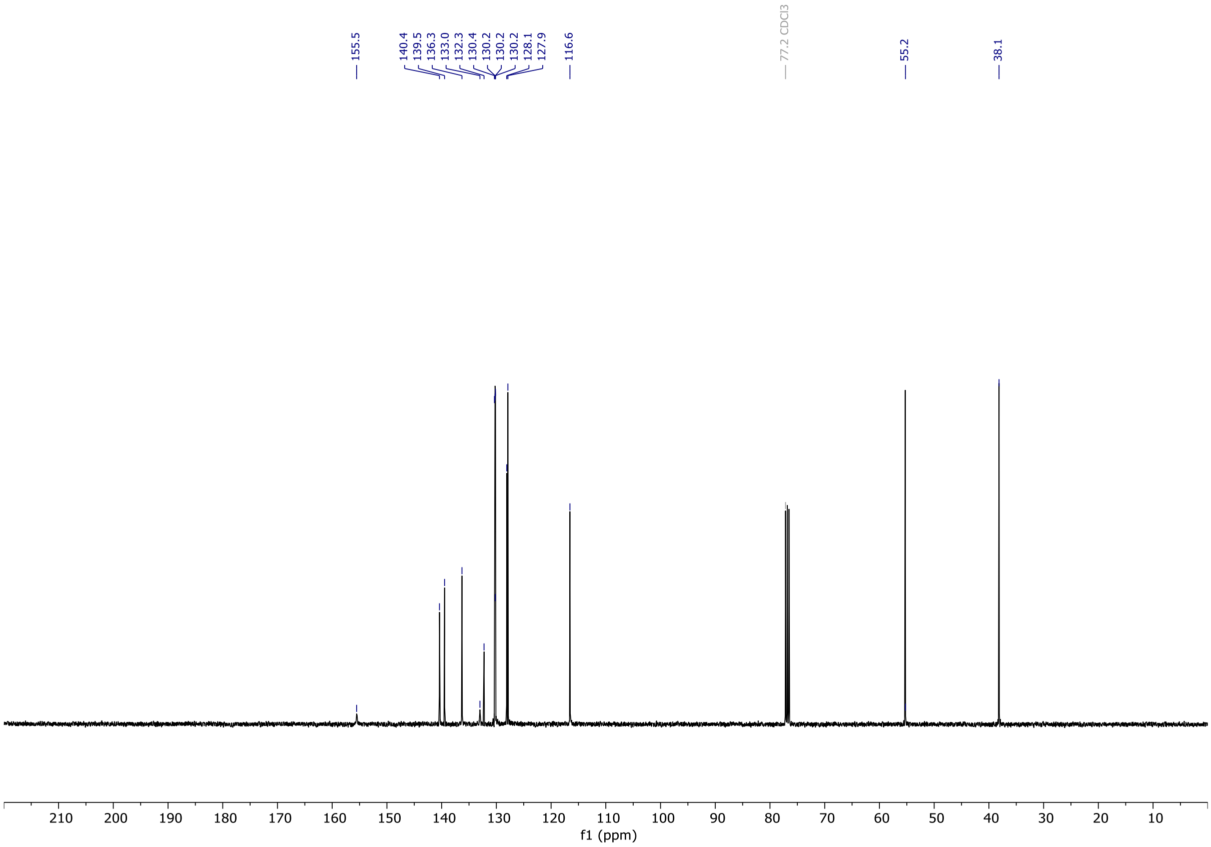

OX04957c

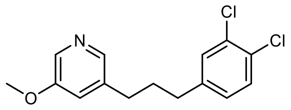

OX04957c

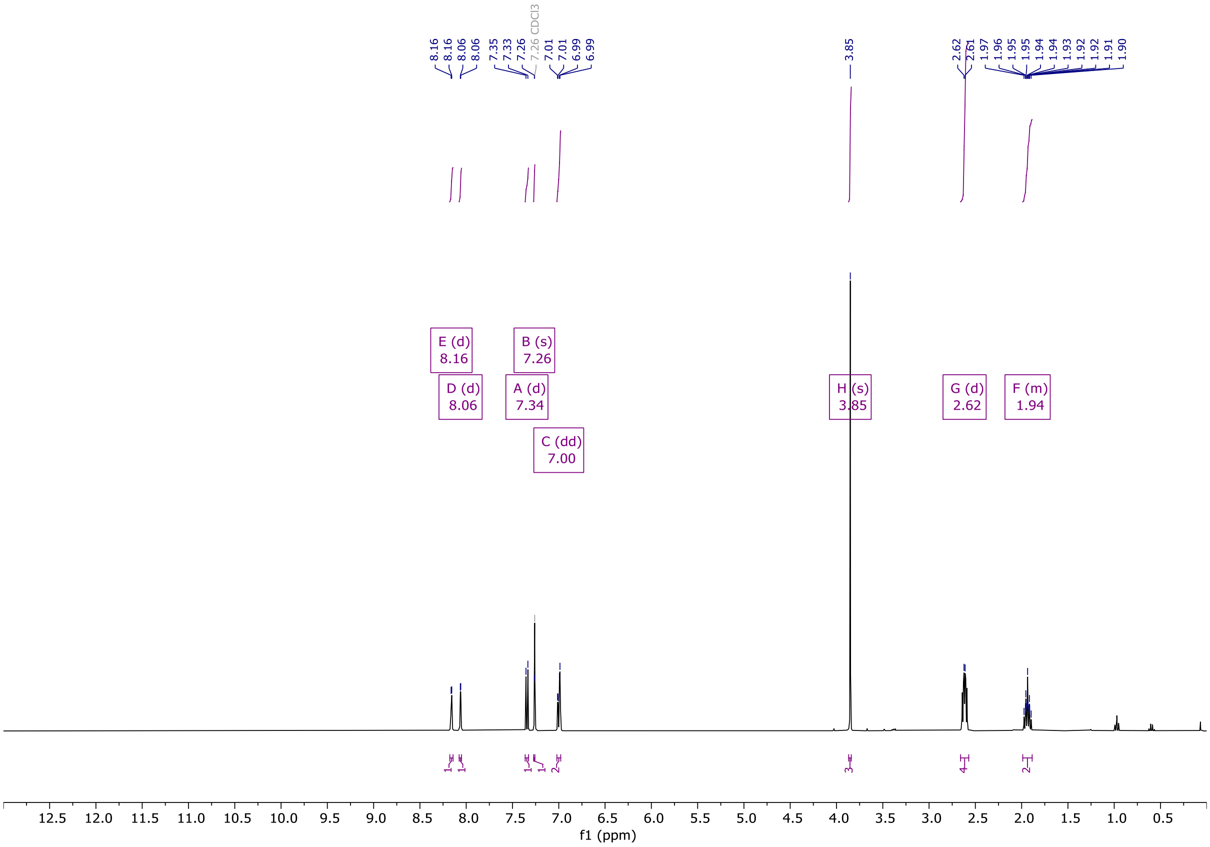

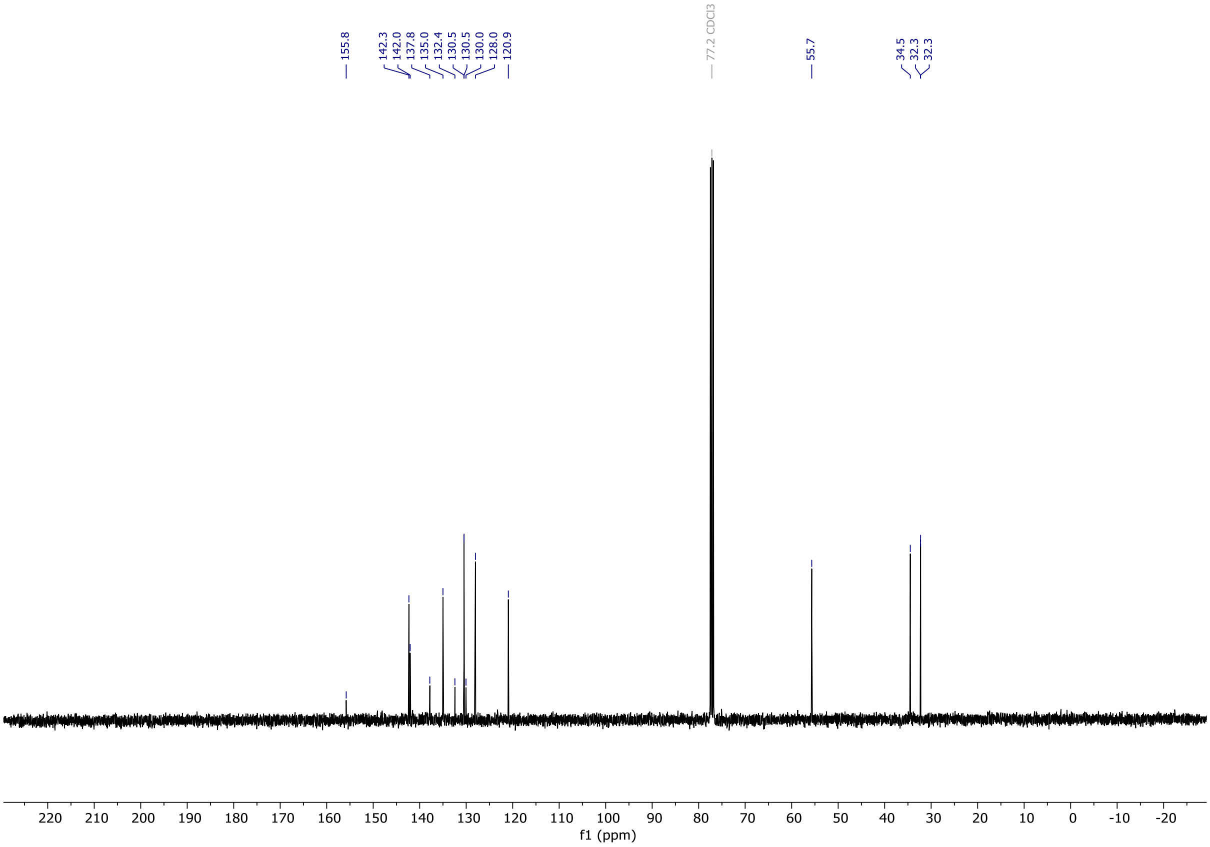

OX04957d

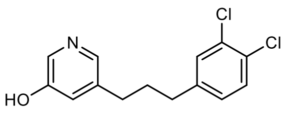

OX04957d

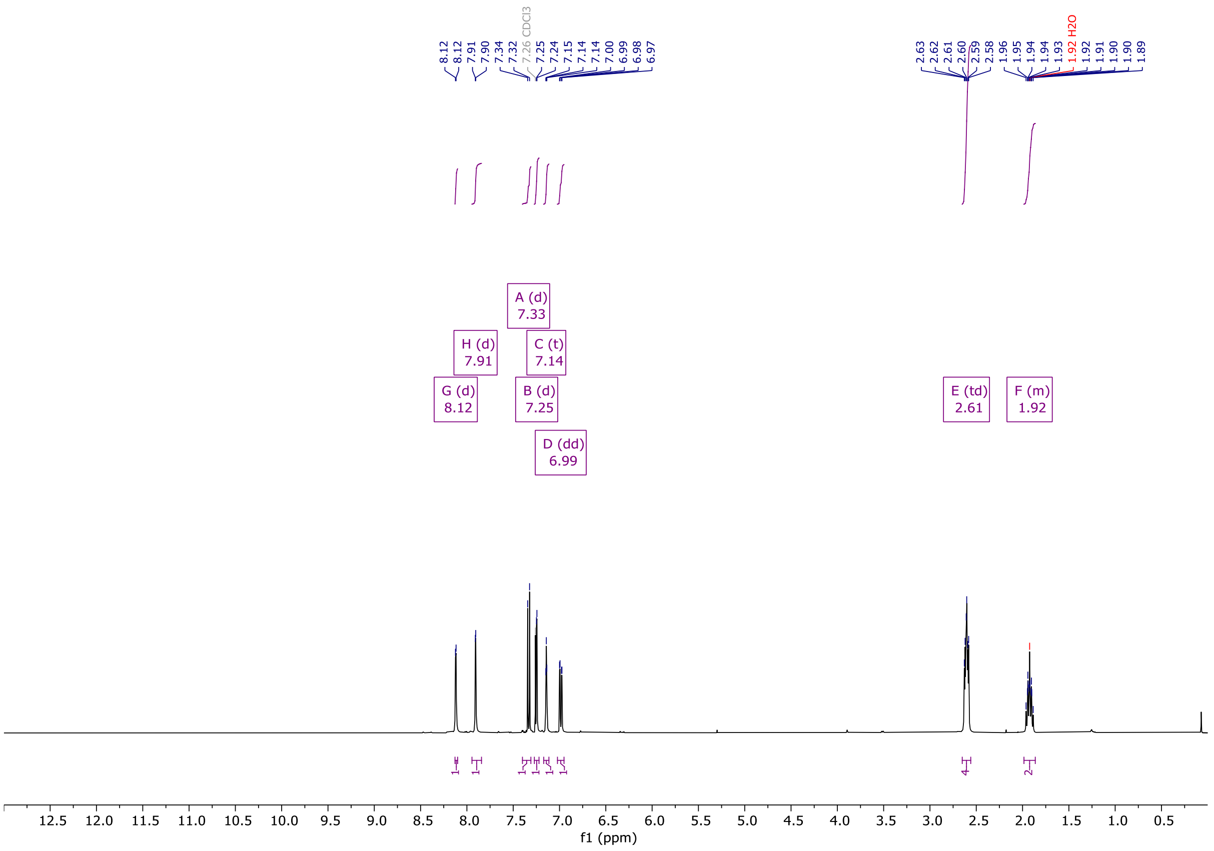

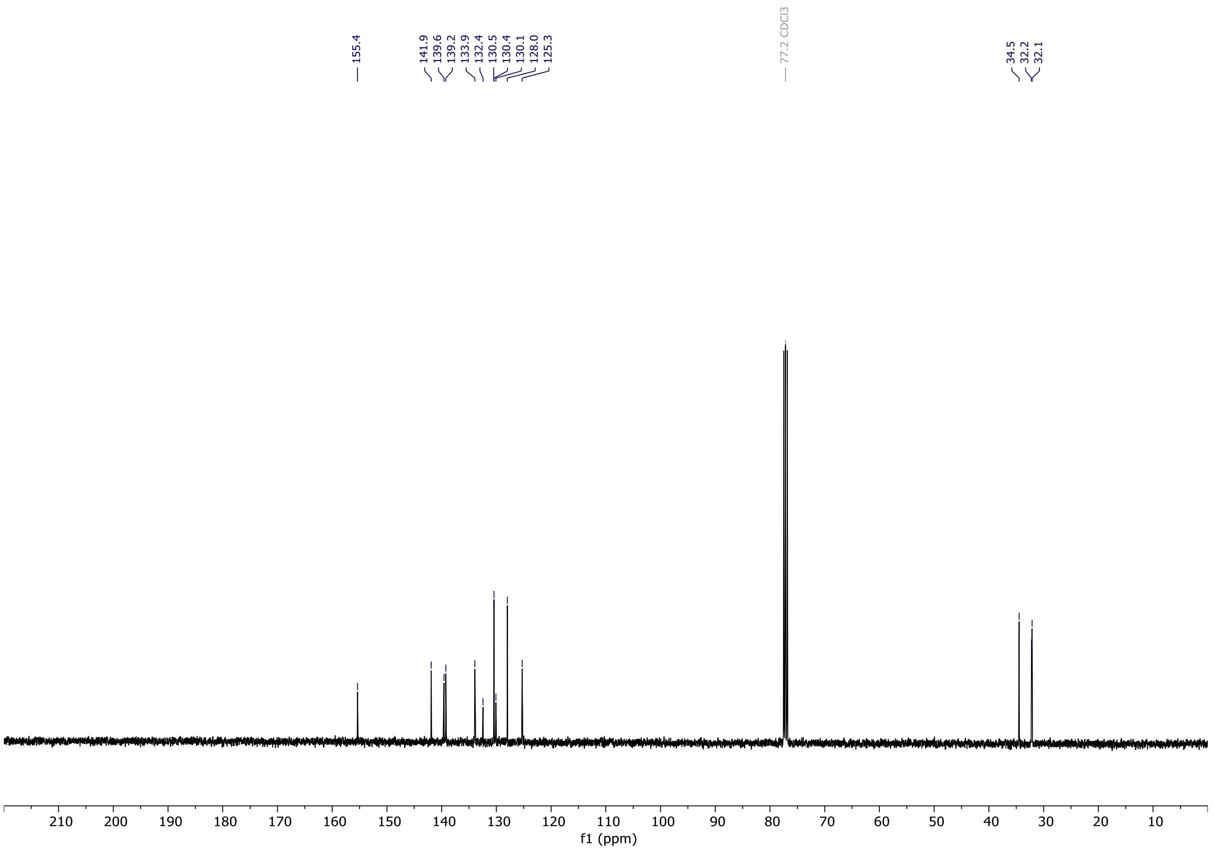

OX04957

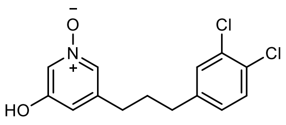

OX04957

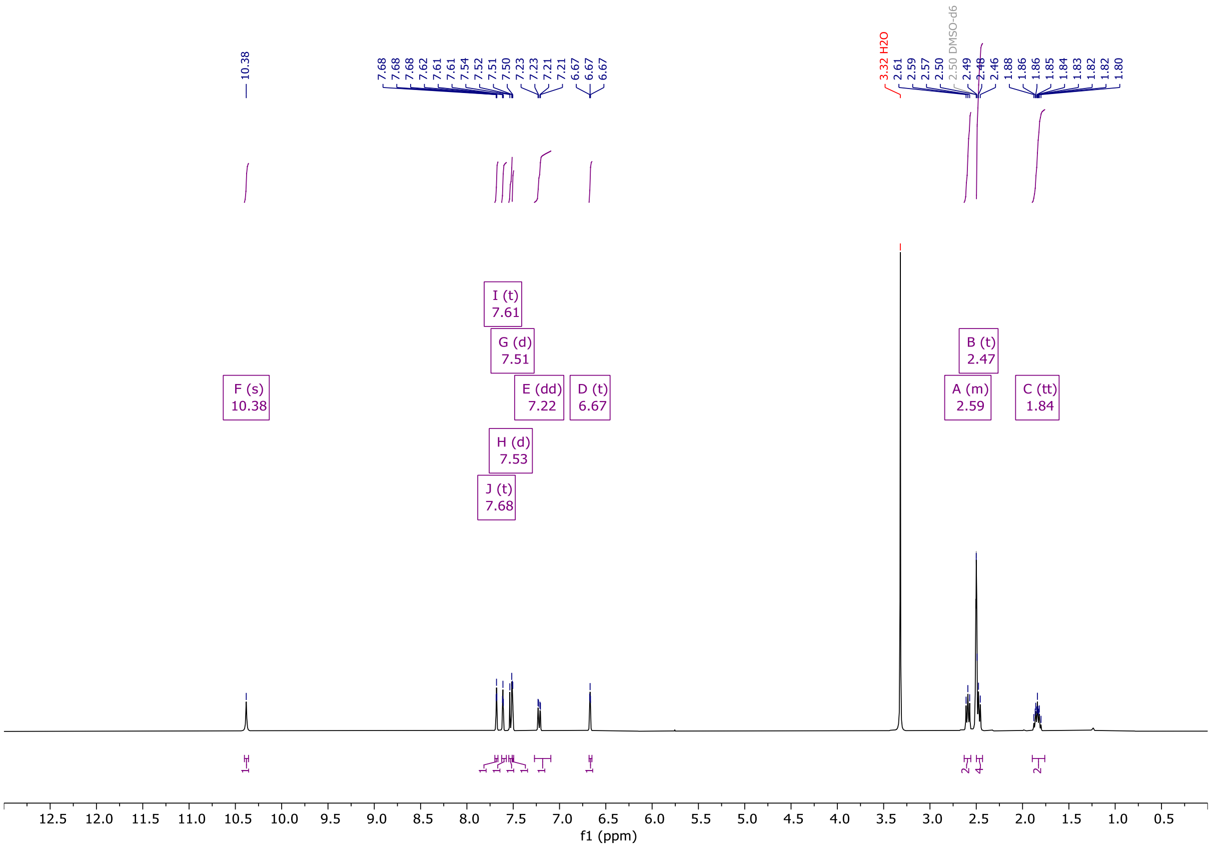

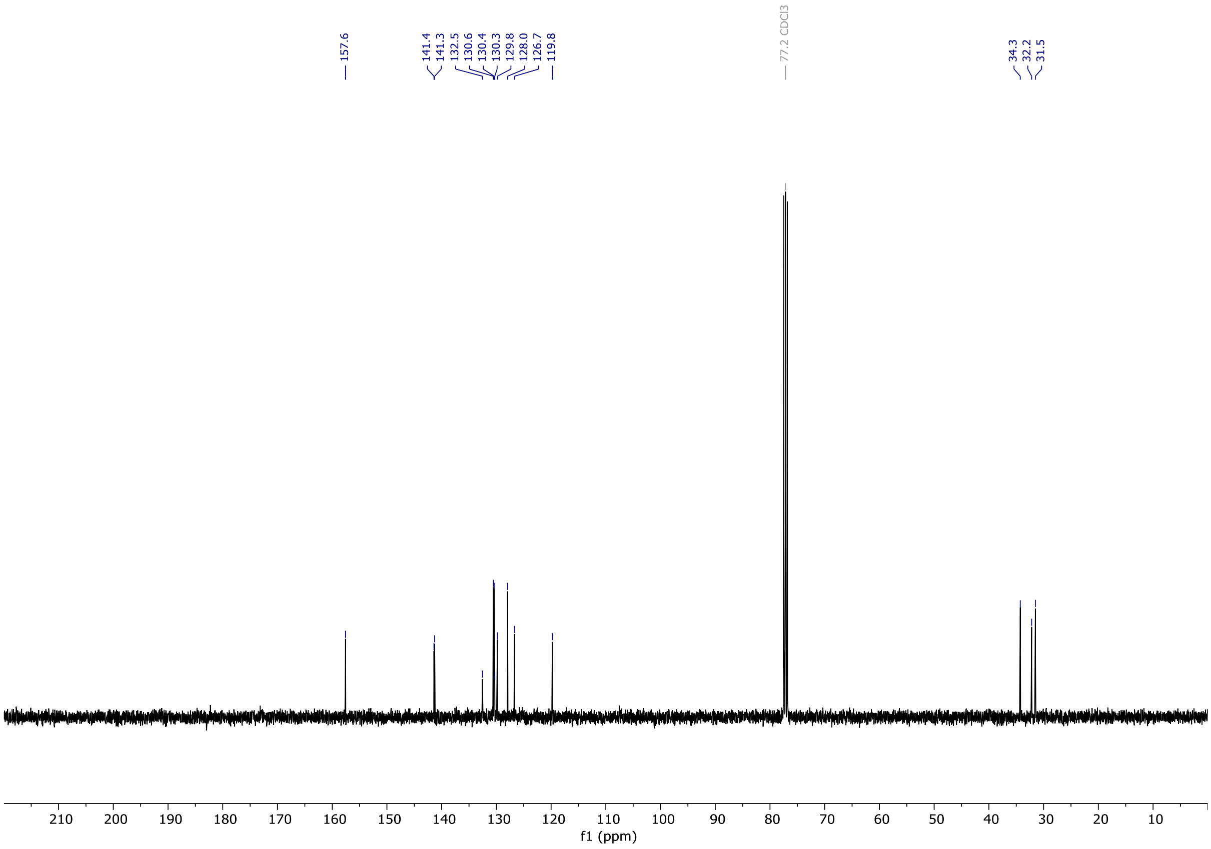

OX04958a

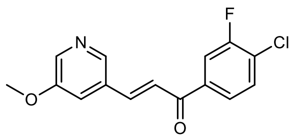

OX04958a

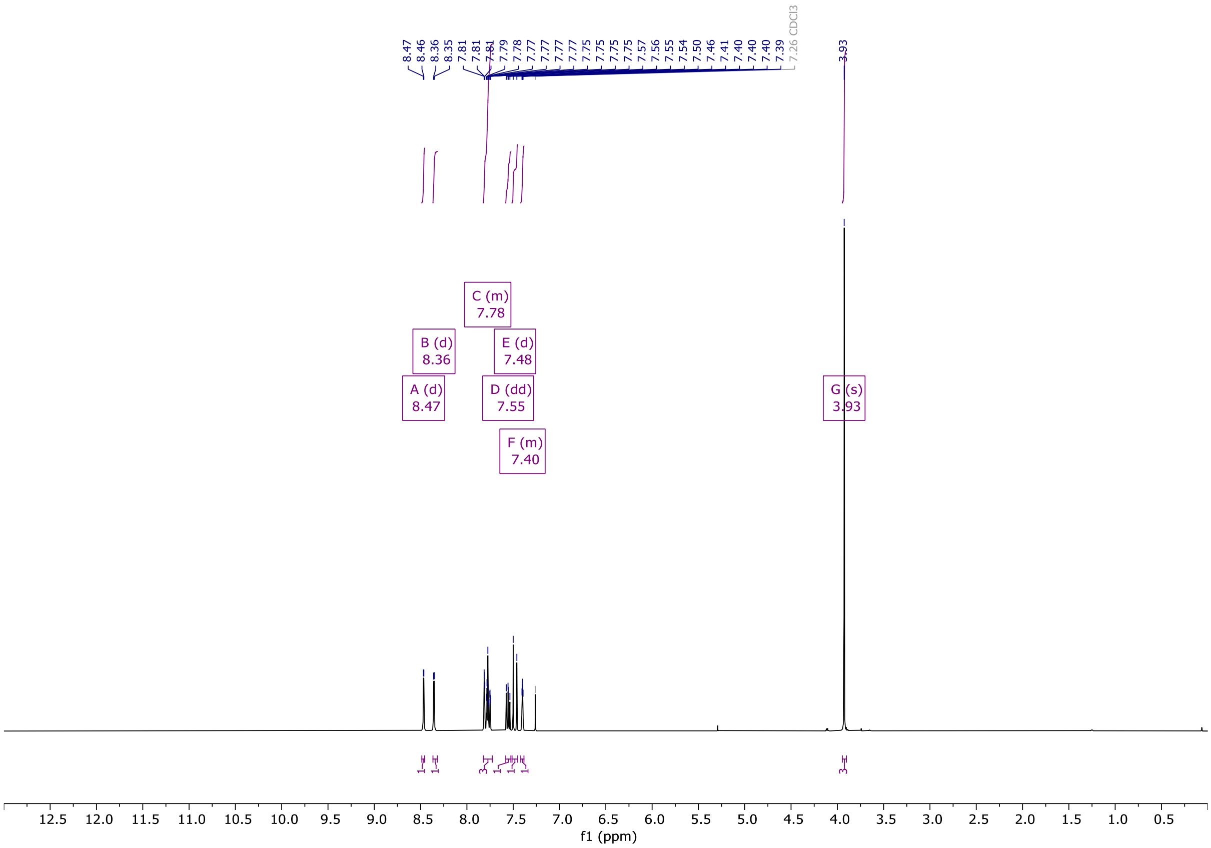

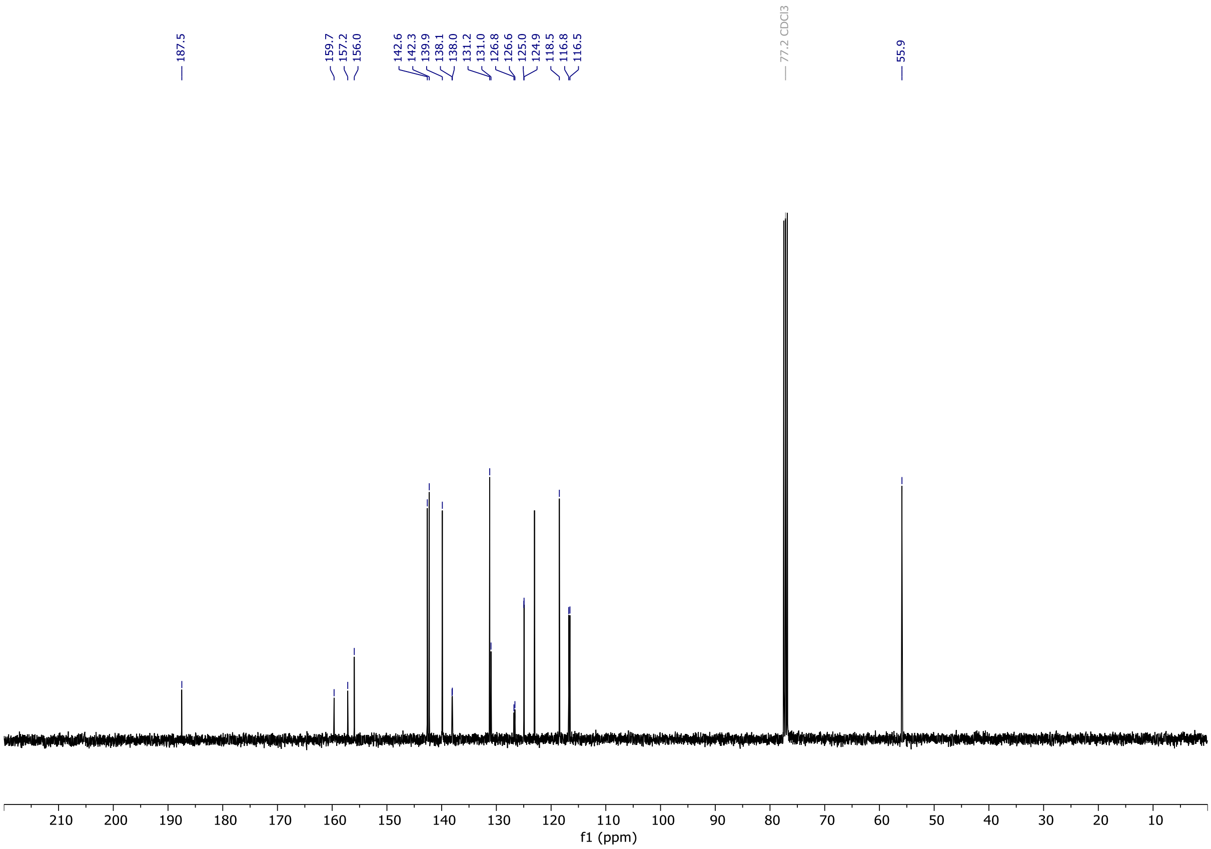

OX04958b

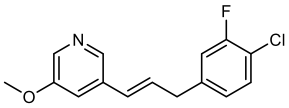

OX04958b

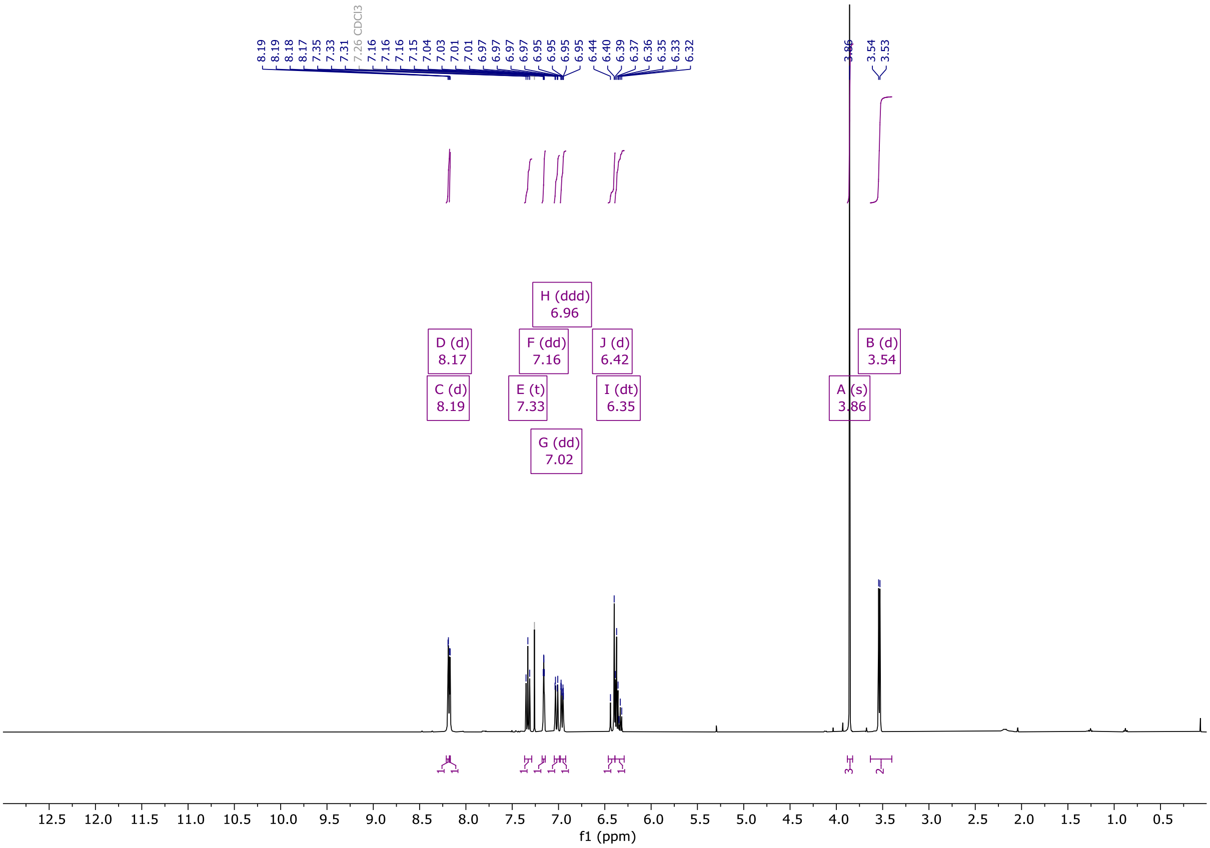

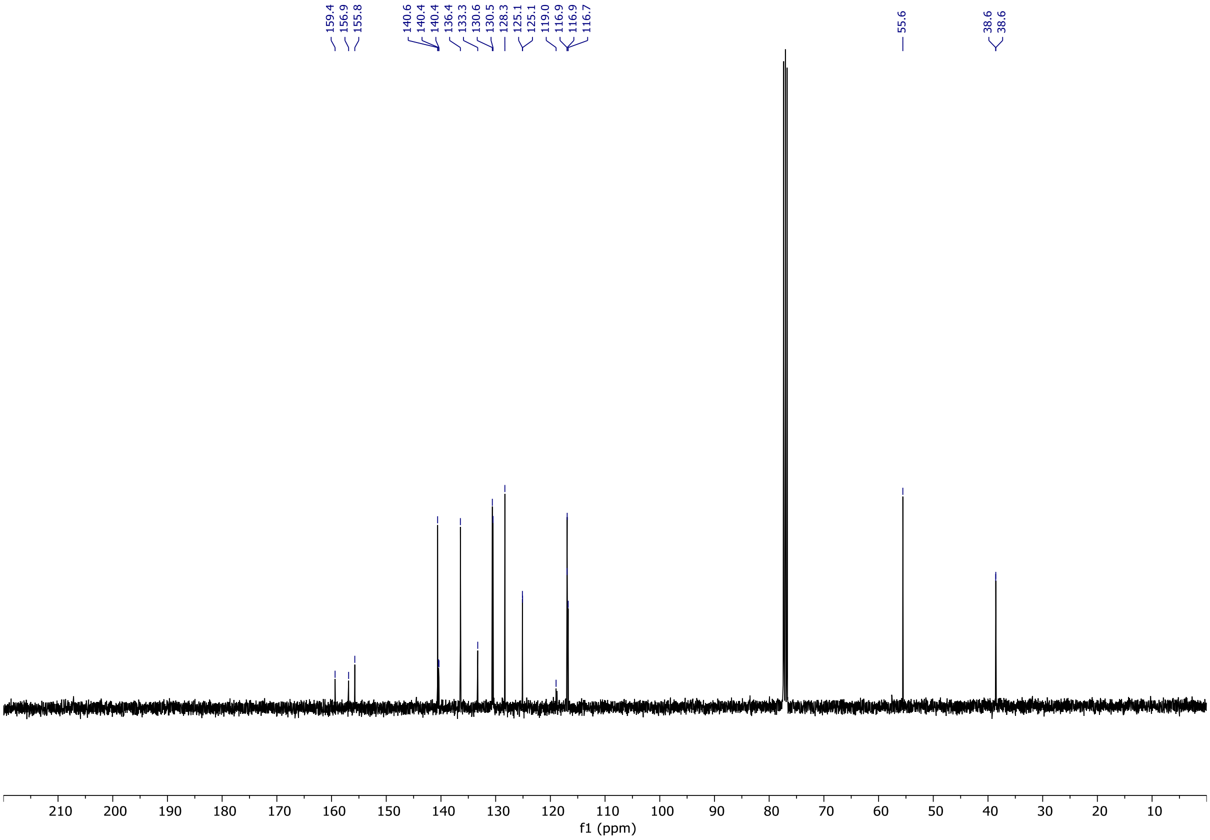

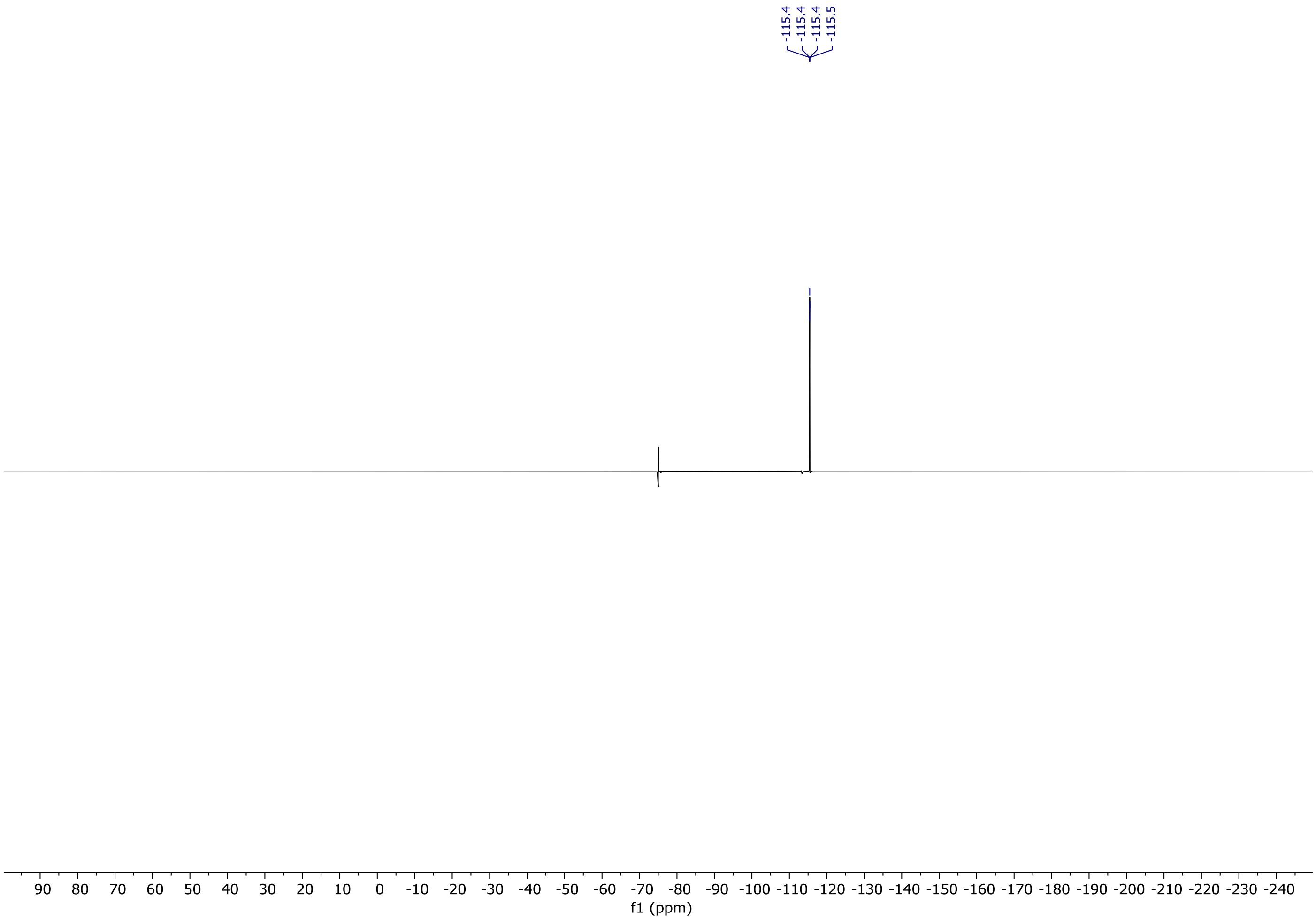
-115.4
-115.4
-115.4
-115.5
90 80 70 60 50 40 30 20 10 0 -10 -20 -30 -40 -50 -60 -70 -80 -90 -100 -110 -120 -130 -140 -150 -160 -170 -180 -190 -200 -210 -220 -230 -240
f1 (ppm)

OX04958c

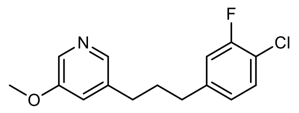

OX04958c

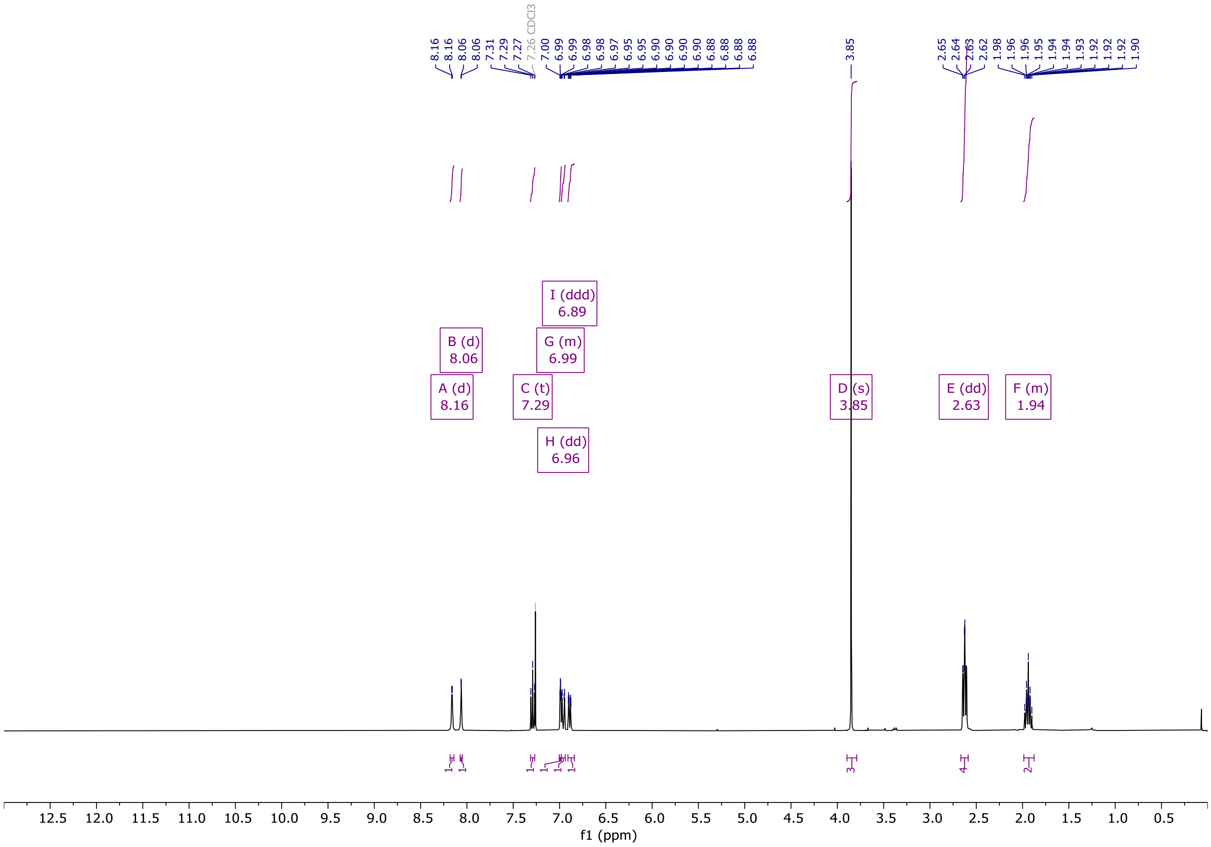

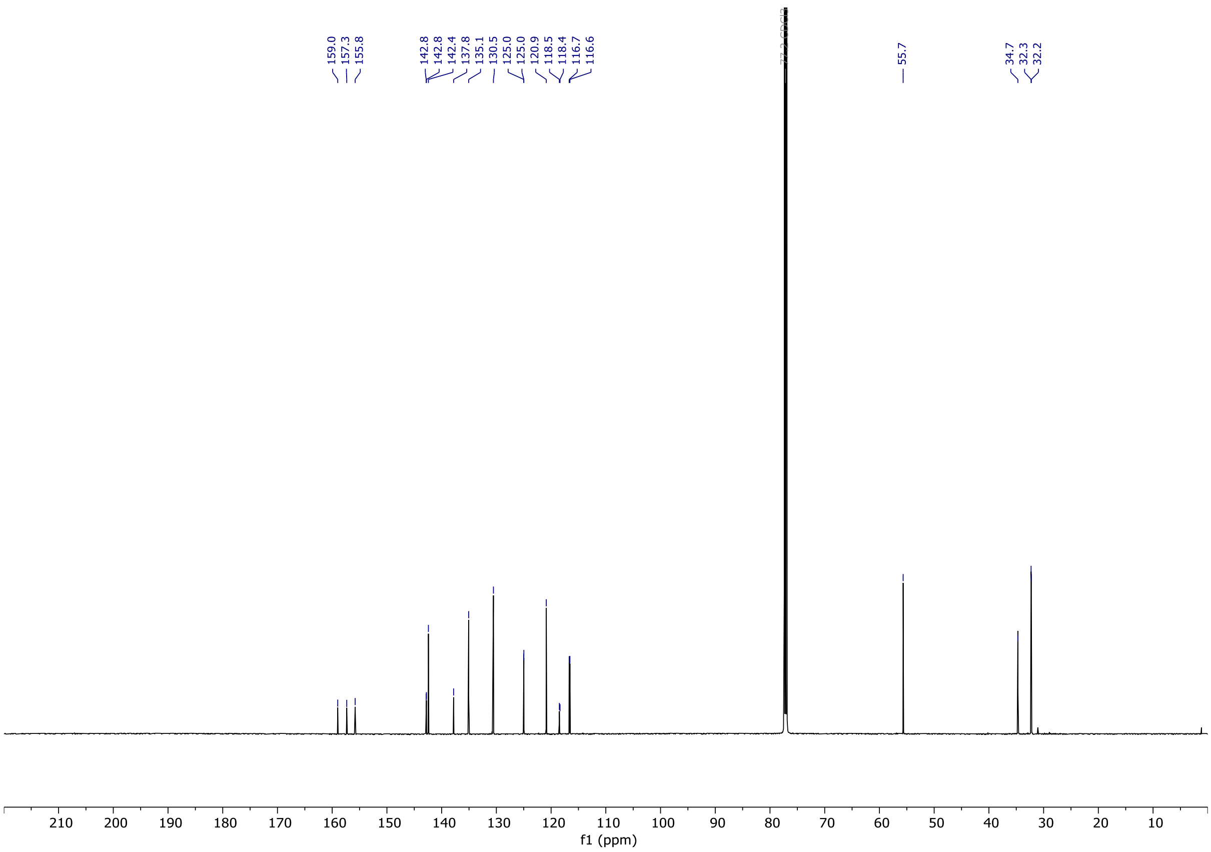

OX04958d

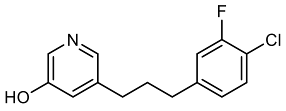

OX04958d

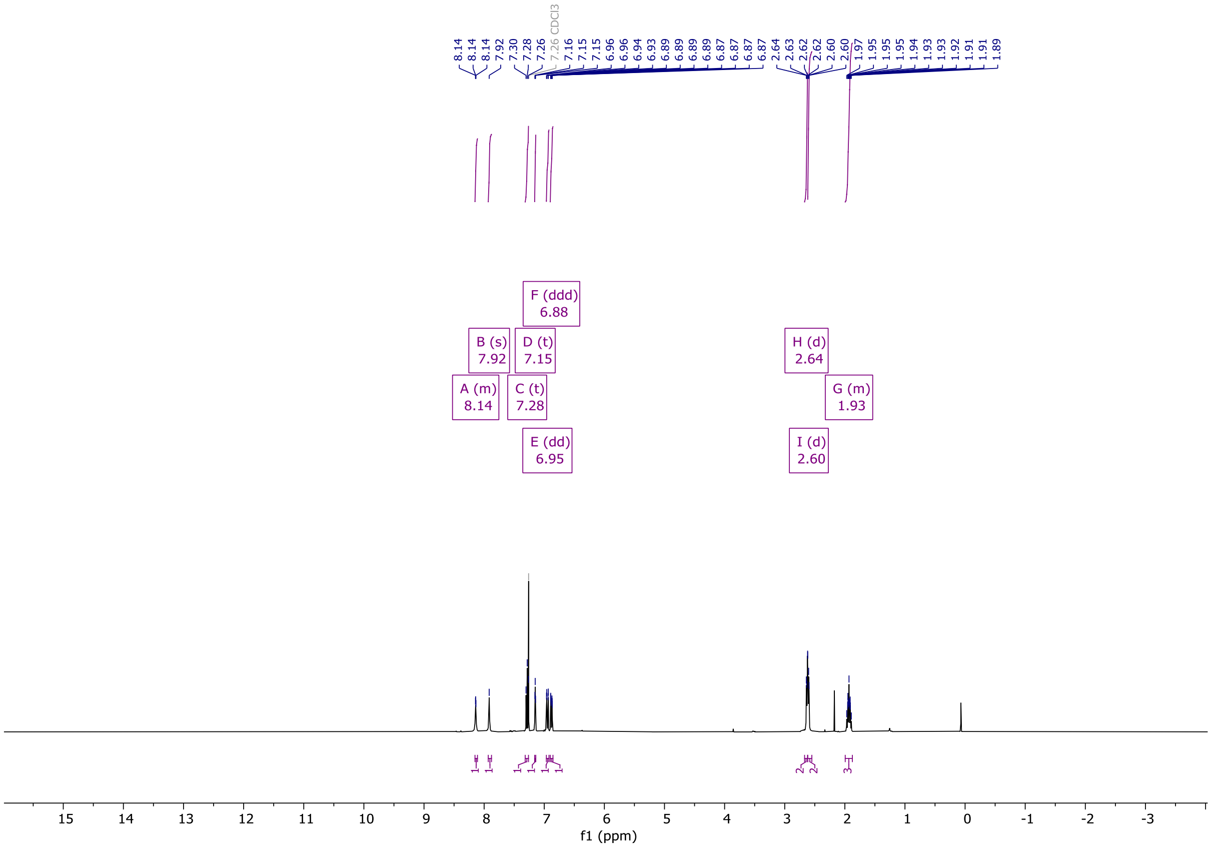

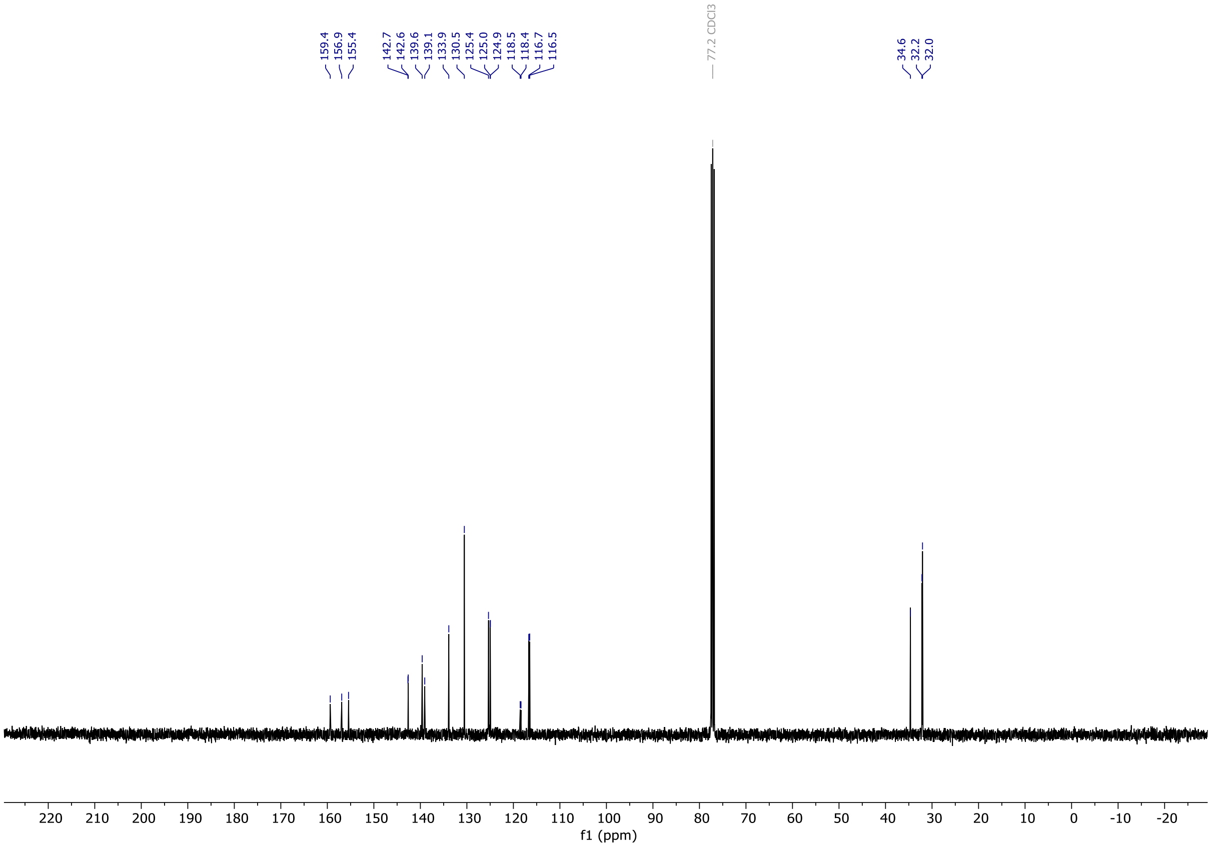

OX04958

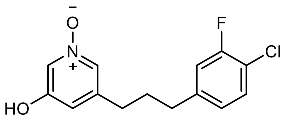

OX04958

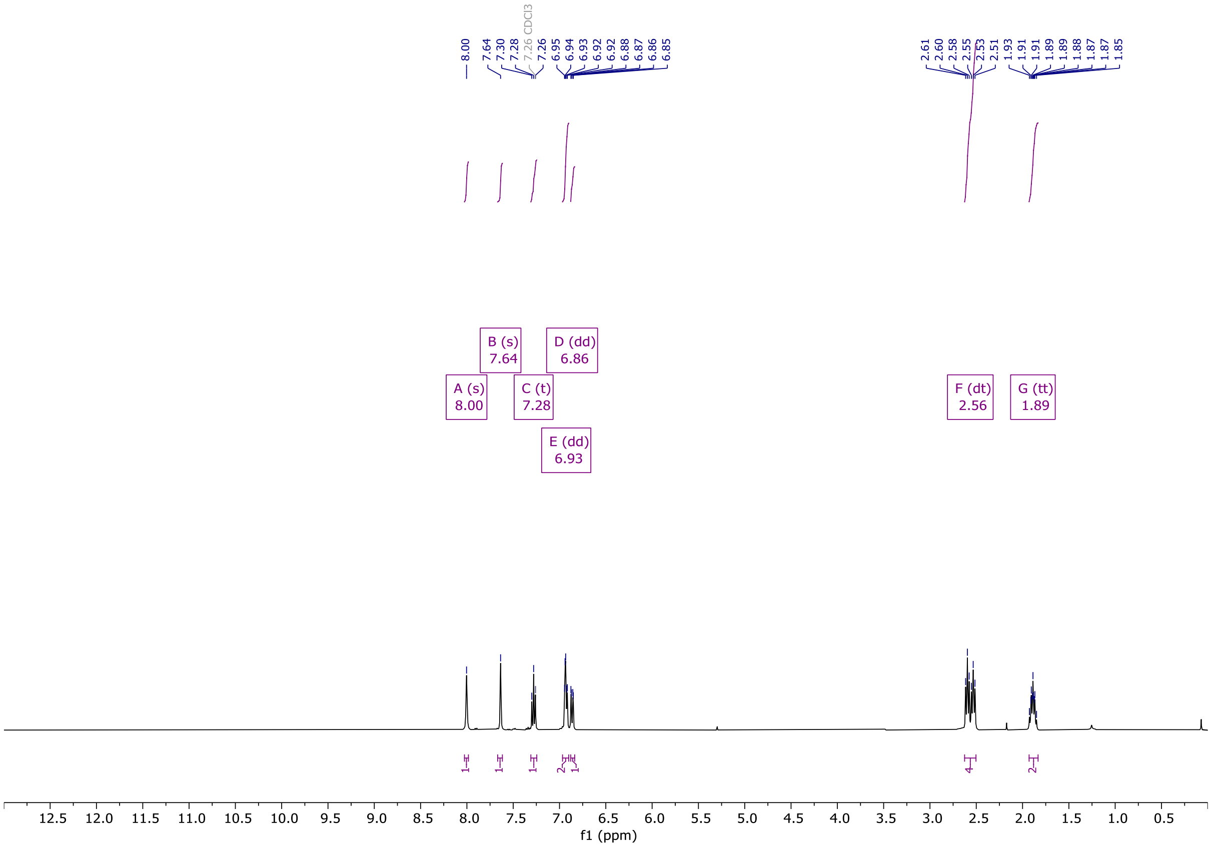

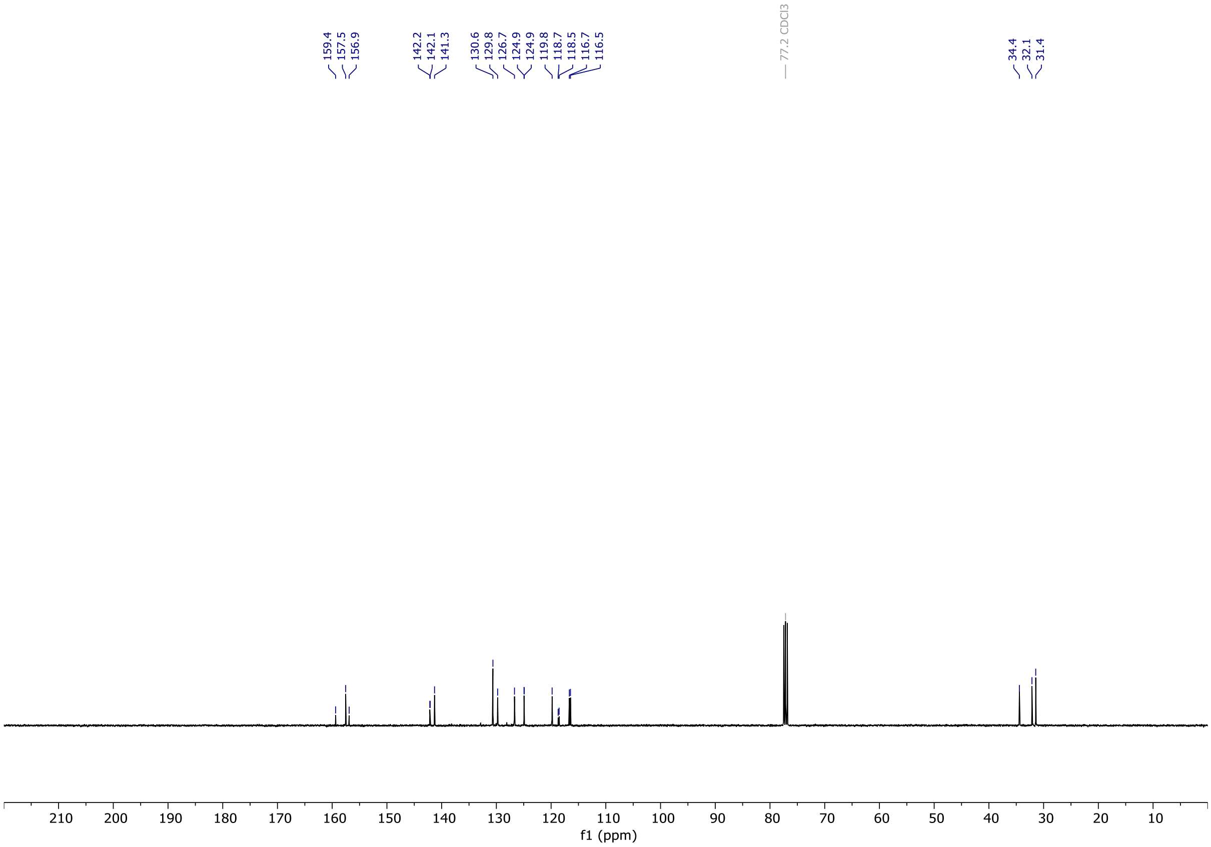

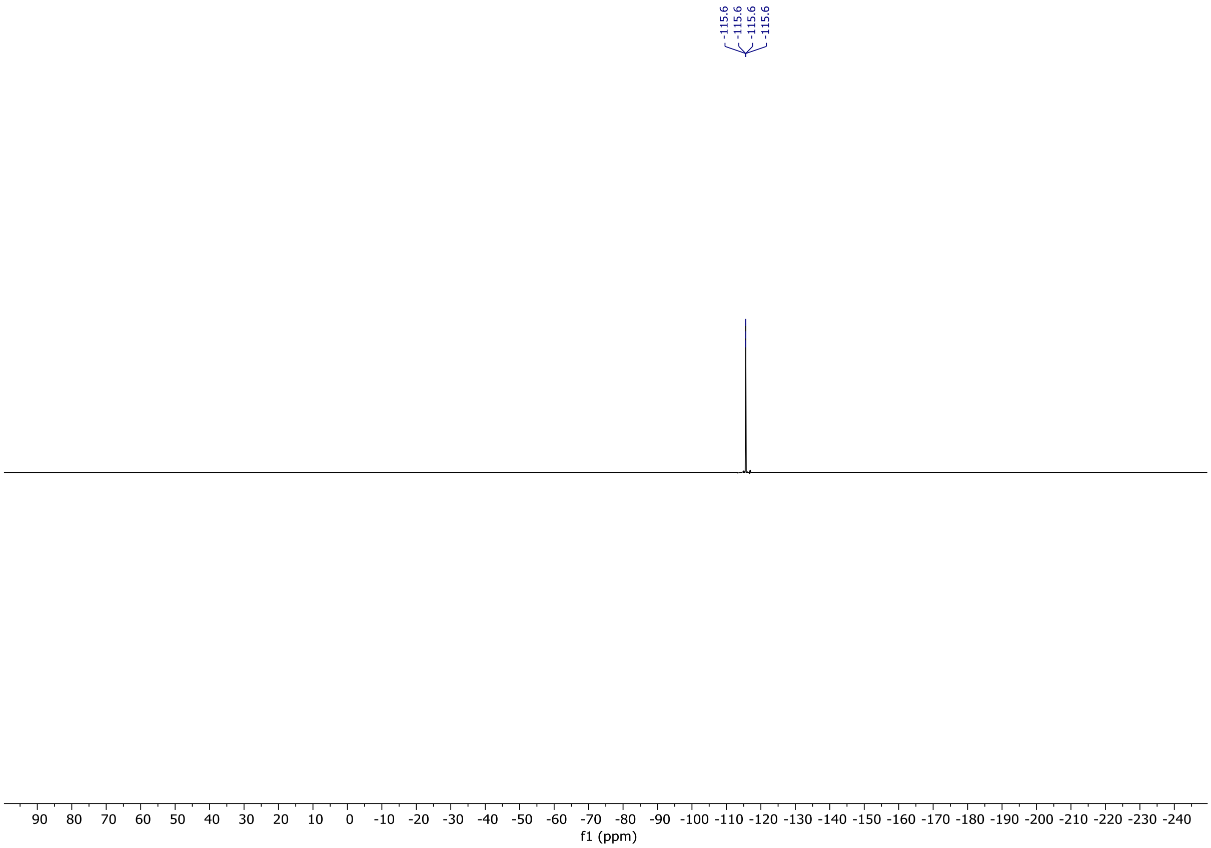
-115.6
-115.6
-115.6
-115.6
90 80 70 60 50 40 30 20 10 0 -10 -20 -30 -40 -50 -60 -70 -80 -90 -100 -110 -120 -130 -140 -150 -160 -170 -180 -190 -200 -210 -220 -230 -240
f1 (ppm)

OX04959a

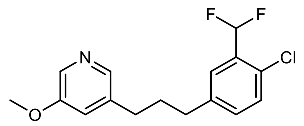

OX04959a

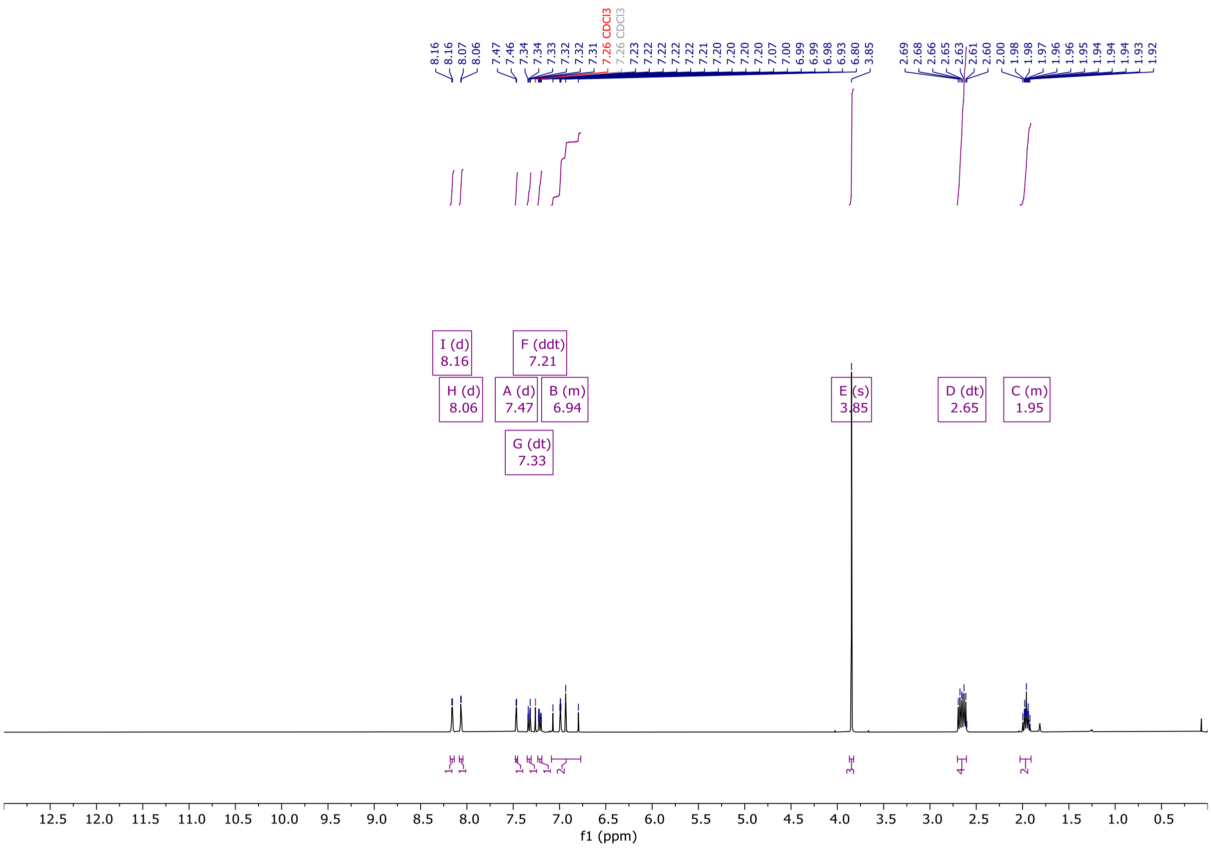

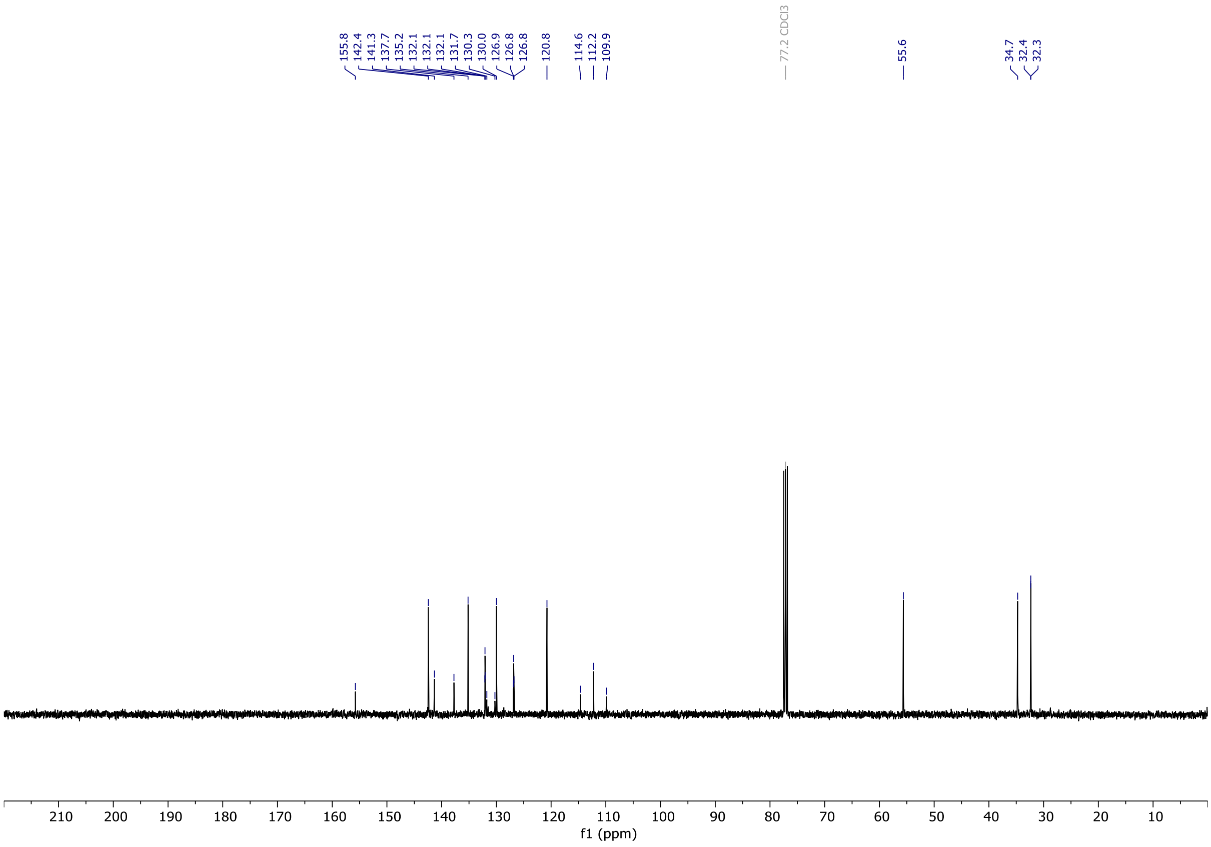

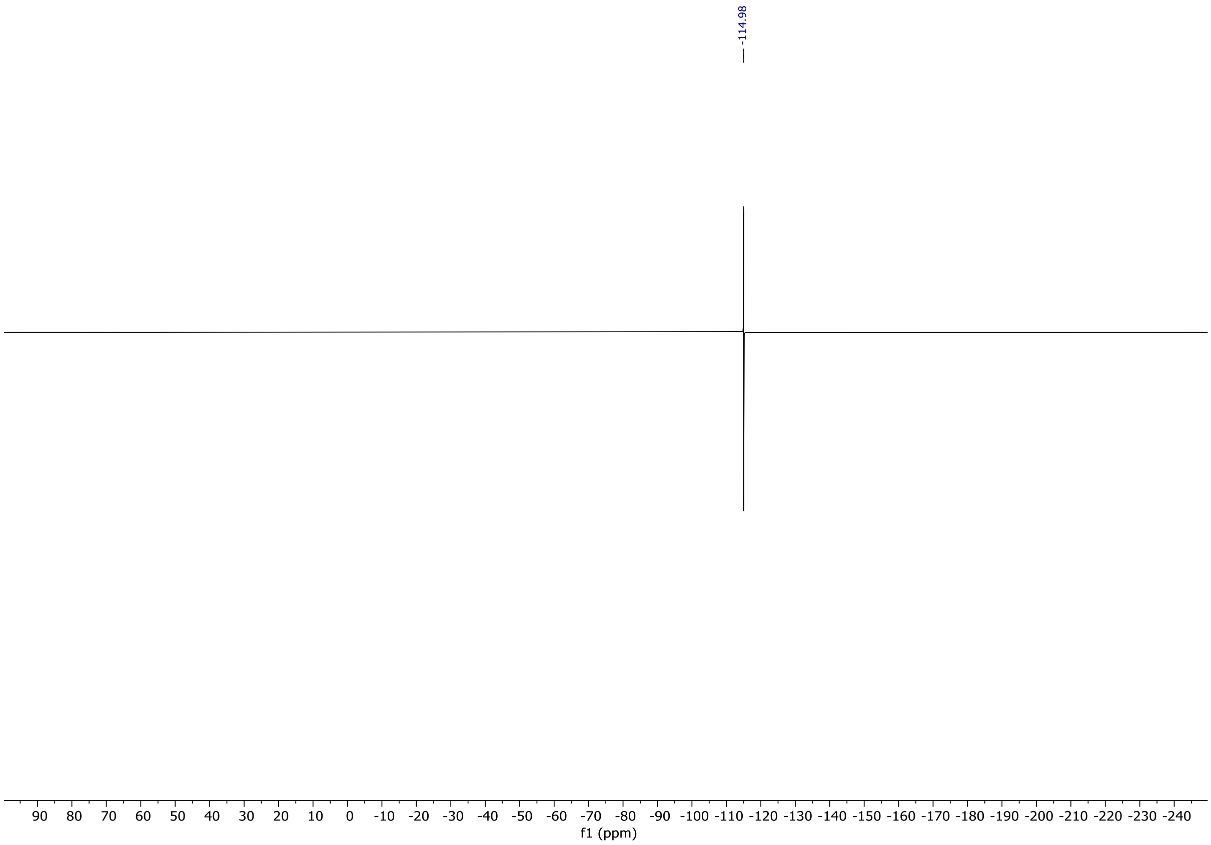

-114.98
90 80 70 60 50 40 30 20 10 0 -10 -20 -30 -40 -50 -60 -70 -80 -90 -100 -110 -120 -130 -140 -150 -160 -170 -180 -190 -200 -210 -220 -230 -240
f1 (ppm)

OX04959b

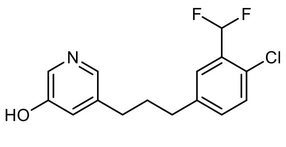

OX04959b

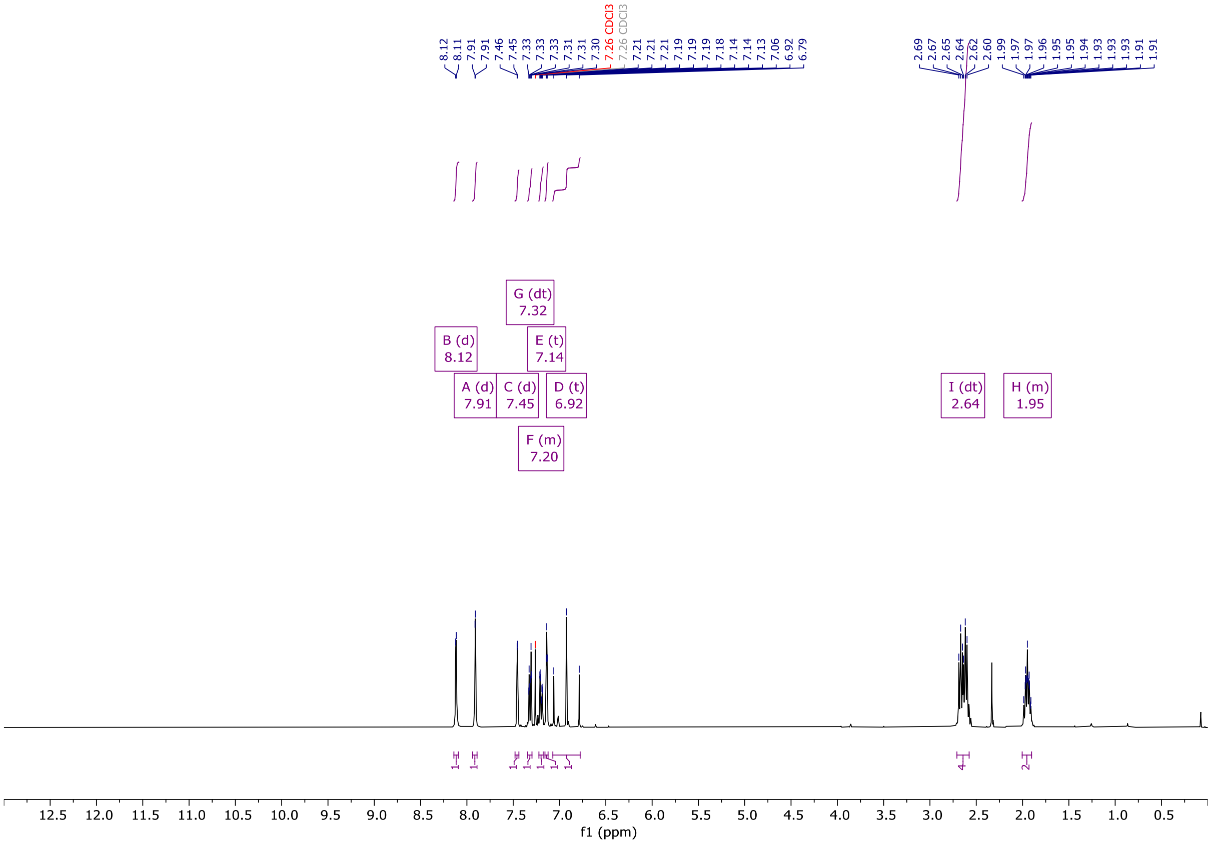

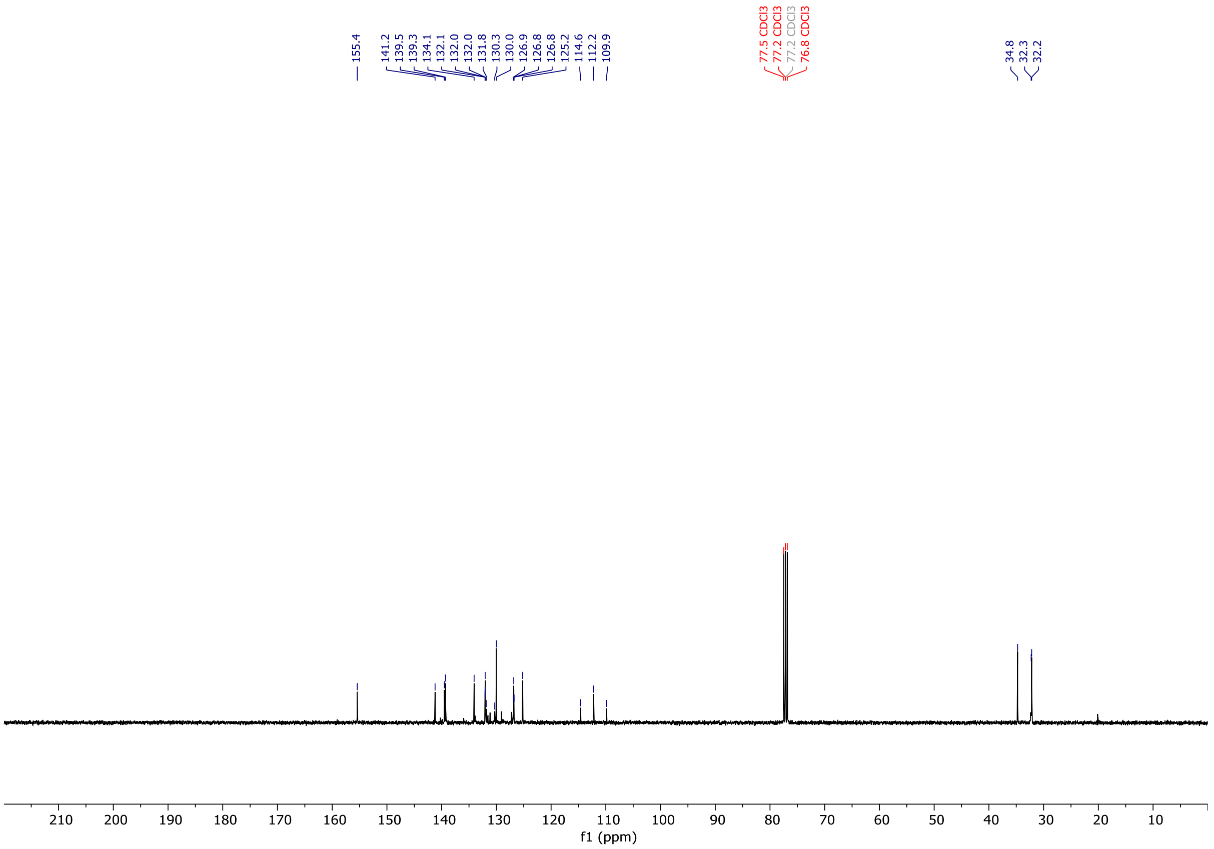

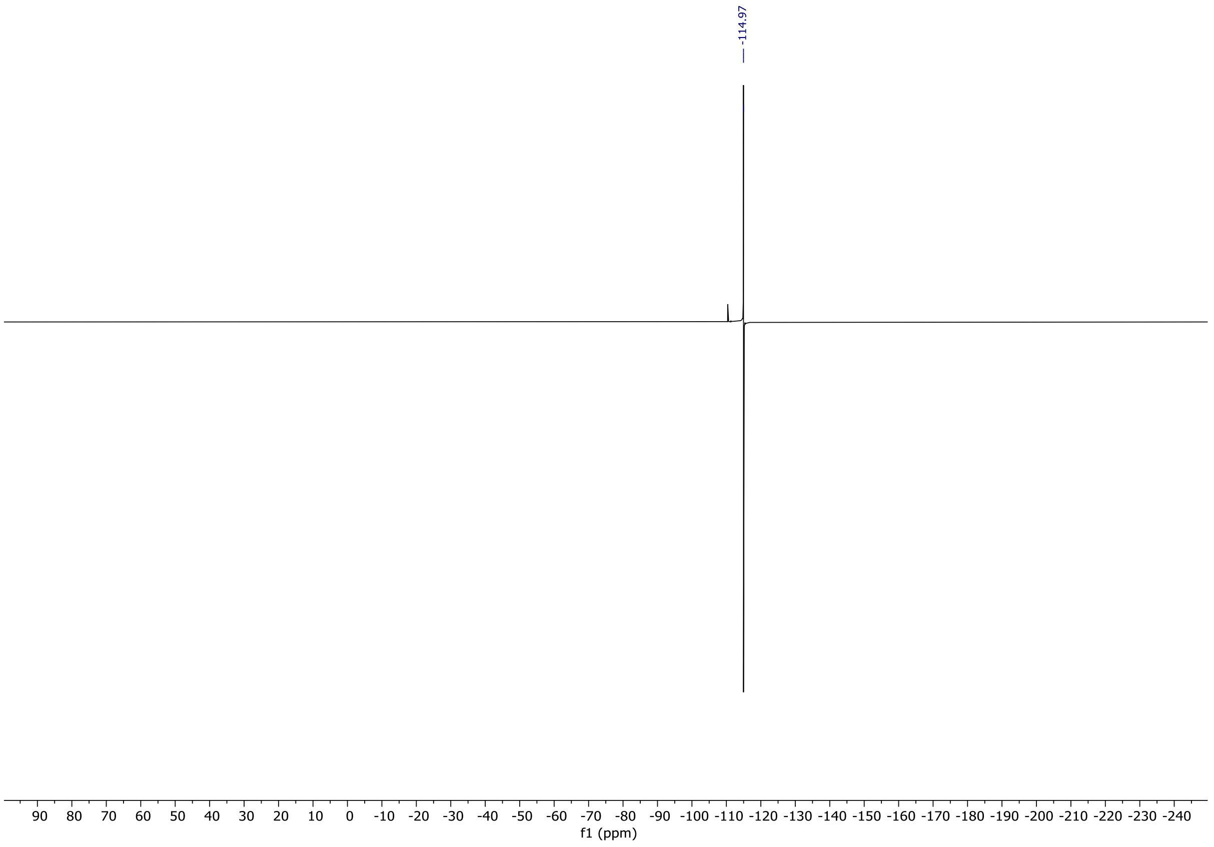
-114.97
90 80 70 60 50 40 30 20 10 0 -10 -20 -30 -40 -50 -60 -70 -80 -90 -100 -110 -120 -130 -140 -150 -160 -170 -180 -190 -200 -210 -220 -230 -240
f1 (ppm)

OX04959

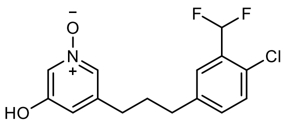

OX04959

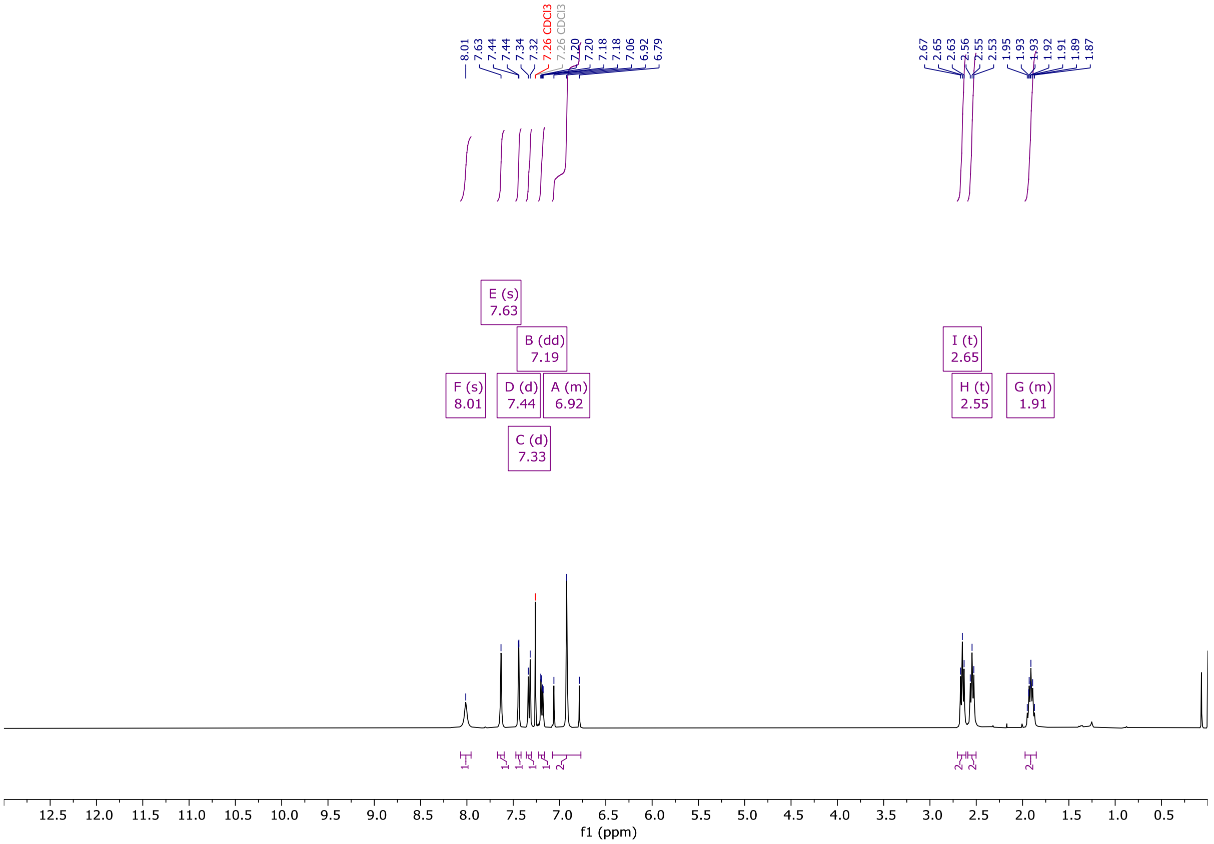

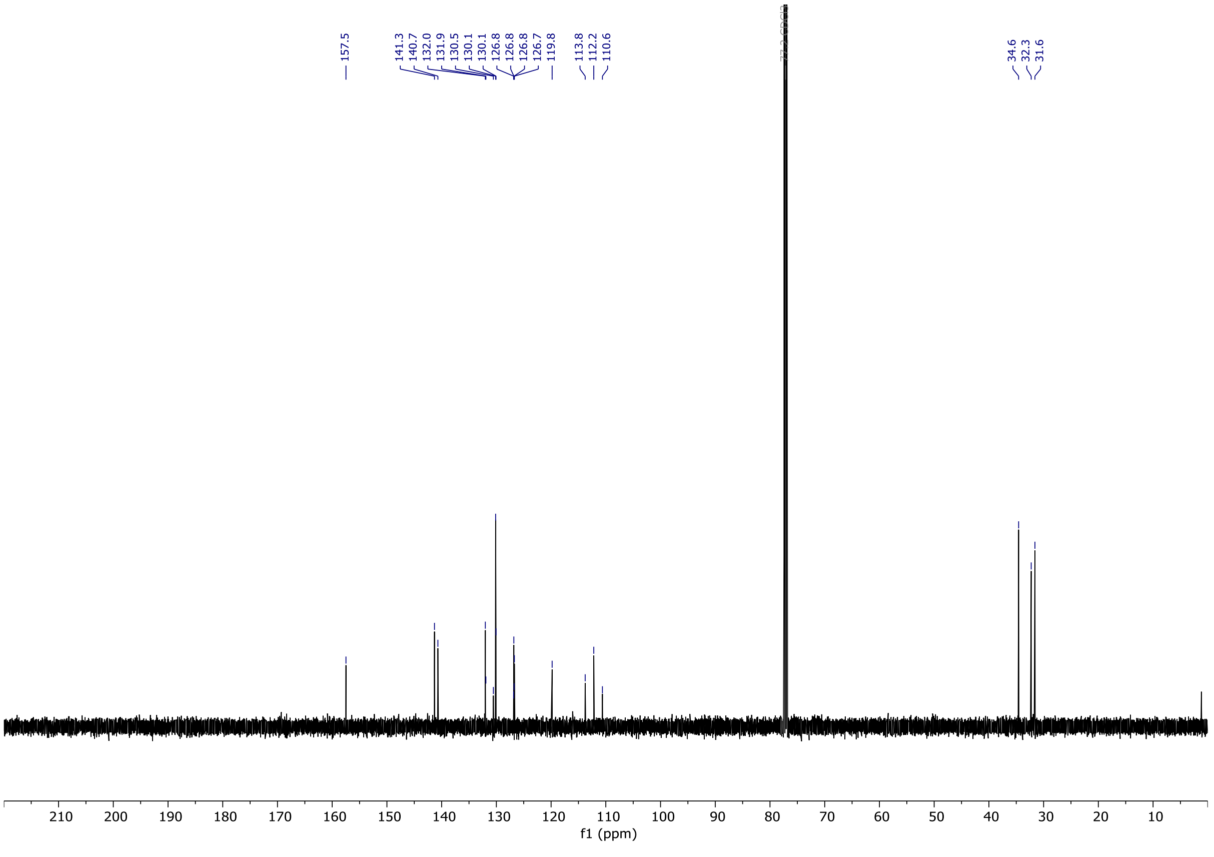

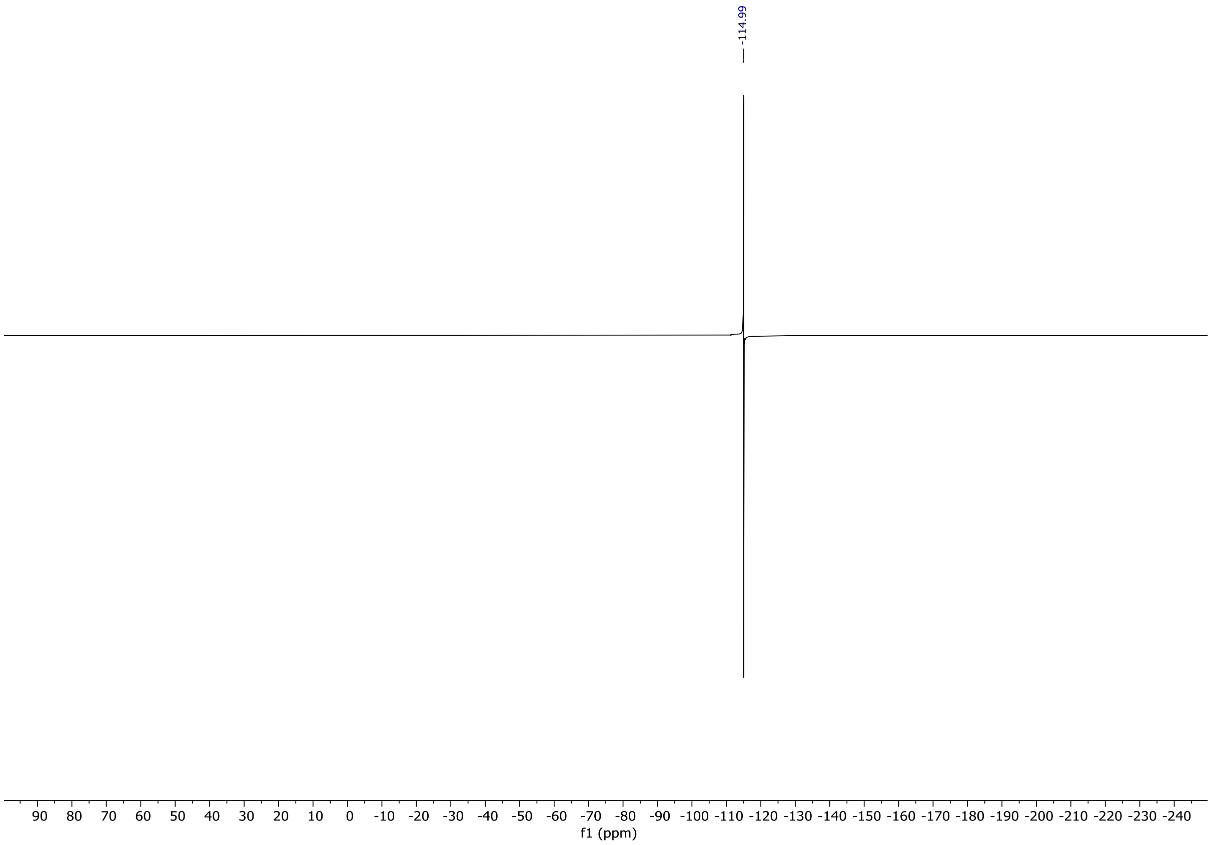
-114.99
90 80 70 60 50 40 30 20 10 0 -10 -20 -30 -40 -50 -60 -70 -80 -90 -100 -110 -120 -130 -140 -150 -160 -170 -180 -190 -200 -210 -220 -230 -240
f1 (ppm)

OX04960a

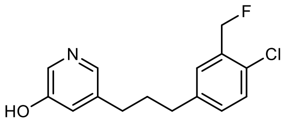

OX04960a

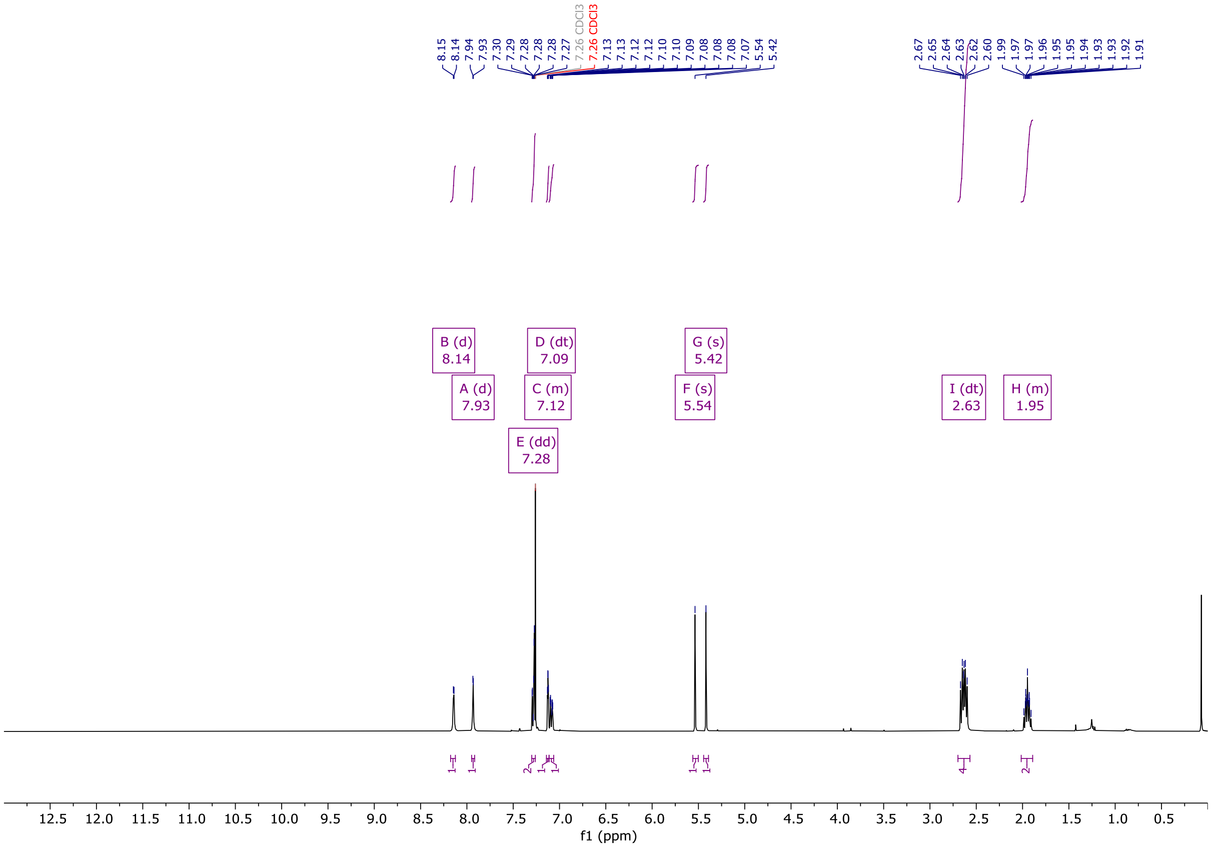

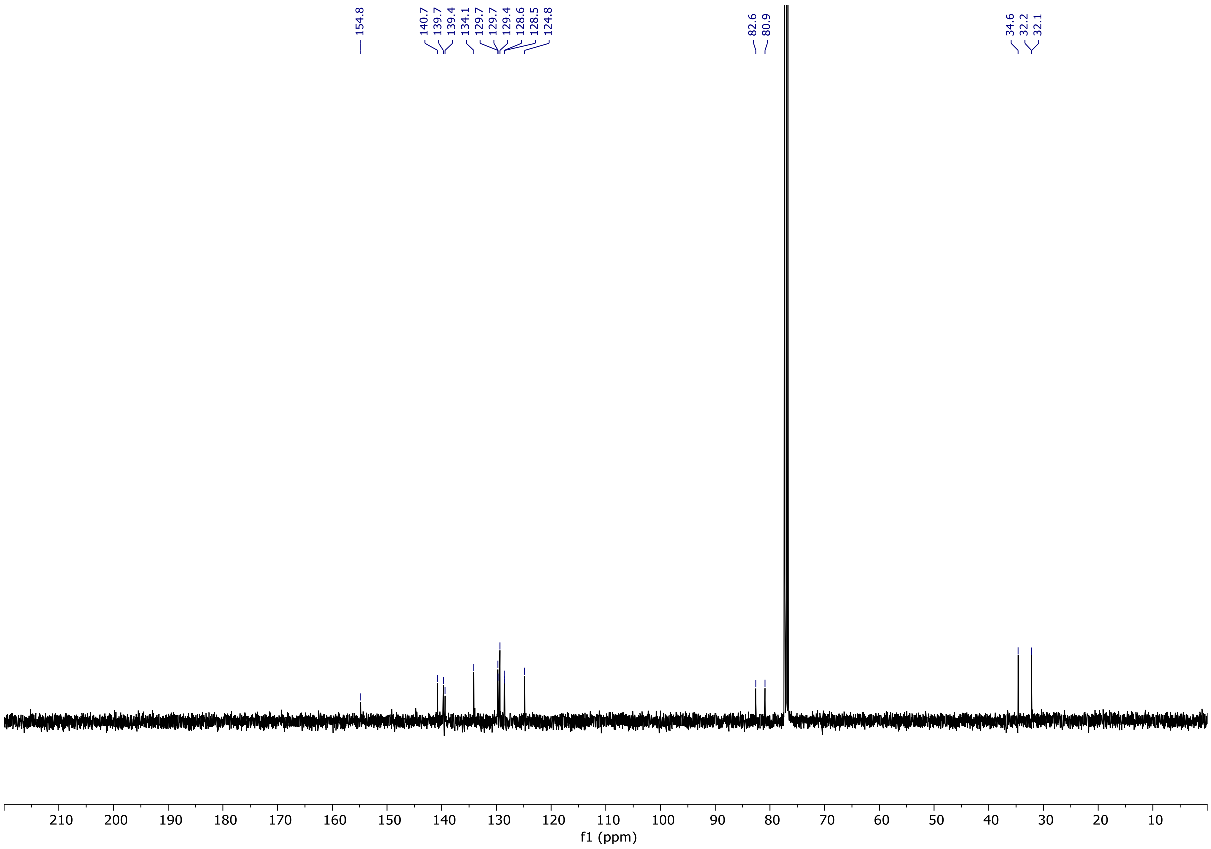

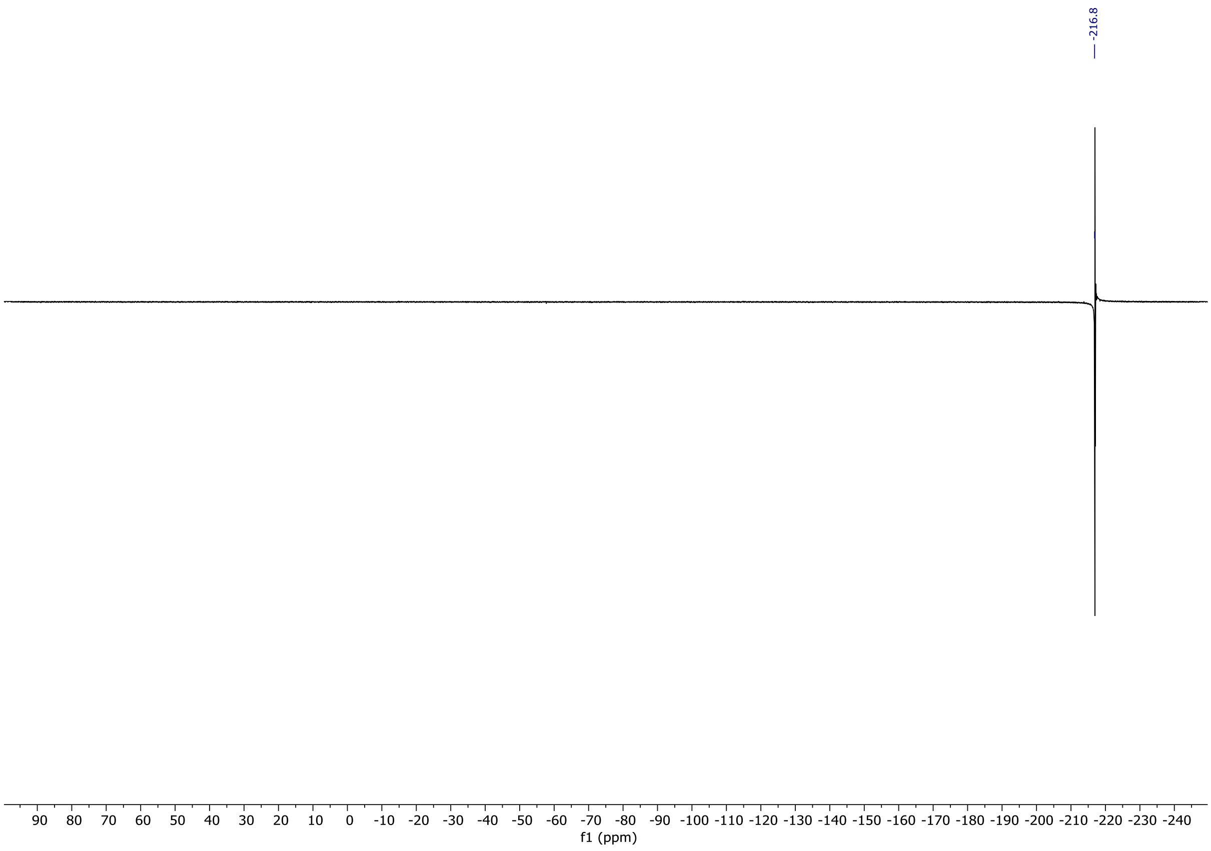

-216.8
90 80 70 60 50 40 30 20 10 0 -10 -20 -30 -40 -50 -60 -70 -80 -90 -100 -110 -120 -130 -140 -150 -160 -170 -180 -190 -200 -210 -220 -230 -240
f1 (ppm)

OX04960

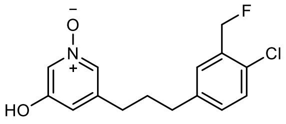

OX04960

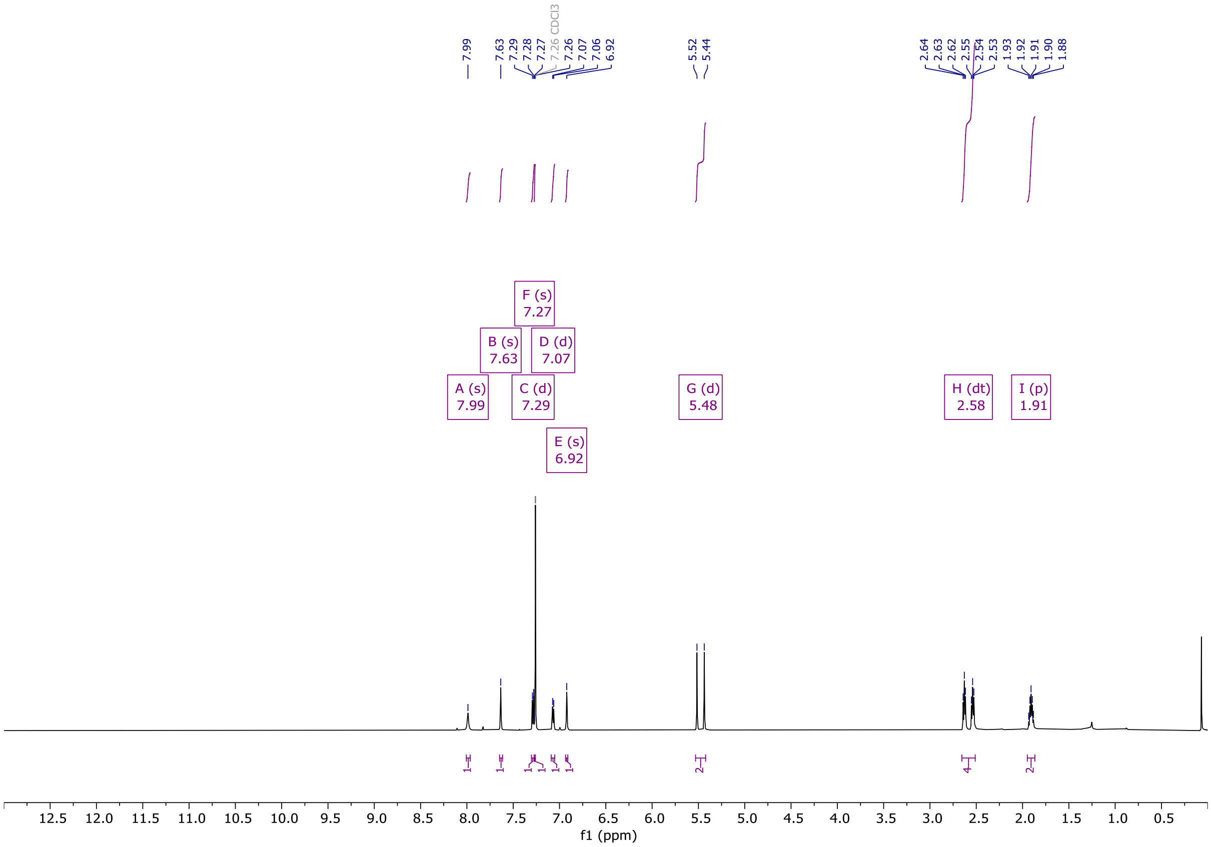

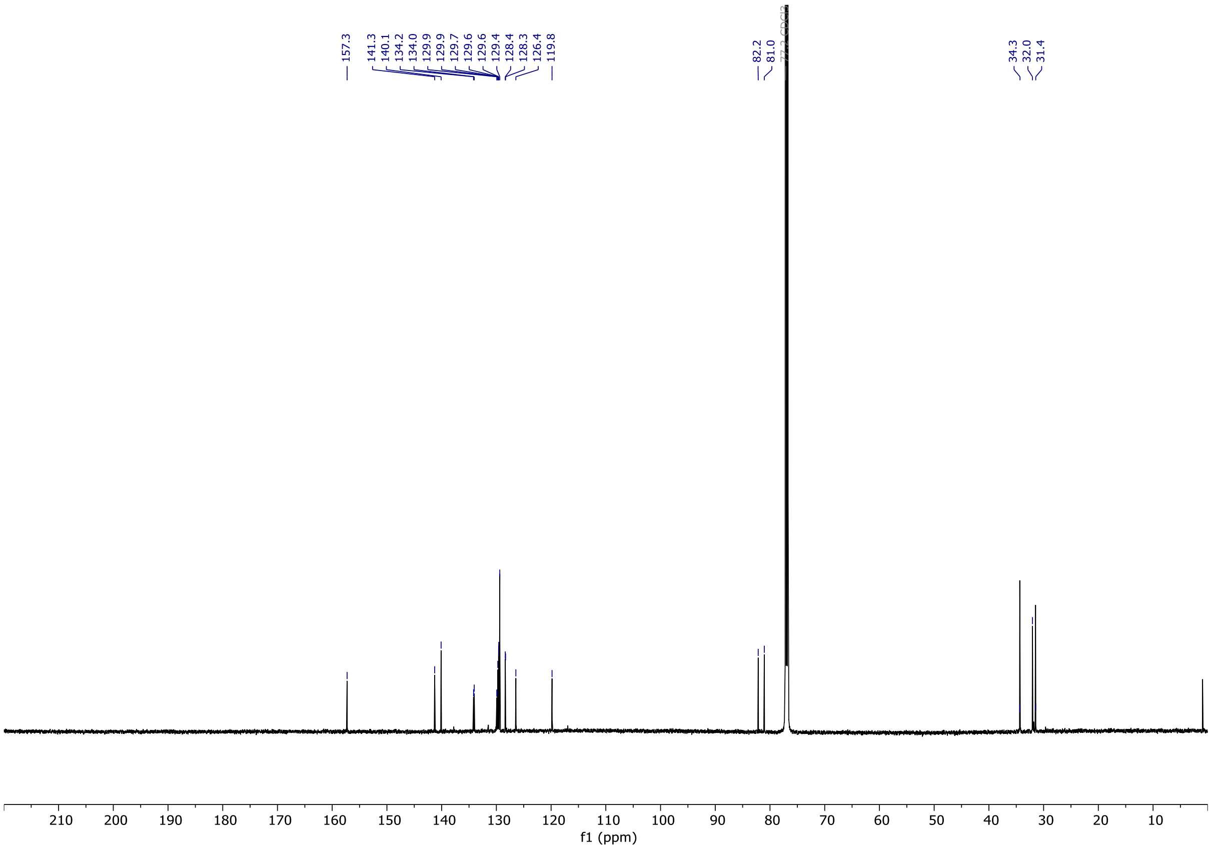

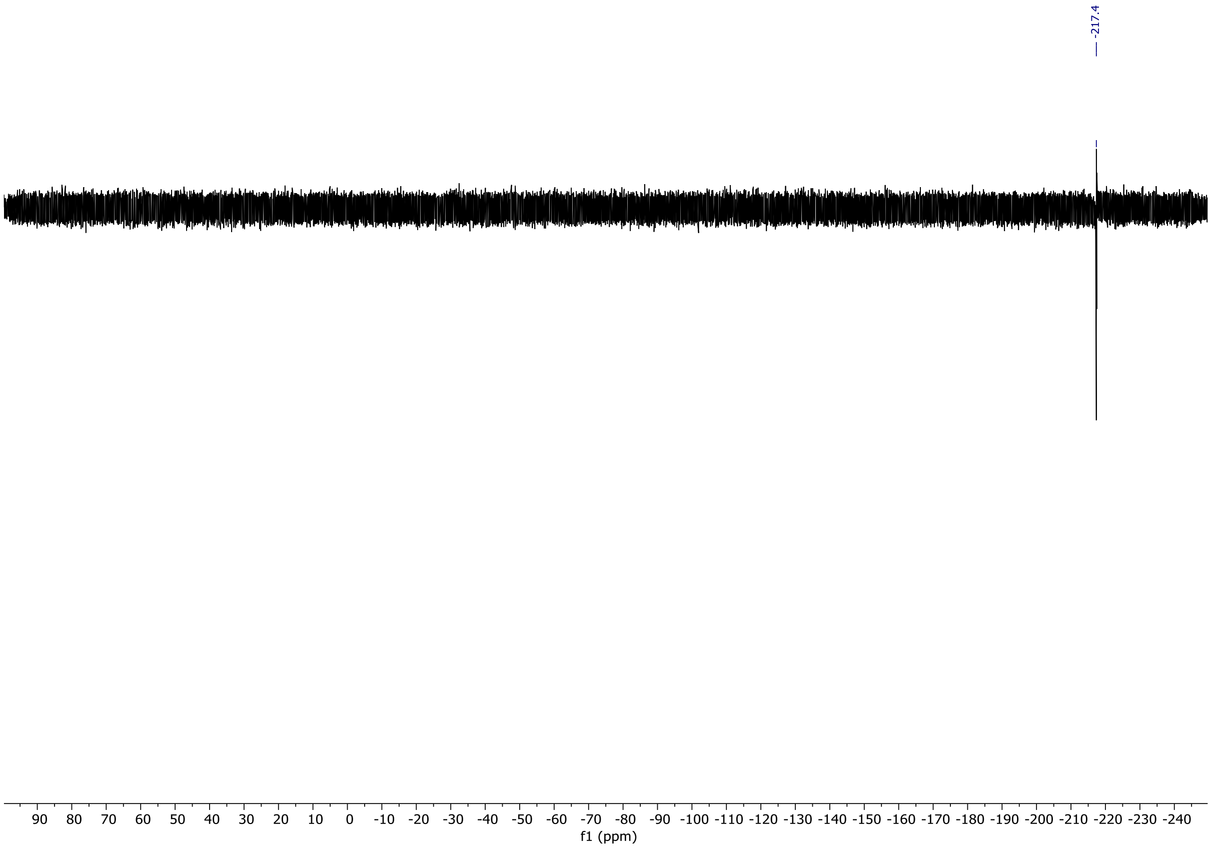

-217.4
90 80 70 60 50 40 30 20 10 0 -10 -20 -30 -40 -50 -60 -70 -80 -90 -100 -110 -120 -130 -140 -150 -160 -170 -180 -190 -200 -210 -220 -230 -240
f1 (ppm)

OX04961a

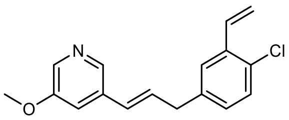

OX04961a

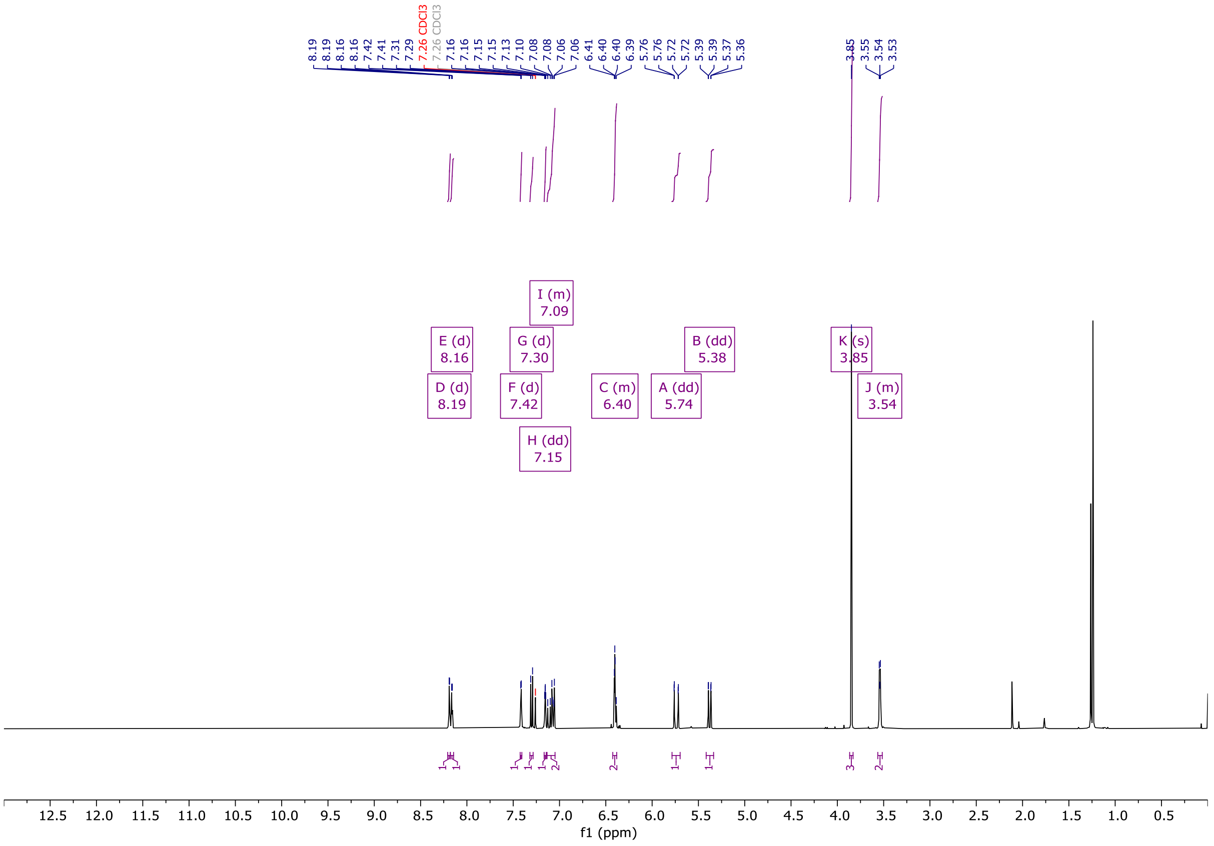

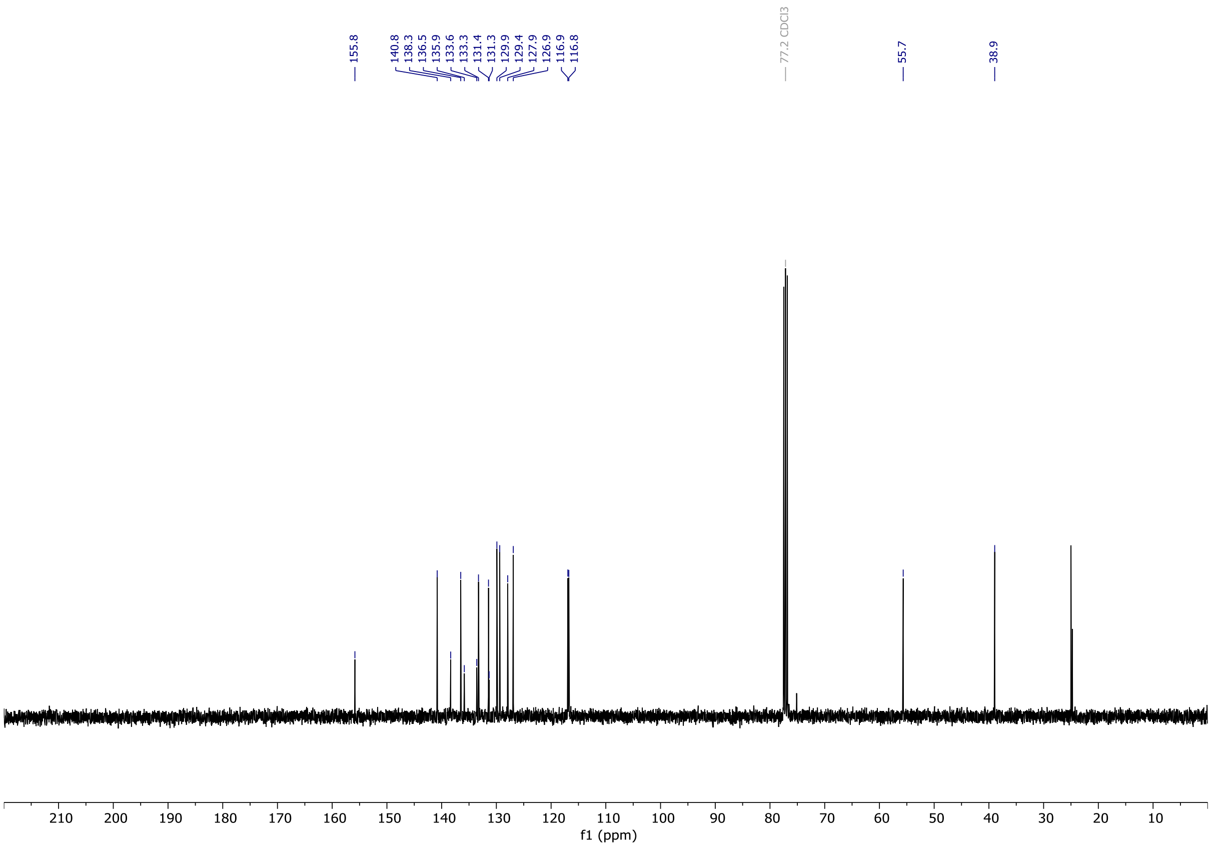

OX04961b

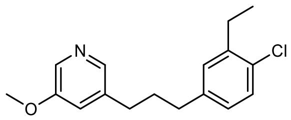

OX04961b

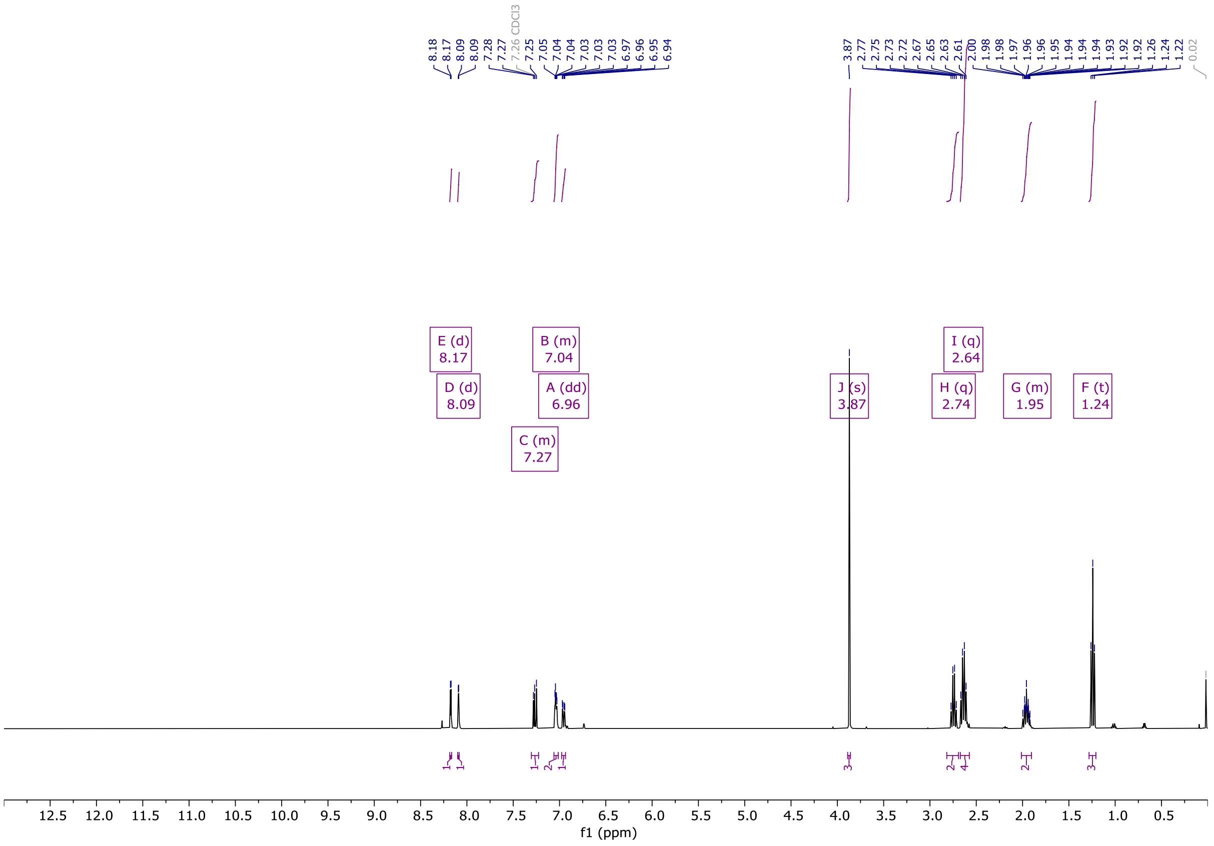

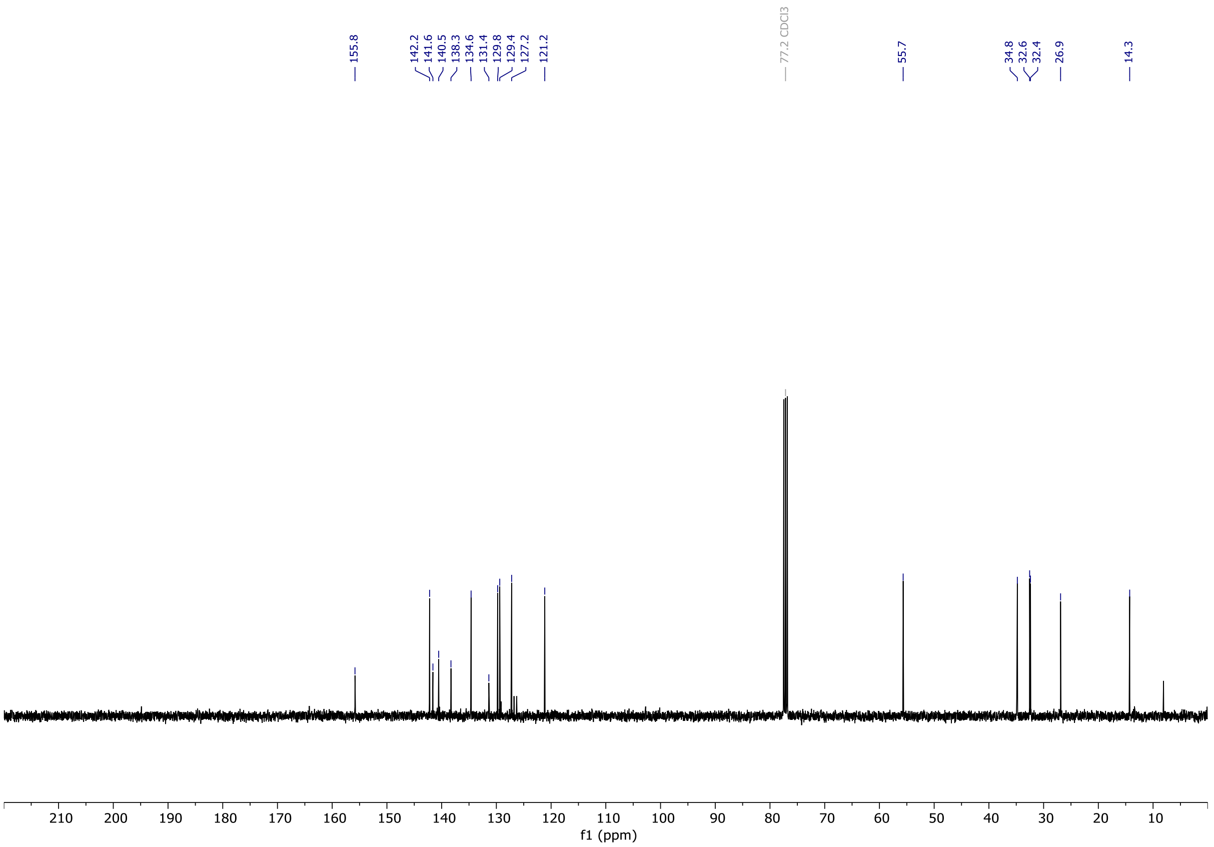

OX04961c

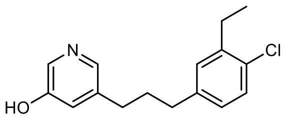

OX04961c

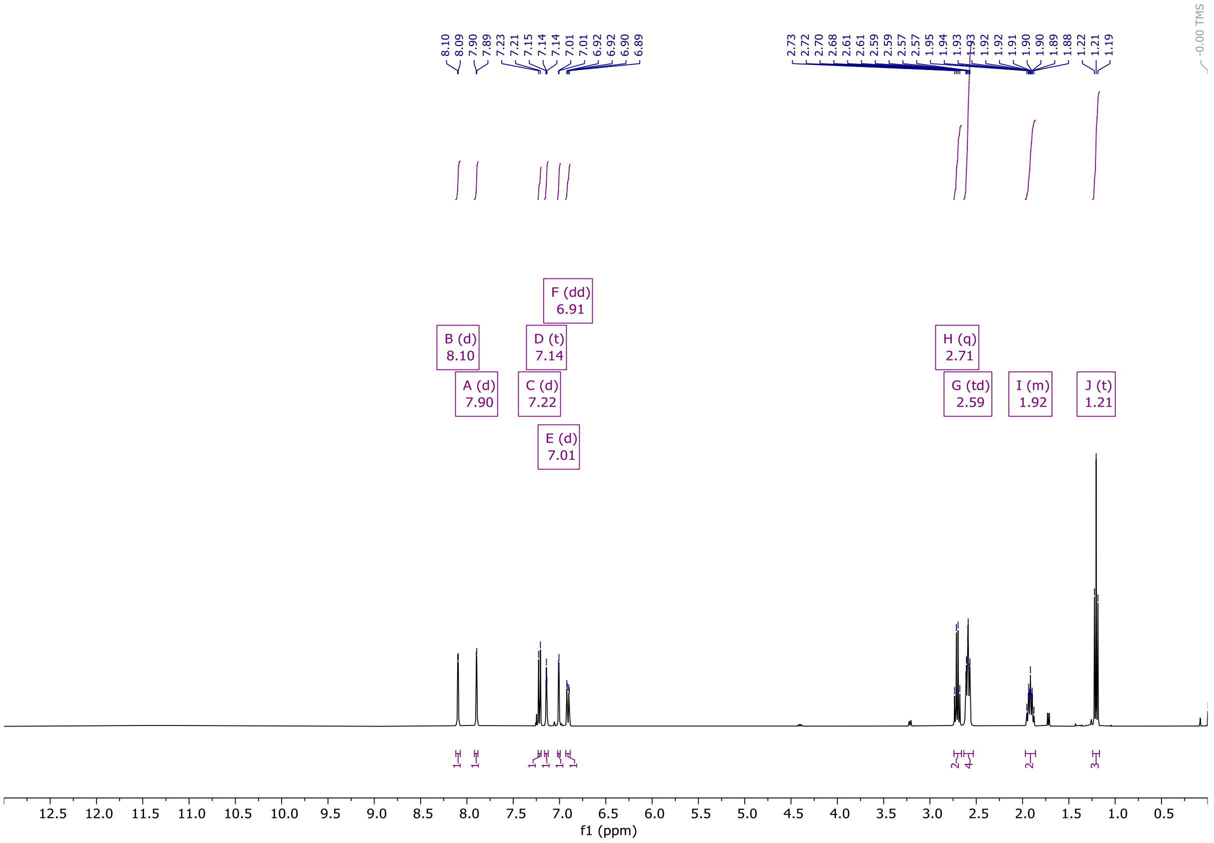

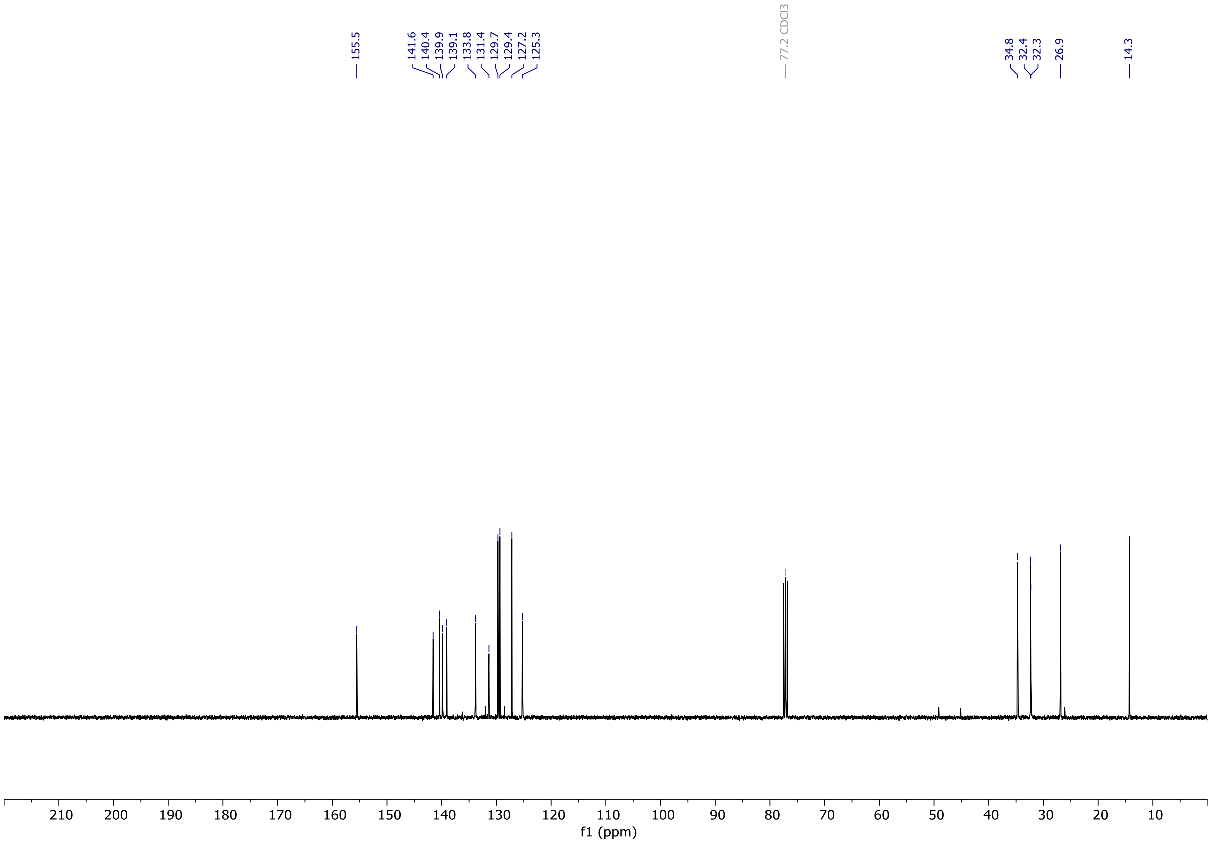

OX04961

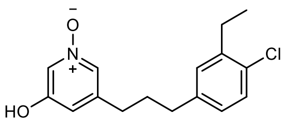

OX04961

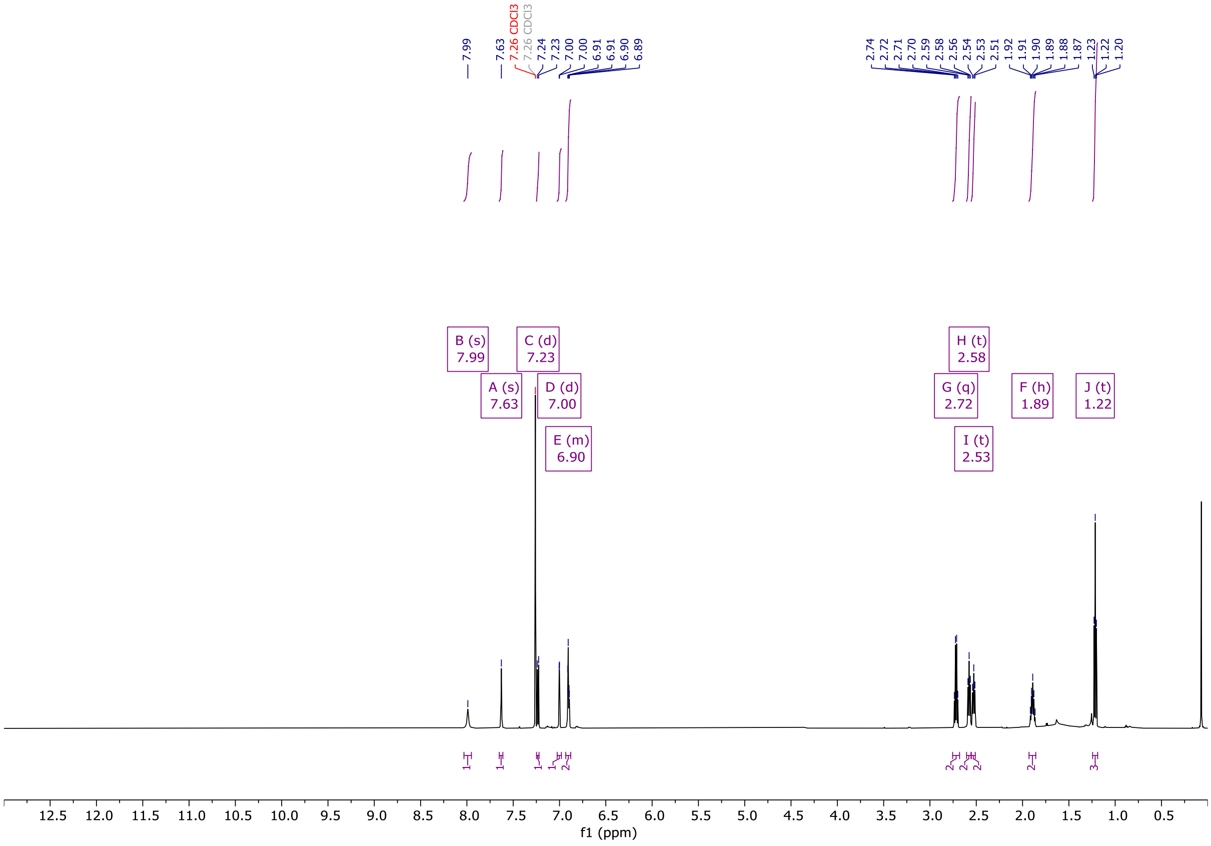

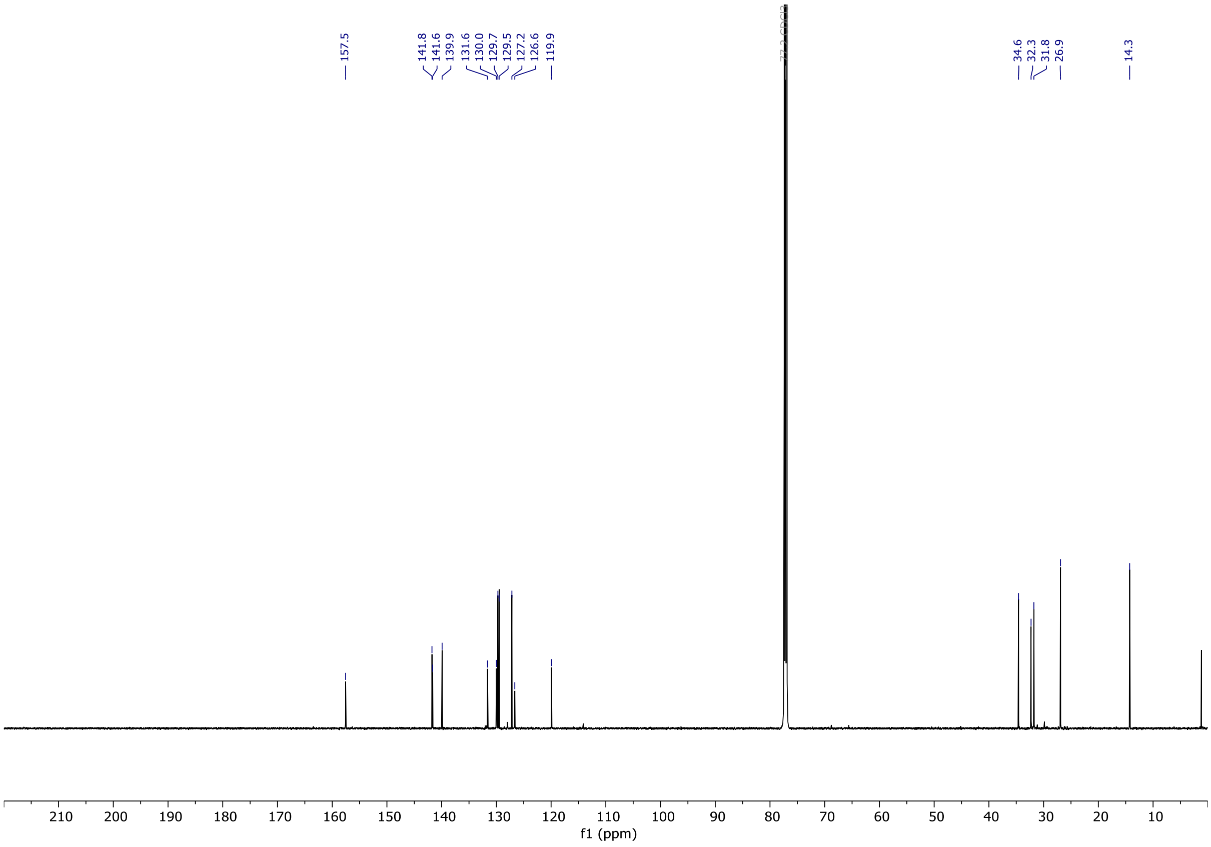

OX04962a

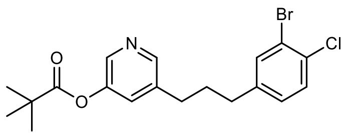

OX04962a

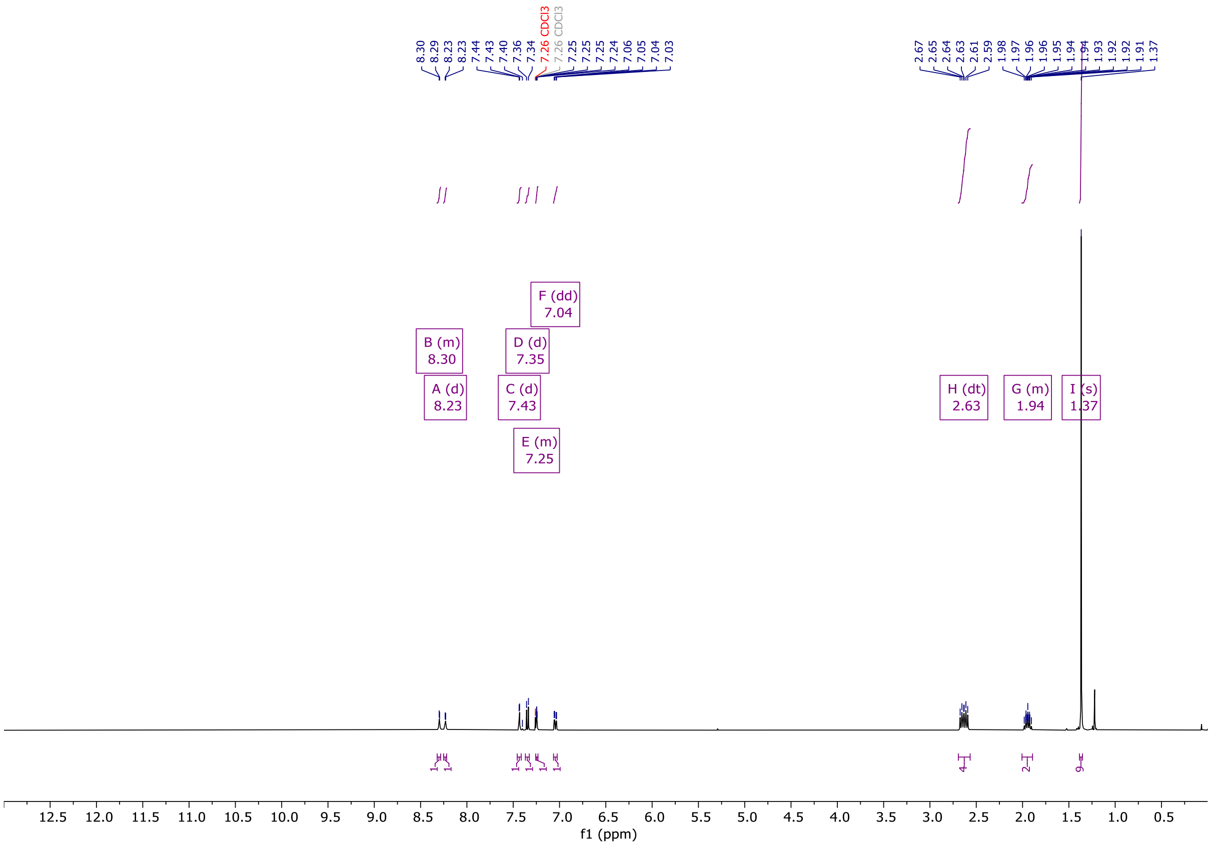

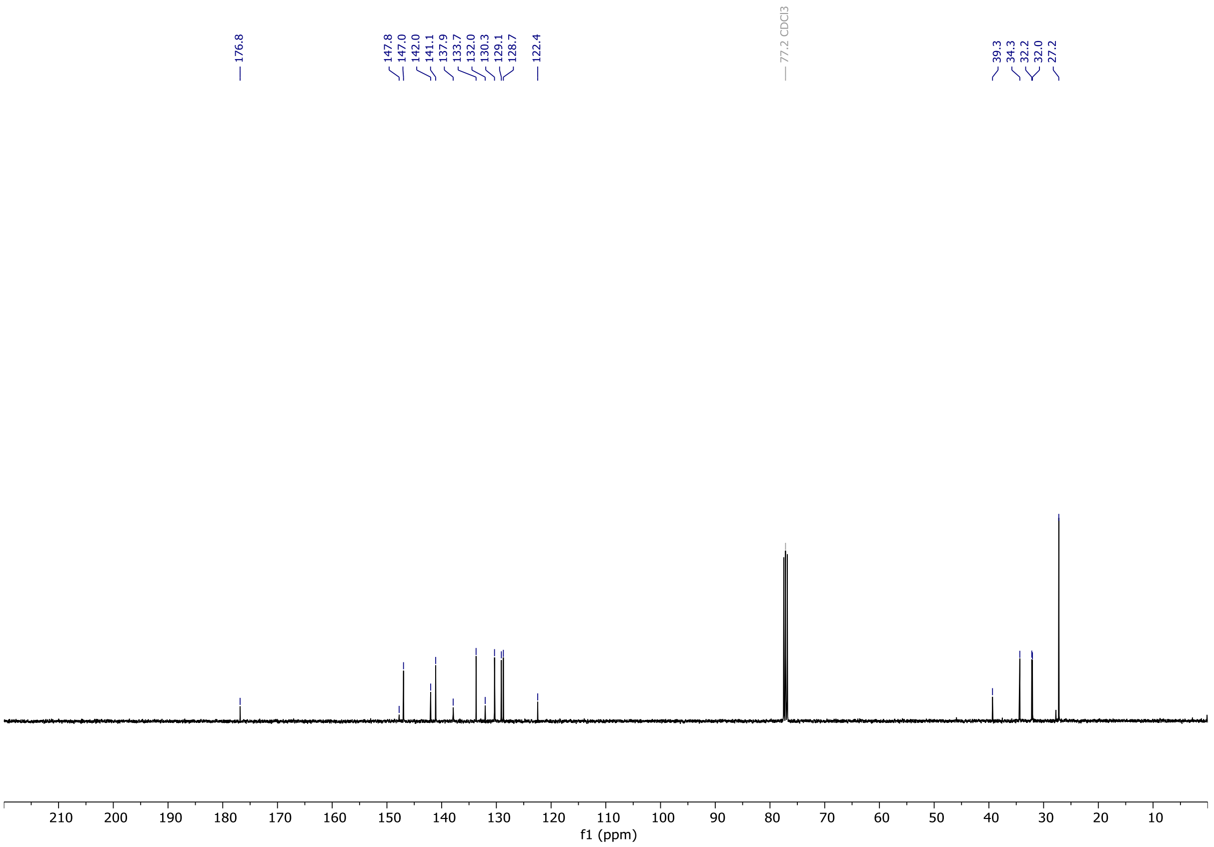

OX04962b

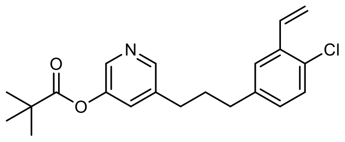

OX04962b

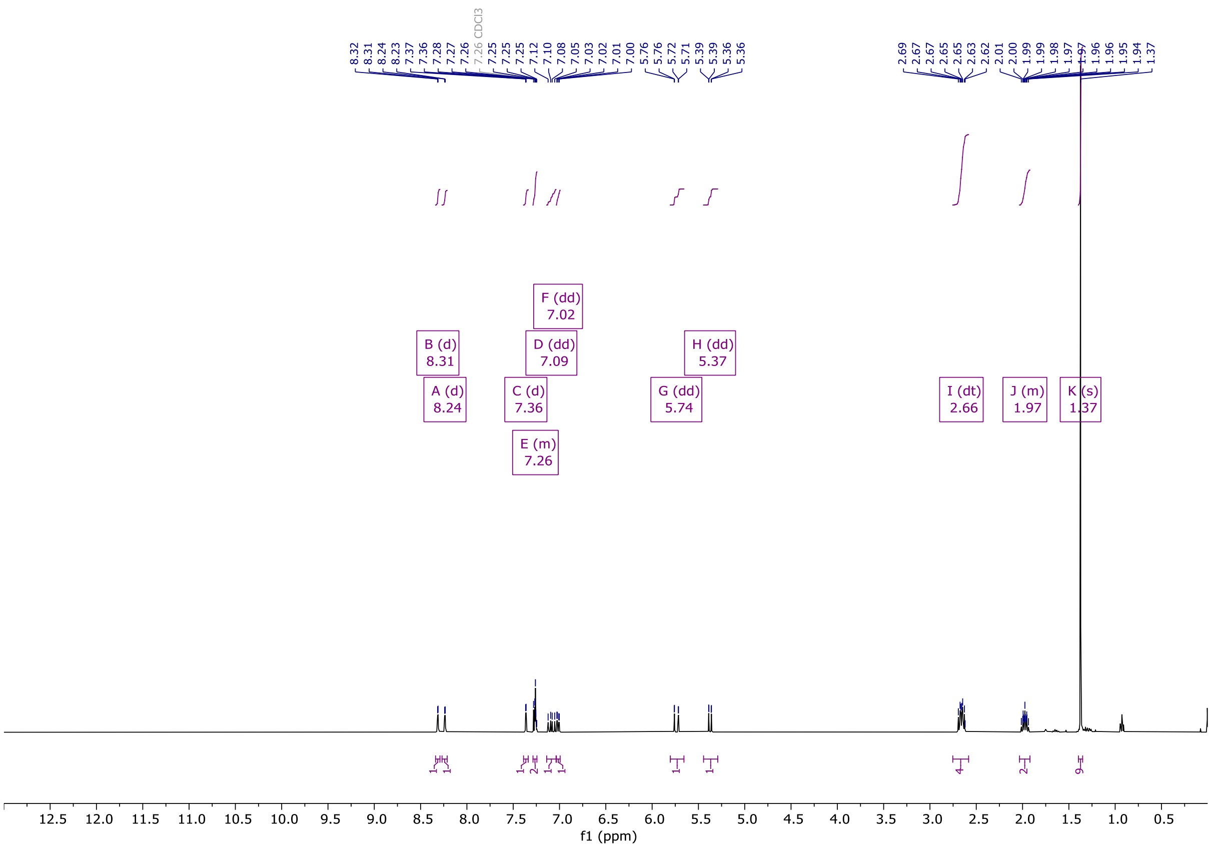

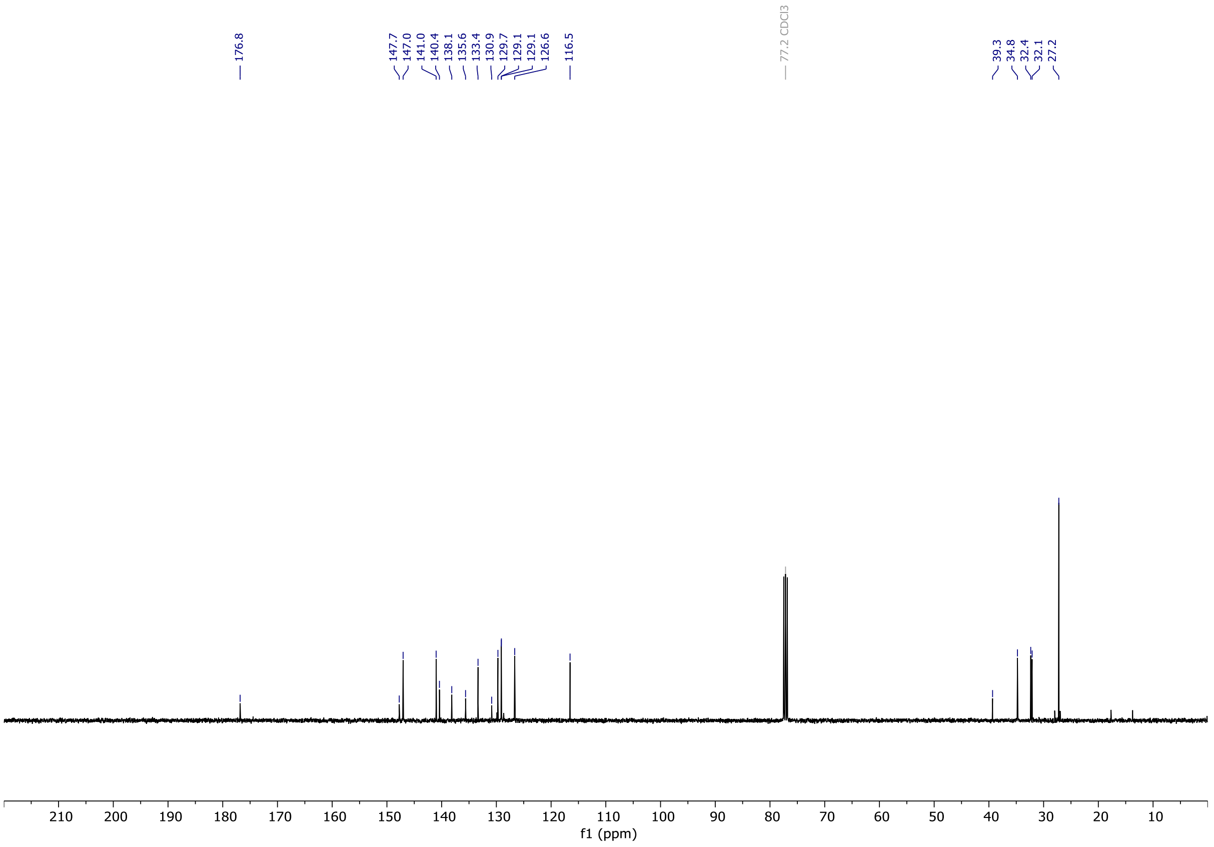

OX04962

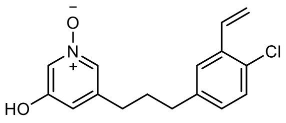

OX04962

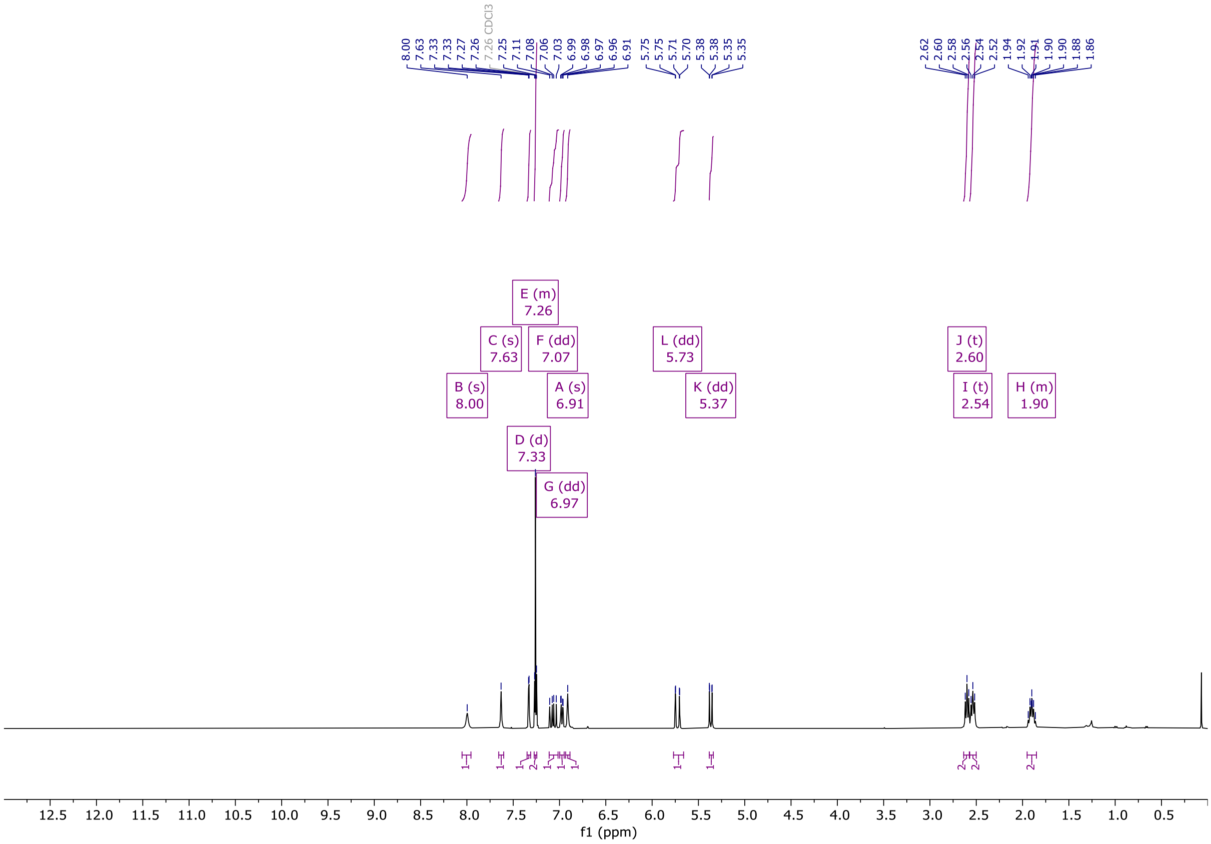

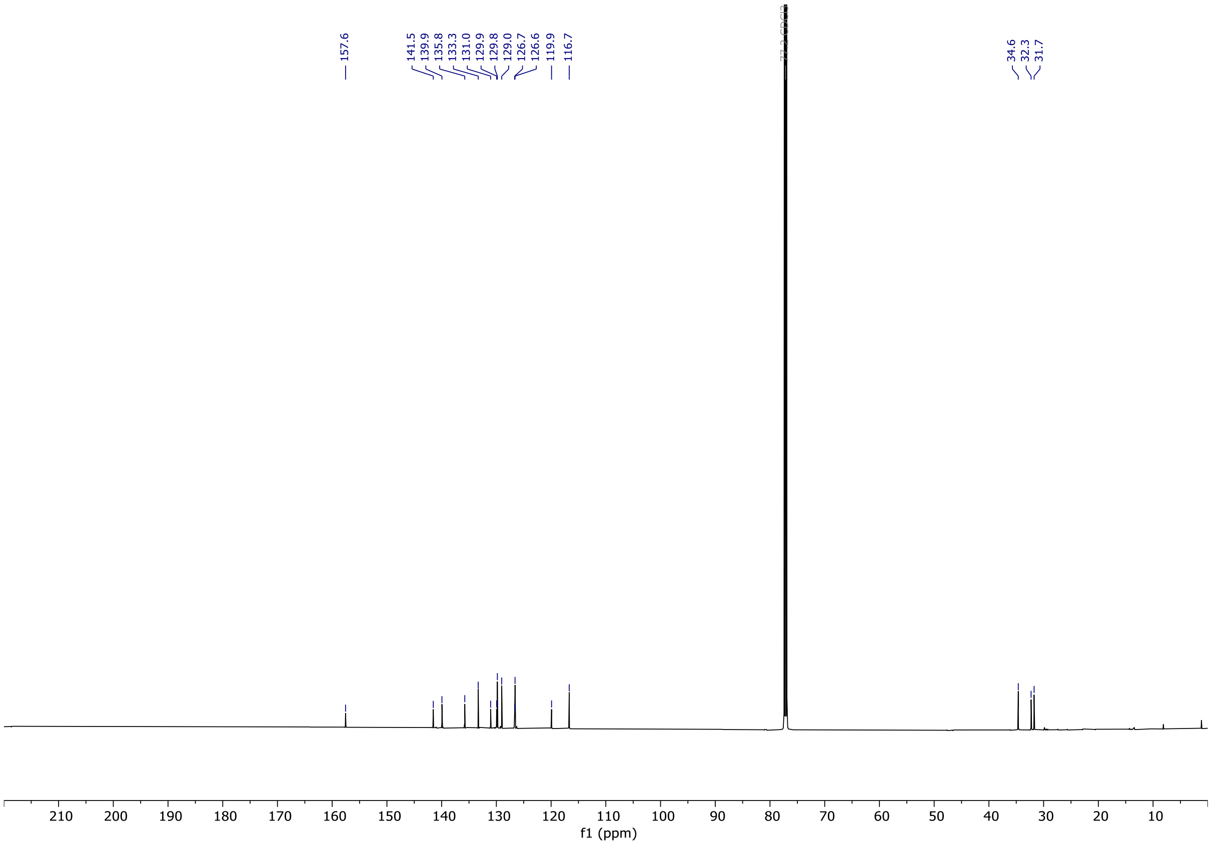

OX04963a

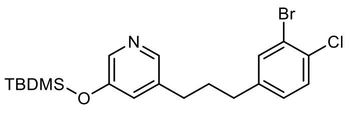

OX04963a

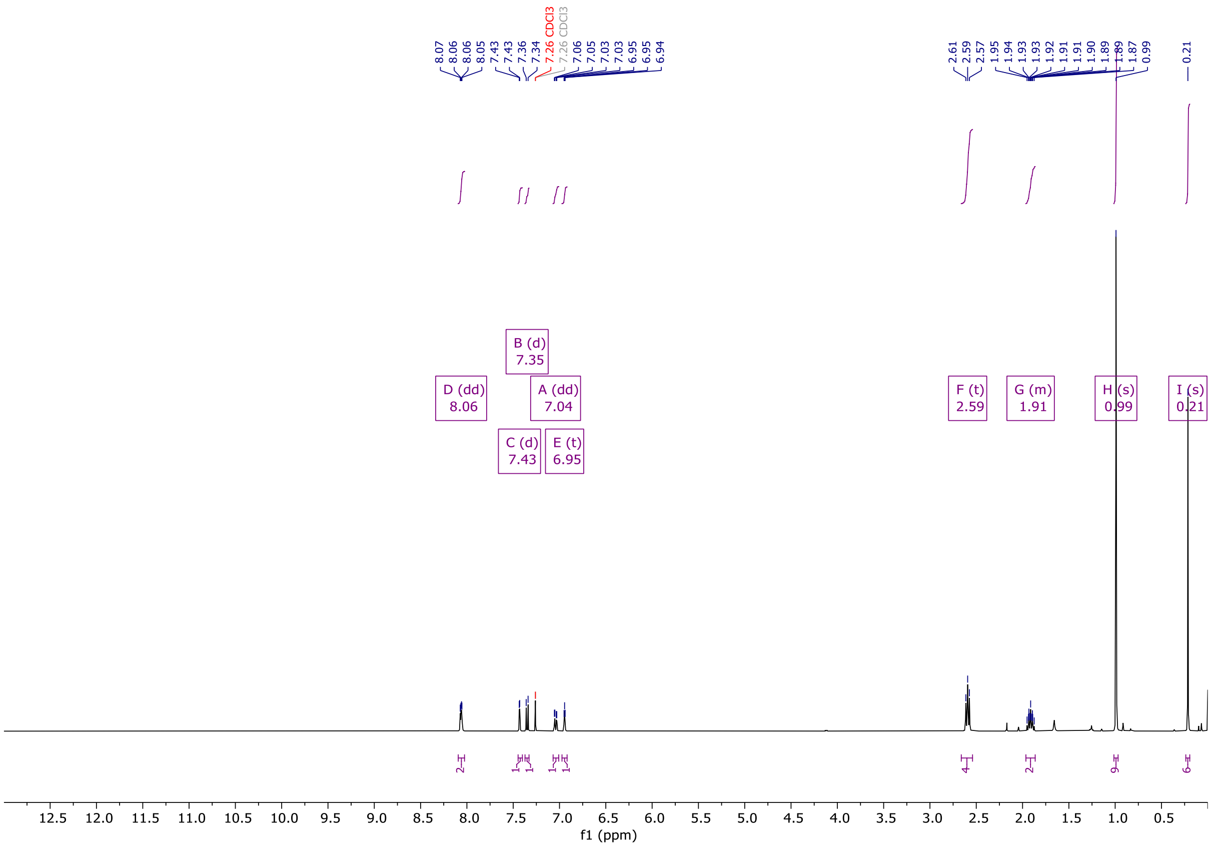

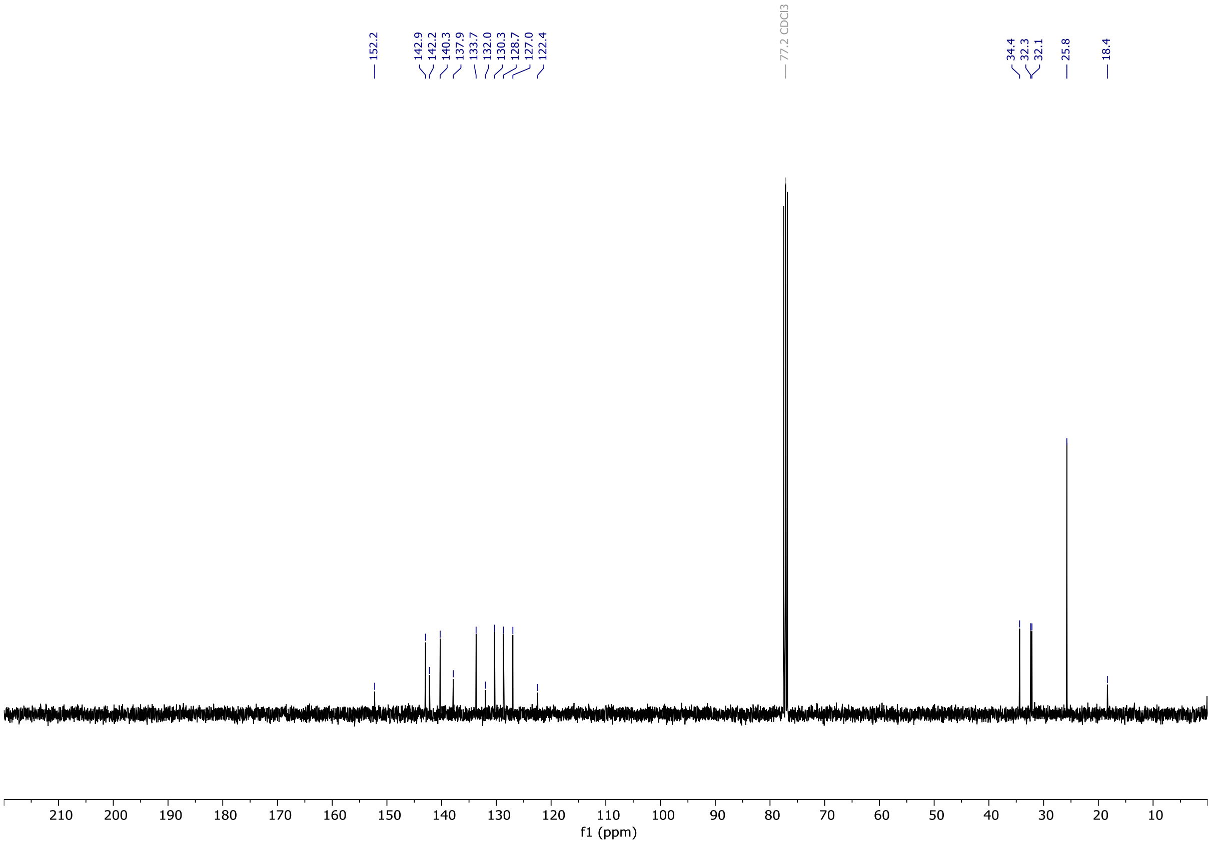

OX04963b

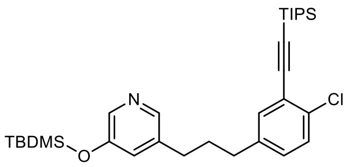

OX04963b

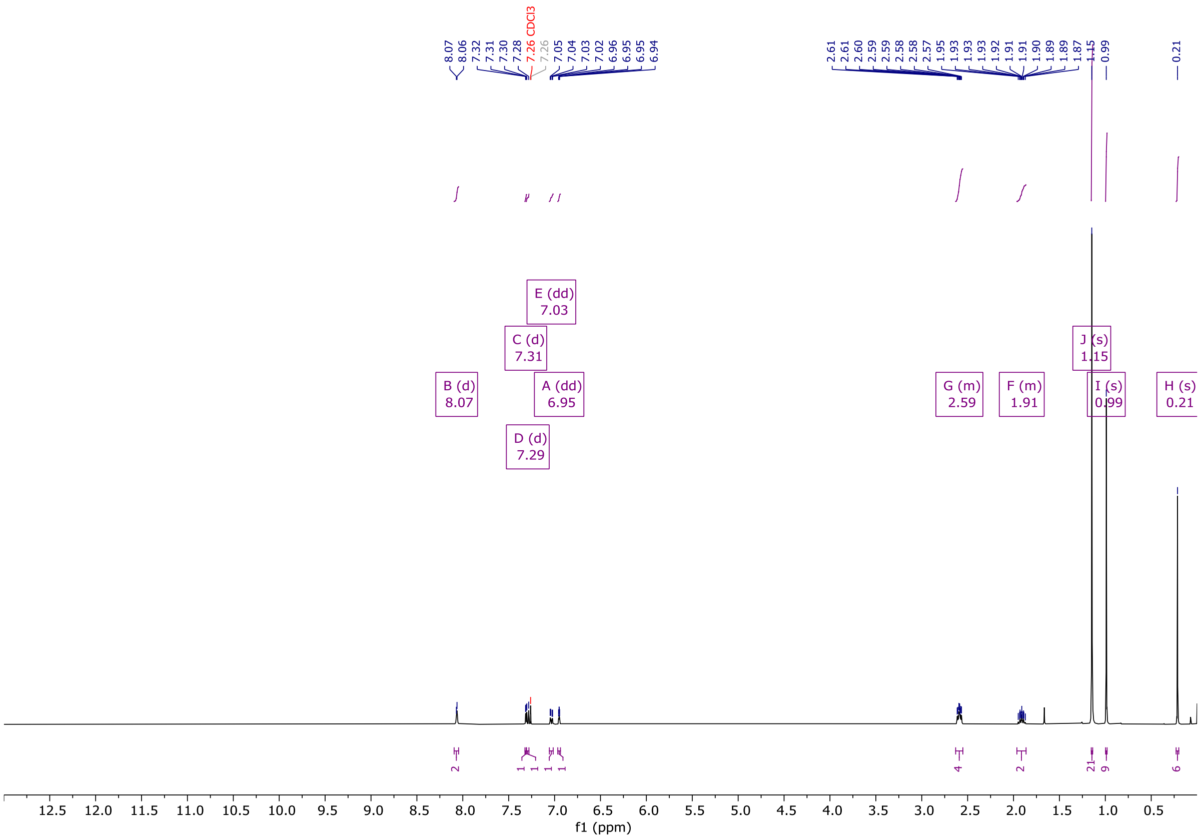

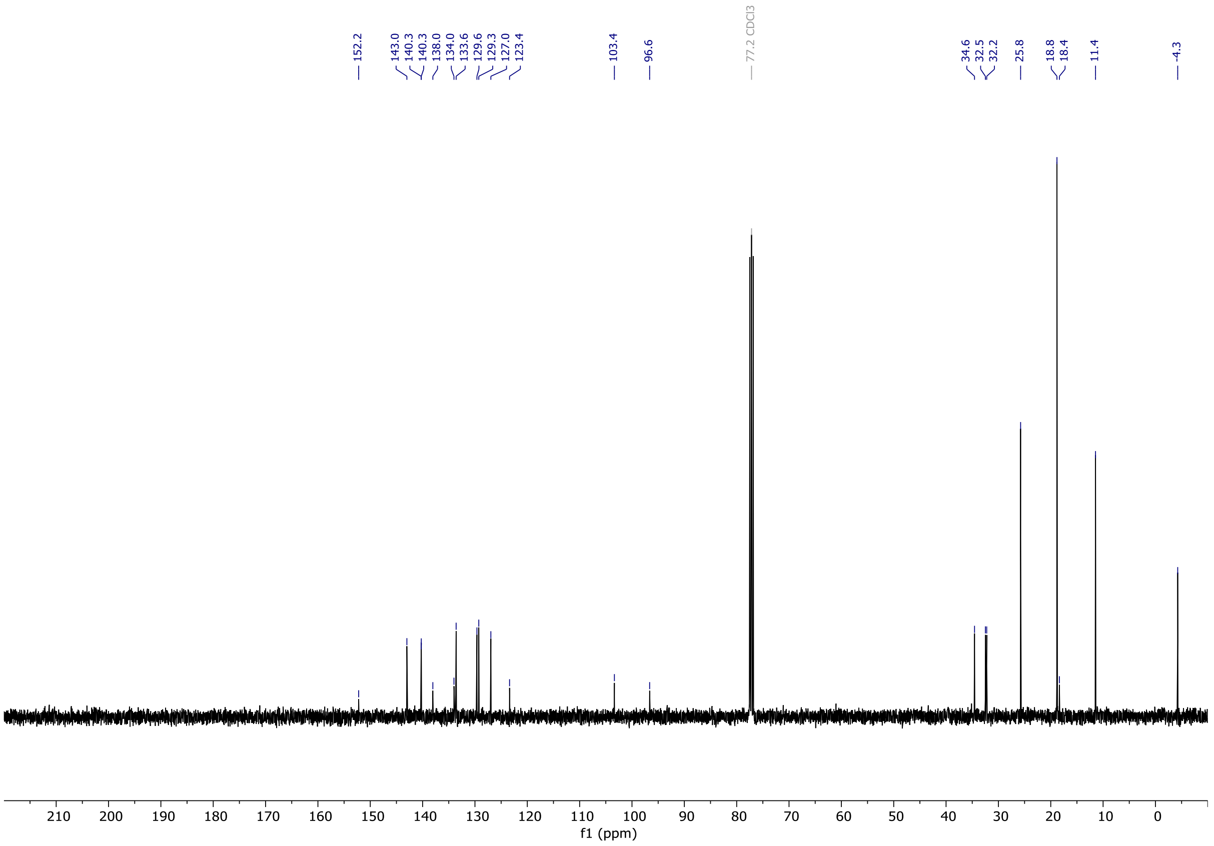

OX04963

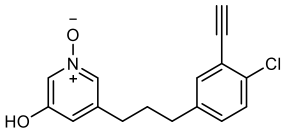

OX04963

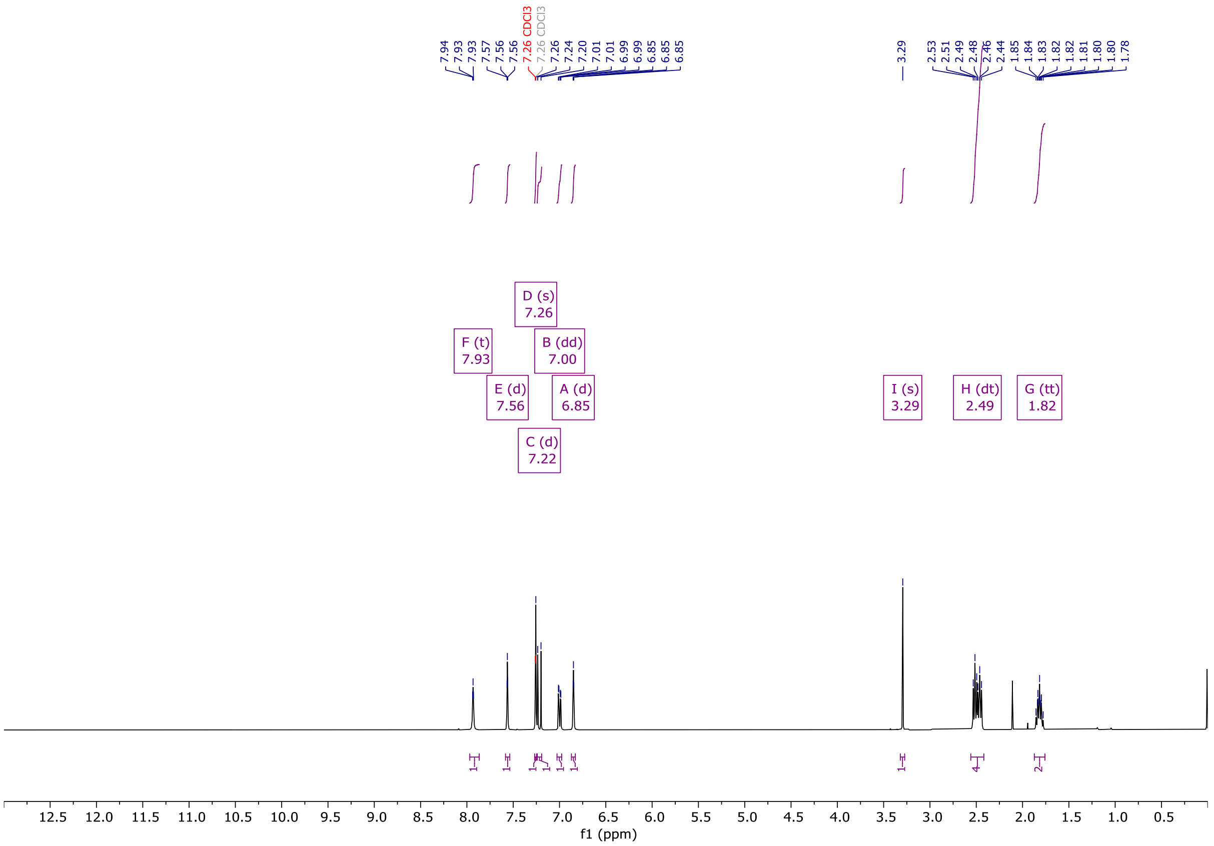

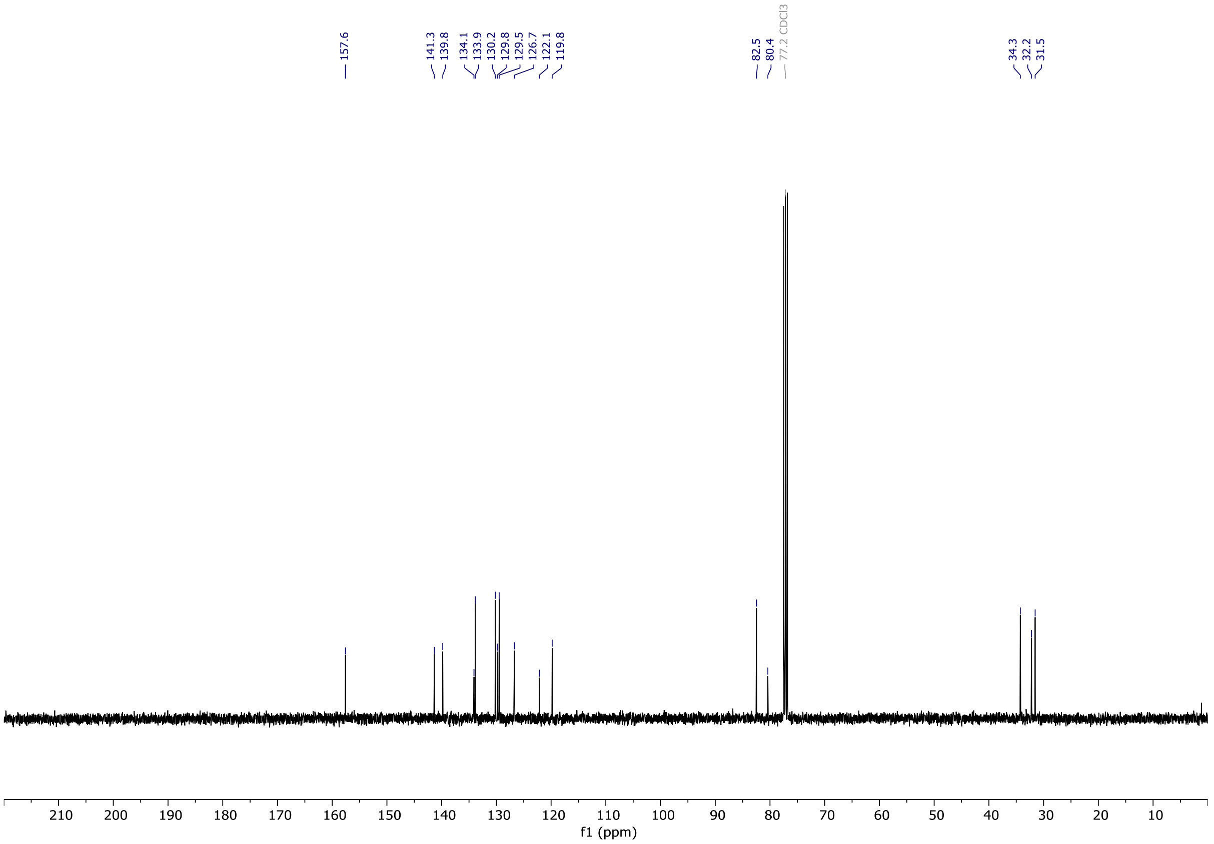

OX04964a

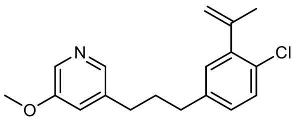

OX04964a

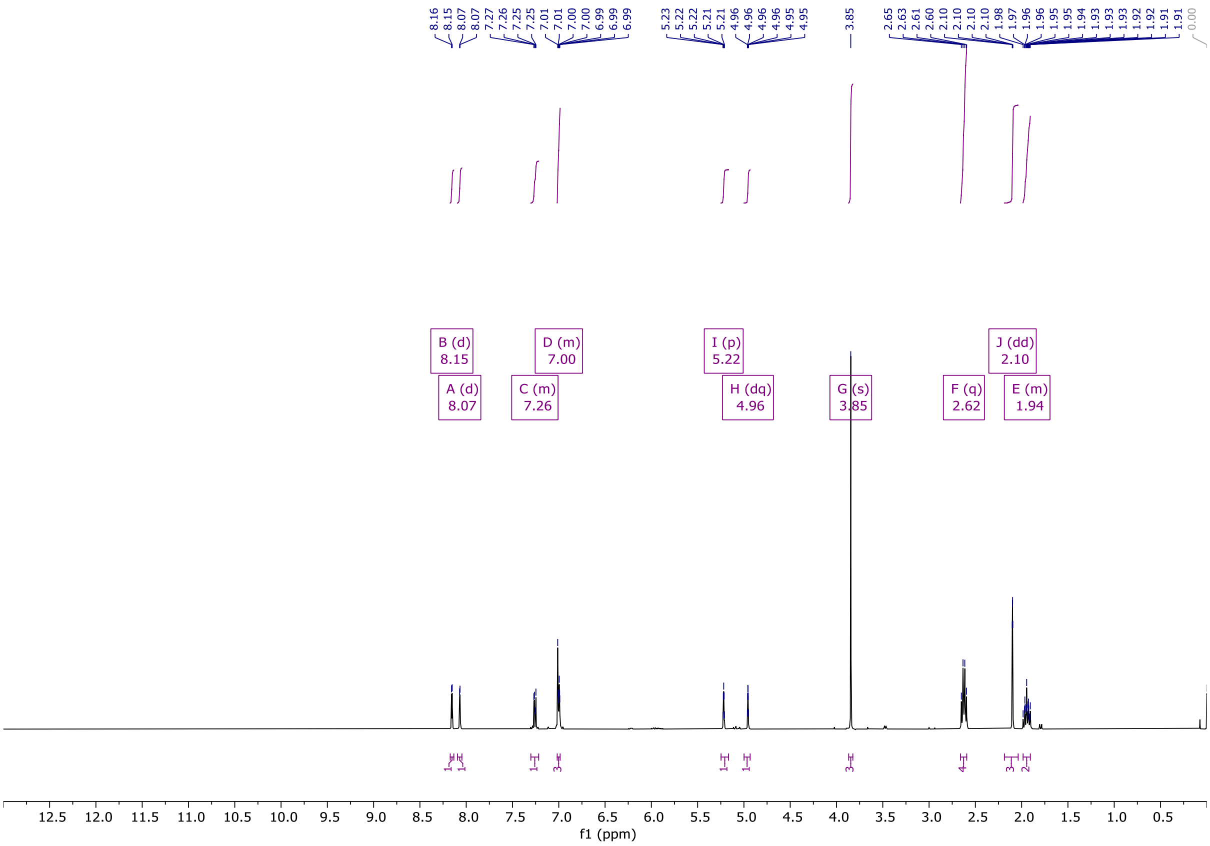

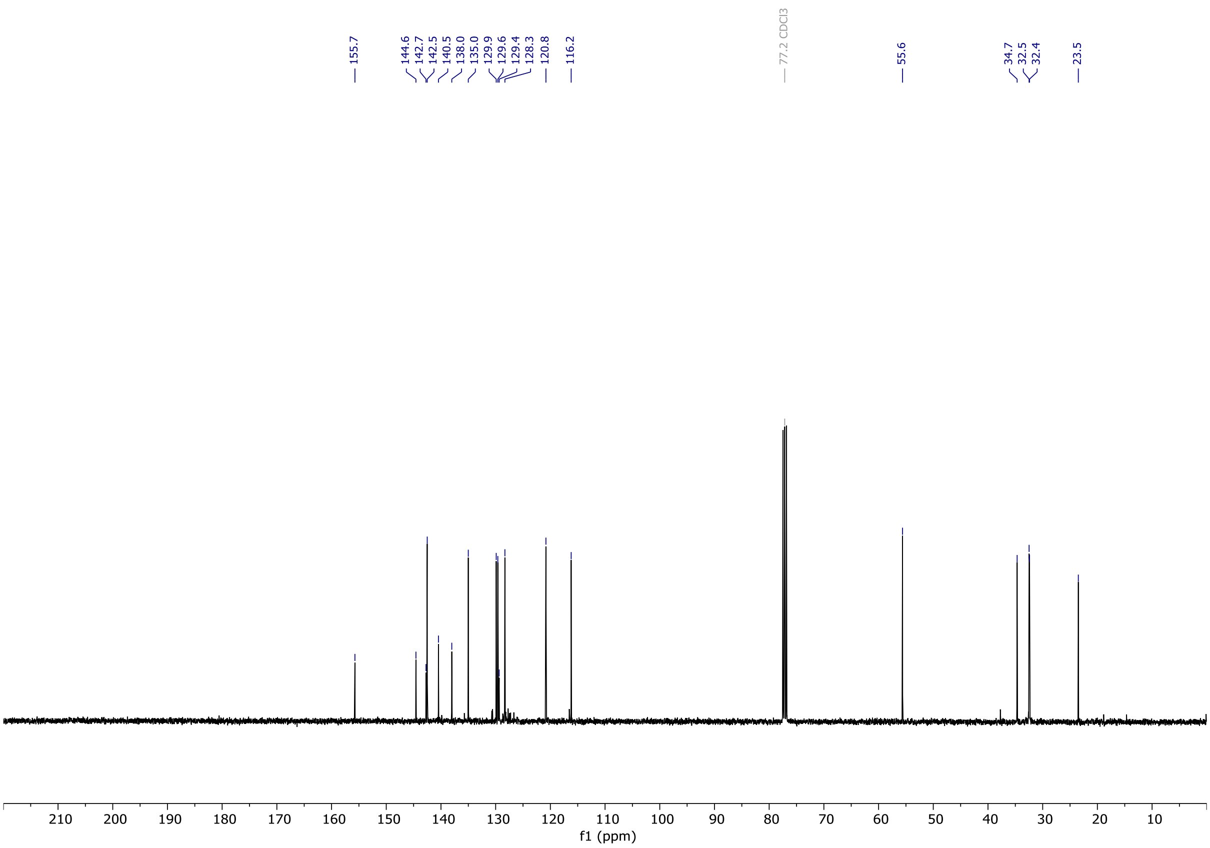

OX04964b

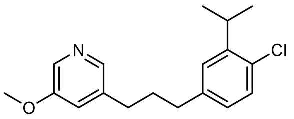

OX04964b

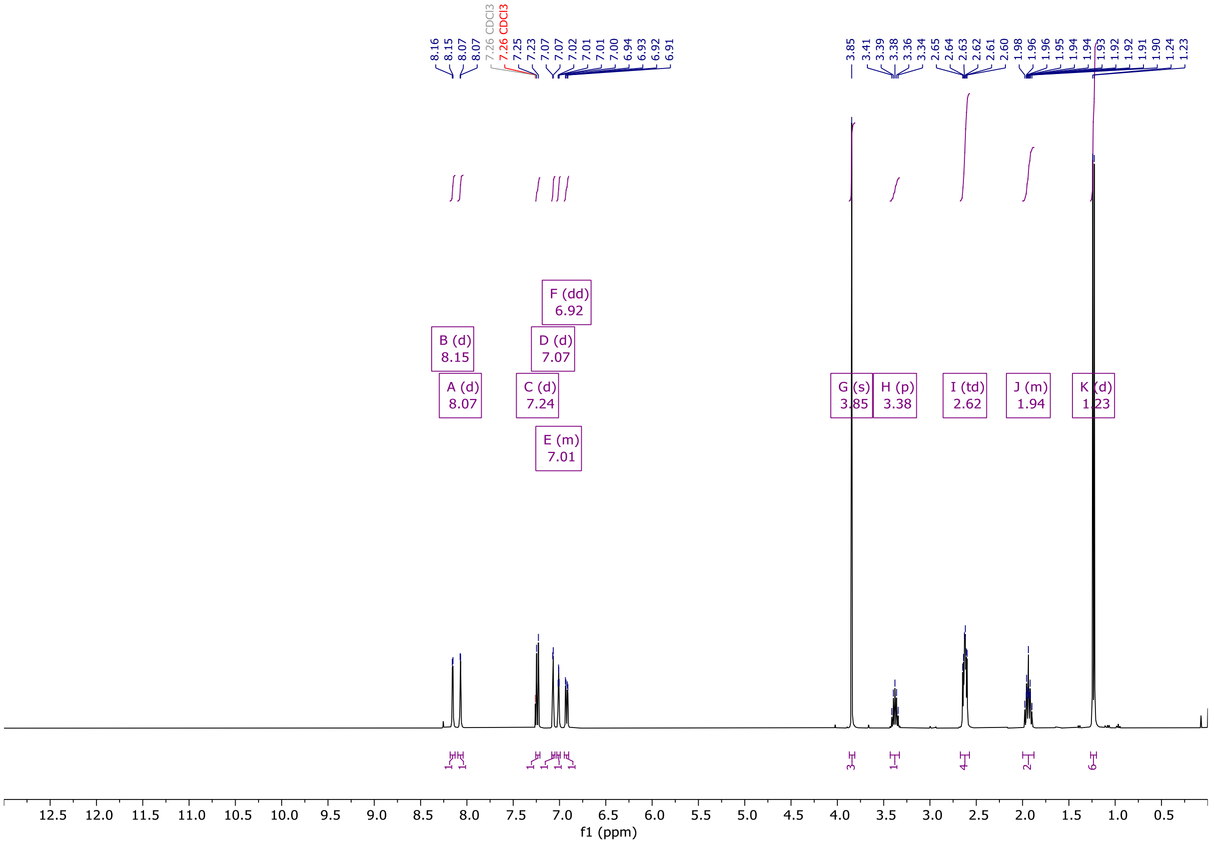

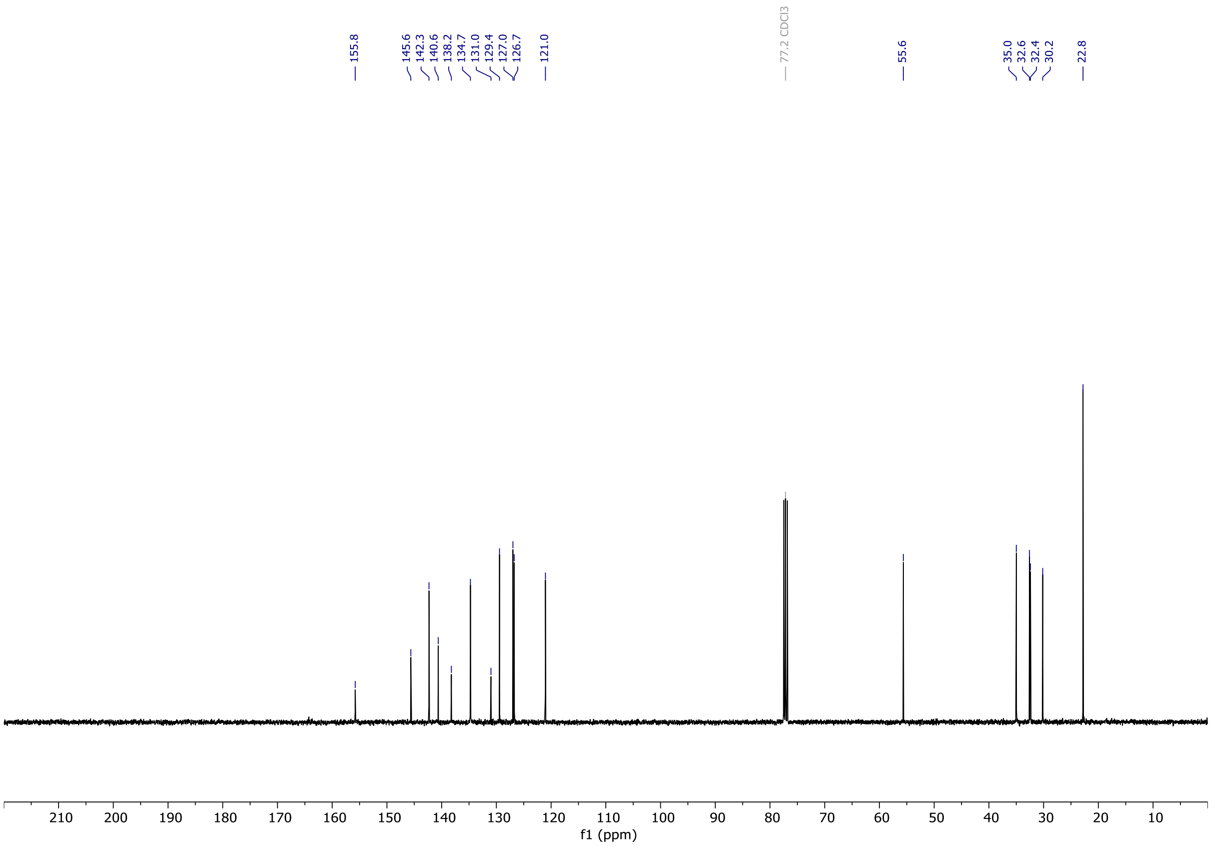

OX04964c

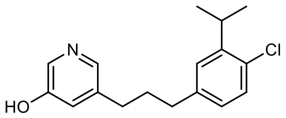

OX04964c

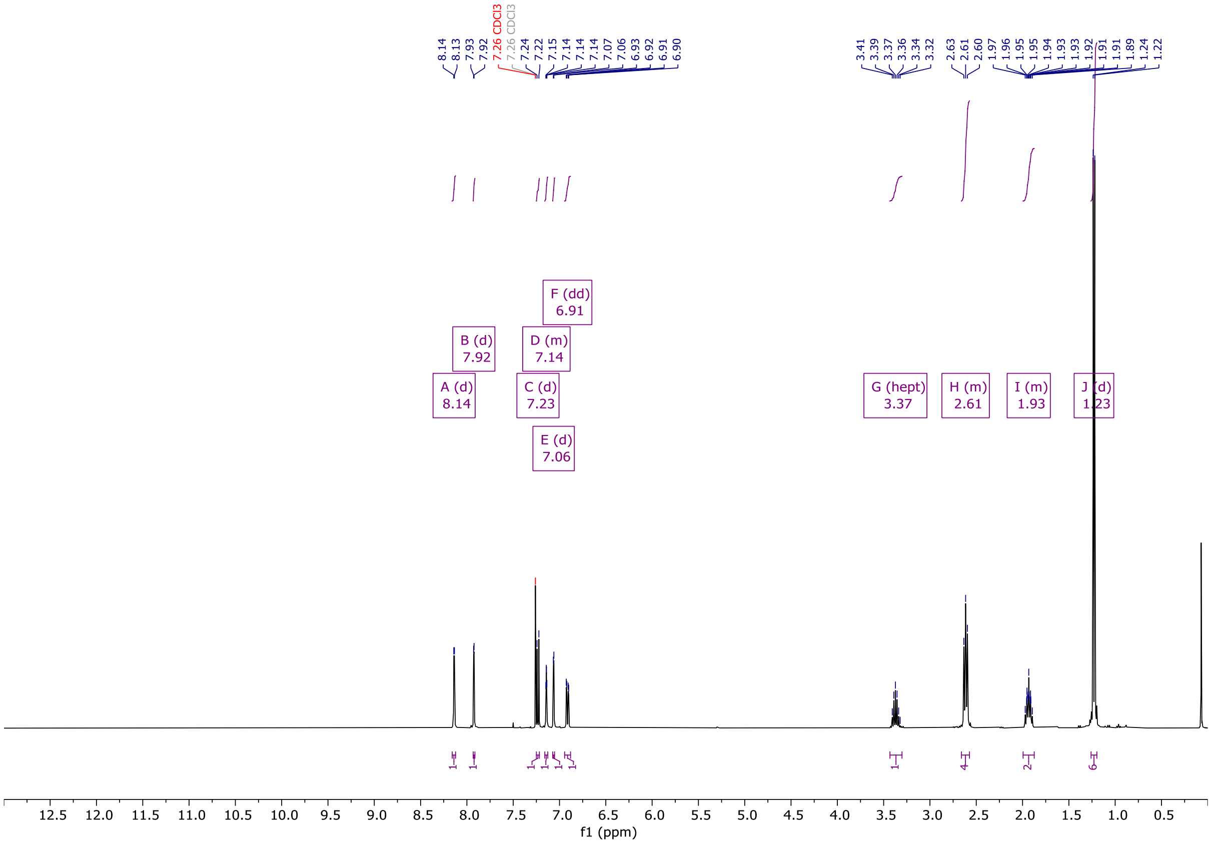

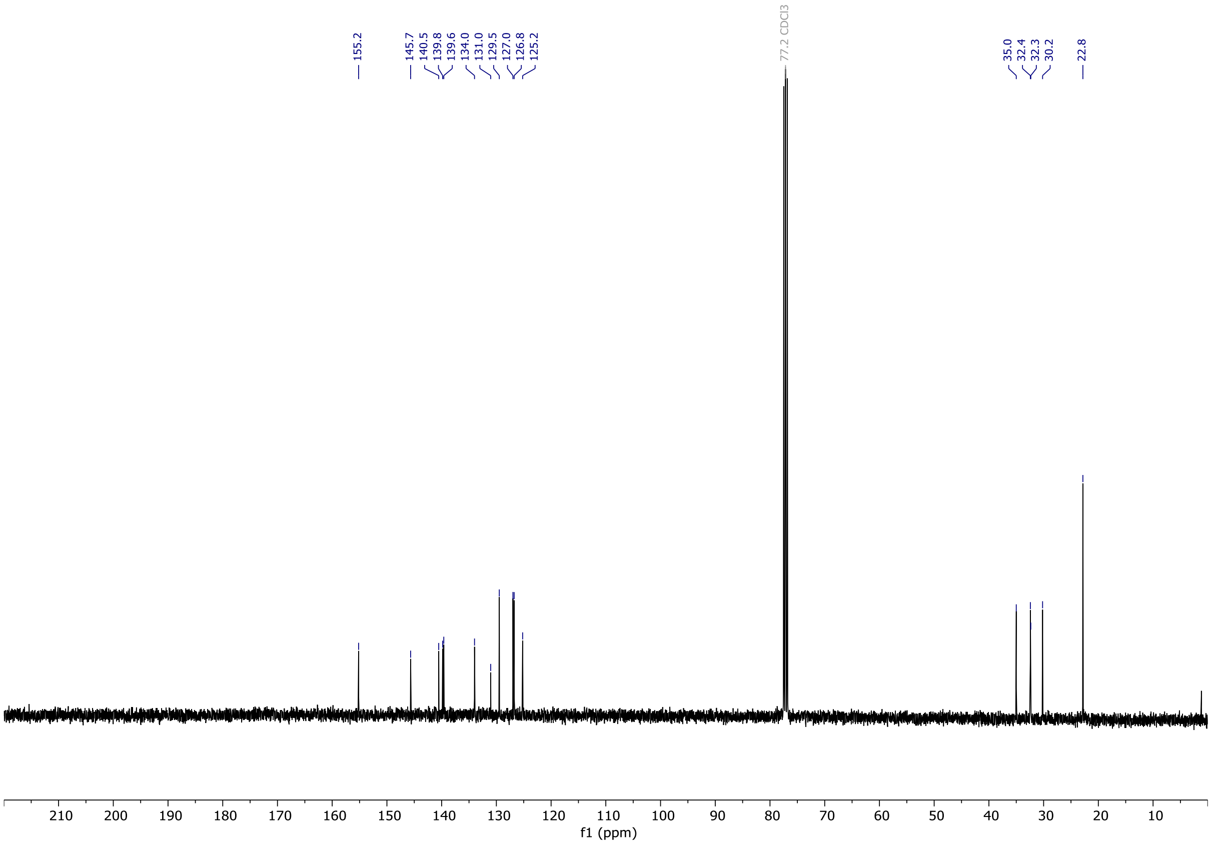

OX04964

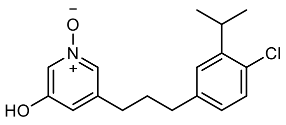

OX04964

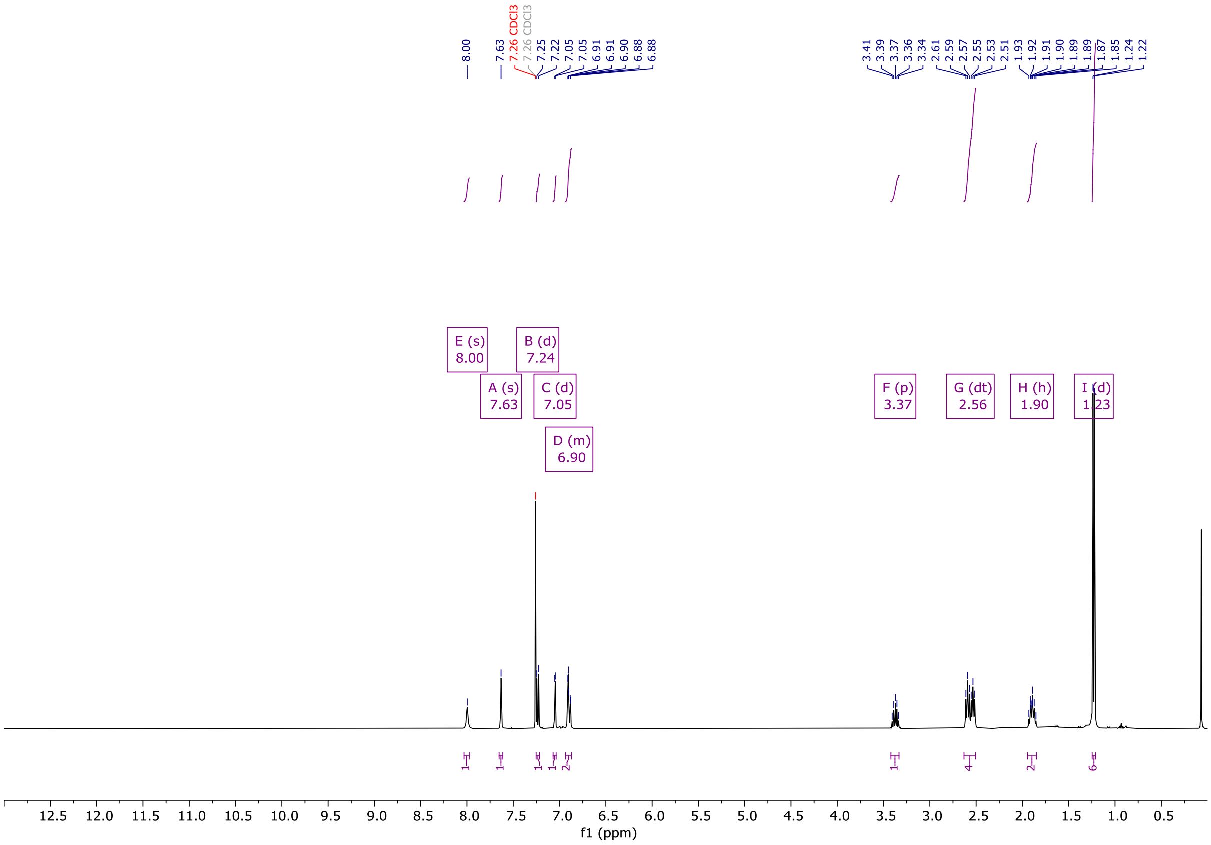

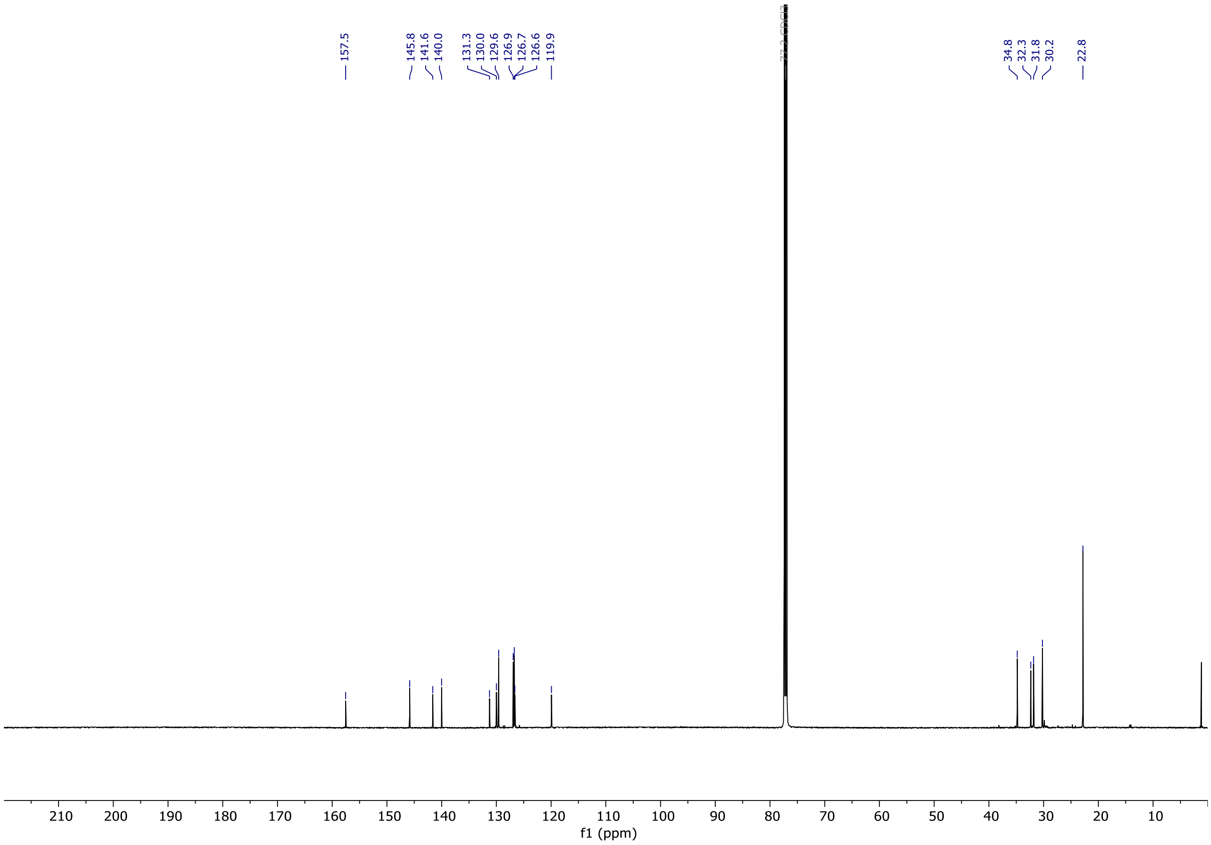

OX04965a

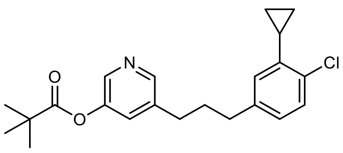

OX04965a

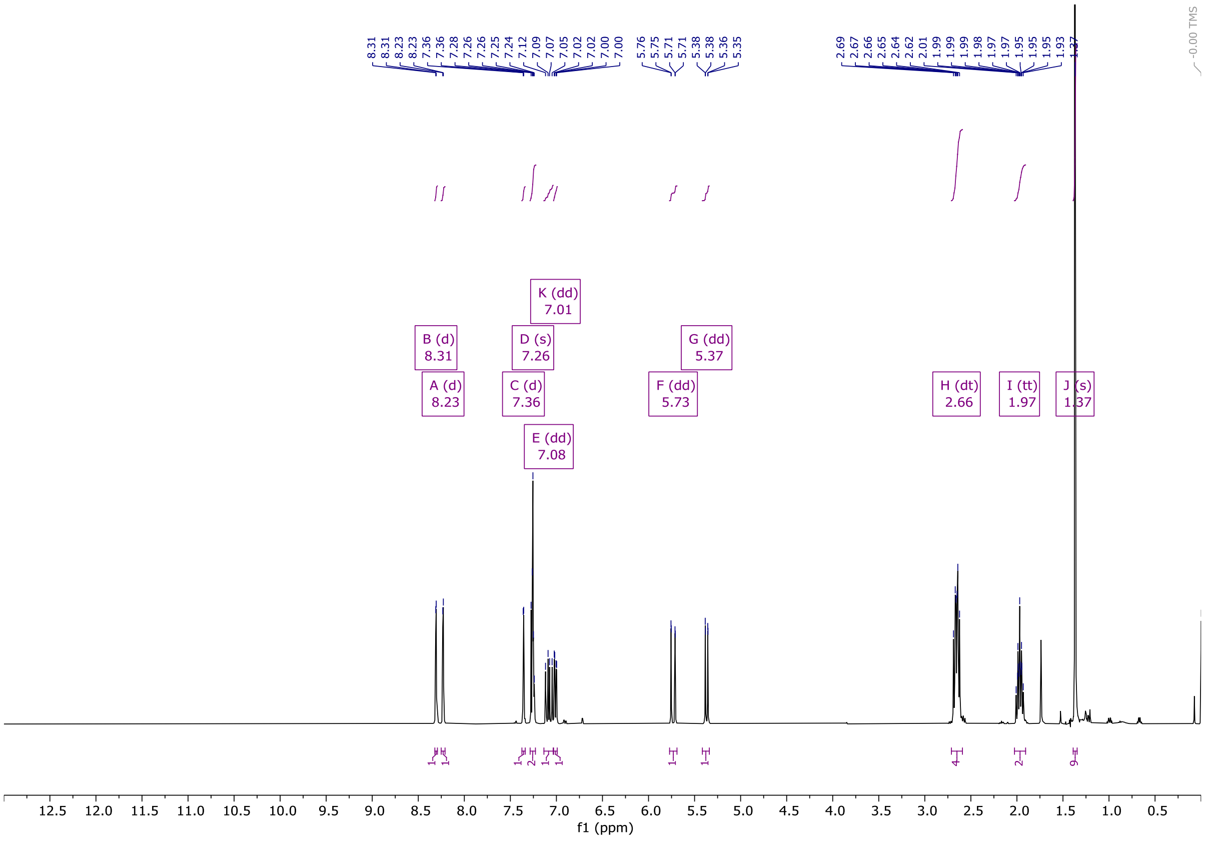

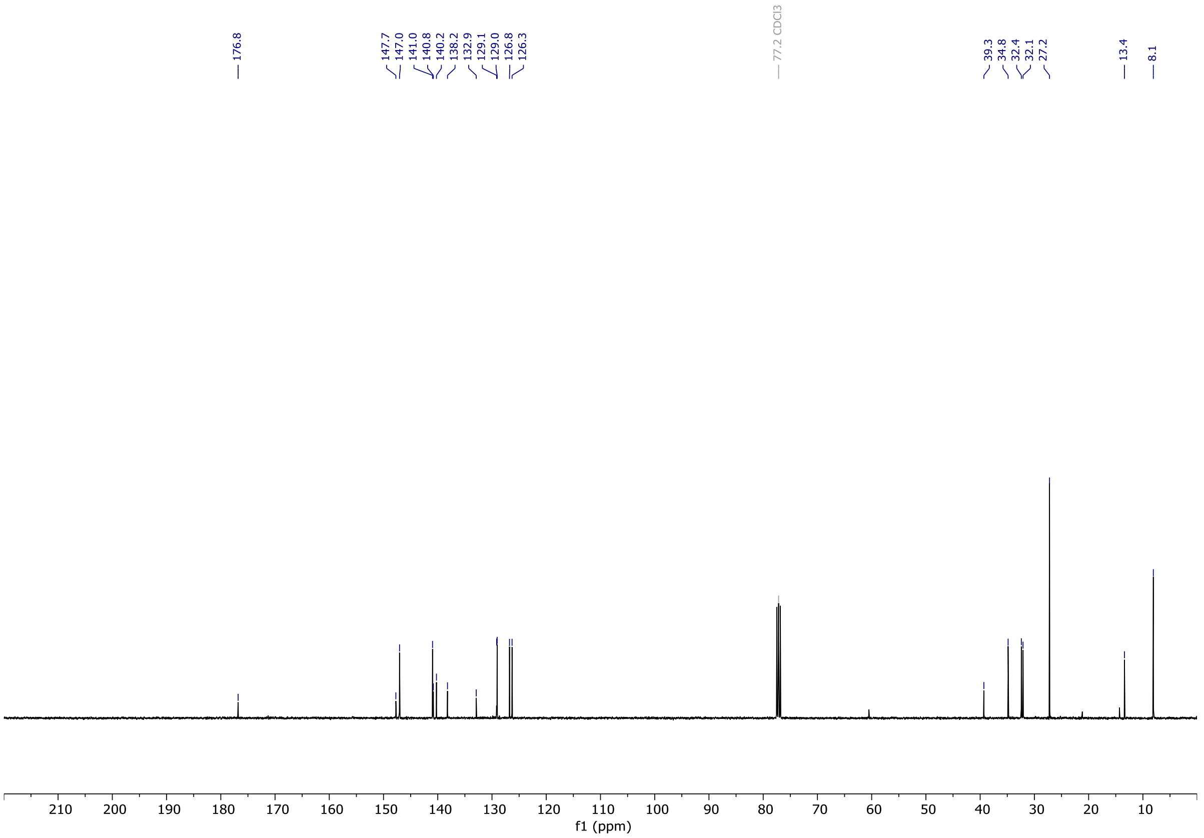

OX04965

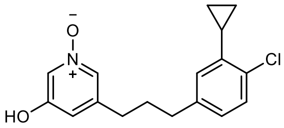

OX04965

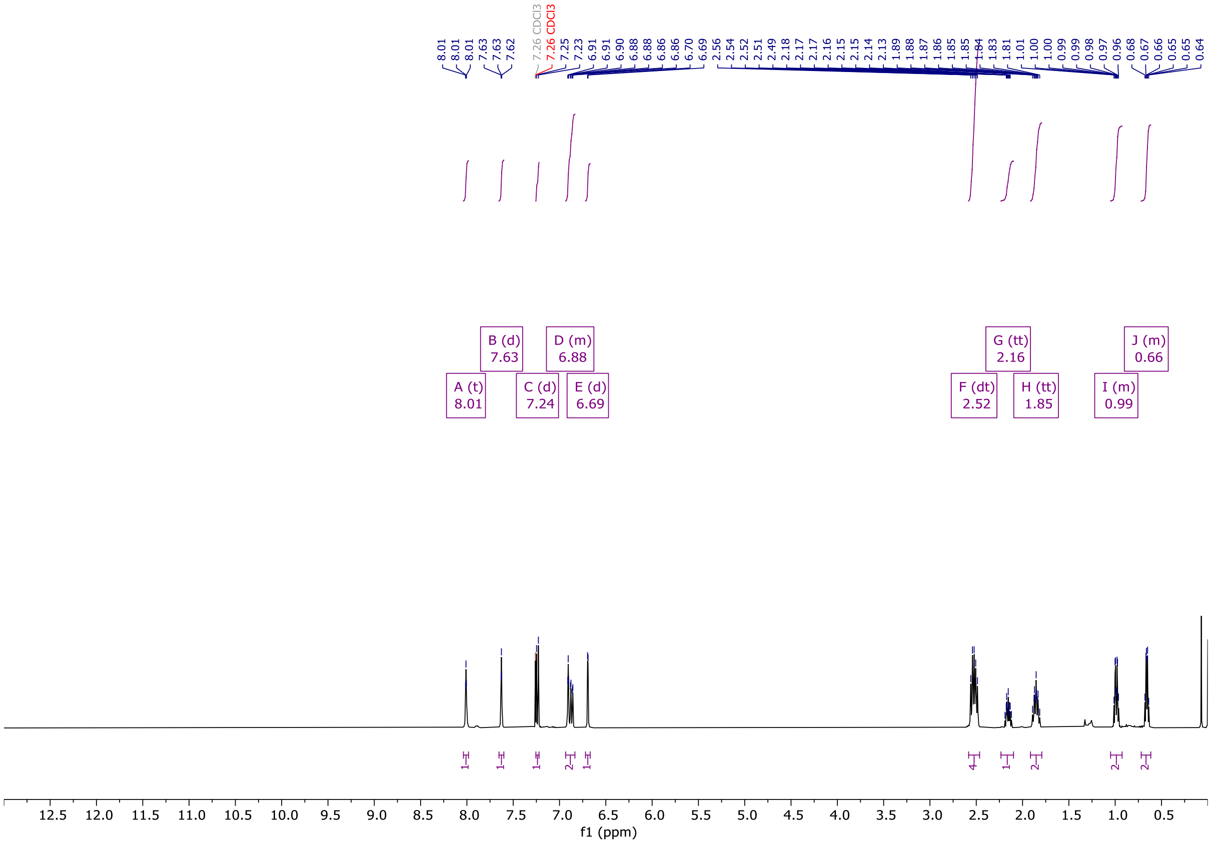

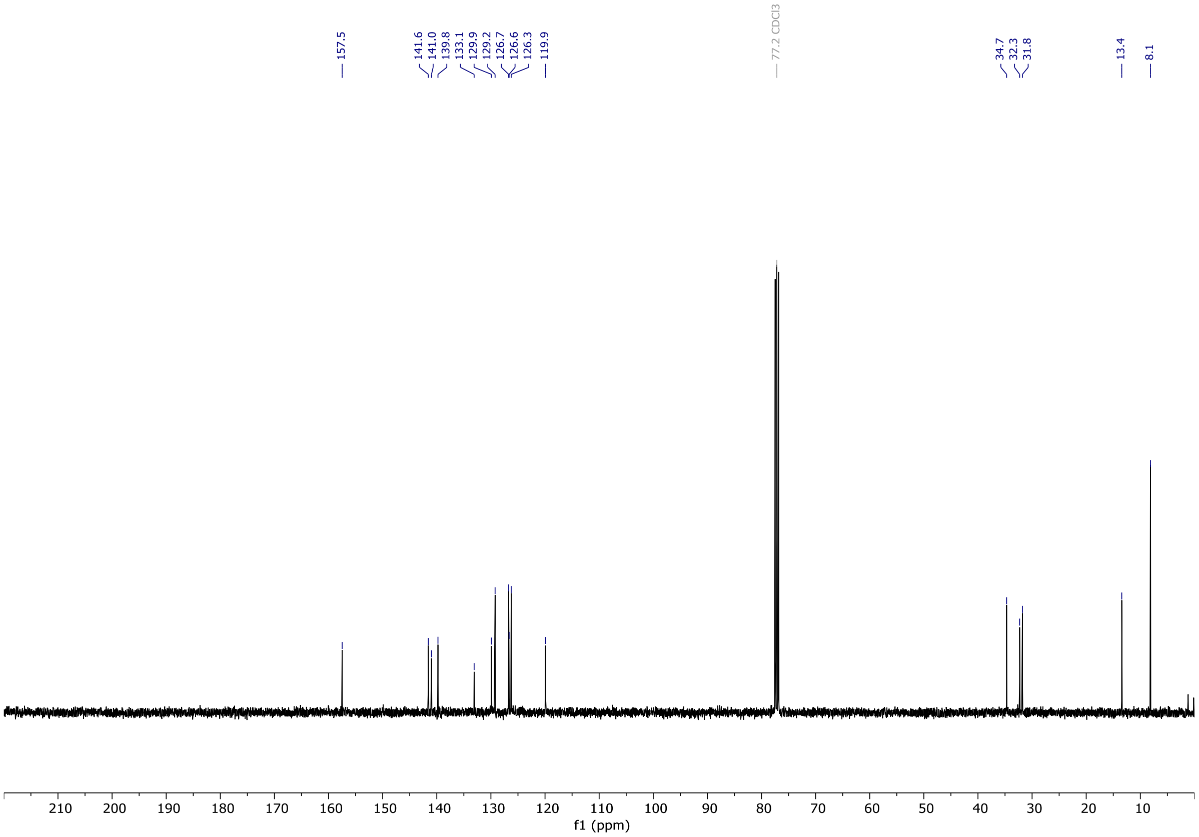

OX04966a

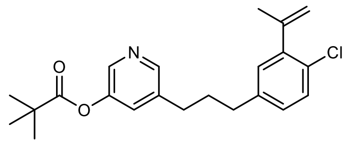

OX04966a

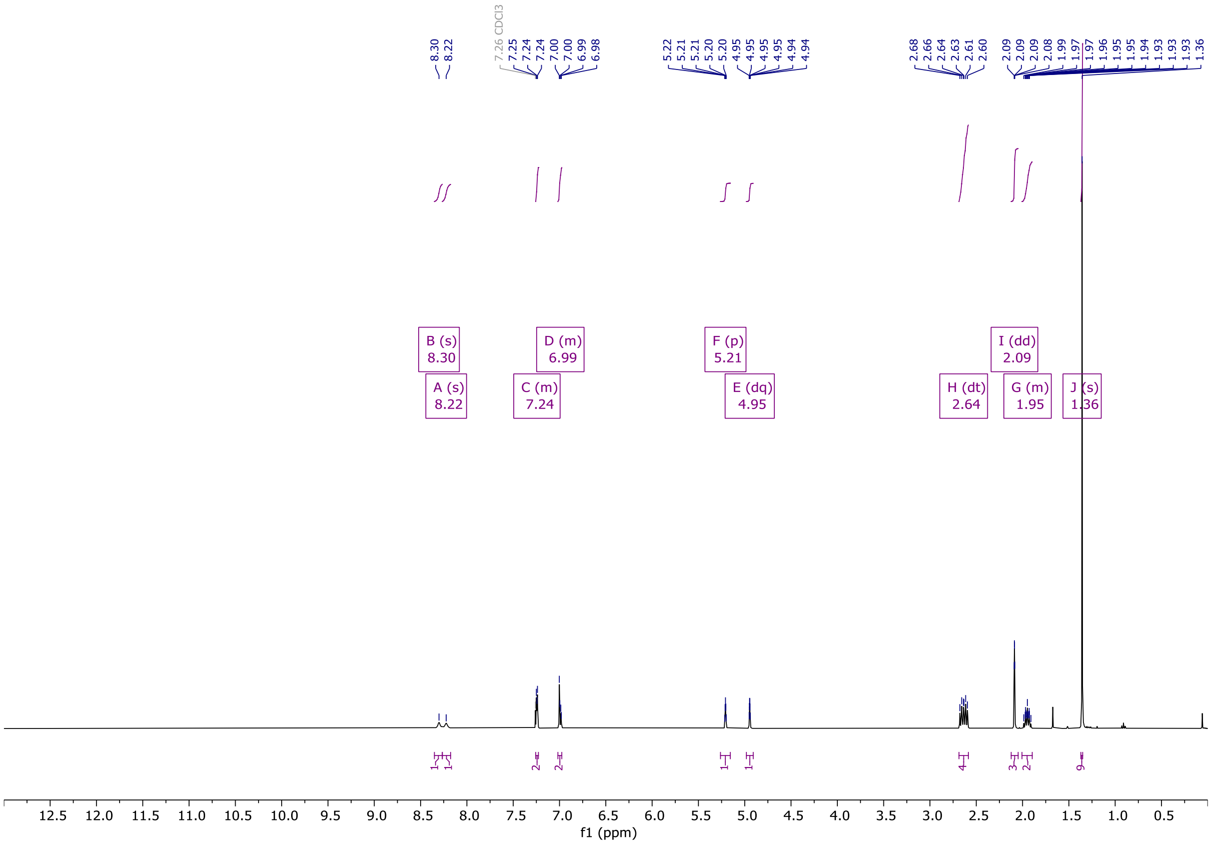

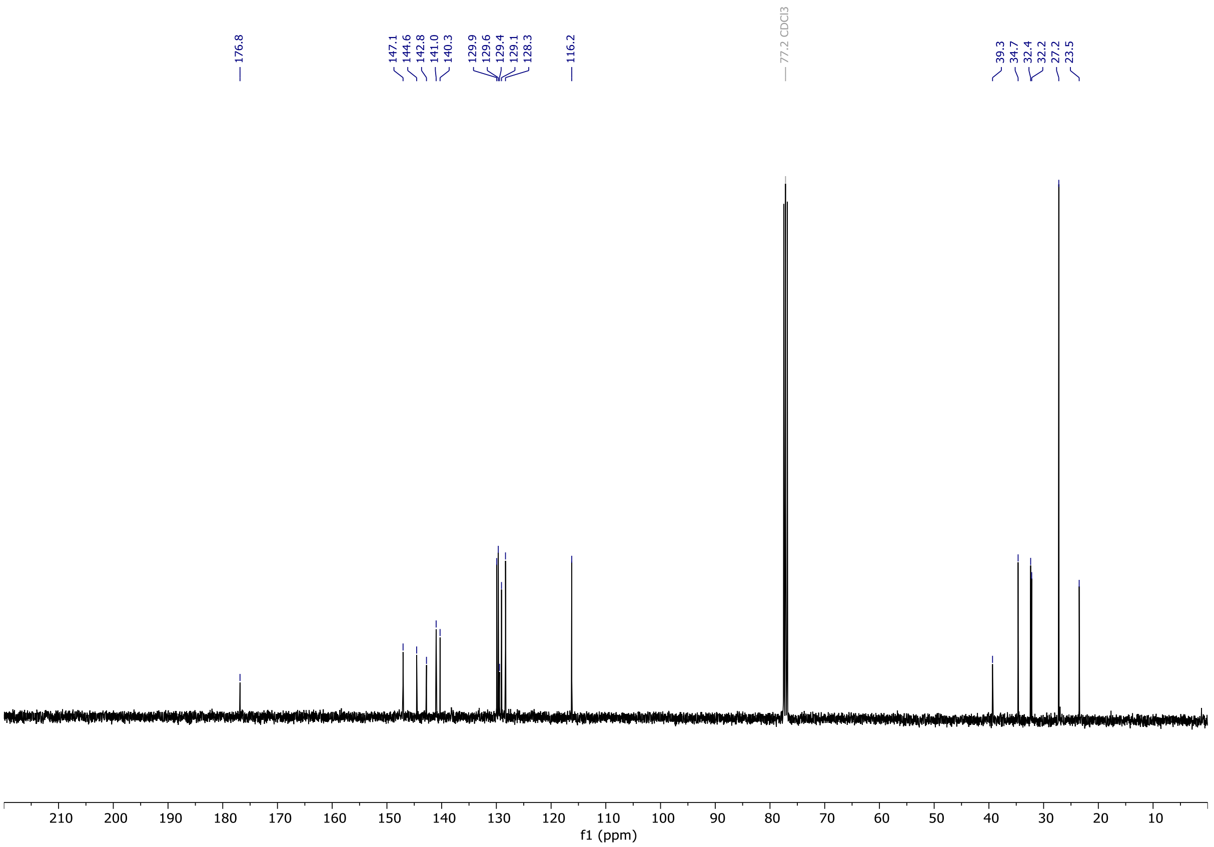

OX04966b

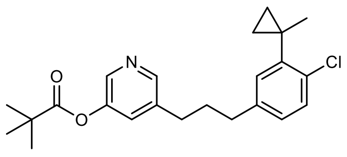

OX04966b

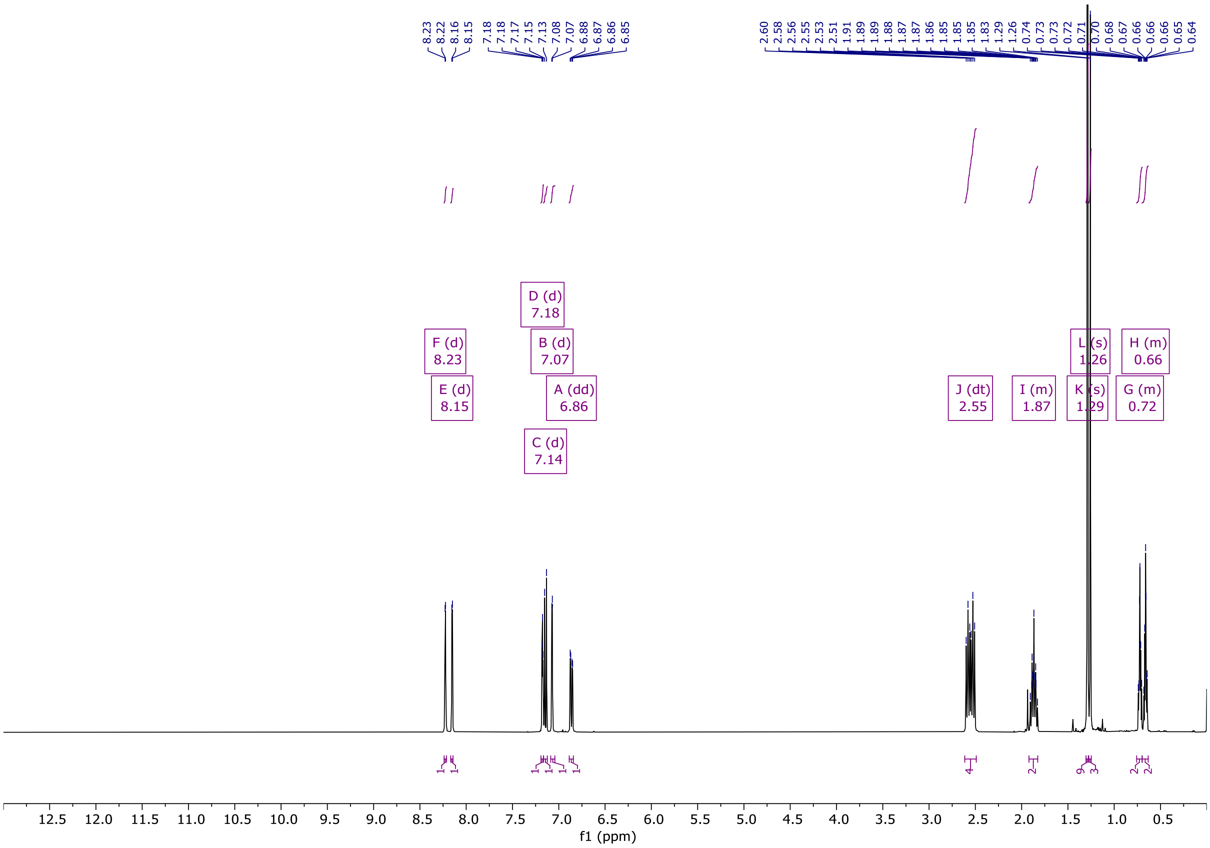

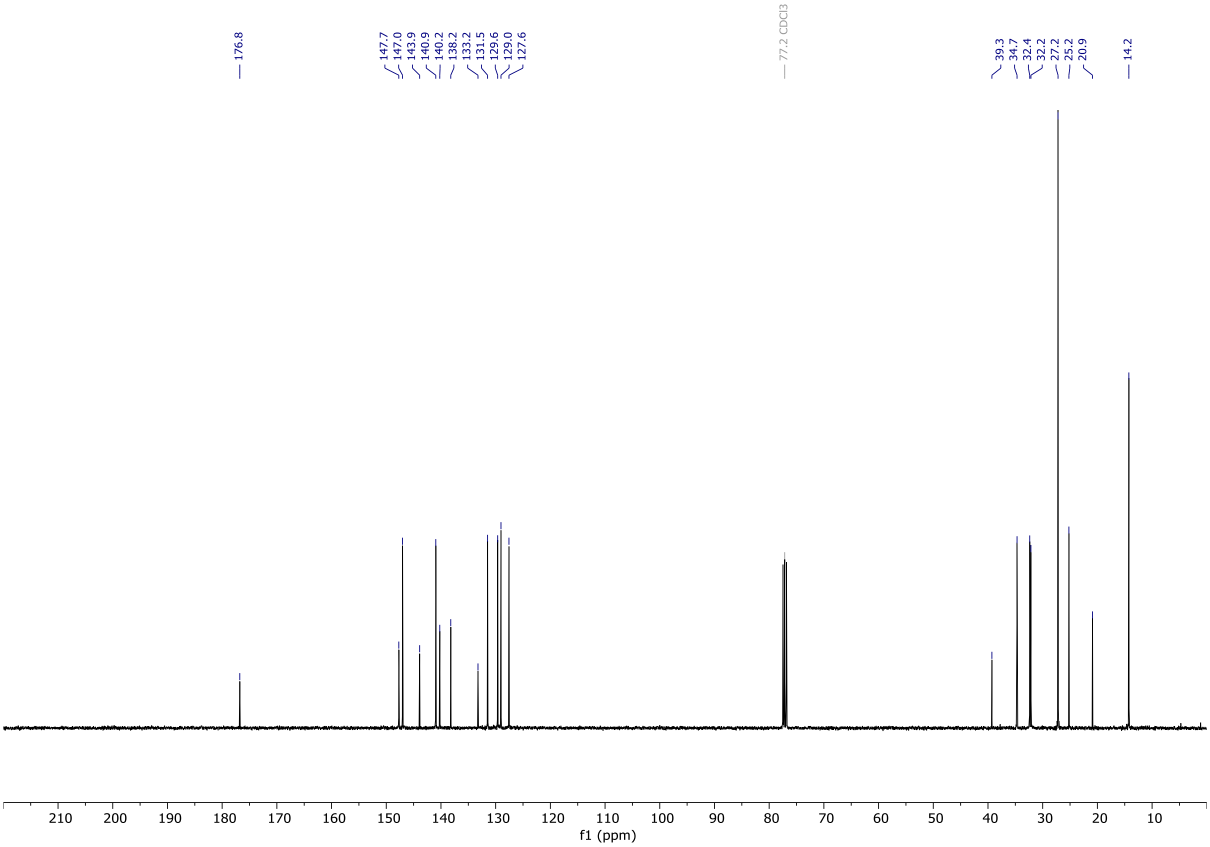

OX04966

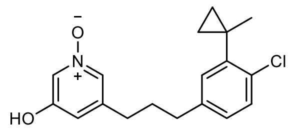

OX04966

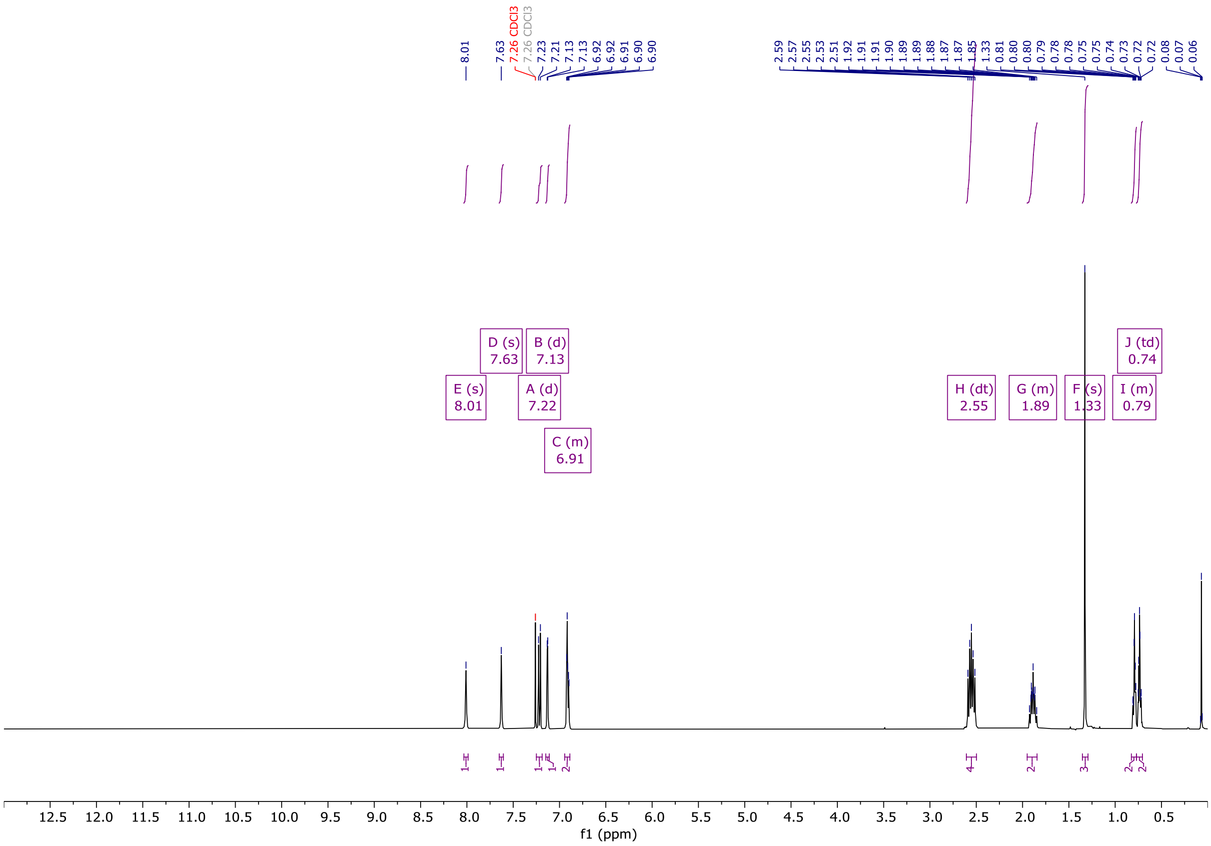

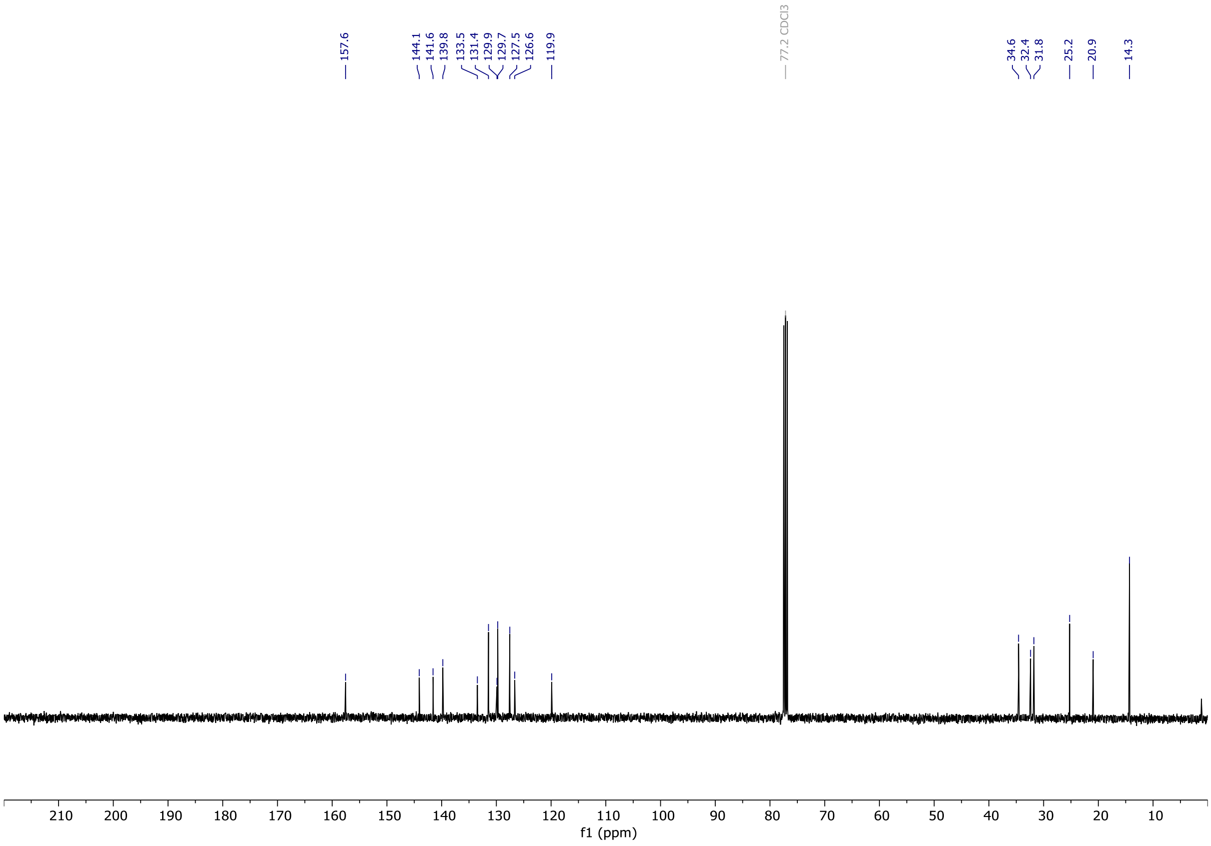

HPLC

OX04539

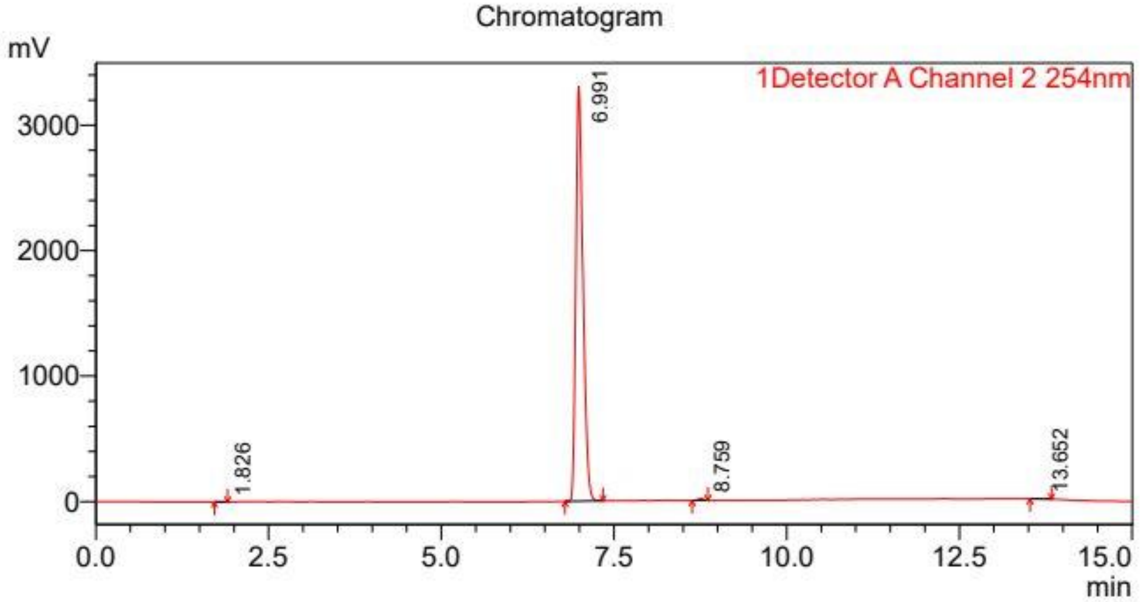

Detector A Channel 2 254nm

Peak#	Ret. Time (min)	Area%
1	1.826	0.096
2	6.991	99.377
3	8.759	0.384
4	13.652	0.143
Total		100.000

OX04540

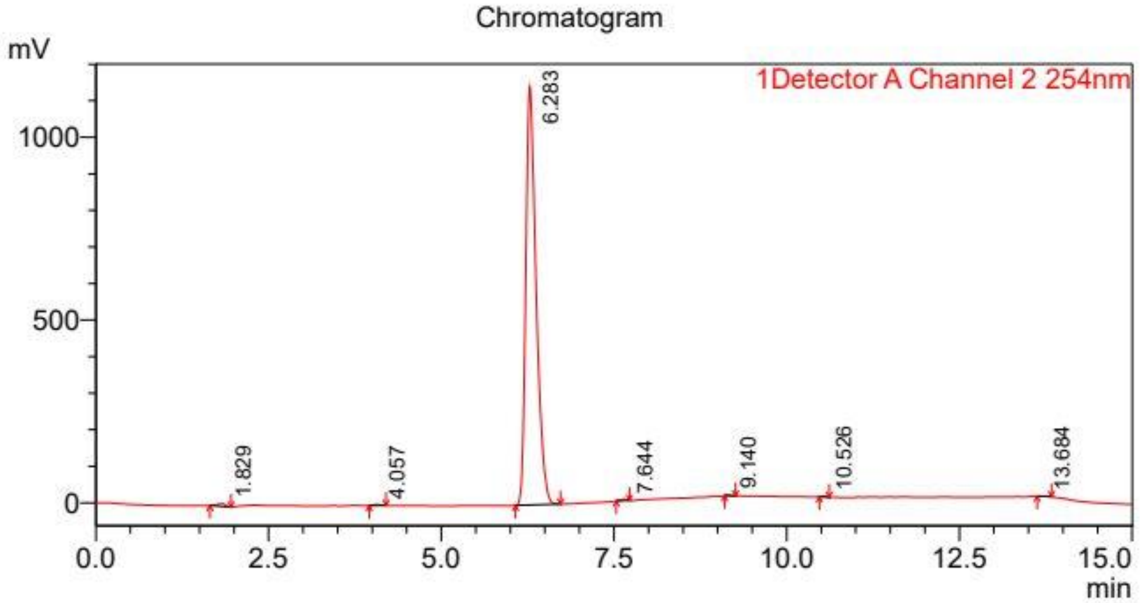

Detector A Channel 2 254nm

Peak#	Ret. Time (min)	Area%
1	1.829	0.506
2	4.057	0.189
3	6.283	98.932
4	7.644	0.178
5	9.140	0.053
6	10.526	0.074
7	13.684	0.067
Total		100.000

OX04954

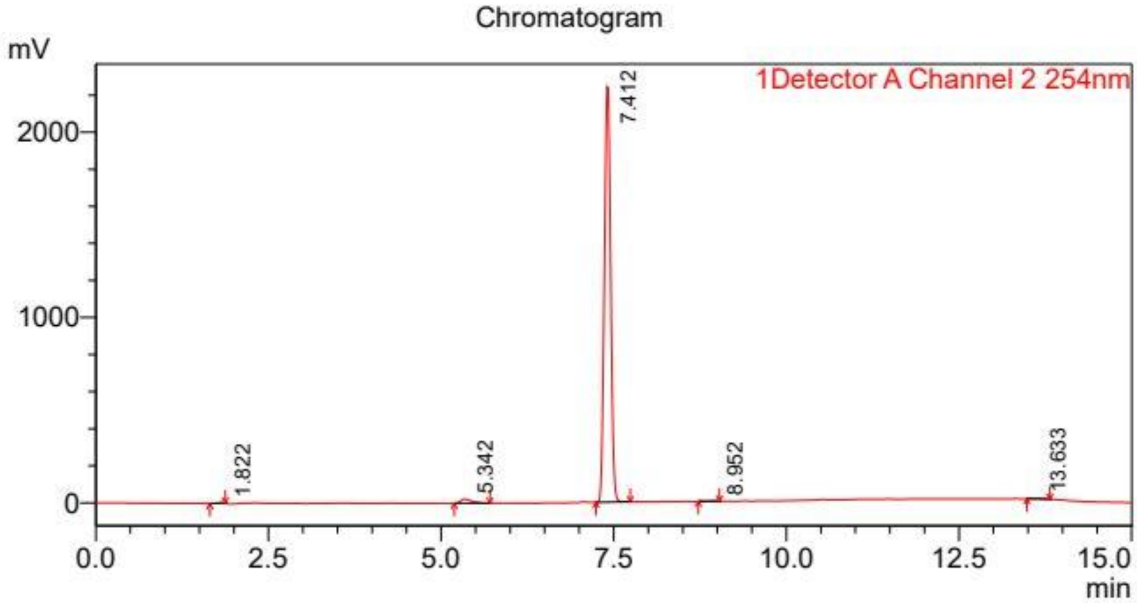

Detector A Channel 2 254nm

Peak#	Ret. Time (min)	Area%
1	1.822	0.185
2	5.342	1.771
3	7.412	97.654
4	8.952	0.150
5	13.633	0.239
Total		100.000

OX04955

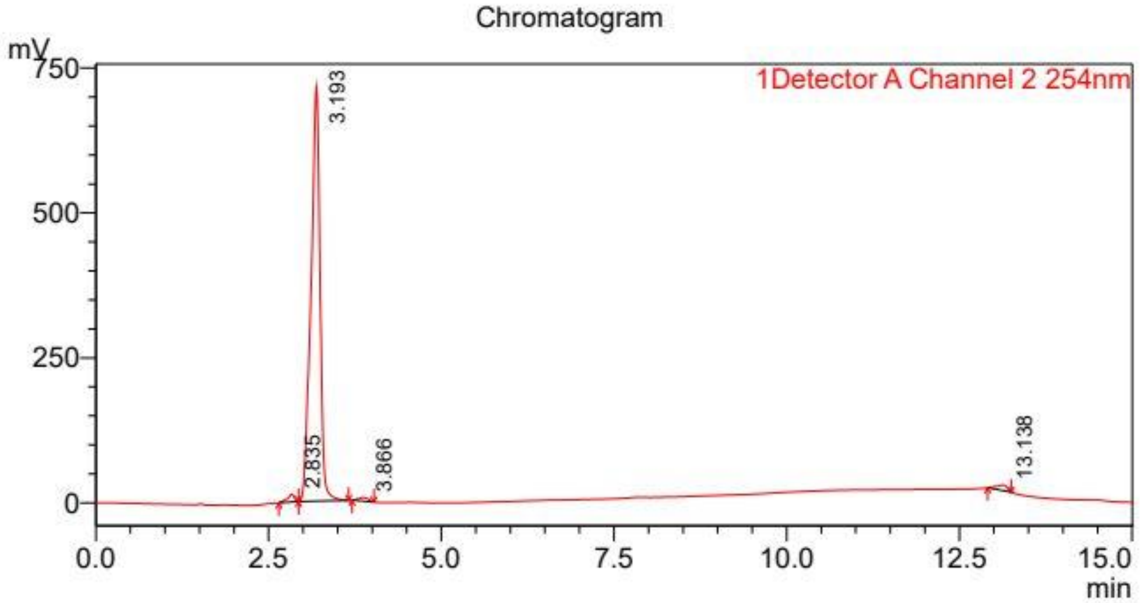

Detector A Channel 2 254nm

Peak#	Ret. Time (min)	Area%
1	2.835	1.399
2	3.193	96.196
3	3.866	0.916
4	13.138	1.489
Total		100.000

OX04956

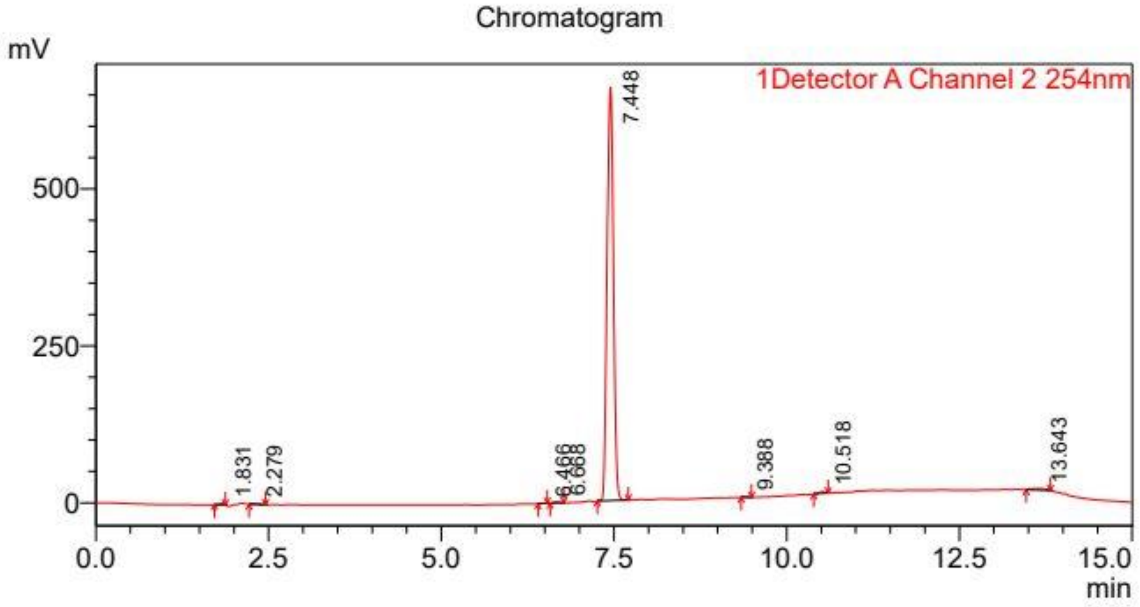

Detector A Channel 2 254nm

Peak#	Ret. Time (min)	Area%
1	1.831	0.230
2	2.279	0.205
3	6.466	0.069
4	6.668	0.234
5	7.448	98.029
6	9.388	0.059
7	10.518	0.368
8	13.643	0.807
Total		100.000

OX04957

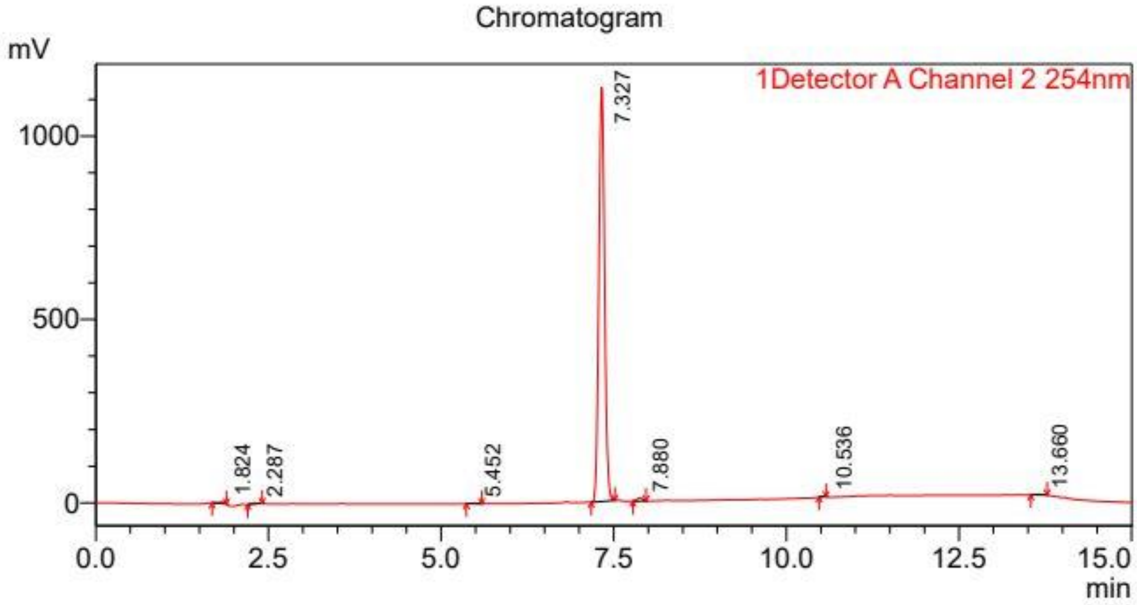

Detector A Channel 2 254nm

Peak#	Ret. Time (min)	Area%
1	1.824	0.410
2	2.287	0.381
3	5.452	0.164
4	7.327	98.053
5	7.880	0.693
6	10.536	0.082
7	13.660	0.217
Total		100.000

OX04958

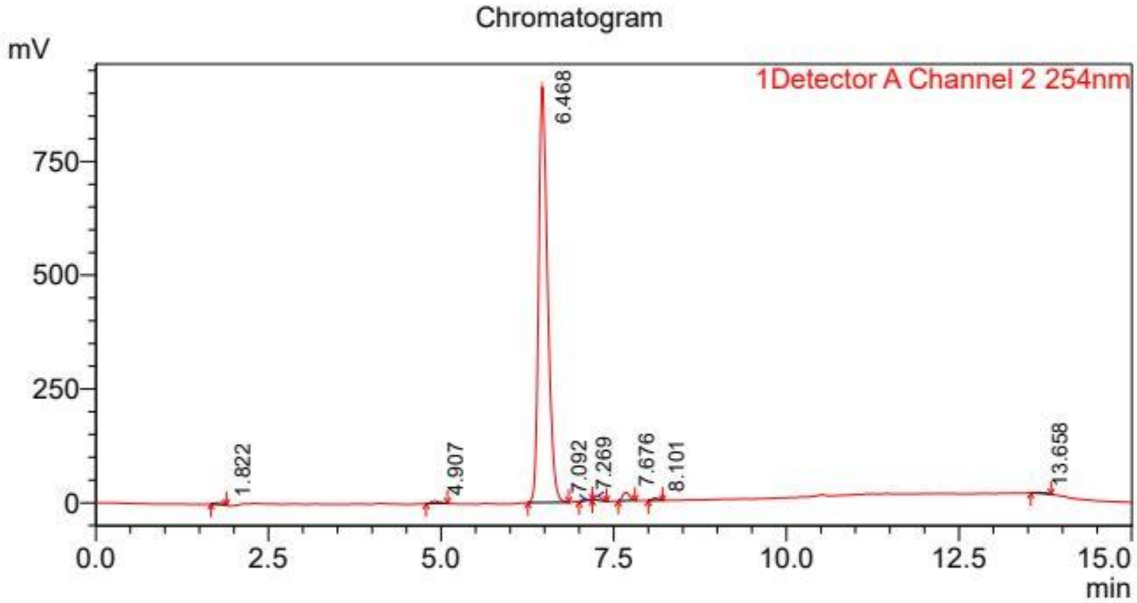

Detector A Channel 2 254nm

Peak#	Ret. Time (min)	Area%
1	1.822	0.444
2	4.907	0.558
3	6.468	96.016
4	7.092	0.192
5	7.269	0.638
6	7.676	1.503
7	8.101	0.419
8	13.658	0.231
Total		100.000

OX04959

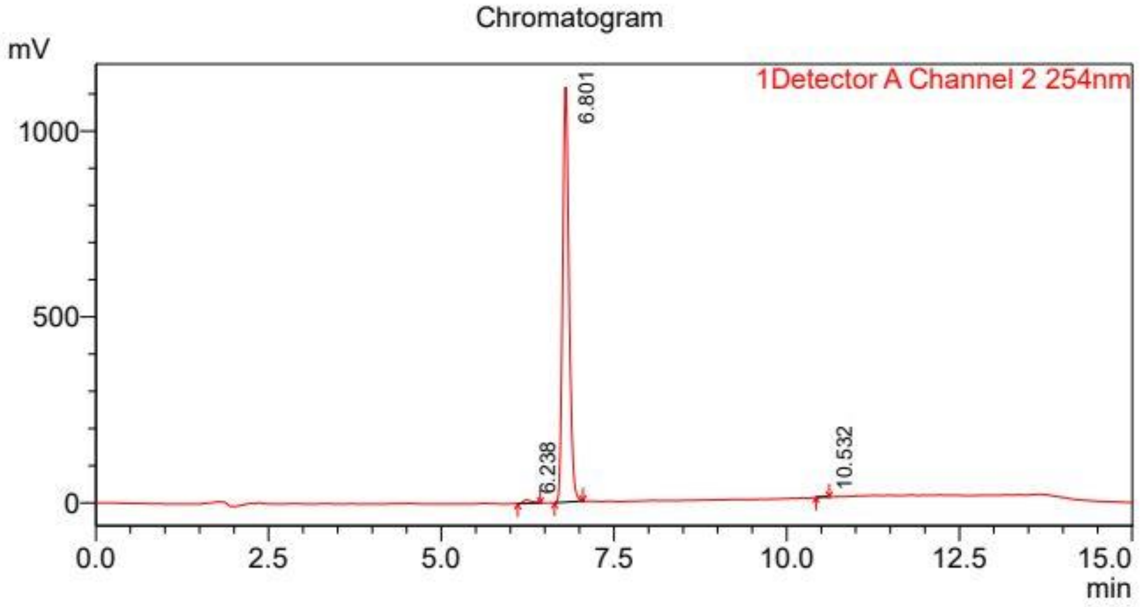

Detector A Channel 2 254nm

Peak#	Ret. Time (min)	Area%
1	6.238	1.055
2	6.801	98.719
3	10.532	0.226
Total		100.000

OX04960

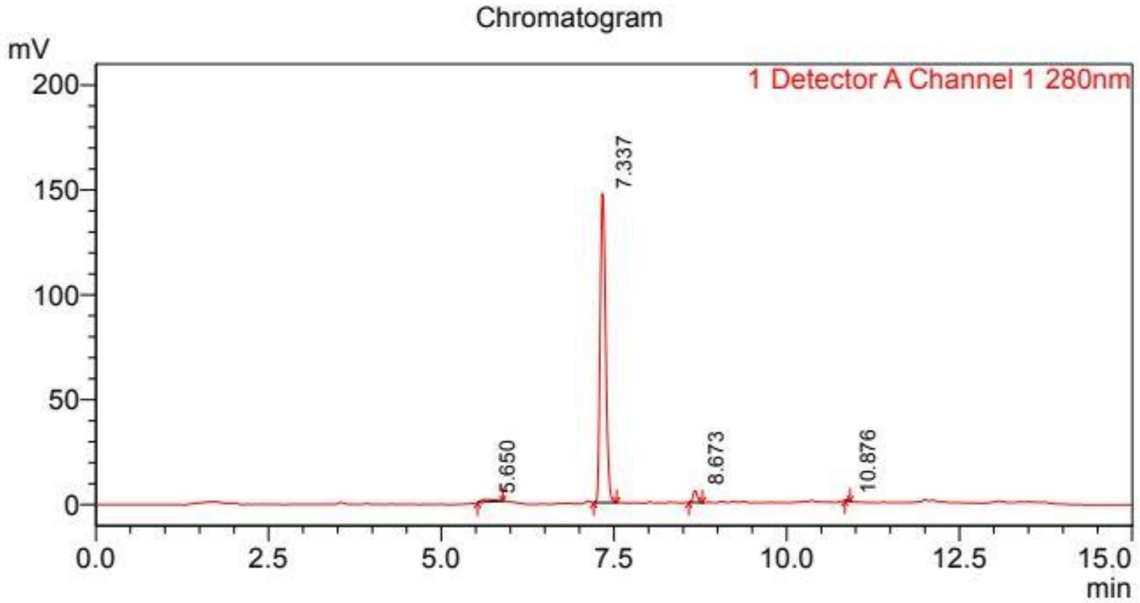

Detector A Channel 1 280nm

Peak#	Ret. Time (min)	Area%
1	5.650	1.857
2	7.337	95.238
3	8.673	2.756
4	10.876	0.148
Total		100.000

OX04961

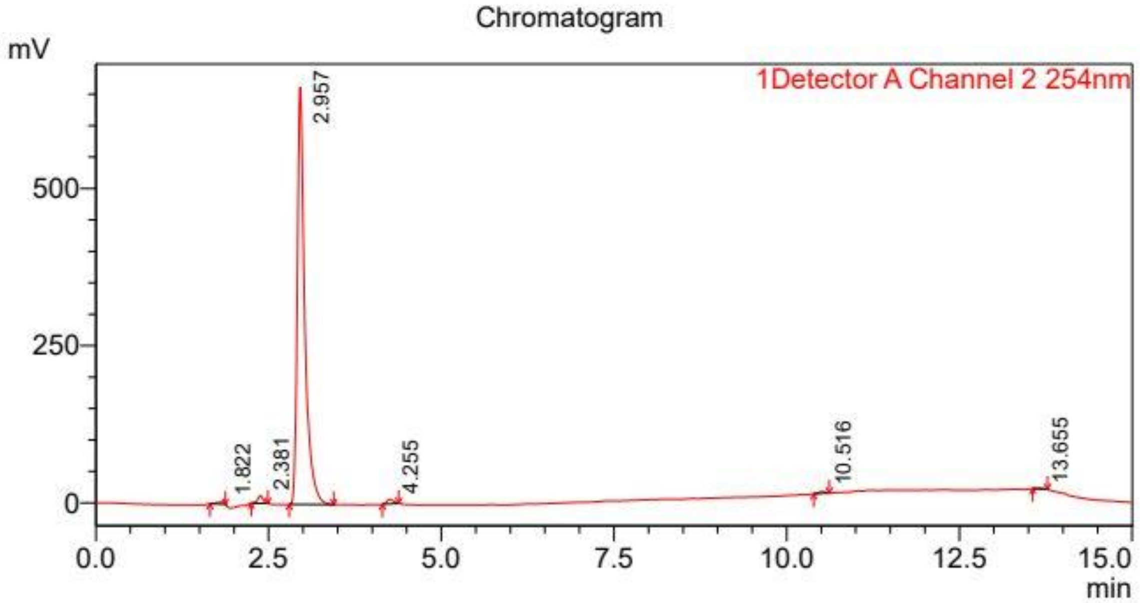

Detector A Channel 2 254nm

Peak#	Ret. Time (min)	Area%
1	1.822	0.467
2	2.381	1.245
3	2.957	96.753
4	4.255	0.961
5	10.516	0.347
6	13.655	0.227
Total		100.000

OX04962

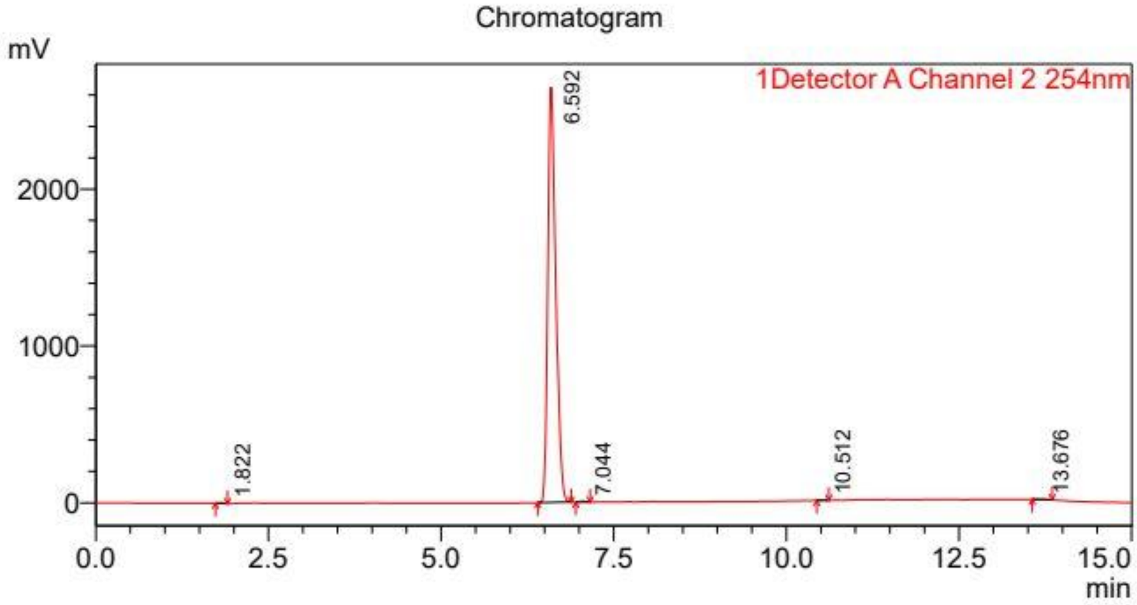

Detector A Channel 2 254nm

Peak#	Ret. Time (min)	Area%
1	1.822	0.112
2	6.592	99.524
3	7.044	0.176
4	10.512	0.056
5	13.676	0.131
Total		100.000

OX04963

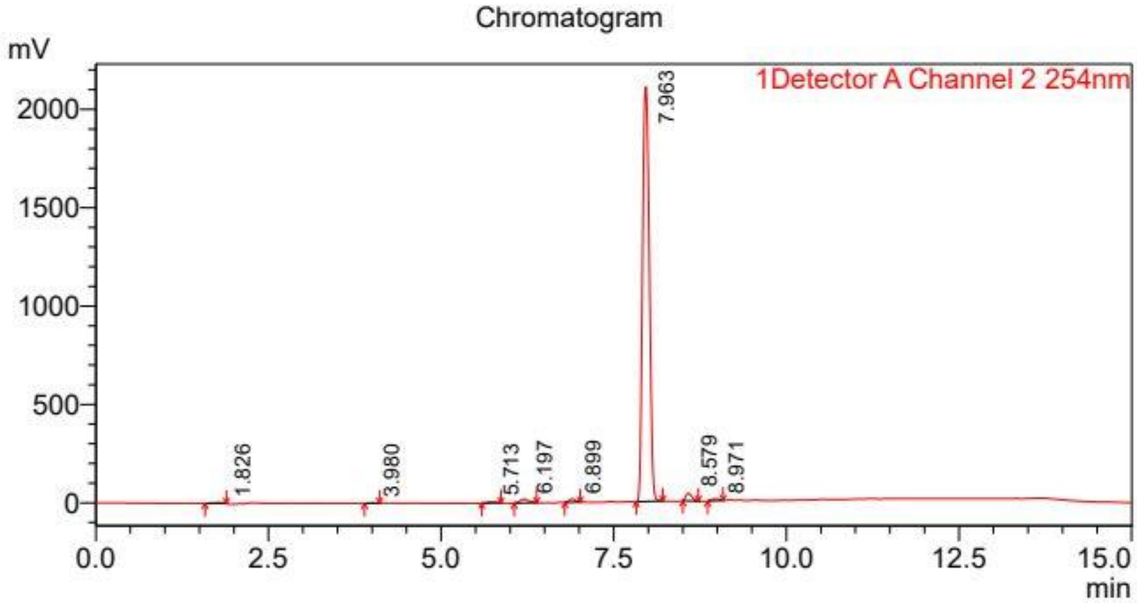

Detector A Channel 2 254nm

Peak#	Ret. Time (min)	Area%
1	1.826	0.364
2	3.980	0.109
3	5.713	0.349
4	6.197	1.096
5	6.899	0.691
6	7.963	95.281
7	8.579	1.595
8	8.971	0.515
Total		100.000

OX04964

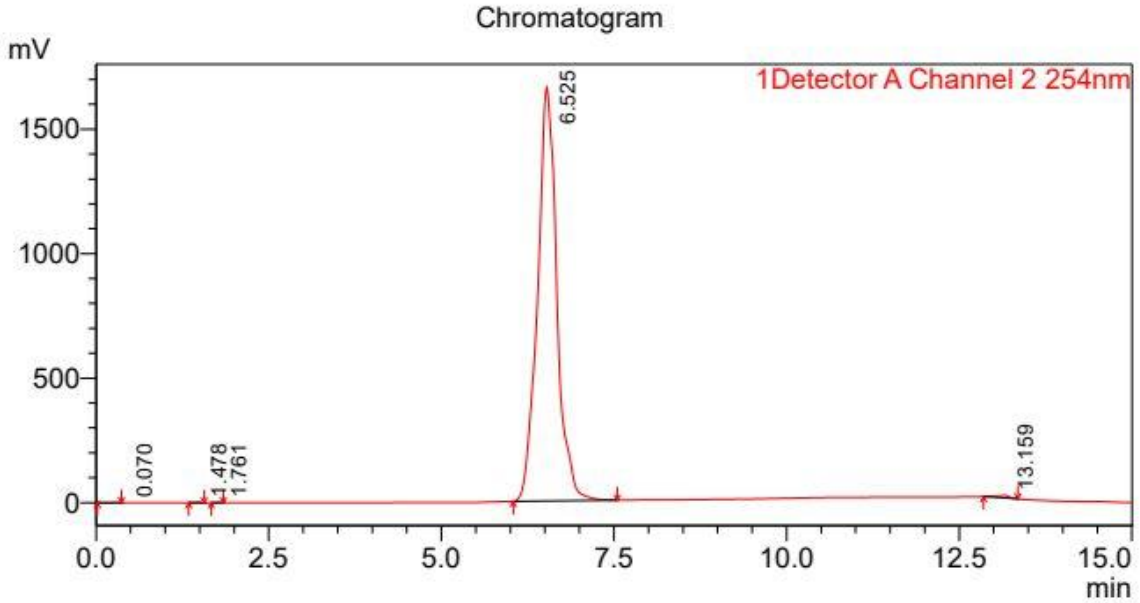

Detector A Channel 2 254nm

Peak#	Ret. Time (min)	Area%
1	0.070	0.005
2	1.478	0.067
3	1.761	0.012
4	6.525	99.482
5	13.159	0.434
Total		100.000

OX04965

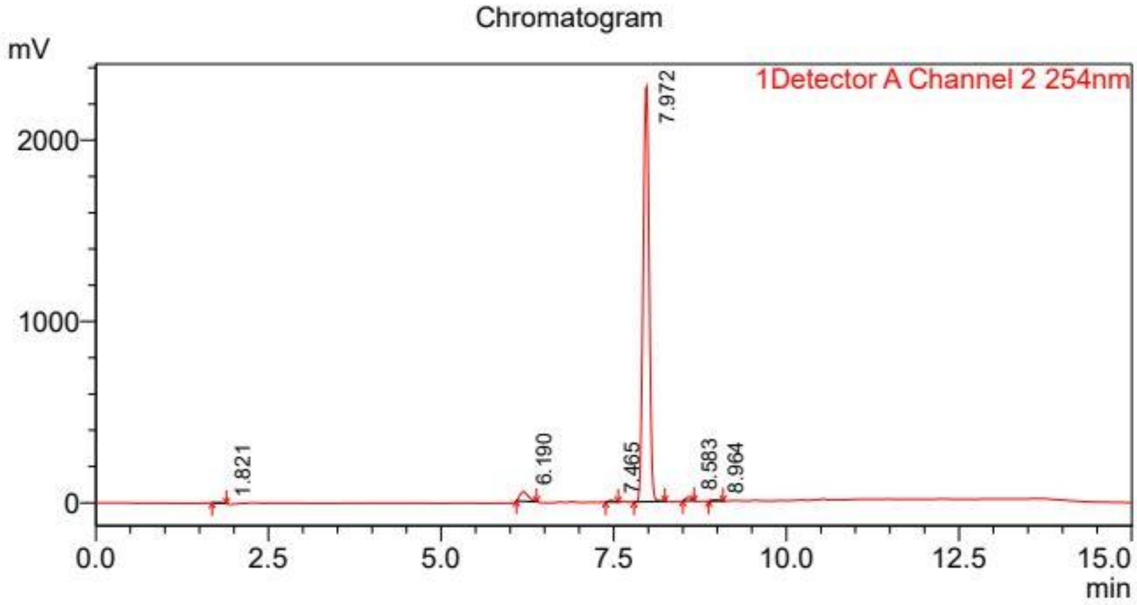

Detector A Channel 2 254nm

Peak#	Ret. Time (min)	Area%
1	1.821	0.237
2	6.190	3.121
3	7.465	0.355
4	7.972	95.148
5	8.583	0.790
6	8.964	0.349
Total		100.000

OX04966

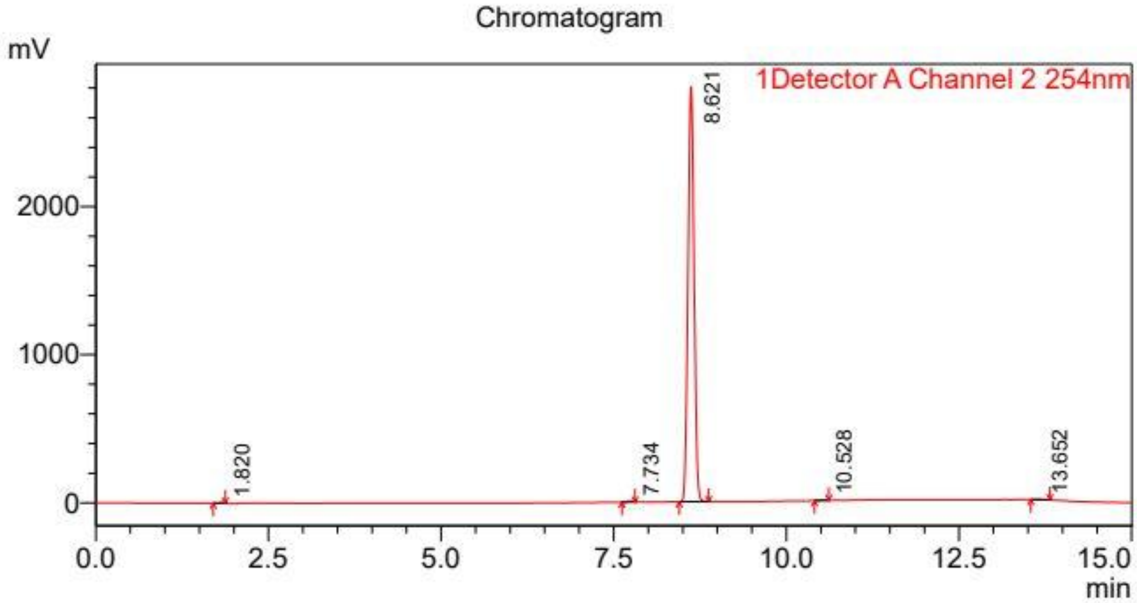

Detector A Channel 2 254nm

Peak#	Ret. Time (min)	Area%
1	1.820	0.118
2	7.734	0.234
3	8.621	99.426
4	10.528	0.094
5	13.652	0.128
Total		100.000
